# Cooperative copper-squaramide catalysis for the enantioselective N–H insertion reaction with sulfoxonium ylides†

**DOI:** 10.1039/d1sc00979f

**Published:** 2021-04-26

**Authors:** Lucas G. Furniel, Radell Echemendía, Antonio C. B. Burtoloso

**Affiliations:** São Carlos Institute of Chemistry, University of São Paulo São Carlos SP CEP 13560-970 Brazil antonio@iqsc.usp.br

## Abstract

The first examples of a highly efficient and enantioselective carbene-mediated insertion reaction, from a sulfur ylide, are described. By way of a catalytic asymmetric insertion reaction into N–H bonds from carbonyl sulfoxonium ylides and anilines, using a copper-bifunctional squaramide cooperative catalysis approach, thirty-seven α-arylglycine esters were synthesized in enantiomeric ratios up to 92 : 8 (99 : 1 after a single recrystallization) and reaction yields ranging between 49–96%. Furthermore, the protocol benefits from quick reaction times and is conducted in a straightforward manner.

## Abstract 2

The first examples of a highly efficient and enantioselective carbene-mediated insertion reaction, from a sulfur ylide, are described.

## Graphical abstract

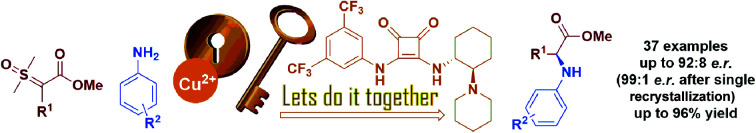

## Introduction

Unnatural amino acids are a particularly important class of molecules, owing to their diverse array of biological activities, being present in pharmaceuticals and also serving as versatile building blocks, especially for peptide synthesis.^1–5^ Amongst these, arylglycines are a peculiar class of building blocks, found in several bioactive compounds, such as antibiotics, agrochemicals, protein kinase C activators and dengue virus inhibitors.^6–11^ One of the most straightforward methodologies to prepare enantioenriched unnatural amino acids is the asymmetric N–H insertion reaction from diazo compounds.^12,13^ This strategy has been perfected over the last fifteen years and a diverse array of diazo compounds and nitrogen nucleophiles can now be coupled with high enantioselectivities.^14–18^ The most recent achievement in this field was described by Zhou^18^ in 2019, where a combination of a copper(i)-scorpionate complex, together with an optically active thiourea, led to high enantioselectivities when strongly coordinating aliphatic amines were reacted in the presence of diazo esters. Despite these great advances, each catalytic system described so far is still only efficient for a specific combination of substrates. For example, an asymmetric version to access α-arylamino phenylglycine derivatives in high enantioselectivities has yet to be discovered.^12,19–25^ Furthermore, notwithstanding its versatility and widespread use in academia, diazo compounds are still less attractive in industry due to their thermal instability, the release of nitrogen gas and the toxicity of diazomethane (a reagent necessary in the synthesis of some diazo compounds).^26–28^ Therefore, seeking viable, safer alternatives to the use of these highly popular synthetic reactants, especially in large-scale preparations is highly desirable. In the past few years, sulfur ylides have emerged as potential surrogates for diazocarbonyl compounds due to their enhanced thermal stability and similar reactivity (for example, as carbene precursors).^29,30^ While these advances have been realized, their use in enantioselective catalytic transformations is still in its infancy when compared to diazo compounds, especially in insertion reactions mediated by carbenes.^31,32^

Seminal studies of X–H insertion reactions mediated by rhodium catalysis with sulfoxonium ylides was disclosed by Baldwin in 1993,^33^ and then latterly in the 2000s^33–35^ by Mangion at Merck with iridium and gold catalysis. Mangion's contribution stablished the first general catalysts for carbene reactions using sulfoxonium ylides, and could also be demonstrated on scale-up in industry. The preference of using sulfoxonium instead of sulfonium ylides is attributed to the better stability, simpler preparation in most cases and by the fact that a sulfoxide is released (generally dimethylsulfoxide) in place of a sulfide (dimethyl or diphenyl sulfide). Very recently, our group demonstrated that the use of inexpensive copper(ii) salts is also efficient for insertion reactions (N–H) with sulfur ylides.^36^

**Scheme 1:**
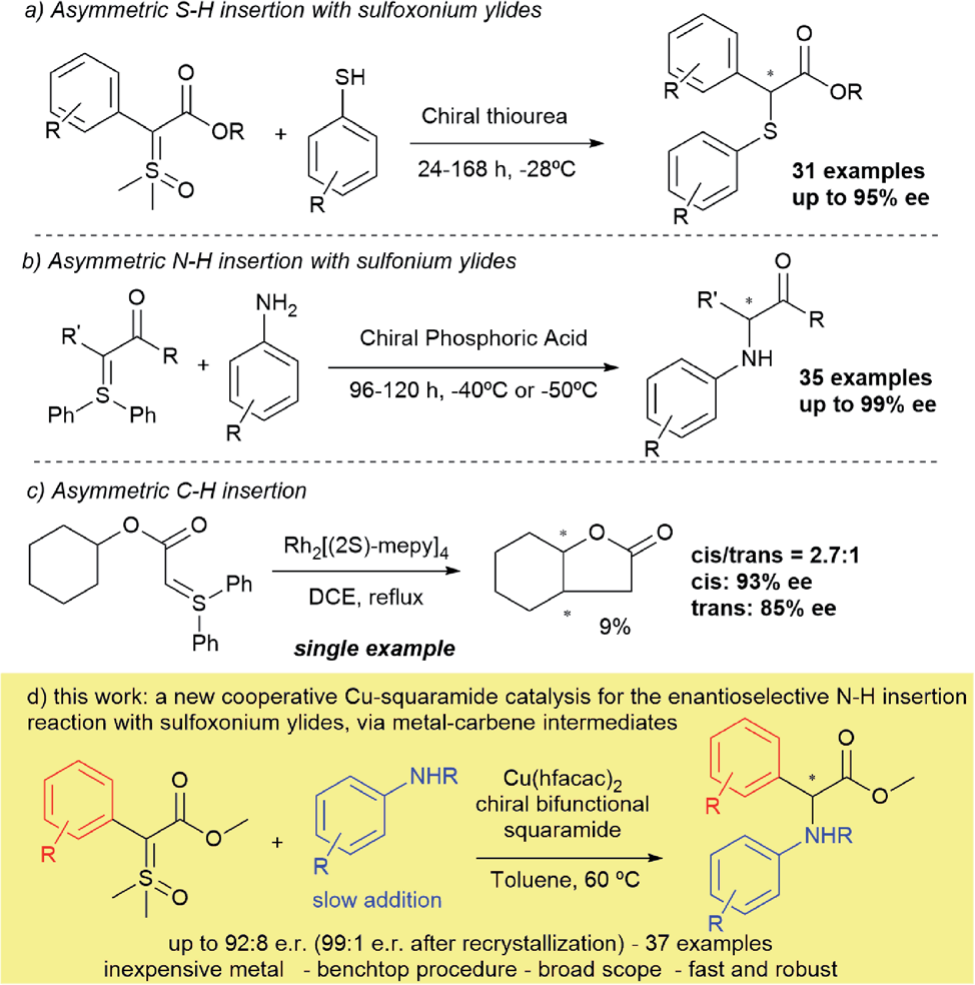
Prior examples and current approach towards asymmetric insertions with sulfur ylides.

Despite these important contributions, the first two enantioselective versions of X–H insertions were reported only last year – the first of which was developed by our research group in collaboration with the Mattson group, based on asymmetric S–H insertions with sulfoxonium ylides and arylthiols, catalysed by a chiral thiourea^37^ (Scheme 1a). The second, describes a chiral phosphoric acid catalysed N–H insertion in the presence of anilines and sulfonium ylides^38^ by Sun (Scheme 1b). Both methodologies relied on the same mechanism: asymmetric protonation of the sulfur ylide, followed by nucleophilic displacement, and were not mediated by a metal-carbene intermediate. In fact, to the best of our knowledge, except for the work by Müller^39^ in 1999 (just a single example shown in 9% yield; Scheme 1c) there has yet to be demonstrated an enantioselective insertion reaction on any type of sulfur ylide that is mediated by carbenes. An approach that would fulfil these criteria would potentially open the field to a broad range of alternative insertion reactions into carbenes.

Herein we describe the first examples of a transformation combining high yields and enantioselectivities up to 92 : 8 (99 : 1 after recrystallization), using a sulfur ylide as a metal carbene precursor (Scheme 1d). Using a catalytic system that combines a chiral squaramide and copper hexafluoroacetylacetonate, thirty-seven α-arylamino arylglycine derivatives have been prepared, many with high enantioselectivities. In addition, the method benefits from a facile bench top procedure and quick reaction times, giving access to these high-value compounds.

## Results and discussion

**Scheme 2:**
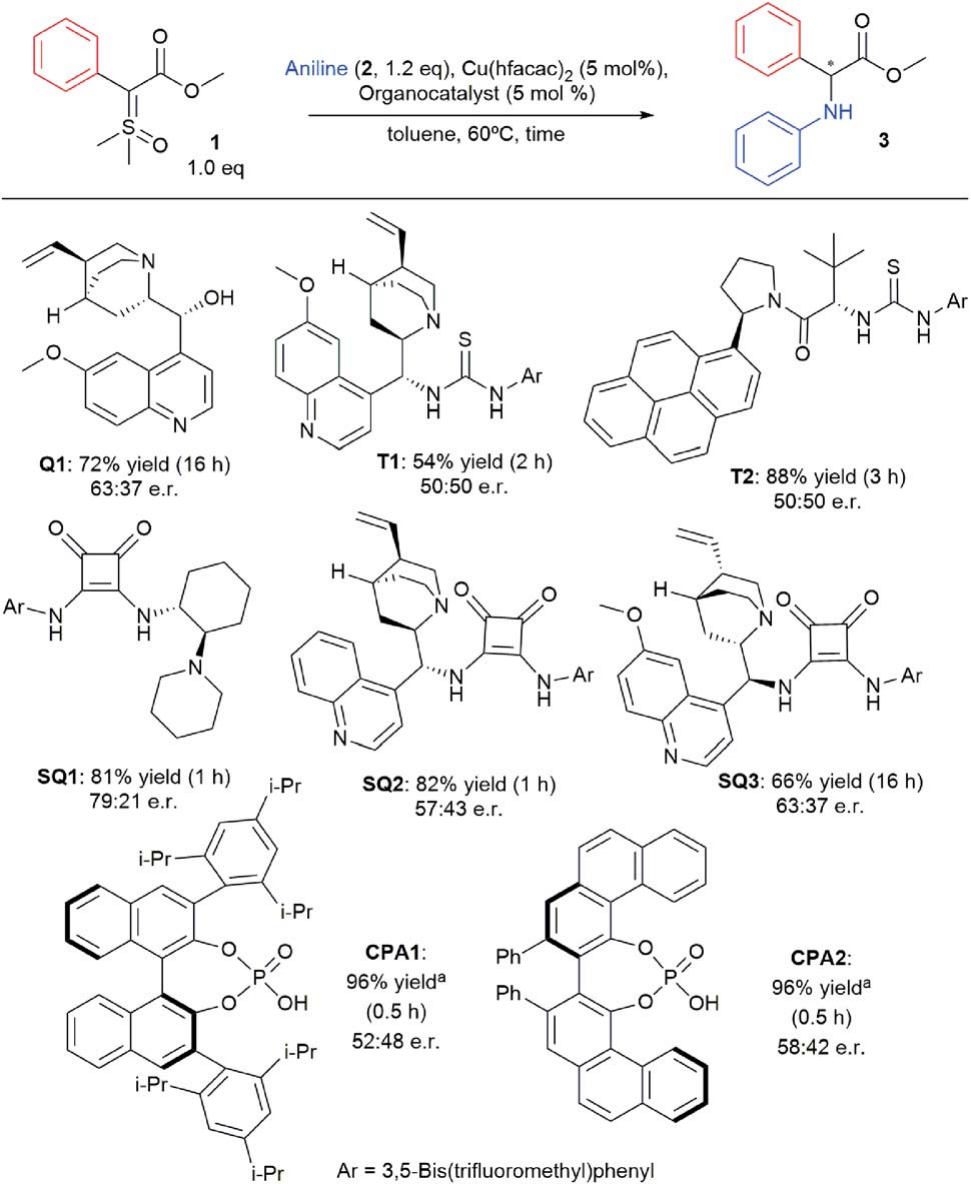
Organocatalyst screening for asymmetric N–H insertion.

Inspired by the early work of Zhou and co-workers that employed chiral phosphoric acids as organocatalysts to promote asymmetric N–H insertion reactions with diazo compounds and rhodium salts, we hypothesized that a bifunctional chiral H-bond donor could also work as a chiral proton shuttle.^17^ Therefore we commenced our studies by screening different organocatalysts in our optimized conditions recently published for the racemic version of the reaction.^36^ The main results are depicted in Scheme 2 (for full optimization tables see ESI†). Using quinine (**Q1**) as organocatalyst, product **3** was formed with 63 : 37 e.r. in 72% yield. However, no chiral induction was observed when thiourea-based organocatalysts were used (**T1** and **T2**). When squaramide-based organocatalyst **SQ1** was used, product **3** was formed with 79 : 21 e.r. in 82% yield. Other squaramide-based organocatalysts (**SQ2** and **SQ3**) failed to improve the enantioselectivity. Lastly, chiral phosphoric acids were also evaluated, yet **CPA1** and **CPA2** also provided inferior enantioselectivity when compared to **SQ1**.

With organocatalyst **SQ1** proving to be the most efficient, we then turned our efforts to screen different conditions, such as combination of solvents, different temperatures, other ylides and addition conditions. The main results are depicted in Table 1 (for complete table, see ESI†). We initiated the study evaluating the reaction at r.t. with DCM as solvent. This condition furnished product **3** in 77% yield and only 70 : 30 e.r. (entry 1). Using a combination of DCM/toluene (1 : 1) as solvent the enantioselectivity was improved to 71 : 29 e.r. at the expense of a longer reaction time (17 h, entry 2). Using toluene as lone solvent at room temperature resulted in almost no reaction taking place (different from DCM, sulfoxonium ylide **1** is visibly insoluble in toluene at r.t.). Increasing the reaction temperature to 40 °C did not result in improvement as product **3** was formed in 8% yield as a racemate (entry 4). This result reaffirms that 60 °C is necessary for best enantioselectivities to be achieved. Interestingly, when Cu(OTf)_2_ was used as the metal catalyst instead of Cu(hfacac)_2_, no enantioselectivity was observed (entry 5). Changing the metal catalyst to [Ir(COD)Cl]_2_, well-known in Mangion's work to promote X–H insertions with sulfoxonium ylides, also didn't result in improvement of the enantioselectivity when compared to Cu(hfacac)_2_ (63 : 37 e.r., entry 6). In fact, several other metal sources and copper complexes were evaluated, but the combination of Cu(hfacac)_2_ and **SQ1** furnished by far the best enantioselectivity. This indicates the uniqueness of our catalyst system for this cooperative catalysis (see ESI† for full optimization table). In entries 7–9, different esters were evaluated, but all performed worse than methyl ester (from 32–38% ee, against 58% ee). Since it is known that another molecule of the X–H nucleophile can catalyse the 1,2 proton shift, leading to racemic product, we decided to evaluate slow addition of aniline into the reaction mixture.^40^ Gratifyingly, this modification in the procedure resulted in an increase of enantioselectivity (89 : 11 e.r., entry 10). Changing the ratio of ylide : aniline (entries 11–13), resulted in little improvement of the enantioselectivity, with the best ratio being 1.5 : 1.0 (entry 13, 91 : 9 e.r.). Lastly, other solvents were screened, but toluene performed better both with respect to yield and e.r. (entries 14–16). The absolute configuration of insertion product **3** was assigned (*S*) by comparison with the described optical rotation value.^41^ It is also worth noting that when we applied our optimized conditions with the corresponding diazo compound, a 56% chemical yield was obtained, but without any enantioselectivity (performing the reaction at lower temperatures did not change this result).

**Table 1:** Reaction conditions screening for asymmetric N–H insertion

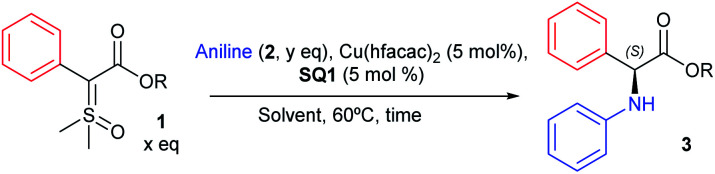						
Entry^a^	1 : 2 eq.	R	Solvent	*t*/min^−1^	Yield^g^	e.r.^h^
1^b^	1.0/1.2	Me	DCM	60	77%	70 : 30
2^b^	1.0/1.2	Me	DCM/toluene	17 h	79%	71 : 29
3^b^	1.0/1.2	Me	Toluene	5 d	Trace	n.d.
4^c^	1.0/1.2	Me	Toluene	21 h	8%	50 : 50
5^d^	1.0/1.2	Me	Toluene	16 h	86%	50 : 50
6^e^	1.0/1.2	Me	Toluene	16 h	74%	63 : 37
7	1.0/1.2	Ph (**4**)	Toluene	120	75%^i^	69 : 31
8	1.0/1.2	Bn (**5**)	Toluene	120	73%^i^	68 : 32
9	1.0/1.2	*t*-Bu (**6**)	Toluene	120	68%^i^	66 : 34
10^f^	1.0/1.2	Me	Toluene	30	64%	89 : 11
11^f^	1.0/1.0	Me	Toluene	30	64%	87 : 13
12^f^	1.25/1.0	Me	Toluene	30	84%	90 : 10
**13** f	**1.5/1.0**	**Me**	**Toluene**	**40**	**83%**	**91 : 9**
14^f^	1.5/1.0	Me	Anisole	40	62%	53 : 47
15^f^	1.5/1.0	Me	1,2-DCE	40	54%	56 : 44
16^f^	1.5/1.0	Me	Cyclohexane	40	66%	90 : 10

a Conditions: 0.1 mmol of **1**, 0.12 mmol of **2**, 0.5 mL of toluene, Cu(hfacac)_2_ and SQ 5 mol%.

b Reaction at room temperature.

c Reaction at 40 °C.

d Cu(OTf)_2_ was used instead of Cu(hfacac)_2_.

e [Ir(COD)Cl]_2_ (2.5 mol%) was used instead of Cu(hfacac)_2_.

f Limiting reagent in 0.1 mmol scale and slow addition of **2** in 0.5 mL of solvent over 15 min.

g Yield calculated by quantitative NMR analysis with hexamethylbenzene as internal standard.

h Determined by HPLC.

i Isolated yield.

**Scheme 3:**
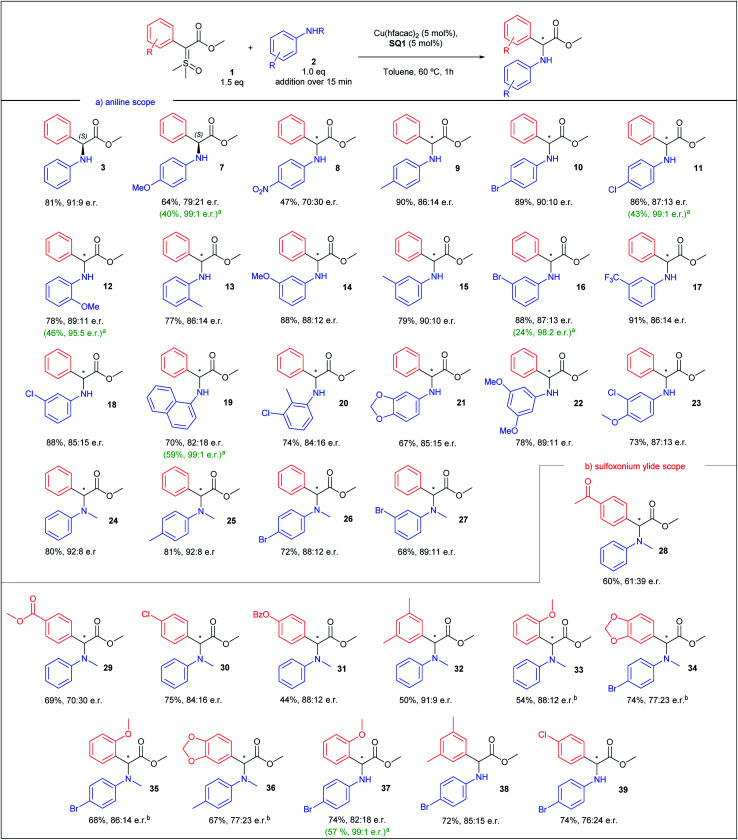
Substrate scope for the asymmetric N–H insertion with sulfoxonium ylides and anilines. All yields correspond to the isolated yield. ^a^ After a single recrystallization. ^b^ Aniline addition over 5 min.

After finding the optimal conditions for the asymmetric N–H insertion we began to evaluate substituent effect on aniline, as depicted in Scheme 3a. Initially, *para* substituted primary anilines were evaluated. Both moderate to strong electron-donating and -withdrawing groups (–OMe and –NO_2_) furnished insertion products with lower enantioselectivities (**7** and **8**, 58 and 40% ee), but after a single recrystallization, product **7** was obtained with 99 : 1 e.r. and 40% yield (absolute configuration assigned (*S*), by optical rotation comparison with literature data).^42^ Next, –Me, –Cl, and –Br substituted anilines produced insertions products with enantioselectivities above 70% ee (72–80% ee, **9–11**). Again, an increase to 99 : 1 e.r. was observed for **11** after recrystallization. *Ortho* substituted anilines were also evaluated. –OMe and –Me substituted anilines provided N–H insertion products with enantiomeric ratios above 86 : 14, in good yields (products **12** and **13**), with **12** providing an enrichment to 95 : 5 e.r. after recrystallization. *Meta* substituted anilines supplied insertion products with e.r. above 85 : 15 for all products, even with –OMe and –CF_3_ substituted anilines (**14**-**18**), in yields above 79%. An enantioenrichment of product **16** was also achieved after recrystallization (98 : 2 e.r.). 1-Naphtylamine provided N–H insertion product with 82 : 18 e.r. and 70% yield (**19**) and 99 : 1 e.r. after recrystallization. Thereafter, di and tri-substituted anilines were screened. Insertion products **20–23** were obtained in yields above 67% and enantiomeric ratios above 84 : 16. Lastly, *N*-methyl anilines were evaluated. Unsubstituted *N*-methylaniline resulted in product **24** with 92 : 8 e.r., in 80% yield. Monosubstituted *N*-methylanilines also provided products **25–27** with enantiomeric ratios up to 92 : 8 and yields above 68%. Observing these results, we can conclude that mild electron-releasing or -withdrawing groups do not cause drastic change in the enantioselectivity, and *meta* position is particularly insensitive to substituent nature. Even *o*-anisidine, where the electron-releasing resonance effect is counterbalanced by stronger electron-withdrawing inductive effect, with substituents closer to the reaction center, provided excellent enantioselectivity. In a comparison between primary and secondary anilines, the latter showed slightly better enantioselectivities.

Having evaluated the aniline scope, we then turned our attention to the sulfoxonium ylide scope. The results are presented in Scheme 3b. We began the ylide scope analysing *para* substituted sulfoxonium ylide in combination with *N*-methylaniline. Ylides with moderate to strong electron-withdrawing groups resulted in insertion products with lower enantioselectivities (**28** and **29**, 22 and 40% ee). *p*-Cl substituted sulfoxonium ylide furnished insertion product **30** with 84 : 16 e.r. and the *p*-OBz gave product **31** with 88 : 12 e.r. Other patterns of ylide substitution were also evaluated. 3,5-Dimethyl substituted ylide furnished insertion product **32** in 91 : 9 e.r., in 50% yield. *o*-OMe substituted ylide resulted in insertion product **33**, with 88 : 12 e.r. Substitution in both ylide and *N*-methylaniline did not provide an increase of the enantioselectivity (products **34–36**). When 4-bromoaniline was used, better enantioselectivity was obtained in combination with ylides with electron-releasing groups (**37** and **38**) than with electron-withdrawing group (**39**).

**Fig. 1:**
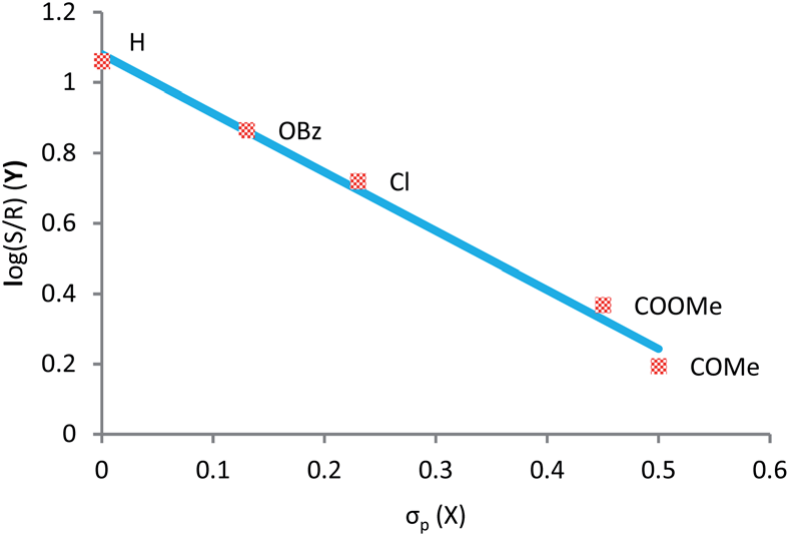
Correlations between logarithms of e.r. and Hammett constants of *para* substituents on ylide. Reaction was carried-out with *N*-methyl aniline.

To gain a better understanding of the substituent tendency correlation on the ylide reactant with the enantiomeric ratio (e.r.), we decided to construct a Hammett plot (Fig. 1).

Observing Fig. 1, the Hammett plot indicates a linear correlation between the nature of the substituent on sulfoxonium ylide and the logarithm of the e.r. Clearly, electron withdrawing substituents results in lower enantiomeric ratios, while electron releasing groups tend to provide insertion products with higher enantiomeric ratios. Unfortunately, the synthesis and isolation of this type of ylide containing moderate to strong electron-donating substituents (such as 4-OMe, 4-OH and 4-NR_2_) is particularly challenging. Our attempts, as well as of other groups, to prepare them have failed so far.^30,43^

**Scheme 4:**
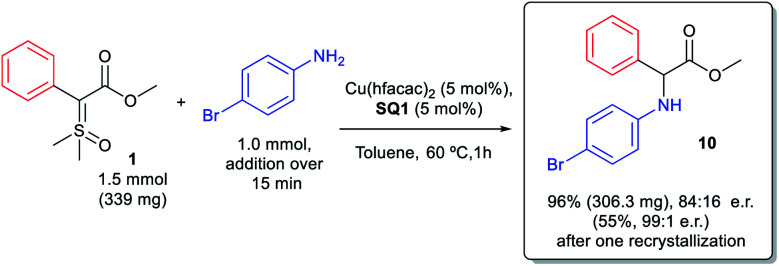
N–H insertion reaction in 1 mmol scale.

We also evaluated the scalability of the reaction. Insertion product **10** was synthesized on a 1 mmol scale, as depicted in Scheme 4. The reaction on 1 mmol scale furnished product **10** in excellent yield (96%) though with a little lower enantioselectivity (84 : 16 e.r. compared to 90 : 10 e.r. on a 0.1 mmol scale). However, after a single recrystallization we were able to enrich the compound to almost its enantiopure form (99 : 1 e.r.) with 55% of recrystallization yield.

**Scheme 5:**
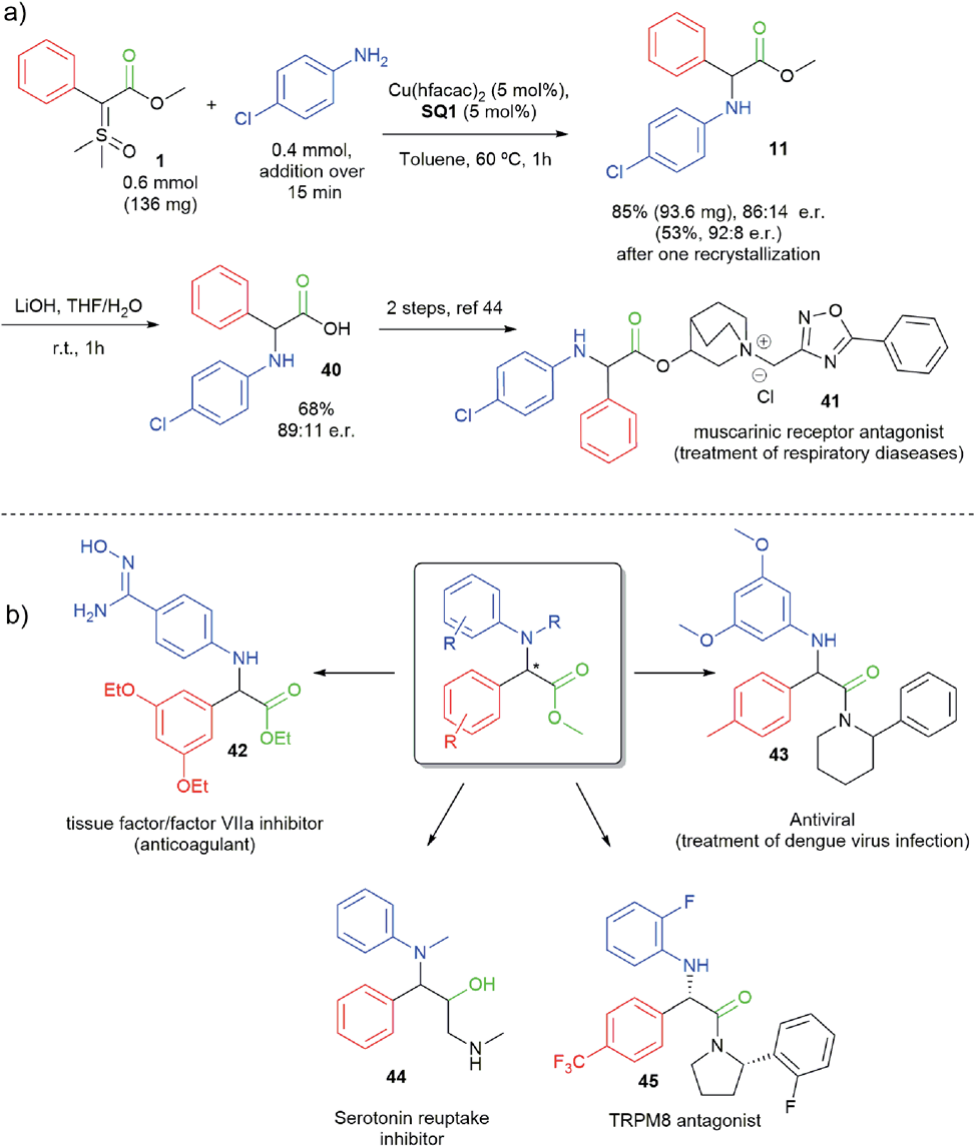
(a) Formal synthesis of muscarinic receptor antagonist **41**. (b) Possible applications of the synthesized α-arylamino phenylglycine derivatives.

To demonstrate the potential of this work, as well as the importance of the prepared α-arylamino phenylglycine derivatives, a short formal synthesis of the muscarinic receptor antagonist **41** was disclosed (Scheme 5a) in just two steps from ylide **1**.^44^ Moreover, Scheme 5b indicates a series of biological important molecules (**42–45**)^45–48^ that could be easily accessed from our enantioselective N–H insertion method using sulfoxonium ylides. It is important to mention that most of these compounds were prepared and evaluated as racemates in the original works.

**Fig. 2:**
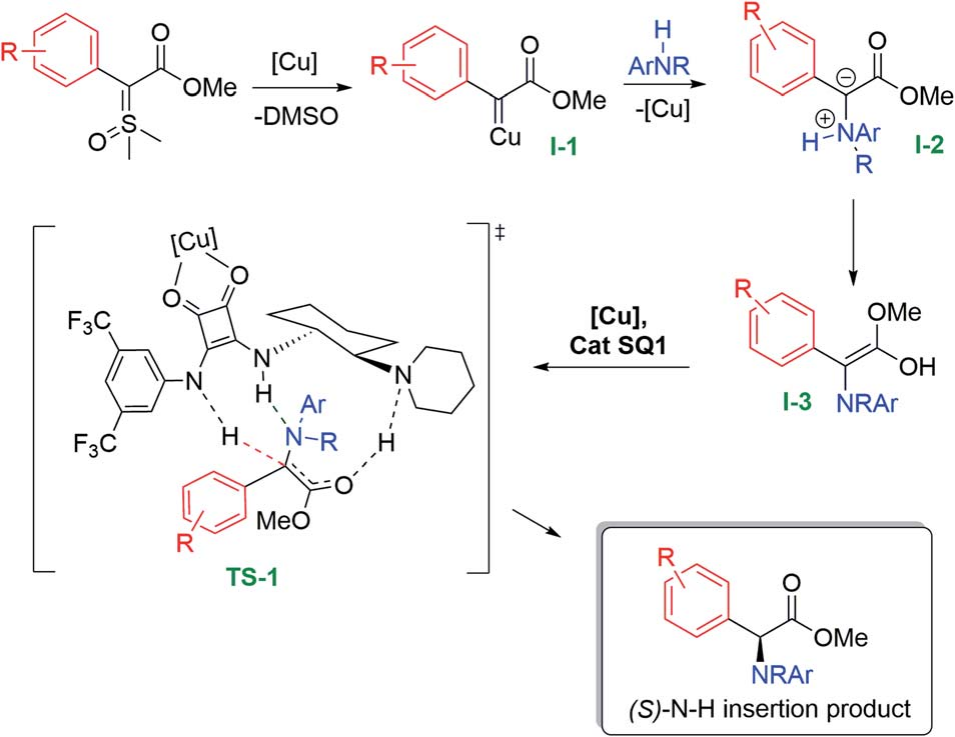
Mechanism proposal for the asymmetric N–H insertion with sulfoxonium ylides.

During the preparation of our product scope, the important contribution described by Zhou was published, where the authors studied in detail the mechanism for the asymmetric N–H insertion reaction with diazo compounds and alkyl amines, mediated by a suchlike cooperative catalysis approach.^18^ As depicted in Fig. 2 and to account for the enantioselectivity, we hypothesize that the reaction mechanism should follow a similar path to the one proposed by Zhou.

Initially, the nucleophilic ylide attacks the copper complex and, after loss of DMSO, forms the copper carbene intermediate (I-1). This electrophilic metal-carbene is then attacked by the aniline, forming the ammonium ylide intermediate (I-2). This intermediate tautomerizes to the free enol (I-3), which readily interacts with a copper-bifunctional squaramide complex generating transition state TS-1. With copper complexed with squaramide carbonyl groups, there is an enhancement of the acidity of the organocatalyst NH proton, which culminates with the asymmetric protonation of the enol intermediate, furnishing the enantioenriched N–H (*S*)-insertion product. However, the fact that our methodology furnished only racemic products when diazo compounds were employed, added to the observation that Zhou's copper scorpionate catalyst gave no insertion product when we applied it to the sulfoxonium ylides, indicates that a comparison of the two mechanisms is not straightforward.

## Conclusions

In summary, the first efficient enantioselective catalytic transformation, with a sulfur ylide acting as a metal-carbene precursor, has been demonstrated. From a straightforward and simple protocol thirty-seven arylglycines were synthesized, where a copper salt and a chiral squaramide cooperatively catalysed the asymmetric N–H insertion reaction in very good to high enantiomeric ratios. Application of this method in the formal synthesis of a muscarinic receptor antagonist was also carried-out. We believe that this methodology will further encourage the development of other enantioselective metal-carbene mediated transformations from sulfur ylides, a field that has been widely unexplored in organic synthesis.

## Author contributions

Conception of the main idea of the manuscript, supervision, and grant support to conduct this work was done by A. C. B. B. Draft preparation was done by L. G. F. and A. C. B. B. Initial screening, intellectual contributions along the work and product scope were performed by L. G. F. Product scope (30%) was done by R. E.

## Conflicts of interest

There are no conflicts to declare.

## Supplementary Material

SC-012-D1SC00979F-s001
