# Supplementary material for: Cooperative copper-squaramide catalysis for the enantioselective N–H insertion reaction with sulfoxonium ylides
Source: Chem Sci. 2021 Apr 26;12(21):7453–9. doi: 10.1039/d1sc00979f (PMC8171336; doi:10.1039/d1sc00979f)
Supplement: SC-012-D1SC00979F-s001 [file SC-012-D1SC00979F-s001.pdf]

Electronic Supplementary Material (ESI) for Chemical Science.
This journal is © The Royal Society of Chemistry 2021

Supporting information

Cooperative Copper-Squaramide Catalysis for the Enantioselective N-H Insertion Reaction with Sulfoxonium Ylides

Lucas G. Furniel, Radell Echemendía, Antonio C. B. Burtoloso*

*São Carlos Institute of Chemistry, University of São Paulo, São Carlos, SP, CEP 13560-970, Brazil. E-mail: antonio@iqsc.usp.br

TABLE OF CONTENTS

1. General information ... 3

2. Optimization of the reaction conditions ... 4

 a. Optimization for asymmetric N-H insertion reaction with heating and without slow addition ... 4

 b. Reaction screening at room temperature or below ... 5

3. Preparation of arylated Sulfoxonium ylides ... 6

4. General procedure for asymmetric N-H insertion with slow addtion ... 10

5. Data for the Hammet Plot ... 28

6. ^{1}H and ^{13}C NMR Spectra ... 29

7. HPLC Cromatograms ... 78

8. References ... 120

1. General information

All solvents were dried and distilled prior to use by standard procedures. Reagents, metal catalysts and organocatalysts **Q1**, **T1**, **CPA1** and **CPA2** were purchased at the highest commercial quality and used without further purification, unless stated otherwise. Reactions were monitored by thin layer chromatography (TLC), carried out using silica gel 60 F_{254} precoated plates, with UV light (254 nm) as visualizing agent, potassium permanganate in aqueous KOH and ethanolic vanilin for staining.

Flash column chromatography was performed using silica gel 60 (200-400 Mesh) or using Biotage Isolera™ Prime (Snap Ultra 10g). Unless stated otherwise, all yields refer to isolated products after purification by flash column chromatography. The solvent mixtures employed in TLC analysis and in flash column chromatography purifications are reported as volume by volume and in percentages.

Proton nuclear magnetic resonance (^{1}H NMR) spectra were recorded using 400 (Agilent Technologies -400/54 Premium Shielded), 500 (Agilent Technologies - 500/54 Premium Shielded) and 600 MHz (Bruker Ultrashield 600) instruments. For ^{1}H NMR spectra, chemical shifts are referenced from TMS (0.00 ppm). Coupling constants (J) are reported in Hz. Carbon nuclear magnetic resonance (^{13}C NMR) spectra were recorded using a NMR spectrometer at 100, 125 or 150 MHz. For ^{13}C NMR spectra, chemical shifts are given from $CDCl_3$ (77.16 ppm).

High resolution mass spectra (HRMS) were recorded using electron spray ionization (ESI) (QqTOF/MS-Microtof-QII models) and matrix-assisted laser desorption/ionization (MALDI) (Bruker - Autoflex maX - MALDI-TOF/TOF)

Optical rotations were recorded on a Jasco Digital P-2000 Polarimeter with a 1 dm cell and a sodium lamp.

Enantiomeric excess was determined by high performance liquid chromatography (HPLC) - LC-20A Prominence Shimadzu and columns packed with a chiral stationary phase (CSP).

α-ester sulfoxonium ylides were prepared according to literature procedure.[1]

Organocatalysts SQ1[2], SQ2[3], SQ3[3], SQ4[4], SQ5[5], SQ6[6] and T2[7], were prepared according to previously reported procedures.

2. Optimization of the reaction conditions

a. Optimization for asymmetric N-H insertion reaction with heating and without slow addition

Table S1. Optimization for reaction with heating and without slow addition.

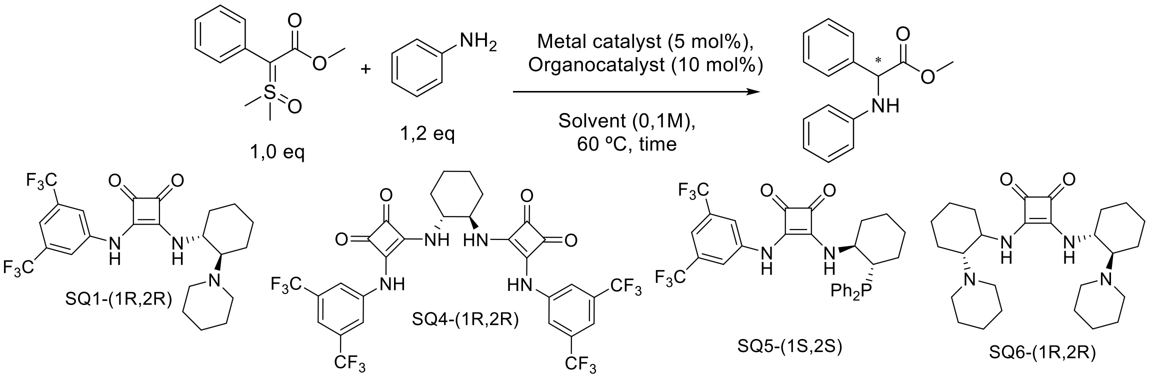

Entry	Metal catalyst	Solvent	Organocatalyst	time	T /°C	Yield	ee
01	Cu(hfacac)$_2$ (5 mol%)	Toluene	SQ1-(1S,2S) (10 mol%)	15 min	60°C	83%	-53%
02	Cu(hfacac)$_2$ (5 mol%)	Tolueno (0,025 M)	SQ1-(1S,2S) (10 mol%)	15 min	60°C	90%	-57%
03[a]	Cu(hfacac)$_2$ (5 mol%)	Toluene	SQ1-(1S,2S) (10 mol%)	5,5 h	40ºC	trace	0%
04	Cu(hfacac)$_2$ (5 mol%)	Toluene	SQ1-(1S,2S) (10 mol%)	15 min	80°C	84%	-59%
05	Cu(hfacac)$_2$ (5 mol%)	Toluene	SQ1-(1S,2S) (10 mol%)	10 min	100°C	84%	-59%
06	Cu(hfacac)$_2$ (2,5 mol%)	Toluene	SQ1-(1S,2S) (5 mol%)	18 h	60°C	47%	-4%
07	Cu(hfacac)$_2$ (5 mol%)	Toluene	SQ1-(1S,2S) (15 mol%)	18 h	60°C	72%	-22%
08	Cu(hfacac)$_2$ (5 mol%)	1,2-DCE	SQ1-(1S,2S) (10 mol%)	15 min	60°C	80%	-48%
09	Cu(hfacac)$_2$ (5 mol%)	$C_6H_5CF_3$	SQ1-(1S,2S) (10 mol%)	15 min	60°C	83%	-48%
10	Cu(hfacac)$_2$ (5 mol%)	TFE	SQ1-(1S,2S) (10 mol%)	15 min	60°C	81%	-2%
11	Cu(hfacac)$_2$ (5 mol%)	Benzene	SQ1-(1S,2S) (10 mol%)	15 min	60°C	88%	-54%
12	Cu(hfacac)$_2$ (5 mol%)	MeCN	SQ1-(1S,2S) (10 mol%)	17 h	60°C	45%	-5%
13	Cu(hfacac)$_2$ (5 mol%)	Toluene	SQ1-(1R,2R) (10 mol%)	15 min	60°C	99%	59%
14	Ni(dppe)Cl$_2$	Toluene	SQ1-(1R,2R) (10 mol%)	16 h	60°C	trace	0%
15	Cu(acac)$_2$ (5 mol%)	Toluene	SQ1-(1R,2R) (10 mol%)	16 h	60°C	trace	0%
16	[Ru(p-cymene)Cl]$_2$ (5 mol%)	Toluene	SQ1-(1R,2R) (10 mol%)	15 min	60°C	67%	20%
17	CuCl (5 mol%)	Toluene	SQ1-(1R,2R) (10 mol%)	16 h	60°C	76%	0%
18	CuCl$_2$ (5 mol%)	Toluene	SQ1-(1R,2R) (10 mol%)	16 h	60°C	24%	0%
19	CoCl$_2$ (5 mol%)	Toluene	SQ1-(1R,2R) (10 mol%)	16 h	60°C	16%	0%
20	FeCl$_3$.6H$_2$O (5 mol%)	Toluene	SQ1-(1R,2R) (10 mol%)	16 h	60°C	18%	0%
21	FeCl$_2$.4H$_2$O (5 mol%)	Toluene	SQ1-(1R,2R) (10 mol%)	16 h	60°C	19%	0%
22	Cu(hfacac)$_2$ (5 mol%)	Toluene	SQ4-(1R,2R) (10 mol%)	3 h	60°C	82%	0%
23	Cu(hfacac)$_2$ (5 mol%)	Toluene	SQ5-(1S,2S) (10 mol%)	4 h	60°C	70%	5%
24	Cu(hfacac)$_2$ (5 mol%)	Toluene	SQ1-(1R,2R) (2,5 mol%)	15 min	60°C	87%	40%
25	Cu(hfacac)$_2$ (5 mol%)	Toluene	SQ1-(1R,2R) (1 mol%)	30 min	60°C	78%	11%

26[b]	CuBr (5 mol%)	Toluene	SQ1-(1R,2R) (10 mol%)	45 min	60°C	23%	0%
27	Cu(hfacac)$_2$ (5 mol%)	Toluene	SQ6-(1R,2R) (5 mol%)	20 min	60°C	92%	0%

[a]TBAI (10 mol%) as additive. [b]$AgSbF_6$ (5 mol%) as additive.

b.Reaction screening at room temperature or below

Table S2. Optimization for reaction at room temperature or below and without slow addition.

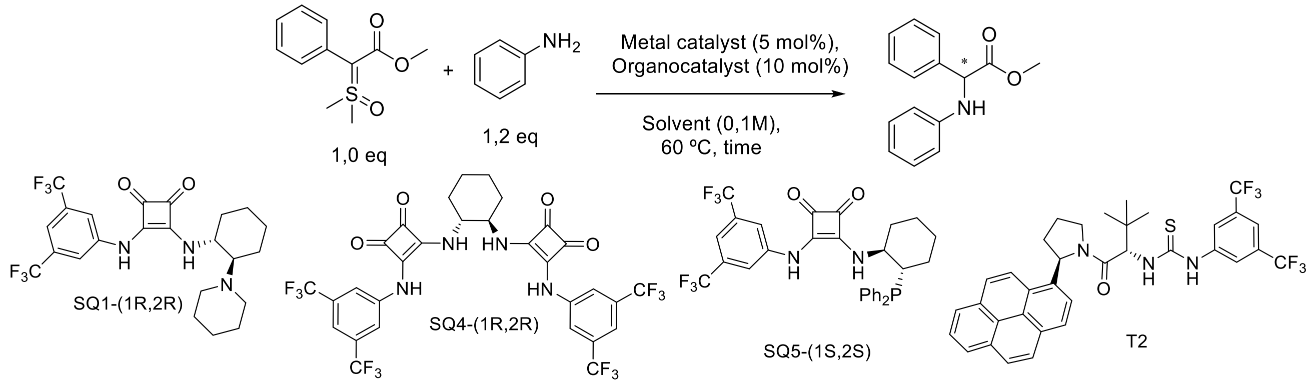

Entry	Ylide	Aniline	Metal catalyst	Solvent	Organocatalyst	time	T /°C	Yield	ee
01	1,0 eq	1,2 eq	Cu(OTf)$_2$ (5 mol%)	DCM	SQ1-(1R,2R) (10 mol%)	44	r.t.	37%	0%
02	1,0 eq	1,2 eq	Cu(hfacac)$_2$ (5 mol%)	DCM	SQ1-(1R,2R) (2,5 mol%)	2,5	r.t.	78%	4%
03	1,0 eq	1,2 eq	Cu(hfacac)$_2$ (5 mol%)	DCM	SQ1-(1R,2R) (1 mol%)	2,5	r.t.	73%	0%
04	1,0 eq	1,2 eq	Cu(hfacac)$_2$ (5 mol%)	1,2-DCE	SQ1-(1R,2R) (10 mol%)	44	r.t.	Trace	0%
05	1,0 eq	1,2 eq	Cu(hfacac)$_2$ (5 mol%)	DCM	SQ1-(1R,2R) (10 mol%)	2,5	r.t.	72%	23%
06	1,0 eq	1,2 eq	Cu(hfacac)$_2$ (5 mol%)	MTBE	SQ1-(1R,2R) (10 mol%)	44	r.t.	Trace	0%
07[a]	1,0 eq	1,2 eq	Cu(hfacac)$_2$ (5 mol%)	DCM	SQ1-(1R,2R) (10 mol%)	2,5	r.t.	84%	0%
08	1,0 eq	1,2 eq	Cu(hfacac)$_2$ (5 mol%)	DCM	SQ1-(1R,2R) (10 mol%)	94	-20ºC	Trace	0%
09	1,0 eq	1,2 eq	Cu(hfacac)$_2$ (5 mol%)	DCM	SQ1-(1R,2R) (10 mol%)	22	-80ºC	Trace	0%
10	1,0 eq	1,2 eq	Cu(hfacac)$_2$ (5 mol%)	DCM	SQ4-(1R,2R) (10 mol%)	22	r.t.	76%	0%
11	1,0 eq	1,2 eq	Cu(hfacac)$_2$ (5 mol%)	DCM	SQ5-(1S,2S) (10 mol%)	22	r.t.	49%	3%
12[b]	1,0 eq	1,2 eq	CuBr (5 mol%)	DCM	SQ1-(1R,2R) (10 mol%)	24	r.t.	77%	0%
13	1,0 eq	1,2 eq	[Ru(p-cymene)Cl]$_2$ (2,5 mol%)	DCM	SQ1-(1R,2R) (10 mol%)	24	r.t.	4%	8%
14	1,0 eq	1,2 eq	[Ir(COD)Cl]$_2$ (1 mol%)	DCM	SQ1-(1R,2R) (10 mol%)	24	r.t.	18%	8%
15	1,0 eq	1,2 eq	[Ir(COD)Cl]$_2$ (2,5 mol%)	DCM	SQ1-(1R,2R) (10 mol%)	6	r.t.	80%	27%
16	1,0 eq	1,2 eq	[Ir(COD)Cl]$_2$ (2,5 mol%)	DCM	SQ1-(1R,2R) (5 mol%)	6	r.t.	67%	40%
17	1,0 eq	1,2 eq	[Ir(COD)Cl]$_2$ (2,5 mol%)	DCM	SQ1-(1R,2R) (2,5 mol%)	18	r.t.	47%	15%
18	1,0 eq	1,2 eq	[Ir(COD)Cl]$_2$ (2,5 mol%)	DCM	SQ1-(1R,2R) (1 mol%)	18	r.t.	11%	6%
19	1,5 eq	1,0 eq	Cu(hfacac)$_2$ (5 mol%)	DCM	T2 (5 mol%)	20	r.t.	74%	0%
20	1,5 eq	1,0 eq	Cu(hfacac)$_2$ (5 mol%)	Toluene	T2 (5 mol%)	120	r.t.	trace	0%

[a]$(PhO)_2POOH$ (10 mol%) as additive. [b]$AgSbF_6$ (5 mol%) as additive.

3. Preparation of arylated Sulfoxonium ylides

General procedure A: A 5 mL round-bottomed flask containing a magnetic stirrer was charged with XPhos (78.6 mg, 0.165 mmol, 0.11 equiv), Pd_2dba_3 (77.6 mg, 0.075 mmol, 0.05 equiv) and Cs_2CO_3 (537.6 mg, 1.65 mmol, 1.1 equiv) under argon. Next, dry MeCN (3.0 mL) was added and the solution was stirred at r.t. for 10 min. In a separate flask were added α-ester sulfoxonium ylide (prepared from dimethylsulfoxonium methylide and dimethyl carbonate)[1] (225.3 mg, 1.0 mmol, 1.0 eq), aryl bromide (2.25 mmol, 1.5 eq) and dry MeCN (3.0 mL) under argon. The ylide solution was then added to the catalyst solution and the reaction was placed in preheated oil bath (75°C) and stirred at this temperature for 15h. The crude mixture was then filtered over a plug of silica at room temperature using a DCM/MeOH (85/15) mixture to transfer all the material and for rinsing. After evaporation of all volatiles, purification by flash chromatography using AcOEt/hexanes as eluent (50% to 100%).

General procedure B: To a 10 mL round-bottomed flask containing a magnetic stirrer and fitted with a Teflon cap, the respective α-ester sulfoxonium ylide (prepared from dimethylsulfoxonium methylide and dimethyl carbonate)[1] (0.6 mmol, 1.0 equiv), activated molecular sieves 4Å powder (115 mg), CsF (273 mg, 1.8 mmol, 3.0 equiv), and dry MeCN (5.0 mL) were added. The reaction mixture was heated to 65°C and the first portion of 2-(trimethylsilyl)phenyl trifluoromethanesulfonate (91.7 mg, 75 µL, 0.3 mmol, 0.5 eq) was added. Two more portions of the aryne precursor were added at 1h and 2h hours of reaction, totalizing 1.5 eq. One hour after the last addition (3 hours of reaction), the organic solvent was removed into rotary evaporator and the crude product purified by flash chromatography, employing the silica gel and mixtures of 0.5 to 2% of MeOH in $CHCl_3$.

Methyl 2-(dimethyl(oxo)-λ6-sulfaneylidene)-2-phenylacetate

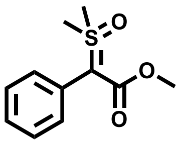

Prepared according general procedure A using 4-Bromobenzene.[1] Yield: 68% (231.1 mg, from 1.5 mmol scale); White solid; R_f = 0,31 (AcOEt); m.p. 184-185°C; ^{1}H RMN (400 MHz, $CDCl_3$) δ 7,38 – 7,22 (m, 5H), 3,61 (s, 3H), 3,40 (s, 6H); ^{13}C RMN (101 MHz, $CDCl_3$) δ 166,8, 133,8, 132,5, 128,6, 127,3, 70,3, 50,6, 43,3; IR (neat): ν (cm^{-1}) = 1624, 1592, 1490, 1436, 1340, 1226, 1168, 1090, 1023, 778, 758, 706. In good agreement with previously reported data.[1] For large scale preparations this ylide can also be prepared, if desired, with a lower cost from the diazo compound route.[1]

Phenyl 2-(dimethyl(oxo)-λ^6-sulfanylidene) 2-phenylacetate

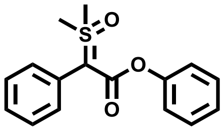

Prepared according to literature procedure B.[8] Yield: 63% (146.6 mg, from 0.8 mmol scale); Yellow solid; R_f = 0,62 (AcOEt); m.p. 139-141°C; ^{1}H RMN (400 MHz, $CDCl_3$) δ 7.44 (m, 2H), 7.39 – 7.26 (m, 5H), 7.15 – 7.10 (m, 1H), 7.09 – 7.04 (m, 2H), 3.43 (s, 6H); ^{13}C RMN (126 MHz, $CDCl_3$) δ 164.6, 151.6, 133.8, 131.8, 129.1, 128.6, 127.4, 124.8, 122.4, 71.0, 43.2; IR (neat): ν (cm^{-1}) = 3016, 2926, 1640, 1593, 1489, 1335, 1173, 1159, 1006, 988, 963, 795, 757, 702. In good agreement with previously reported data.[1]

Benzyl 2-(dimethyl(oxo)-λ^6-sulfanylidene) 2-phenylacetate

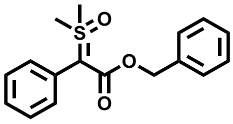

Prepared according general procedure B.[8] Yield: 34% (62.4 mg, from 0.6 mmol scale); Brown oil; R_f = 0,72 (AcOEt); ^{1}H RMN (500 MHz, $CDCl_3$) δ 7.39 – 7.18 (m, 10H), 5.13 (s, 2H), 3.39 (s, 5H); ^{13}C RMN (101 MHz, $CDCl_3$) δ 165.9, 137.9, 133.8, 132.5, 128.5, 128.4, 127.4, 127.3, 127.2, 70.7, 64.5, 43.3; IR (neat): ν (cm^{-1}) = 3028, 2927, 1628, 1593, 1322, 1216, 1171, 1087, 1016, 754, 700. In good agreement with previously reported data.[1]

t-Butyl 2-(dimethyl(oxo)-λ^6-sulfanylidene) 2-phenylacetate

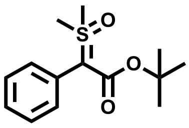

Prepared according to literature procedure B.[8] Yield: 56% (224 mg, from 1.5 mmol scale); Yellow solid; R_f = 0,51 (2% MeOH/AcOEt); m.p. 106-108°C; ^{1}H RMN (400 MHz, $CDCl_3$) δ 7.30 (s, 4H), 7.20 (s, 1H), 3.38 (s, 6H), 1.44 (s, 9H); ^{13}C RMN (126 MHz, $CDCl_3$) δ 166.22, 133.7, 133.5, 128.2, 126.6, 78.9, 71.5, 43.7, 29.0; IR (neat): ν (cm^{-1}) = 2973, 2928, 1619, 1337, 1238, 1151, 1014, 701. In good agreement with previously reported data.[1]

Methyl 2-(4-acetylphenyl)-2-(dimethyl(oxo)-λ6-sulfaneylidene)acetate

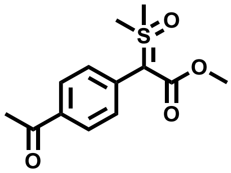

Prepared according general procedure A using 4'-Bromoacetophenone.[1] Yield: 73% (295 mg, from 1.5 mmol scale); White Solid. R_f = 0,45 (AcOEt); m.p. 119-121°C; ^{1}H NMR (400 MHz, $CDCl_3$) δ 7.88 (m, 2H), 7.42 – 7.37 (m,2H), 3.62 (s, 3H), 3.47 (s, 6H), 2.56 (s, 3H); ^{13}C NMR (126 MHz, $CDCl_3$) δ 197.8, 166.1, 138.0, 134.8, 132.5, 129.9, 128.8, 128.4, 128.2, 69.5, 50.6, 43.7, 26.6. IR (neat): ν (cm^{-1}) = 3013, 2930, 1673, 1634, 1592, 1435, 1358, 1333, 1269, 1230, 1171, 1079, 1021, 757. In good agreement with previously reported data.[1]

Methyl 2-(4-chlorophenyl)-2-(dimethyl(oxo)-λ6-sulfaneylidene)acetate

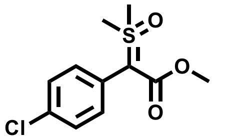

Prepared according general procedure A using 4-Bromochlorobenzene.[1] Yield: 67% (262 mg, from 1.5 mmol scale); White solid; R_f = 0,39 (AcOEt); m.p. 163-165°C; ^{1}H NMR (400 MHz, $CDCl_3$) δ 7.32 – 7.19 (m, 4H), 3.60 (s, 3H), 3.42 (s, 6H). ^{13}C NMR (126 MHz, $CDCl_3$) δ 166.5, 134.9, 133.1, 130.9, 128.7, 68.7, 50.6, 43.4; IR (neat): ν (cm^{-1}) = 3009, 2944, 2927, 1627, 1490, 1435, 1326, 1223, 1167, 1088, 1019, 830, 756, 721. In good agreement with previously reported data.[1]

4-(1-(dimethyl(oxo)- λ6-sulfaneylidene)-2-methoxy-2-oxoethyl)phenyl benzoate

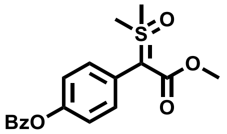

Prepared according general procedure A using methyl 4-bromophenyl benzoate.[1] Yield: 49% (253.5 mg from 1.5 mmol scale); White Solid; m.p. 164-165ºC (decomposes); R_f = 0.32 (AcOEt); ^{1}H NMR (400 MHz, CDCl3) δ 8.18 (m, 2H), 7.64 (m, 1H), 7.51 (m, 2H), 7.35 (m, 2H), 7.20 (m, 2H), 3.62 (s, 3H), 3.43 (s, 6H); ^{13}C NMR (151 MHz, CDCl3) δ 166.7, 165.1, 150.1, 134.8, 133.7, 130.2, 129.6, 128.7, 121.7, 69.5, 50.5, 43.2; IR (neat): ν (cm^{-1}) = 3014, 2931, 1730, 1631, 1504, 1333, 1262, 1227, 1197, 1165, 1081, 1063, 1022, 757, 708; HRMS (MALDI): *m/z* calcd for $C_{18}H_{18}O_5S+H^+$: 347.09477 $[M+H]^+$; found 347.09254.

Methyl 2-(dimethyl(oxo)-λ6-sulfaneylidene)-2-(3,5-dimethylphenyl)acetate

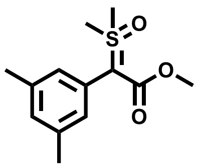

Prepared according general procedure A using 1-bromo-3,5-dimethylbenzene.[1] Yield: 52% (223.6 mg, from 1.5 mmol scale); White solid; R_f = 0,41 (AcOEt); m.p. 109-111°C; ^{1}H RMN (500 MHz, $CDCl_3$) δ 6.91 (m, 3H), 3.60 (s, 3H), 3.38 (s, 6H), 2.29 (s, 6H); ^{13}C NMR (126 MHz, $CDCl_3$) δ 166.9, 138.0, 132.1, 131.7, 129.4, 70.5, 50.6, 43.1, 21.3; IR (neat): ν (cm^{-1}) = 3016, 2927, 2864, 1627, 1595, 1436, 1342, 1270, 1185, 1170, 1072, 1018, 703. In good agreement with previously reported data.[1]

Methyl 4-(1-(dimethyl(oxo)- λ6-sulfaneylidene)-2-methoxy-2-oxoethyl)benzoate

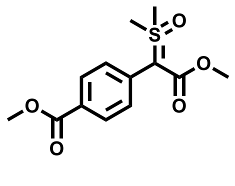

Prepared according general procedure A using methyl 4-bromobenzoate.[1] Yield: 64% (328.4 mg from 1.5 mmol scale); Gray Solid; R_f = 0.39 (AcOEt); m.p. 140ºC (decomposes); ^{1}H NMR (500 MHz, $CDCl_3$) δ 7.96 (d, *J* = 8.4 Hz, 2H), 7.38 (d, *J* = 8.4 Hz, 2H), 3.90 (s, 3H), 3.63 (s, 3H), 3.47 (s, 6H); ^{13}C NMR (126 MHz, $CDCl_3$) δ 167.1, 166.1, 137.8, 132.5, 129.5, 127.8, 69.6, 52.1, 50.6, 43.6; IR (neat): ν (cm^{-1}) = 3014, 2951, 1711, 1634, 1597, 1435, 1326, 1275, 1230, 1177, 1112, 1022, 710; HRMS (MALDI): *m/z* calcd for $C_{13}H_{16}O_5S+Na^+$: 307.06107 $[M+Na]^+$; found 307.0612.

The following sulfoxonium ylides appear to be highly basic and could only be purified employing silica gel previously treated with 5% Et_3N/Hexanes and maintaining a concentration of 5% Et_3N in every eluent mixture during the column. Also the reaction mixture was directly inserted into the column, with no previous filtration in celite or silica. Those ylides appear to have a short lifetime in solution of polar solvents under air and also seem to decompose over time. We could not obtain a molecular ion peak in the HRMS spectra of these compounds probably due to decomposition. It’s best to use shortly after preparation.

Methyl 2-(dimethyl(oxo)-λ6-sulfaneylidene)-2-(2-methoxyphenyl)acetate

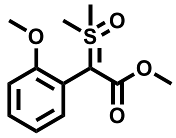

Prepared according general procedure A using 2-bromoanisole (280 mg, 187 µL, 1.5 mmol, 1.5 eq).[1] Yield: 67% (171 mg, from 1.0 mmol scale); White solid; R_f = 0.49 (9:1 AcOEt/Et_3N); m.p. 132°C; ^{1}H NMR (600 MHz, $CDCl_3$): δ 7.32-7.27 (m, 2H); 6.99-6.87 (m, 2H); 3.82 (s, 3H); 3.56 (2xs, 6H); 3.24 (s, 3H); ^{13}C

NMR (151MHz, $CDCl_3$): δ= 167.4, 159.2, 137.2 (2C), 129.6 (2C), 121.1, 111.3, 55.8, 50.5, 43.0, 41.4. IR (neat): ν (cm-1) = 2925, 1734, 1623, 1436, 1352, 1297, 1245, 1186, 1142, 1025, 759.

Methyl 2-(benzo[d][1,3]dioxol-5-yl)-2-(dimethyl(oxo)-λ6-sulfaneylidene)acetate

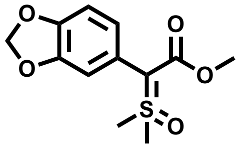

Prepared according general procedure A using 1-bromo-3,4-(methylenedioxy)benzene (301 mg, 180 µL, 1.5 mmol, 1.5 eq). Yield: 43% (113 mg, from 1.0 mmol scale); White solid; R_f = 0.48 (9:1 AcOEt/Et_3N); m.p. 136°C; ^{1}H NMR (600 MHz, $CDCl_3$): δ= 6.78-6.75 (m, 3H); 5.95 (s, 2H); 3.60 (s, 3H); 3.38 (s, 6H). ^{13}C NMR (151 MHz, $CDCl_3$): δ= 167.0, 147.7, 147.3, 127.8 (2C), 114.5, 108.4, 101.3, 50.6, 43.0 (2C).

4. General procedure for asymmetric N-H insertion with slow addtion

To a 4 mL vial were added sulfoxonium ylide **1** (33.9 mg, 0.15 mmol, 1.5 eq), organocatalyst **SQ1** (2.44 mg, 0.005 mmol, 0.05 eq) and $Cu(hfacac)_2.xH_2O$ (2.39 mg, 0.005 mmol, 0.05 eq) at room temperature. The vial was then capped and argon atmosphere was inserted with aid of an argon filled balloon. Next, dry toluene (1.0 mL) was added and this heterogeneous solution was stirred for 15 min at r.t. (sulfoxonium ylides are not soluble at r.t. in toluene). After this time, a solution of aniline (9.1 µL, 0.10 mmol, 1.0 eq) in toluene (1.0 mL) was added over 15 min to the reaction mixture, with aid of a syringe pump (after the first drop of the aniline solution, the reaction mixture was placed in a preheated heating block at 60°C). After the addition of the aniline solution, the reaction mixture was stirred for additional 45 min at 60°C. After this time the reaction was cooled down, the solvent was evaporated and the crude reaction mixture was direct inserted into a Biotage Isolera™ Prime cartridge (Snap Ultra 10 g) and purified using AcOEt and Hexanes eluents (as described for the TLC elution).

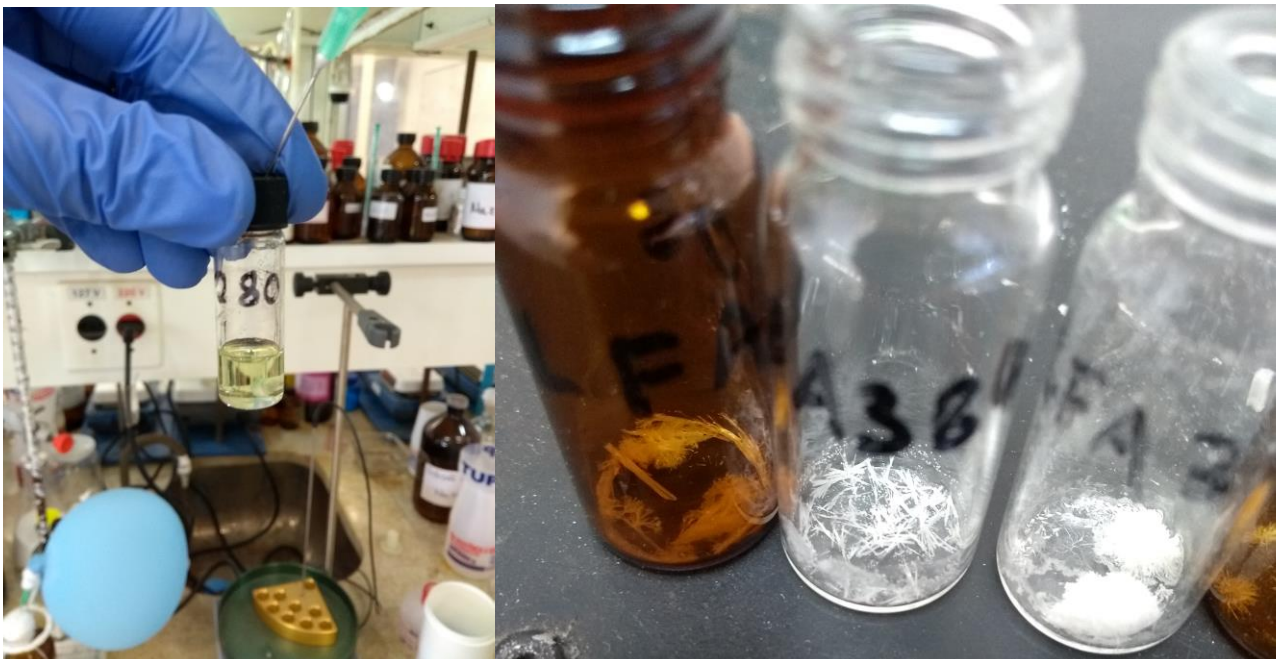

Figure 1. Left: reaction after total consumption of limiting starting material. Right: Recrystallized products from reactions at 0.1 mmol scale.

(S)-Methyl 2-phenyl-2-(phenylamino)acetate (**3**)

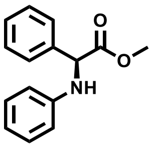

Yield: 81% (19.7 mg); White solid; R_f = 0.47 (10% AcOEt/Hexanes); m.p. 80-82°C; ^{1}H RMN (500 MHz, $CDCl_3$) δ 7,52 – 7,46 (m, 2H), 7,37-7,27 (m, 3H), 7,11 (m, 2H), 6,72 – 6,67 (m, 1H), 6,55 (m, 2H), 5,08 (s, 1H), 4,95 (s, 1H), 3,72 (s, 3H); ^{13}C RMN (101 MHz, $CDCl_3$) δ 172,4, 146,1, 137,7, 129,4, 129,0, 128,4, 127,4, 118,2, 113,5, 60,9, 52,9. In good agreement with previously reported data.[1]

$[\alpha]_D^{20}$= +49.3 (*c* = 0.33 in THF) for 91:9 e.r.; (lit. $[\alpha]_D^{26}$= +68.3 (*c* = 0.32 in THF) for 98% ee (S) sample).[9]

HPLC: t_R = 8.670 min (maj); t_R = 9.722 min (min), *e.r.* = 91:9 (CHIRALPAK ® AD-H, 9:1 hexane/isopropanol, 1.0 mL/min, 220 nm).

Phenyl 2-phenyl-2-(phenylamino)acetate (**4**)

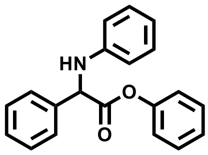

Yield: 75% (22.1 mg); Pale yellow solid; R_f = 0.42 (5% AcOEt/Hexanes); m.p. 90-93°C; ^{1}H RMN (400 MHz, $CDCl_3$) δ 7.64 – 7.58 (m, 2H), 7.45 – 7.28 (m, 5H), 7.26 – 7.11 (m, 3H), 6.98 – 6.91 (m, 2H), 6.74 (m, 1H), 6.64 (m, 2H), 5.34 (d, *J* = 5.6 Hz, 1H), 4.95 (d, *J* = 5.2 Hz, 1H); ^{13}C NMR (126 MHz, $CDCl_3$) δ

170.8, 150.6, 146.0, 137.2, 129.6, 129.5, 129.2, 128.7, 127.5, 126.3, 121.3, 118.6, 113.6, 61.2. In good agreement with previously reported data.[1]

$[\alpha]_D^{22}$= +18.48 (*c* = 1.48 in $CHCl_3$) for e.r.: 69:31.

HPLC: t_R = 16.076 min (maj); t_R = 21.078 min (min), *e.r.* = 69:31 (CHIRALPAK ® AD-H, 95:5 hexane/isopropanol, 1.0 mL/min, 220 nm).

Benzyl 2-phenyl-2-(phenylamino)acetate (**5**)

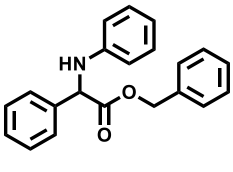

Yield: 73% (23.1 mg); Oily solid; R_f = 0.42 (5% AcOEt/Hexanes). ^{1}H NMR (500 MHz, $CDCl_3$) δ 7.52 – 7.45 (m, 2H), 7.36 – 7.25 (m, 6H), 7.21 – 7.15 (m, 2H), 7.14 – 7.07 (m, 2H), 6.69 (tt, *J* = 7.4, 1.0 Hz, 1H), 6.61 – 6.53 (m, 2H), 5.23 – 5.08 (m, 3H), 4.95 (s, 1H); ^{13}C NMR (126 MHz, $CDCl_3$) δ 171.8, 146.0, 137.6, 135.4, 129.34, 129.0, 128.7, 128.5, 128.4, 128.0, 127.4, 118.3, 113.6, 67.5, 61.0. In good agreement with previously reported data.[1]

$[\alpha]_D^{22}$= +19.34 (*c* = 1.73 in $CHCl_3$) for e.r.: 68:32.

HPLC: t_R = 9.221 min (min); t_R = 11.880 min (maj), *e.r.* = 32:68 (Phenomenex Amylose 2, 93:7 hexane/isopropanol, 1.0 mL/min, 254 nm).

Tert-butyl 2-phenyl-2-(phenylamino)acetate (**6**)

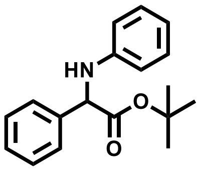

Yield: 68% (19.3 mg); Oily solid; R_f = 0.66 (10% AcOEt/Hexanes); ^{1}H NMR (500 MHz, $CDCl_3$) δ 7.47 (m, 2H), 7.35 – 7.39 (m, 2H), 7.30 – 7.24 (m, 1H), 7.14 – 7.08 (m, 2H), 6.70 – 6.65 (m, 1H), 6.54 (m, 2H), 4.95 (s, 2H), 1.38 (s, 9H); ^{13}C NMR (126 MHz, $CDCl_3$) δ 171.0, 146.3, 138.3, 129.3, 128.8, 128.1, 127.2, 117.9, 113.5, 82.5, 61.4, 28.0. In good agreement with previously reported data.[1]

$[\alpha]_D^{22}$= +15.02 (*c* = 0.43 in $CHCl_3$) for e.r.: 66:34.

HPLC: t_R = 6.005 min (min); t_R = 8.952 min (maj), *e.r.* = 34:66 (Phenomenex Amylose 2, 98:2 hexane/isopropanol, 1.0 mL/min, 220 nm).

(*S*)-Methyl 2-((4-methoxyphenyl)amino)-2-phenylacetate (**7**)

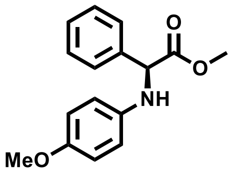

Yield: 64% (17.5 mg); Brown solid; R_f = 0.33 (10% AcOEt/Hexanes); ^{1}H RMN (400 MHz, $CDCl_3$): δ 7.52 – 7.45 (m, 2H), 7.39 – 7.27 (m, 3H), 6.76 – 6.67 (m, 2H), 6.58 – 6.49 (m, 2H), 5.02 (s, 1H), 4.69 (s, 1H), 3.71 (s, 3H), 3.70 (s, 3H); ^{13}C RMN (126 MHz, $CDCl_3$) δ 172.7, 152.6, 140.3, 137.9, 129.0, 128.4, 127.4, 115.0, 114.9, 61.8, 55.8, 52.8. In good agreement with previously reported data.[1]

$[\alpha]_D^{22}$= +50.1 (c = 1.29 in $CHCl_3$) for e.r.: 79:21;

Sample recrystallized from 10% AcOEt/Hex: brown oil (40% yield, enantioenriched in solution); $[\alpha]_D^{21}$ = +70.5 (c = 0.56 in $CHCl_3$) for 99:1 e.r. (lit. $[\alpha]_D^{20}$= -99.2 (c = 0.125 in $CHCl_3$) for 99% ee (R)-sample).[9]

HPLC: t_R = 14.278 min (min); t_R = 20.273 min (maj), *e.r.* = 21:79 (Phenomenex Amylose 2, 9:1 hexane/isopropanol, 1.0 mL/min, 254 nm); t_R = 15.375 min (min); t_R = 22.913 min (maj), *e.r.* = 1:99 (Phenomenex Amylose 2, 9:1 hexane/isopropanol, 1.0 mL/min, 254 nm).

Methyl 2-((4-nitrophenyl)amino)-2-phenylacetate (**8**)

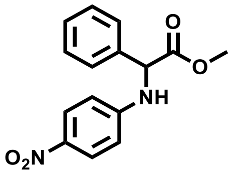

Yield: 47% (13.6 mg); Yellow solid; R_f = 0.36 (15% AcOEt/Hexanes); m.p. 134-136 °C; ^{1}H NMR (400 MHz, $CDCl_3$) δ 8.05 – 7.97 (m, 2H), 7.45 (m, 2H), 7.42 – 7.31 (m, 3H), 6.56 – 6.45 (m, 2H), 5.85 (d, J = 5.3 Hz, 1H), 5.14 (d, J = 5.6 Hz, 1H), 3.76 (s, 3H).^{13}C RMN (126 MHz, $CDCl_3$) δ 171.3, 150.9, 139.0, 136.1, 129.3, 129.0, 127.2, 126.3, 112.2, 60.0, 53.4. In good agreement with previously reported data.[1]

$[\alpha]_D^{22}$ = +42.31 (c = 0.65 in $CHCl_3$) for 70:30 e.r.

HPLC: t_R = 15.295 min (maj); t_R = 16.976 min (min), *e.r.* = 70:30 (CHIRALPAK ® AD-H, 8:2 hexane/isopropanol, 1.0 mL/min, 254 nm).

Methyl 2-phenyl-2-(p-tolylamino)acetate (**9**)

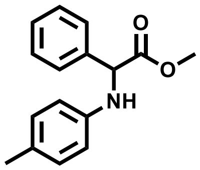

Yield: 90% (23.0 mg); White solid; R_f = 0.46 (5% AcOEt/Hexanes); m.p. 84-85 °C; ^{1}H RMN (400 MHz, $CDCl_3$) δ 7.55 – 7.47 (m, 2H), 7.41 – 7.28 (m, 3H), 6.98 – 6.91 (m, 2H), 6.54 – 6.47 (m, 2H), 5.08 (s, 1H),

4.83 (s, 1H), 3.74 (s, 3H), 2.22 (s, 3H). ^{13}C NMR (101 MHz, $CDCl_3$) δ 172.6, 143.8, 137.8, 129.8, 129.0, 128.4, 127.4, 127.4, 113.6, 61.1, 52.9, 20.5. In good agreement with previously reported data.[1]

$[\alpha]_D^{22}$ = +68.57 (*c* = 1.40 in $CHCl_3$) for 86:14 e.r.

HPLC: t_R = 11.672 min (maj); t_R = 13.207 min (min), *e.r.* = 86:14 (CHIRALPAK ® AD-H, 95:5 hexane/isopropanol, 1.0 mL/min, 254 nm).

Methyl 2-((4-bromophenyl)amino)-2-phenylacetate (**10**)

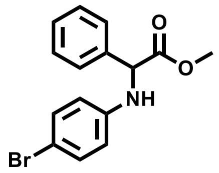

Yield: 89% (28.78 mg); White solid; Rf = 0.41 (5% AcOEt/Hexanes); m.p. 110-112 °C; ^1H NMR (400 MHz, CDCl3) δ 7.51 – 7.42 (m, 2H), 7.41 – 7.28 (m, 3H), 7.25 – 7.15 (m, 2H), 6.48 – 6.38 (m, 2H), 5.03 (s, 2H), 3.74 (s, 3H); ^{13}C NMR (101 MHz, CDCl3) δ 172.1, 144.9, 137.2, 132.1, 129.1, 128.6, 127.3, 115.1, 109.9, 60.7, 53.0. In good agreement with previously reported data.[1]

$[\alpha]_D^{22}$ = +61.14 (c = 2.80 in CHCl3) for 90:10 e.r.

1 mmol scale: Yield: 96% (306.3 mg); 84:16 e.r. Sample recrystallized from hexanes: white crystals (55% yield, Figure 1); $[\alpha]_D^{21}$= +67.2 (c = 0.73 in CHCl3) for 99:1 e.r.

HPLC: t_R = 15.272 min (maj); t_R = 16.621 min (min), e.r. = 90:10 (CHIRALPAK ® AD-H, 95:5 hexane/isopropanol, 1.0 mL/min, 254 nm; t_R = 15.127 min (maj); t_R = 16.564 min (min), e.r. = 99:11 (CHIRALPAK ® AD-H, 95:5 hexane/isopropanol, 1.0 mL/min, 254 nm.

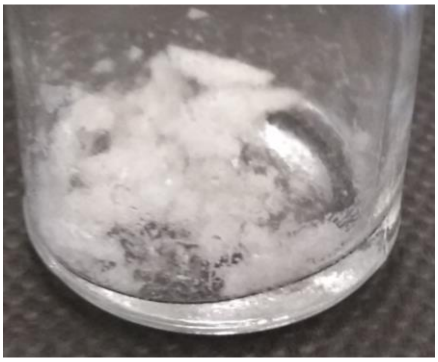

Figure 2. Enantioenriched crystals of **10**.

Methyl 2-((4-chlorophenyl)amino)-2-phenylacetate (**11**)

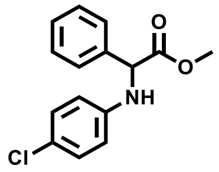

Yield: 86% (23.5 mg); White solid; R_f = 0.41 (5% AcOEt/Hexanes); ^{1}H NMR (400 MHz, $CDCl_3$) δ 7.49 – 7.43 (m, 2H), 7.39 – 7.28 (m, 3H), 7.10 – 7.03 (m, 2H), 6.51 – 6.44 (m, 2H), 5.04 (superimposed 2H), 3.74 (s, 3H); ^{13}C NMR (101 MHz, $CDCl_3$) δ 172.1, 144.5, 137.2, 129.2, 129.1, 128.6, 127.3, 122.8, 114.6, 60.8, 53.0. In good agreement with previously reported data.[1]

$[\alpha]_D^{22}$= +67.75 (*c* = 2.31 in $CHCl_3$) for 87:13 e.r.

Sample recrystallized from hexanes: yellow oil (43% yield, solution enantioenriched); $[\alpha]_D^{21}$= +88.5 (*c* = 0.96 in $CHCl_3$) for 99:1 e.r.

HPLC: t_R = 7.994 min (min); t_R = 9.631 min (maj), *e.r.* = 13:87 (Phenomenex Cellulose 1, 95:5 hexane/isopropanol, 1.0 mL/min, 220 nm); t_R = 8.284 min (min); t_R = 9.979 min (maj), *e.r.* = 1:99 (Phenomenex Cellulose 1, 95:5 hexane/isopropanol, 1.0 mL/min, 220 nm).

Sample prepared from a 0.4 mmol scale of 4-chloroaniline: 85% (93.6 mg), White solid; 86:14 e.r. Sample recrystallized from hexanes: yellow oil (53% yield, solution enantioenriched); 92:8 e.r.

HPLC: t_R = 7.627 min (min); t_R = 9.051 min (maj), *e.r.* = 14:86 (Phenomenex Cellulose 1, 95:5 hexane/isopropanol, 1.0 mL/min, 220 nm); t_R = 8.315 min (min); t_R = 9.113 min (maj), *e.r.* = 8:92 (Phenomenex Cellulose 1, 95:5 hexane/isopropanol, 1.0 mL/min, 220 nm).

Methyl 2-((2-methoxyphenyl)amino)-2-phenylacetate (**12**)

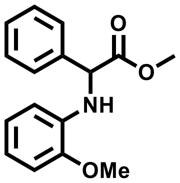

Yield: 78% (21.2 mg); White solid; R_f = 0.56 (5% AcOEt/Hexanes); ^{1}H NMR (400 MHz, $CDCl_3$) δ 7.57 – 7.43 (m, 2H), 7.43 – 7.25 (m, 3H), 6.84 – 6.55 (m, 3H), 6.34 (dd, *J* = 7.7, 1.6 Hz, 1H), 5.47 (d, *J* = 5.1 Hz, 1H), 5.08 (d, *J* = 5.6 Hz, 1H), 3.87 (s, 3H), 3.72 (s, 3H); ^{13}C NMR (101 MHz, $CDCl_3$) δ 172.4, 147.1, 137.8, 136.0, 129.0, 128.4, 127.4, 121.1, 117.5, 110.7, 109.7, 60.8, 55.6, 52.8. In good agreement with previously reported data.[1]

$[\alpha]_D^{22}$= +52.95 (*c* = 2.10 in $CHCl_3$) for 89:11 e.r.

Sample recrystallized from 10% AcOEt/Hex (an cooled at -28°C): oily solid at r.t. (46% yield, enantioenriched crystals); $[\alpha]_D^{21}$ = +67.5 (*c* = 0.96 in THF) for 95:5 e.r.

HPLC: t_R = 7.333 min (min); t_R = 8.029 min (maj), *e.r.* = 11:89 (CHIRALPAK ® AD-H, 95:5 hexane/isopropanol, 1.0 mL/min, 254 nm); t_R = 7.539 min (min); t_R = 8.287 min (maj), *e.r.* = 5:95 (CHIRALPAK ® AD-H, 95:5 hexane/isopropanol, 1.0 mL/min, 254 nm).

Methyl 2-phenyl-2-(o-tolylamino)acetate (**13**)

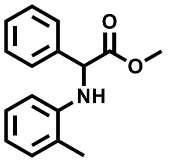

Yield: 77% (19.6 mg); White solid; R_f = 0.51 (5% AcOEt/Hexanes); ^{1}H RMN (400 MHz, $CDCl_3$) δ 7.48 – 7.39 (m, 2H), 7.32 – 7.19 (m, 3H), 6.99 (d, *J* = 7.1 Hz, 1H), 6.95 – 6.88 (m, 1H), 6.57 (t, *J* = 7.4 Hz, 1H), 6.26 (d, *J* = 7.9 Hz, 1H), 5.04 (s, 1H), 4.82 (s, 1H), 3.66 (s, 3H), 2.20 (s, 3H); ^{13}C RMN (126 MHz, $CDCl_3$) δ 172.6, 144.1, 137.8, 130.4, 129.0, 128.4, 127.3, 127.1, 122.6, 117.9, 110.8, 60.9, 53.0, 17.6. In good agreement with previously reported data.[1]

$[\alpha]_D^{22}$= +49.25 (*c* = 0.40 in $CHCl_3$) for 85:15 e.r.

HPLC: t_R = 6.051 min (min); t_R = 6.867 min (maj), *e.r.* = 15:85 (CHIRALPAK ® AD-H, 98:2 hexane/isopropanol, 1.0 mL/min, 220 nm).

Methyl 2-((3-methoxyphenyl)amino)-2-phenylacetate (**14**)

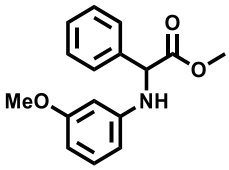

Yield: 88% (23.8 mg); Yellow oil; R_f = 0.32 (5% AcOEt/Hexanes); ^{1}H NMR (500 MHz, $CDCl_3$) δ 7.52 – 7.47 (m, 2H), 7.41 – 7.28 (m, 3H), 7.04 (t, *J* = 8.1 Hz, 1H), 6.27 (dd, *J* = 8.1, 2.1 Hz, 1H), 6.19 (dd, *J* = 8.0, 1.7 Hz, 1H), 6.11 (t, *J* = 2.1 Hz, 1H), 5.09 (s, 1H), 4.92 (br s, 1H), 3.74 (s, 3H), 3.71 (s, 3H); ^{13}C NMR (101 MHz, $CDCl_3$) δ 172.5, 160.7, 147.3, 137.8, 130.1, 129.0, 128.4, 127.3, 106.6, 103.5, 99.7, 60.8, 55.1, 53.0. In good agreement with previously reported data.[1]

$[\alpha]_D^{22}$= +72.35 (c = 2.30, $CHCl_3$) for 88:12 e.r.

HPLC: t_R = 15.942 min (min); t_R = 22.143 min (maj), *e.r.* = 12:88 (Phenomenex Cellulose 1, 95:5 hexane/isopropanol, 1.0 mL/min, 254 nm).

Methyl 2-phenyl-2-(m-tolylamino)acetate (**15**)

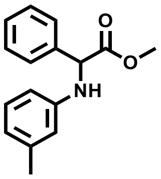

Yield: 79% (20.1 mg); White solid; R_f = 0.44 (5% AcOEt/Hexanes); m.p. 107-108 °C; ^{1}H NMR (500 MHz, $CDCl_3$) δ 7.52 – 7.45 (m, 2H), 7.38 – 7.26 (m, 3H), 7.00 (t, *J* = 7.8 Hz, 1H), 6.52 (d, *J* = 7.5 Hz, 1H), 6.41

(m, 1H), 6.34 (m, 1H), 5.07 (s, 1H), 4.87 (s, 1H), 3.71 (s, 3H), 2.22 (s, 3H); ^{13}C NMR (126 MHz, $CDCl_3$) δ 172.5, 146.1, 139.1, 137.8, 129.2, 129.0, 128.4, 127.4, 119.2, 114.5, 110.5, 60.9, 52.9, 21.7. In good agreement with previously reported data.[1]

$[\alpha]_D^{22}$= +77.45 (*c* = 1.40 in $CHCl_3$) for 90:10 e.r.

HPLC: t_R = 9.895 min (min); t_R = 13.613 min (maj), *e.r.* = 10:90 (Phenomenex Cellulose 1, 95:5 hexane/isopropanol, 1.0 mL/min, 254 nm).

Methyl 2-((3-bromophenyl)amino)-2-phenylacetate (**16**)

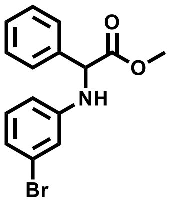

Yield: 88% (28.3 mg); White solid; R_f = 0.41 (5% AcOEt/Hexanes); ^1H NMR (500 MHz, $CDCl_3$) δ 7.47 – 7.43 (m, 2H), 7.39 – 7.28 (m, 3H), 6.94 (t, *J* = 8.0 Hz, 1H), 6.80 (m, 1H), 6.70 (t, *J* = 2.0 Hz, 1H), 6.46 – 6.41 (m, 1H), 5.05 (m, 2H), 3.73 (s, 3H); ^{13}C NMR (126 MHz, $CDCl_3$) δ 172.0, 147.2, 137.1, 130.6, 129.1, 128.6, 127.3, 123.3, 121.0, 116.3, 112.1, 60.5, 53.1. In good agreement with previously reported data.[1]

$[\alpha]_D^{22}$= +60.62 (*c* = 2.72 in $CHCl_3$) for 87:13 e.r.

Sample recrystallized from 10% AcOEt/Hex: yellow oil (24% yield, enantioenriched solution); $[\alpha]_D^{21}$= +229.4 (*c* = 0.647 in $CHCl_3$) for 98:2 e.r.

HPLC: t_R = 11.773 min (maj); t_R = 16.317 min (min), *e.r.* = 87:13 (CHIRALPAK ® AD-H, 95:5 hexane/isopropanol, 1.0 mL/min, 254 nm); t_R = 11.921 min (maj); t_R = 17.403 min (min), *e.r.* = 98:2 (CHIRALPAK ® AD-H, 95:5 hexane/isopropanol, 1.0 mL/min, 254 nm).

Methyl 2-phenyl-2-((3-(trifluoromethyl)phenyl)amino)acetate (**17**)

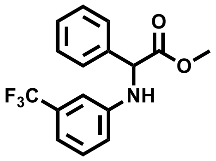

Yield: 91% (28.2 mg); White solid; R_f = 0.33 (5% AcOEt/Hexanes); m.p. 93-94 °C; ^1H NMR (500 MHz, $CDCl_3$) δ 7.53 – 7.46 (m, 2H), 7.44 – 7.30 (m, 3H), 7.20 (t, *J* = 7.9 Hz, 1H), 6.97 – 6.91 (m, 1H), 6.80 (s, 1H), 6.67 (dd, *J* = 8.1, 2.3 Hz, 1H), 5.22 (s, 1H), 5.10 (s, 1H), 3.75 (s, 3H); ^{13}C NMR (101 MHz, $CDCl_3$) δ 172.0, 146.1, 137.0, 131.6 (q, *J* = 31.9 Hz), 129.81, 129.2, 128.7, 127.3, 124.3 (q, *J* = 272.3 Hz), 116.2, 114.62 (q, *J* = 3.9 Hz), 110.0 (q, *J* = 4.0 Hz), 60.5, 53.1. In good agreement with previously reported data.[1]

$[\alpha]_D^{22}$= +50.39 (c = 2.80 in $CHCl_3$) 86:14 for e.r.

HPLC: t_R = 8.031 min (maj); t_R = 9.977 min (min), *e.r.* = 86:14 (CHIRALPAK ® AD-H, 95:5 hexane/isopropanol, 1.0 mL/min, 220 nm).

Methyl 2-((3-chlorophenyl)amino)-2-phenylacetate (**18**)

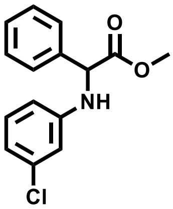

Yield: 88% (24.4 mg); White solid; R_f = 0.44 (5% AcOEt/Hexanes); m.p. 80-82 °C; ^{1}H NMR (400 MHz, $CDCl_3$) δ 7.50 – 7.42 (m, 2H), 7.40 – 7.28 (m, 3H), 7.00 (t, J = 8.1 Hz, 1H), 6.65 (ddd, J = 7.9, 1.9, 0.8 Hz, 1H), 6.53 (t, J = 2.1 Hz, 1H), 6.41 (ddd, J = 8.2, 2.3, 0.7 Hz, 1H), 5.06 (m, 2H), 3.72 (s, 3H); ^{13}C NMR (101 MHz, $CDCl_3$) δ 172.0, 147.1, 137.1, 135.0, 130.3, 129.1, 128.6, 127.3, 118.1, 113.4, 111.8, 60.6, 53.1. In good agreement with previously reported data.[1]

$[\alpha]_D^{22}$= +66.31 (c = 2.44 in $CHCl_3$) for 85:15 e.r.

HPLC: t_R = 11.409 min (maj); t_R = 15.142 min (min), *e.r.* = 85:15 (CHIRALPAK ® AD-H, 95:5 hexane/isopropanol, 1.0 mL/min, 254 nm).

Methyl 2-(naphthalen-1-ylamino)-2-phenylacetate (**19**)

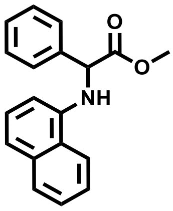

Yield: 70% (20.6 mg); Brown solid; R_f = 0.42 (5% AcOEt/Hexanes); ^{1}H NMR (400 MHz, $CDCl_3$) δ 8.04 – 7.98 (m, 1H), 7.82 – 7.73 (m, 1H), 7.59 – 7.53 (m, 2H), 7.52 – 7.43 (m, 2H), 7.39 – 7.27 (m, 3H), 7.19 (m, 2H), 6.33 (dd, J = 7.1, 1.4 Hz, 1H), 5.78 (s, 1H), 5.24 (s, 1H), 3.76 (s, 3H); ^{13}C NMR (101 MHz, $CDCl_3$) δ 172.5, 141.1, 137.5, 134.4, 129.0, 128.8, 128.5, 127.4, 126.4, 126.0, 125.1, 123.6, 120.2, 118.2, 105.7, 60.9, 53.1. In good agreement with previously reported data.[1]

$[\alpha]_D^{22}$= -34.26 (c = 2.05, $CHCl_3$) for 82:18 e.r.

Sample recrystallized from 25% AcOEt/Hex: brown needles (59% yield, enantioenriched solution); m.p. 131-133 °C; $[\alpha]_D^{21}$= -51.9 (c = 1.15 in $CHCl_3$) for 99:1 e.r.

HPLC: t_R = 17.055 min (min); t_R = 25.549 min (maj), *e.r.* = 18:82 (Phenomenex Cellulose 1, 95:5 hexane/isopropanol, 1.0 mL/min, 254 nm); t_R = 16.607 min (min); t_R = 25.437 min (maj), *e.r.* = 1:99 (Phenomenex Cellulose 1, 95:5 hexane/isopropanol, 1.0 mL/min, 254 nm).

Methyl 2-((3-chloro-2-methylphenyl)amino)-2-phenylacetate (**20**)

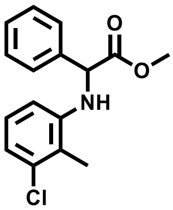

Yield: 74% (21.4 mg); Yellowish solid; R_f = 0.42 (10% AcOEt/Hexanes); m.p. 95-97°C; ^{1}H NMR (400 MHz, $CDCl_3$) δ 7.51 – 7.43 (m, 2H), 7.40 – 7.27 (m, 3H), 6.85 (t, *J* = 8.1 Hz, 1H), 6.74 (d, *J* = 7.5 Hz, 1H), 6.21 (d, *J* = 8.0 Hz, 1H), 5.06 (m, 2H), 3.74 (s, 3H), 2.35 (s, 3H); ^{13}C NMR (101 MHz, $CDCl_3$) δ 172.31, 145.2, 137.3, 134.8, 129.1, 128.6, 127.3, 127.2, 120.3, 118.8, 109.2, 60.9, 53.2, 13.8. In good agreement with previously reported data.[1]

$[\alpha]_D^{22}$= +13.49 (*c* = 1.75 in $CHCl_3$) for 84:16 e.r.

HPLC: t_R = 4.627 min (min); t_R = 5.119 min (maj), *e.r.* = 16:84 (Phenomenex Amylose 2, 9:1 hexane/isopropanol, 1.0 mL/min, 254 nm).

Methyl 2-(benzo[d][1,3]dioxol-5-ylamino)-2-phenylacetate (**21**)

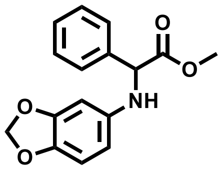

Yield: 67% (19.1 mg); Brown solid; R_f = 0.27 (5% AcOEt/Hexanes); m.p. 88-90 °C; ^{1}H NMR (400 MHz, $CDCl_3$) δ 7.50 – 7.42 (m, 2H), 7.39 – 7.27 (m, 3H), 6.58 (d, *J* = 8.3 Hz, 1H), 6.20 (d, *J* = 2.3 Hz, 1H), 5.98 (dd, *J* = 8.3, 2.4 Hz, 1H), 5.81 (superimposed, 2H), 4.99 (s, 1H), 4.72 (s, 1H), 3.71 (s, 3H); ^{13}C NMR (126 MHz, $CDCl_3$) δ 172.5, 148.4, 141.8, 140.2, 137.7, 129.0, 128.5, 127.4, 108.7, 105.3, 100.7, 96.7, 61.7, 52.9. In good agreement with previously reported data.[1]

$[\alpha]_D^{22}$= +61.31 (*c* = 1.91 in $CHCl_3$) for 85:15 e.r.

HPLC: t_R = 9.594 min (min); t_R = 11.925 min (maj), *e.r.* = 15:85 (Phenomenex Cellulose 1, 9:1 hexane/isopropanol, 1.0 mL/min, 254 nm).

Methyl 2-((3,5-dimethoxyphenyl)amino)-2-phenylacetate (**22**)

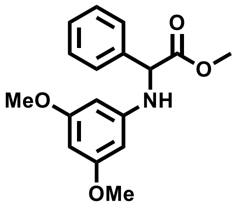

Yield: 78% (23.9 mg); Brown solid; R_f = 0.21 (5% AcOEt/Hexanes); m.p. 85-86 °C; ^{1}H NMR (400 MHz, $CDCl_3$) δ 7.47 (m, 2H), 7.40 – 7.26 (m, 3H), 5.86 (t, *J* = 2.1 Hz, 1H), 5.74 (d, *J* = 2.1 Hz, 2H), 5.05 (d, *J* = 4.5 Hz, 1H), 4.97 (s, 1H), 3.72 (s, 3H), 3.68 (s, 6H); ^{13}C NMR (126 MHz, $CDCl_3$) δ 172.4, 161.7, 147.9, 137.7, 129.0, 128.4, 127.3, 92.5, 90.6, 60.8, 55.2, 53.0. In good agreement with previously reported data.[1]

$[\alpha]_D^{22}$= +63.62 (*c* = 2.35 in $CHCl_3$) for 89:11 e.r.

HPLC: t_R = 12.363 min (min); t_R = 22.858 min (maj), *e.r.* = 11:89 (Phenomenex Cellulose 1, 9:1 hexane/isopropanol, 1.0 mL/min, 254 nm).

Methyl 2-((3-chloro-4-methoxyphenyl)amino)-2-phenylacetate (**23**)

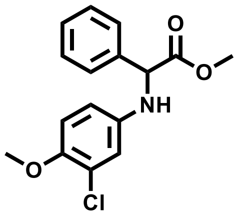

Yield: 73% (22.2 mg); Yellowish solid; R_f = 0.35 (10% AcOEt/Hexanes); ^{1}H NMR (500 MHz, $CDCl_3$) δ 7.51 – 7.42 (m, 2H), 7.40 – 7.27 (m, 3H), 6.73 (d, *J* = 8.8 Hz, 1H), 6.63 (d, *J* = 2.6 Hz, 1H), 6.40 (dd, *J* = 8.8, 2.5 Hz, 1H), 5.01 (s, 1H), 4.65 (br s, 1H), 3.77 (s, 3H), 3.72 (s, 3H); ^{13}C NMR (126 MHz, $CDCl_3$) δ 172.5, 148.0, 140.6, 137.5, 129.1, 128.6, 127.3, 123.4, 116.0, 114.1, 112.7, 61.2, 57.0, 53.0. In good agreement with previously reported data.[1]

$[\alpha]_D^{22}$= +48.91 (*c* = 2.20 in $CHCl_3$) for 87:13 e.r.

HPLC: t_R = 12.709 min (maj); t_R = 16.541 min (min), *e.r.* = 87:13 (CHIRALPAK ® AD-H, 8:2 hexane/isopropanol, 1.0 mL/min, 220 nm).

Methyl 2-(methyl(phenyl)amino)-2-phenylacetate (**24**)

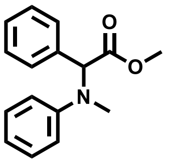

Yield: 80% (20.5 mg); Colorless oil; R_f = 0.45 (5% AcOEt/Hexanes); ^{1}H NMR (400 MHz, $CDCl_3$) δ 7.43 – 7.20 (m, 7H), 6.87 (m, 2H), 6.80 (t, *J* = 7.3 Hz, 1H), 5.67 (s, 1H), 3.77 (s, 3H), 2.79 (s, 3H); ^{13}C NMR (126

MHz, $CDCl_3$) δ 172.5, 145.0, 135.9, 129.4, 128.8, 128.5, 128.2, 118.2, 113.6, 65.8, 52.1, 34.6. In good agreement with previously reported data.[1]

$[\alpha]_D^{22}$= +74.40 (*c* = 2.05 in $CHCl_3$) for 92:8 e.r.

HPLC: t_R = 5.868 min (min); t_R = 6.496 min (maj), *e.r.* = 8:92 (CHIRALPAK ® AD-H, 95:5 hexane/isopropanol, 1.0 mL/min, 254 nm).

Methyl 2-(methyl(p-tolyl)amino)-2-phenylacetate (**25**)

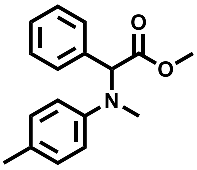

Yield: 81% (21.8 mg); Yellow oil; R_f = 0.51 (5% AcOEt/Hex); ^{1}H NMR (600 MHz, $CDCl_3$): δ 7.38-7.30 (m, 3H); 7.29-7.26 (m, 2H); 7.08 (d, *J*= 8.4 Hz, 2H); 6,80 (d, *J*= 8.6 Hz, 2H); 5.61 (s, 1H); 3.76 (s, 3H); 2.76 (s, 3H); 2.26 (s, 3H); ^{13}C NMR (151MHz, $CDCl_3$): δ= 172.6, 148.0, 136.1, 129.9, 128.7, 128.6, 128.2, 127.7, 114.1, 66.4, 52.1, 34.9, 20.4; HRMS (ESI+qTOF) m/z: calcd for $C_{17}H_{20}NO_2$ $[M+H]^+$ 270.14940; found 270.1497.

$[\alpha]_D^{22}$ = +57.8 (*c* = 0.02 in $CHCl_3$) for 92:8 e.r.

HPLC: t_R = 5.14 min (minor), t_R = 6.22 min (major), e.r. = 8:92; (CHIRALPAK ® AD-H, 9:1 hexane/isopropanol, 1.0 mL/min, 254 nm).

Methyl 2-((4-bromophenyl)(methyl)amino)-2-phenylacetate (**26**)

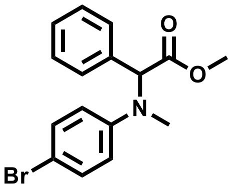

Yield: 72% (24.0 mg); Pale yellow oil; R_f = 0.48 (5% AcOEt/Hex); ^{1}H NMR (600 MHz, $CDCl_3$): δ 7.38-7.29 (m, 5H); 7.23-7.21 (m, 2H); 6.75-6.68 (m, 2H); 5.58 (s, 1H); 3.77(s, 3H); 2.74 (s, 3H); ^{13}C NMR (151MHz, $CDCl_3$): δ 172.1, 149.0, 135.5, 132.1, 128.9, 128.5, 128.4, 115.3, 66.0, 52.3, 34.7; HRMS (ESI+qTOF) m/z: calcd for $C_{16}H_{17}BrNO_2$ $[M+H]^+$ 334.04427; found 334.0445.

$[\alpha]_D^{22}$ = +52.7 (*c* = 0.01 in $CHCl_3$) for 88:12 e.r.

HPLC: t_R = 6.09 min (minor), t_R = 8.09 min (major), e.r. = 12:88; (CHIRALPAK ® AD-H, 9:1 hexane/isopropanol, 1.0 mL/min, 220 nm, 22 °C).

Methyl 2-((3-bromophenyl)(methyl)amino)-2-phenylacetate (**27**)

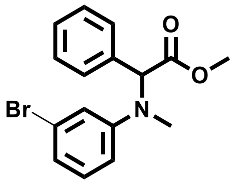

Yield: 68% (22.7 mg); Yellow oil; R_f = 0.49 (5% AcOEt: Hex); ^{1}H NMR (600 MHz, $CDCl_3$): δ 7.41-7.33 (m, 3H); 7.25-7.23 (m, 2H); 7.10 (t, *J*= 8.2 Hz, 1H); 7.02-6.96 (m, 1H); 6.94-6.90 (m, 1H); 6.77 (dd, *J*= 8.4, 2.5 Hz, 1H); 5.71(s, 1H); 3.77(s, 3H); 2.75 (s, 3H); ^{13}C NMR (151MHz, $CDCl_3$): δ= 172.0, 151.2, 135.4, 130.6, 128.9, 128.5, 128.5, 123.7, 120.9, 116.3, 111.8, 65.6, 52.3, 34.6. HRMS (ESI+qTOF) m/z: calcd for $C_{16}H_{17}BrNO_2$ $[M+H]^+$ 334.0443; found 334.0456.

$[\alpha]_D^{22}$ =+43.2 (*c* = 0.01, $CHCl_3$) for 89:11 e.r.

HPLC: t_R = 5.96 min (major), t_R = 8.04 min (minor), e.r. = 89:11; (Phenomenex Lux® cellulose-1, 9:1 hexane/isopropanol, 1.0 mL/min, 220 nm).

Methyl 2-(4-acetylphenyl)-2-(methyl(phenyl)amino)acetate (**28**)

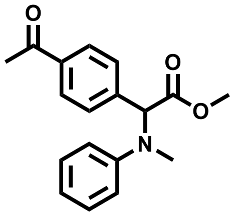

Yield: 60% (17.9 mg); Yellow oil; R_f = 0.45 (20% AcOEt/Hexanes); ^{1}H NMR (600 MHz, $CDCl_3$) δ 7.97 (d, *J* = 8.2 Hz, 2H), 7.39 (d, *J* = 8.0 Hz, 2H), 7.27 (m, 2H), 6.87 (d, *J* = 8.1 Hz, 2H), 6.83 (t, *J* = 7.3 Hz, 1H), 5.68 (s, 1H), 3.80 (s, 3H), 2.81 (s, 3H), 2.61 (s, 3H); ^{13}C NMR (151 MHz, $CDCl_3$) δ 197.7, 171.7, 149.7, 141.3, 136.9, 129.5, 128.8, 128.6, 118.7, 113.8, 65.9, 52.4, 35.0, 26.8. HRMS (ESI): *m/z* calcd for $C_{18}H_{20}NO_3+H^+$:298.14377 $[M+H]^+$; found 298.1451.

$[\alpha]_D^{22}$= +29.56 (*c* = 1.79 in $CHCl_3$) for 61:39 e.r.

HPLC: t_R = 7.799 min (min); t_R = 12.010 min (maj), *e.r.* = 39:61 (CHIRALPAK ® AD-H, 8:2 hexane/isopropanol, 1.0 mL/min, 220 nm).

Methyl 4-(2-methoxy-1-(methyl(phenyl)amino)-2-oxoethyl)benzoate (**29**)

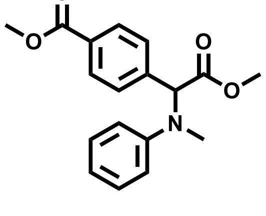

Yield: 69% (21.6 mg); Pale yellow oil; R_f = 0.50 (5% AcOEt/ Hex); ^{1}H NMR (600 MHz, $CDCl_3$): δ= 8.04 (d, *J*= 8.0 Hz, 2H); 7,36 (d, *J*= 8.0 Hz, 2H); 7.31-7.21 (m, 2H); 6.87 (d, *J*= 7.7 Hz, 2H); 6,85-6.81 (m, 1H); 5.68 (s, 1H); 3.92 (s, 3H); 3.79 (s, 3H); 2.80 (s, 3H); ^{13}C NMR (151MHz, $CDCl_3$): δ= 171.8, 166.8, 149.8, 141.1, 130.0, 129.5, 128.4, 118.6, 113.8, 65.8, 52.3, 35.0; HRMS (ESI+qTOF) m/z: calcd for $C_{18}H_{20}NO_4$ $[M+H]^+$ 314.1392; found 314.1392.

$[\alpha]_D^{22}$ = +4.5 (*c* = 0.005 in $CHCl_3$) for 70:30 e.r.

HPLC: t_R = 16.39 min (minor), t_R = 24.25 min (major), 30:70 e.r.; (CHIRALPAK ® AD-H, 9:1 hexane/isopropanol, 1.0 mL/min, 254 nm).

Methyl 2-(4-chlorophenyl)-2-(methyl(phenyl)amino)acetate (**30**)

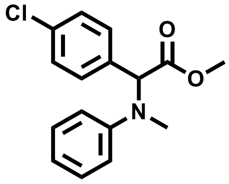

Yield: 75% (21.6 mg); Colorless oil; R_f = 0.34 (2% AcOEt/Hexanes); ^{1}H NMR (500 MHz, $CDCl_3$) δ 7.37 – 7.32 (m, 2H), 7.30 – 7.19 (m, 4H), 6.89 – 6.79 (m, 3H), 5.60 (s, 1H), 3.78 (s, 3H), 2.79 (s, 3H); ^{13}C NMR (151 MHz, $CDCl_3$) δ 172.0, 149.8, 134.5, 134.2, 129.8, 129.5, 129.0, 118.6, 113.8, 65.4, 52.3, 34.8; HRMS (MALDI): *m/z* calcd for $C_{16}H_{17}ClNO_2+H^+$: 290.09423 $[M+H]^+$; found 290.0961.

$[\alpha]_D^{22}$= +62.38 (c = 1.32, $CHCl_3$) for 84:16 e.r.

HPLC: t_R = 7.723 min (min); t_R = 8.803 min (maj), *e.r.* = 16:84 (CHIRALPAK ® AD-H, 98:2 hexane/isopropanol, 1.0 mL/min, 220 nm).

4-(2-methoxy-1-(methyl(phenyl)amino)-2-oxoethyl)phenyl benzoate (**31**)

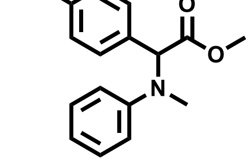

Yield: 44% (16.5 mg); Yellow oil; R_f = 0.47 (10% AcOEt/Hexanes); ^{1}H NMR (400 MHz, $CDCl_3$) δ 8.20 (d, *J* = 7.3 Hz, 2H), 7.65 (t, *J* = 7.4 Hz, 1H), 7.52 (t, *J* = 7.7 Hz, 2H), 7.35 (d, *J* = 8.5 Hz, 2H), 7.27 (m, 4H), 6.89 (d, *J* = 8.2 Hz, 2H), 6.82 (t, *J* = 7.3 Hz, 1H), 5.67 (s, 1H), 3.79 (s, 3H), 2.83 (s, 3H); ^{13}C NMR (151 MHz, $CDCl_3$) δ 172.2, 165.2, 150.8, 149.9, 133.9, 133.5, 130.3, 129.7, 129.5, 129.5, 128.8, 122.1, 118.4, 113.8, 65.4, 52.2, 34.8; HRMS (MALDI): *m/z* calcd for $C_{23}H_{22}NO_4+H^+$: 376.15433 $[M+H]^+$; found 376.1548.

$[\alpha]_D^{22}$= +65.60 (*c* = 1.65 in $CHCl_3$) for 88:12 e.r.

HPLC: t_R = 8.328 min (min); t_R = 9.383 min (maj), *e.r.* = 12:88 (CHIRALPAK ® AD-H, 8:2 hexane/isopropanol, 1.0 mL/min, 254 nm).

Methyl 2-(3,5-dimethylphenyl)-2-(methyl(phenyl)amino)acetate (**32**)

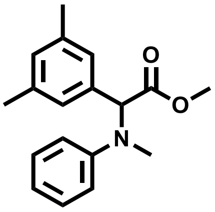

Yield: 50% (14.3 mg); Colorless oil; R_f = 0.44 (2% AcOEt/Hexanes); ^{1}H NMR (500 MHz, $CDCl_3$) δ 7.31 – 7.24 (m, 2H), 6.97 (s, 1H), 6.87 (m, 4H), 6.80 (t, *J* = 7.3 Hz, 1H), 5.60 (s, 1H), 3.77 (s, 3H), 2.79 (s, 3H), 2.31 (s, 6H); ^{13}C NMR (151 MHz, $CDCl_3$) δ 172.8, 150.0, 138.4, 135.8, 130.0, 129.4, 126.4, 118.0, 113.4, 65.7, 52.1, 34.5, 21.5. HRMS (ESI): *m/z* calcd for $C_{18}H_{22}NO_2+H^+$: 284.16451 $[M+H]^+$; found 284.1655.
$[\alpha]_D^{22}$= +86.85 (*c* = 1.43 in $CHCl_3$) for 91:9 e.r.
HPLC: t_R = 5.531 min (maj); t_R = 6.341 min (min), *e.r.* = 91:9 (CHIRALPAK ® AD-H, 98:2 hexane/isopropanol, 1.0 mL/min, 254 nm).

Methyl 2-(2-methoxyphenyl)-2-(methyl(phenyl)amino)acetate (**33**)

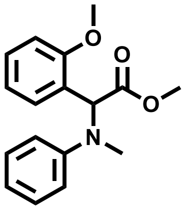

Yield: 50% (15.5 mg); Colorless oil; R_f = 0.29 (5% AcOEt/Hexanes); ^{1}H NMR (600 MHz, $CDCl_3$) δ 7.32 (m, 1H), 7.24 (m, 2H), 7.18 (dd, *J* = 7.5, 1.3 Hz, 1H), 6.99 – 6.88 (m, 2H), 6.86 (d, *J* = 8.6 Hz, 2H), 6.77 (t, *J* = 7.3 Hz, 1H), 5.85 (s, 1H), 3.78 (s, 3H), 3.73 (s, 3H), 2.85 (s, 3H); ^{13}C NMR (151 MHz, $CDCl_3$) δ 172.4, 157.8, 150.1, 129.7, 129.4, 129.2, 124.6, 120.6, 117.8, 113.7, 111.1, 61.7, 55.8, 52.1, 34.0. HRMS (MALDI): *m/z* calcd for $C_{17}H_{19}NO_3+H^+$: 286.14377 $[M+H]^+$; found 286.1456.
$[\alpha]_D^{22}$= +90.58 (*c* = 1.55 in $CHCl_3$) for 88:12 e.r.
HPLC: t_R = 9.927 min (min); t_R = 12.128 min (maj), *e.r.* = 12:88 (Phenomenex Cellulose 4, 98:2 hexane/isopropanol, 1.0 mL/min, 254 nm).

Methyl 2-(benzo[d][1,3]dioxol-5-yl)-2-((4-bromophenyl)(methyl)amino)acetate (**34**)

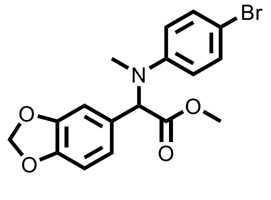

Yield: 74% (28.0 mg); Yellow oil; R_f = 0.49 (95% AcOEt/Hex); ^{1}H NMR (600 MHz, $CDCl_3$): δ= 7.31 (d, *J*= 9.0 Hz, 2H); 6.78 (d, *J*= 8.0 Hz, 1H); 6.72-6.67 (m, 3H); 6.46 (d, *J*= 8.8 Hz, 1H); 5.96 (s, 2H); 5.45 (s, 1H); 3.75 (s, 3H); 2.76 (s, 3H); ^{13}C NMR (151MHz, $CDCl_3$): δ= 172.1, 148.9, 148.2, 147.7, 132.1, 129.1, 122.0, 115.3, 114.0, 109.0, 108.5, 101.5, 65.7, 52.3, 34.6; HRMS (ESI+qTOF) m/z: calcd for $C_{17}H_{17}BrNO_4$ $[M+H]^+$ 378.0341; found 378.0344.

$[\alpha]_D^{22}$ = +34.8 (*c* = 0.001 in $CHCl_3$) for 77:23 e.r.

HPLC: t_R = 10.72 min (minor), t_R = 12.29 min (major), 23:77 e.r.; (CHIRALPAK ® AD-H, 9:1 hexane/isopropanol, 1.0 mL/min, 220 nm).

Methyl 2-((4-bromophenyl)(methyl)amino)-2-(2-methoxyphenyl)acetate (**35**)

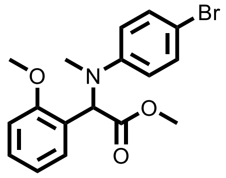

Yield: 68% (24.8 mg); Yellow oil; R_f = 0.50 (5% AcOEt/Hex); ^{1}H NMR (600 MHz, $CDCl_3$): δ 7,38-7.28 (m, 3H); 7.15 (d, *J*= 7.5 Hz, 1H); 6.99-6.89 (m, 2H); 6.76-6.78(m, 2H); 5.79 (s, 1H); 3.78 (s, 3H); 3.74 (s, 3H); 2.80 (s, 3H); ^{13}C NMR (151MHz, $CDCl_3$): δ 174.9, 160.4, 151.8, 134.6, 132.6, 132.1, 126.9, 123.4, 118.0, 113.9, 112.5, 64.3, 58.5, 54.9, 36.7; HRMS (ESI+qTOF) m/z: calcd for $C_{17}H_{19}BrNO_3$ $[M+H]^+$ 364.0548; found 364.0556.

$[\alpha]_D^{22}$ = +37.2 (c = 0.01 in $CHCl_3$) for 86:14 e.r.

HPLC: t_R = 7.17 min (minor), t_R = 7.84 min (major), 14:86 e.r.; (CHIRALPAK ® AD-H, 9:1 hexane/isopropanol, 1.0 mL/min, 220 nm).

Methyl 2-(benzo[d][1,3]dioxol-5-yl)-2-(methyl(p-tolyl)amino)acetate (**36**)

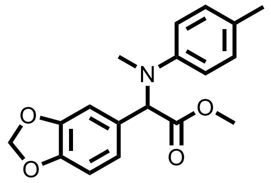

Yield: 67% (21.0 mg); Pale yellow oil; R_f = 0.48 (5% AcOEt/Hex); ^{1}H NMR (600 MHz, $CDCl_3$): δ 7.07 (d, *J*= 9.0 Hz, 2H); 6.81-6.77 (m, 4H); 6.76-6.71 (m, 1H); 5.97 (s, 2H); 5.47 (s, 1H); 3.75 (s, 3H); 2.77 (s, 3H); 2.27 (s, 3H); ^{13}C NMR (151MHz, $CDCl_3$): δ 172.6, 148.1, 147.9, 147.6, 129.9, 127.8, 122.0, 114.3, 109.1,

108.4, 101.4, 66.2, 52.1, 34.9, 20.5. HRMS (ESI+qTOF) m/z: calcd for $C_{18}H_{20}NO_4$ $[M+H]^+$ 314.1392; found 314.1393.

$[\alpha]_D^{22}$ = +31.9 (c = 0.02 in $CHCl_3$) for 77:23 e.r.

HPLC: t_R = 7.58 min (minor), t_R = 9.02 min (major), 23:77 e.r.; (CHIRALPAK ® AD-H, 9:1 hexane/isopropanol, 1.0 mL/min, 220 nm, 22 °C).

Methyl 2-((4-bromophenyl)amino)-2-(2-methoxyphenyl)acetate (**37**)

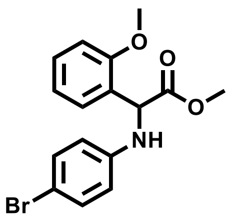

Yield: 74% (25.8 mg); White solid; R_f = 0.31 (10% AcOEt/Hexanes); ^{1}H NMR (600 MHz, $CDCl_3$) δ 7.28 (m, 2H), 7.19 (d, J = 8.8 Hz, 2H), 6.93 (m, 2H), 6.48 (d, J = 8.7 Hz, 2H), 5.46 (s, 1H), 4.93 (s, 1H), 3.91 (s, 3H), 3.70 (s, 3H); ^{13}C NMR (151 MHz, $CDCl_3$) δ 172.7, 157.2, 145.4, 132.0, 129.8, 128.2, 126.0, 121.2, 115.2, 111.3, 109.8, 56.0, 54.7, 52.8. HRMS (ESI): m/z calcd for $C_{16}H_{16}BrNO_3+H^+$: 350.03863 $[M+H]^+$; found 350.0391.

Sample recrystallized from 10% AcOEt/Hex: white needles (57% yield, enantioenriched crystals); m.p. 121-122 °C; $[\alpha]_D^{21}$= +67.2 (c = 1.26 in $CHCl_3$) for 99:1 e.r.

HPLC: t_R = 9.709 min (min); t_R = 12.396 min (maj), *e.r.* = 18:82 (Phenomenex Amylose 2, 9:1 hexane/isopropanol, 1.0 mL/min, 254 nm); t_R = 9.925 min (min); t_R = 12.452 min (maj), *e.r.* = 1:99 (Phenomenex Amylose 2, 9:1 hexane/isopropanol, 1.0 mL/min, 254 nm).

Methyl 2-((4-bromophenyl)amino)-2-(3,5-dimethylphenyl)acetate (**38**)

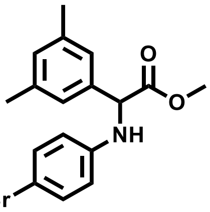

Yield: 72% (25.0 mg); White solid; R_f = 0.45 (5% AcOEt/Hexanes); m.p. 115-116 °C; ^{1}H NMR (400 MHz, $CDCl_3$) δ 7.21 – 7.14 (m, 2H), 7.05 (s, 2H), 6.94 (s, 1H), 6.48 – 6.37 (m, 2H), 4.92 (s, 2H), 3.72 (s, 3H), 2.29 (s, 6H); ^{13}C NMR (101 MHz, $CDCl_3$) δ 172.4, 145.2, 138.7, 137.0, 132.0, 130.4, 125.0, 115.0, 109.8, 60.7, 53.0, 21.4. In good agreement with previously reported data.[1]

$[\alpha]_D^{22}$= +66.34 (c = 2.35 in $CHCl_3$) for e.r.: 85:15.

HPLC: t_R = 6.289 min (min); t_R = 7.615 min (maj), *e.r.* = 15:85 (Phenomenex Amylose 2, 9:1 hexane/isopropanol, 1.0 mL/min, 254 nm).

Methyl 2-((4-bromophenyl)amino)-2-(4-chlorophenyl)acetate (**39**)

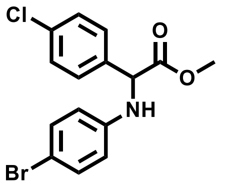

Yield: 74% (25.6 mg); White solid; R_f = 0.34 (5% AcOEt/Hexanes); m.p. 119-120 °C; ^{1}H NMR (400 MHz, $CDCl_3$) δ 7.43 – 7.36 (m, 2H), 7.36 – 7.28 (m, 2H), 7.23 – 7.15 (m, 2H), 6.45 – 6.33 (m, 2H), 5.04 (br s, 1H), 4.99 (s, 1H), 3.73 (s, 3H). ^{13}C NMR (101 MHz, $CDCl_3$) δ 171.6, 144.6, 135.8, 134.5, 132.1, 129.3, 128.6, 115.1, 110.2, 60.1, 53.2. In good agreement with previously reported data.[1]

$[\alpha]_D^{22}$= +49.35 (c = 2.46 in $CHCl_3$) for 76:24 e.r.

HPLC: t_R = 11.765 min (maj); t_R = 16.201 min (min), *e.r.* = 76:24 (CHIRALPAK ® AD-H, 9:1 hexane/isopropanol, 1.0 mL/min, 220 nm).

2-((4-chlorophenyl)amino)-2-phenylacetic acid (**40**)

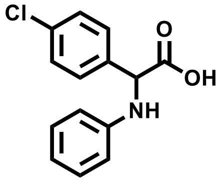

Compound **11** (24.6 mg, 0.089 mmol) was dissolved in THF/H_2O (2:1, 1 mL) and LiOH.H_2O (9.4 mg, 0.22 mmol) was added at 0 °C. The mixture was stirred at 0°C for 1 h, then 10 mL of ethyl acetate was added and acidified with HCl 2% to pH 3. The resulting phases were separated, and the aqueous phase was additionally extracted with EtOAc (2x3 mL). The combined organic phases were dried over anhydrous Na_2SO_4 and concentrated under reduced pressure to yield the titled compound (15.9 mg, 68%) as a white solid.

^{1}H NMR (500 MHz, DMSO-d_6) δ 12.94 (s, 1H), 7.53 – 7.46 (m, 2H), 7.37 (m, 2H), 7.33 – 7.27 (m, 1H), 7.10 – 7.02 (m, 2H), 6.73 – 6.63 (m, 2H), 5.09 (s, 1H). ^{13}C NMR (126 MHz, DMSO-d_6) δ 172.7, 145.9, 138.2, 129.4, 128.5, 128.4, 127.8, 127.5, 114.5, 59.7. This compound in known.[10,11]

HPLC: enantiomeric ratio for this compound was determined from its methyl ester after derivatization with (Trimethylsilyl)diazomethane (solution, 2.0 M in diethyl ether): t_R = 7.697 min (min); t_R = 9.330 min (maj), *e.r.* = 11:89 (Phenomenex Cellulose 1, 95:5 hexane/isopropanol, 1.0 mL/min, 220 nm).

5. Data for the Hammet Plot

Table S3. Data for the Hammet plot (reactions using *N*-methylaniline)

p-Substituted sulfoxonium ylide	σ_p	Maj	Min	Maj/Min	log (Maj/Min)
H	0	92	8	11,5	1,06069784
Obz	0,13	88	12	7,333333	0,865301426
Cl	0,23	84	16	5,25	0,720159303
COOMe	0,45	70	30	2,333333	0,367976785
COMe	0,5	61	39	1,564103	0,194265228

6. ^{1}H and ^{13}C NMR Spectra

Methyl 2-(dimethyl(oxo)-λ6-sulfaneylidene)-2-phenylacetate ^{1}H NMR (400 MHz, $CDCl_3$)

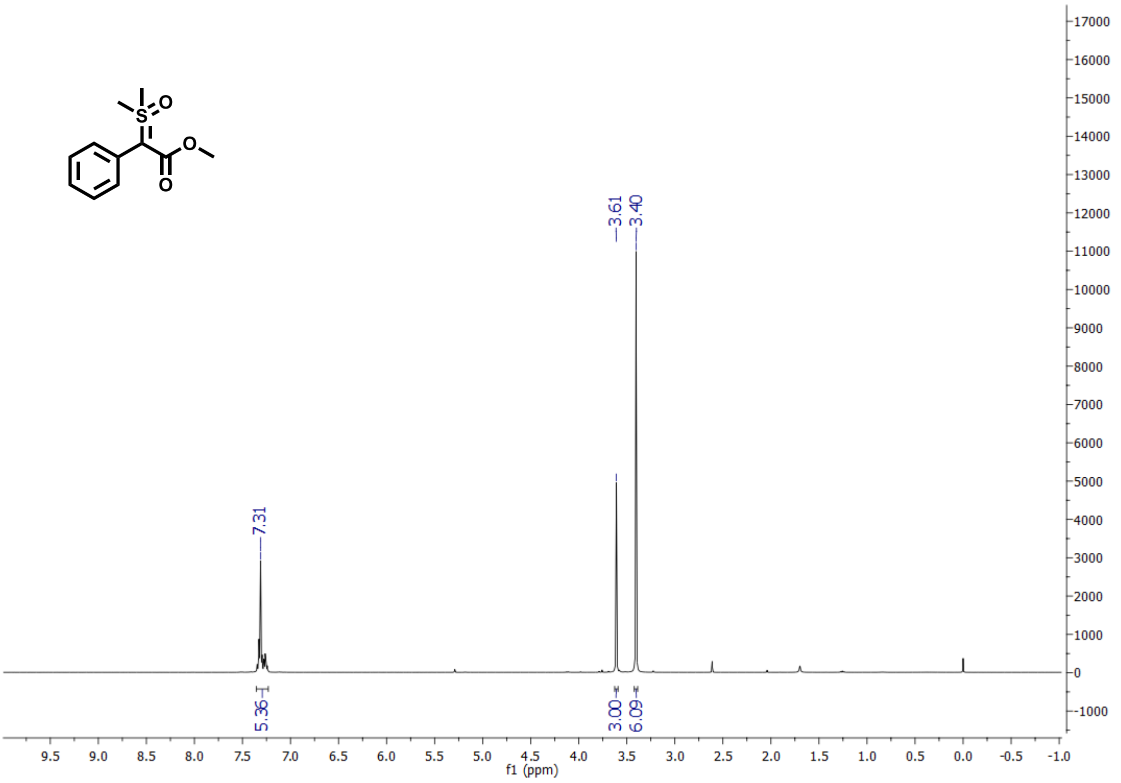

Methyl 2-(dimethyl(oxo)-λ6-sulfaneylidene)-2-phenylacetate ^{13}C NMR (101 MHz, $CDCl_3$)

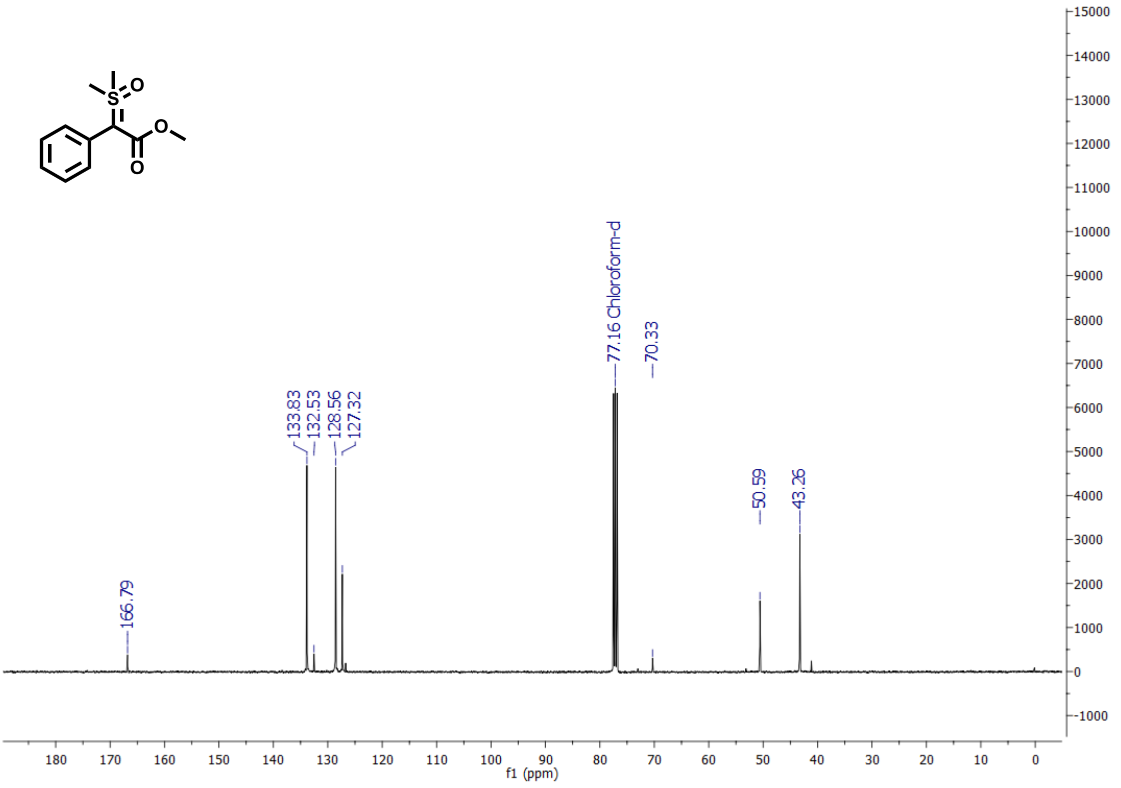

Phenyl 2-(dimethyl(oxo)-λ^6-sulfanylidene) 2-phenylacetate ^{1}H NMR (400 MHz, $CDCl_3$)

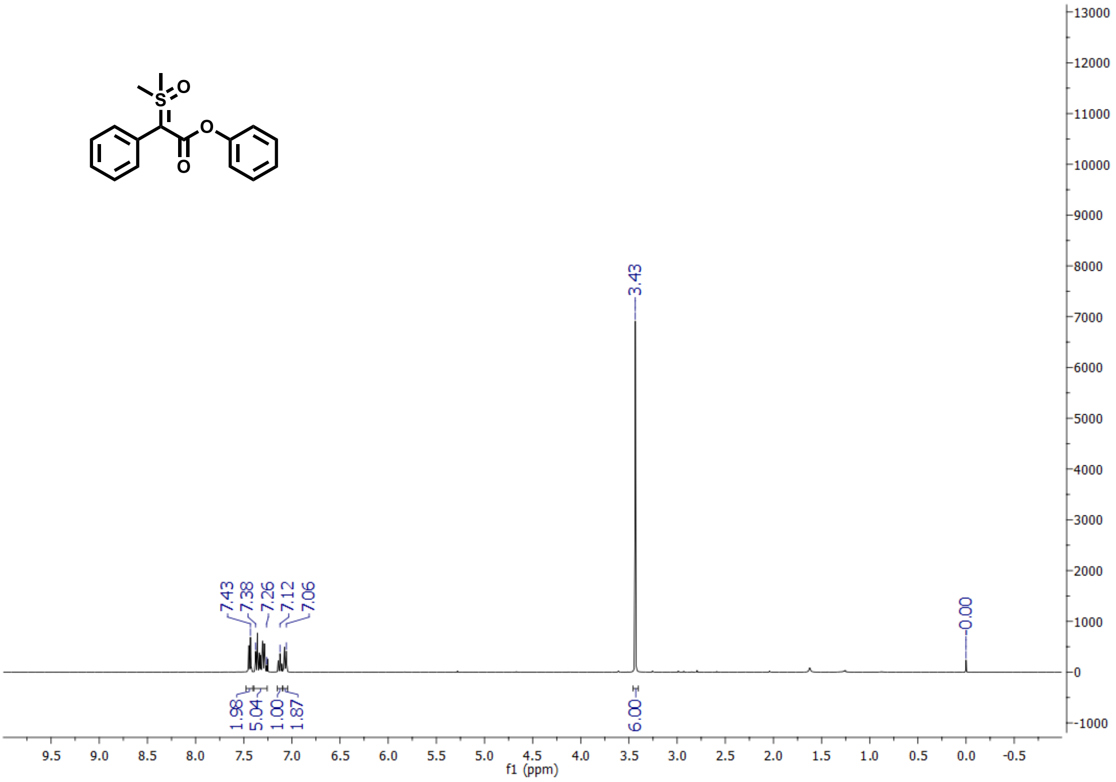

Phenyl 2-(dimethyl(oxo)-λ^6-sulfanylidene) 2-phenylacetate ^{13}C NMR (126 MHz, $CDCl_3$)

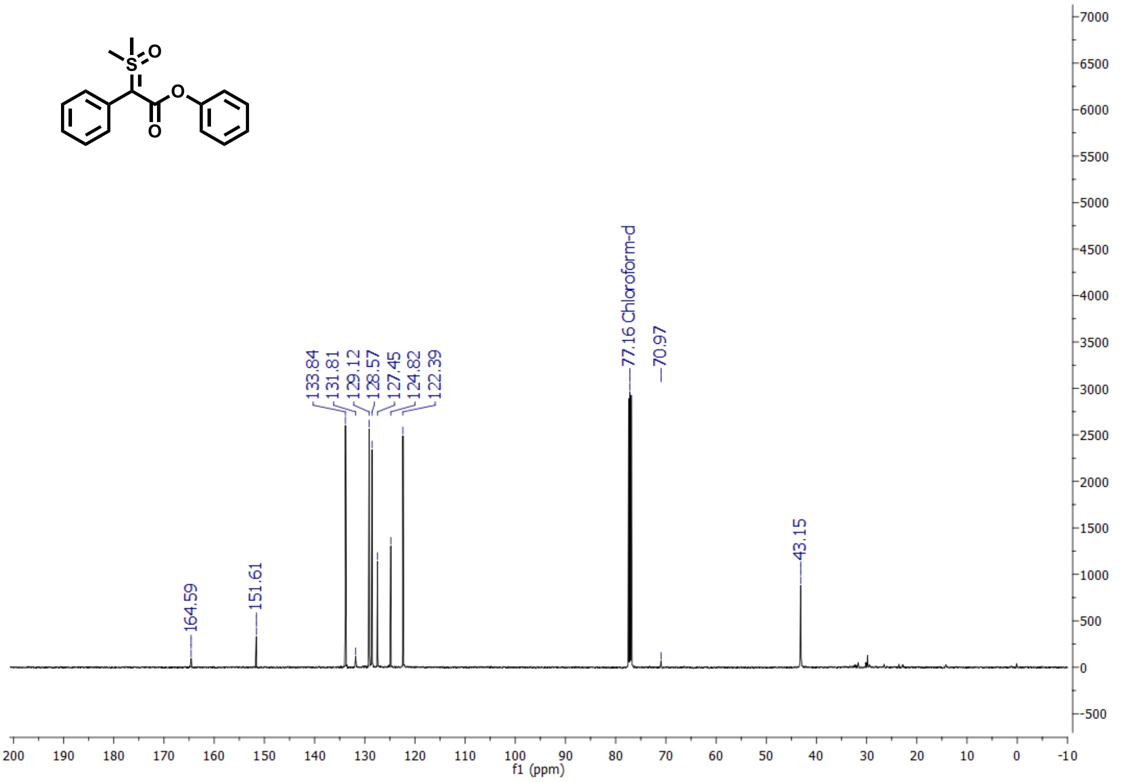

Benzyl 2-(dimethyl(oxo)-λ^6-sulfanylidene) 2-phenylacetate ^{1}H NMR (500 MHz, $CDCl_3$)

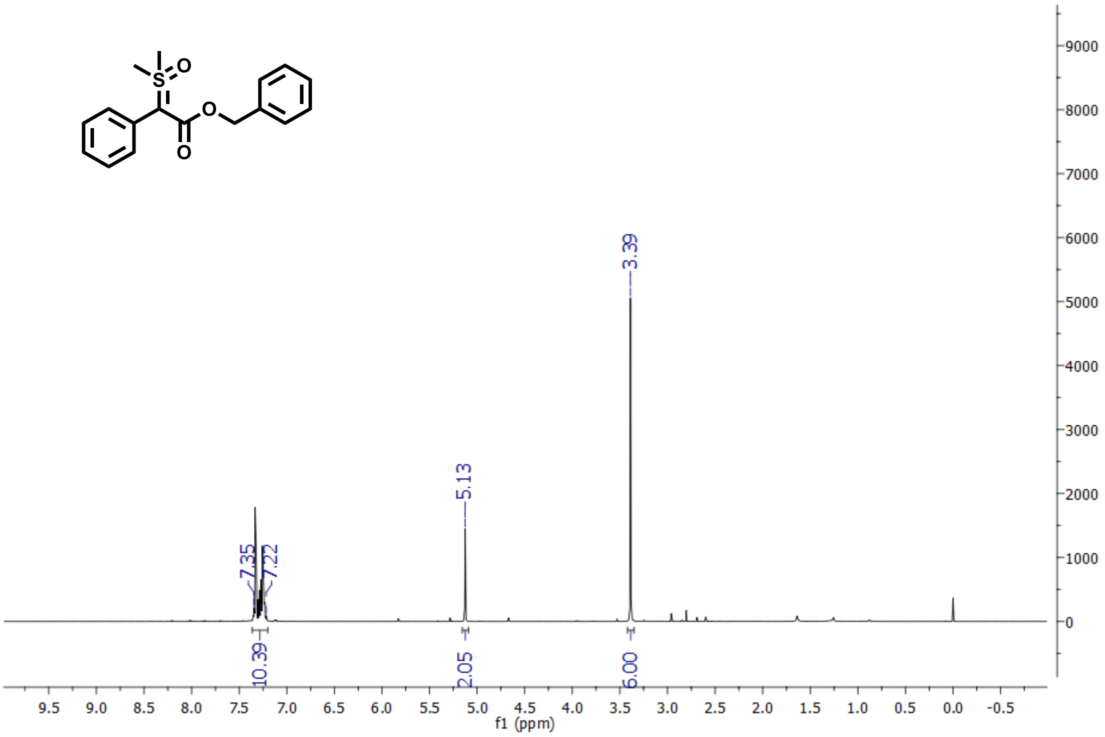

Benzyl 2-(dimethyl(oxo)-λ^6-sulfanylidene) 2-phenylacetate ^{13}C NMR (101 MHz, $CDCl_3$)

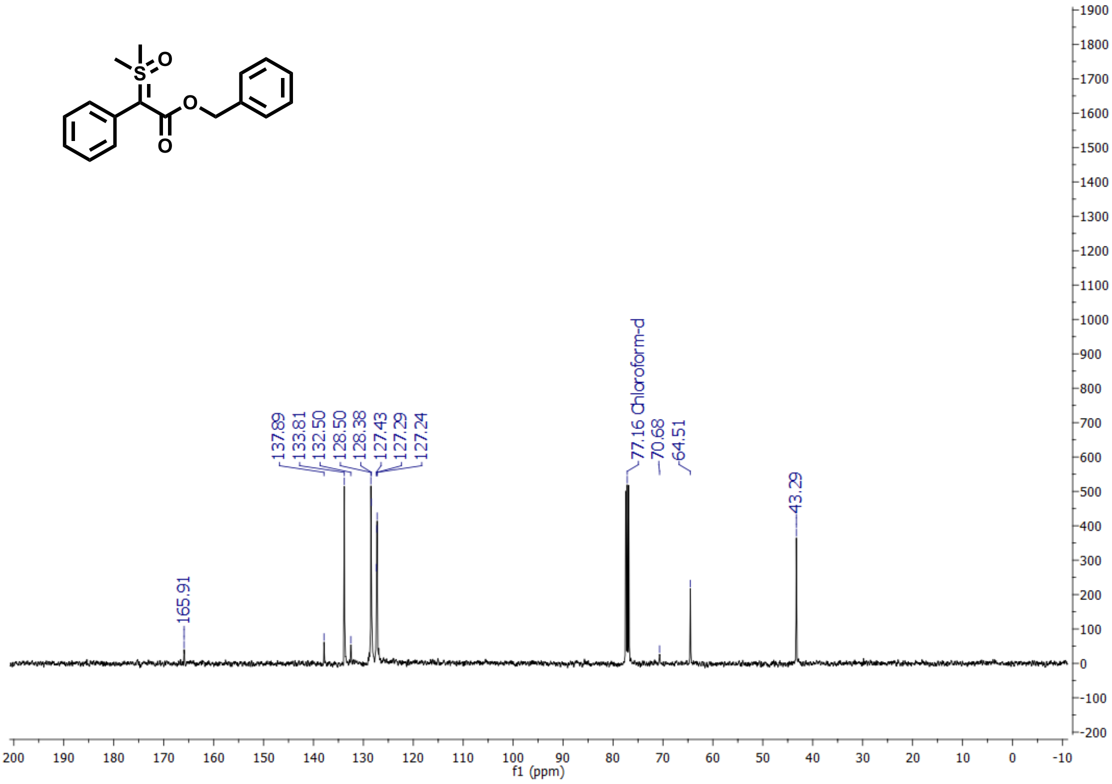

t-Butyl 2-(dimethyl(oxo)-λ[6]-sulfanylidene) 2-phenylacetate ^{1}H NMR (400 MHz, $CDCl_3$)

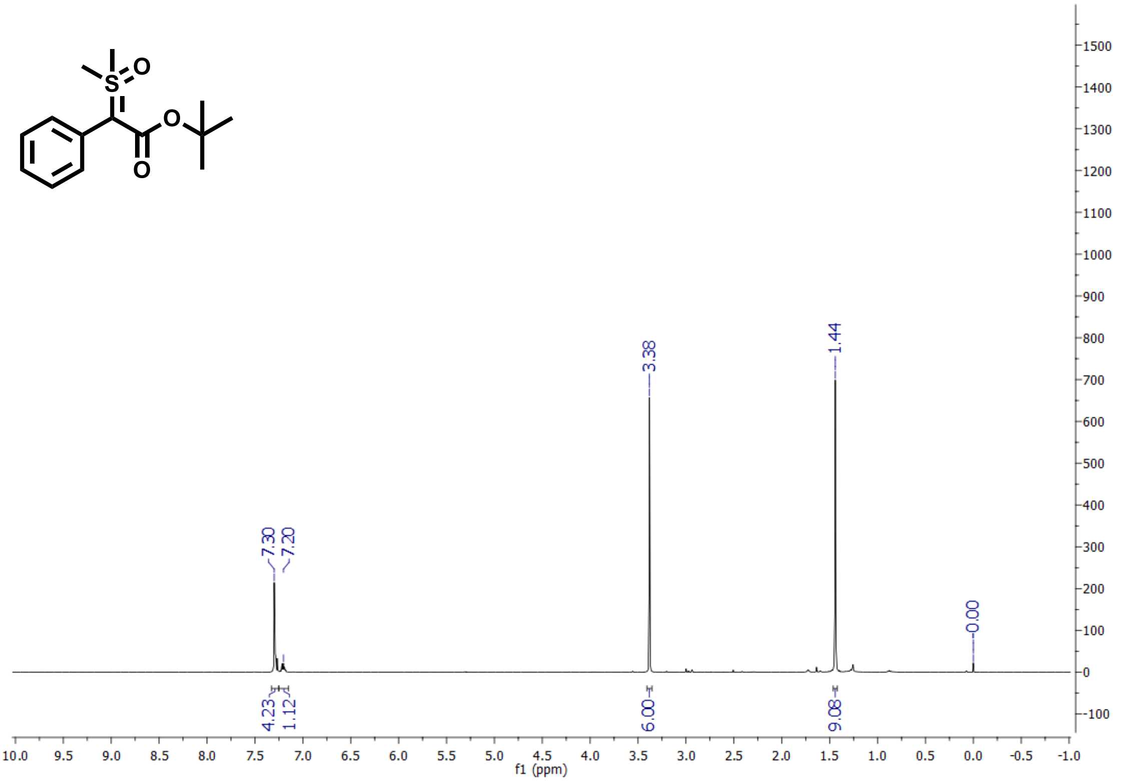

t-Butyl 2-(dimethyl(oxo)-λ[6]-sulfanylidene) 2-phenylacetate ^{13}C NMR (126 MHz, $CDCl_3$)

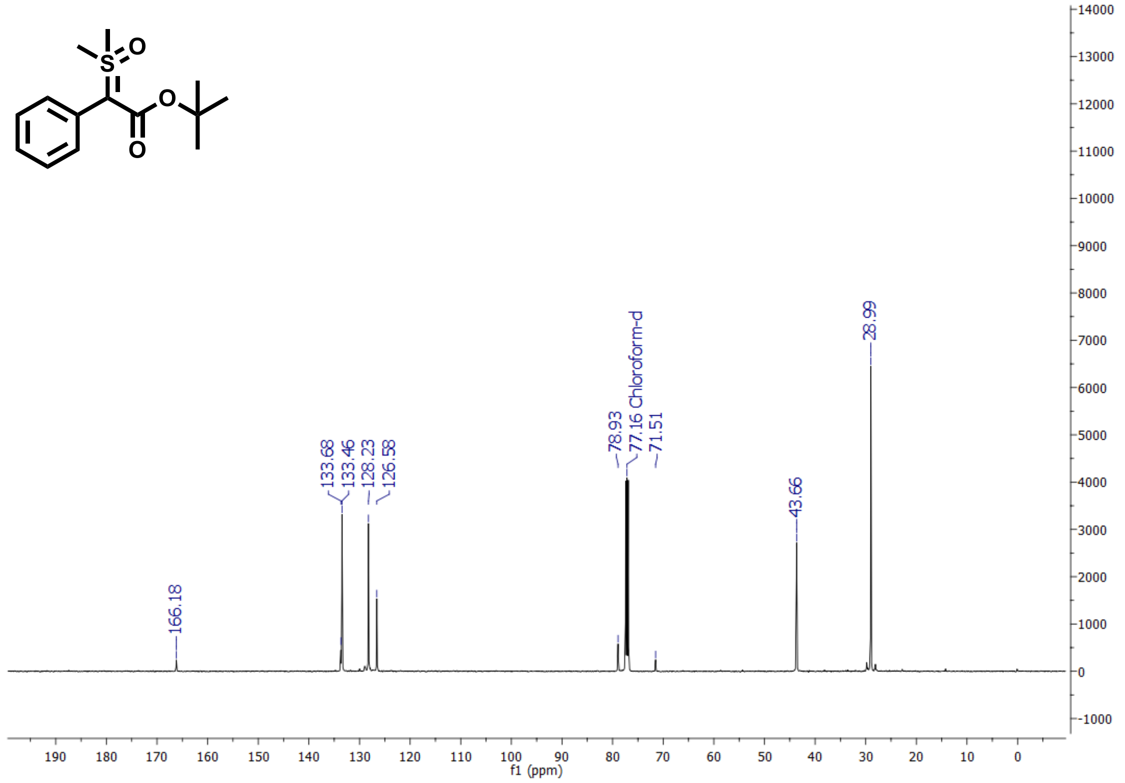

Methyl 2-(4-acetylphenyl)-2-(dimethyl(oxo)-λ6-sulfaneylidene)acetate ^{1}H NMR (400 MHz, $CDCl_3$)

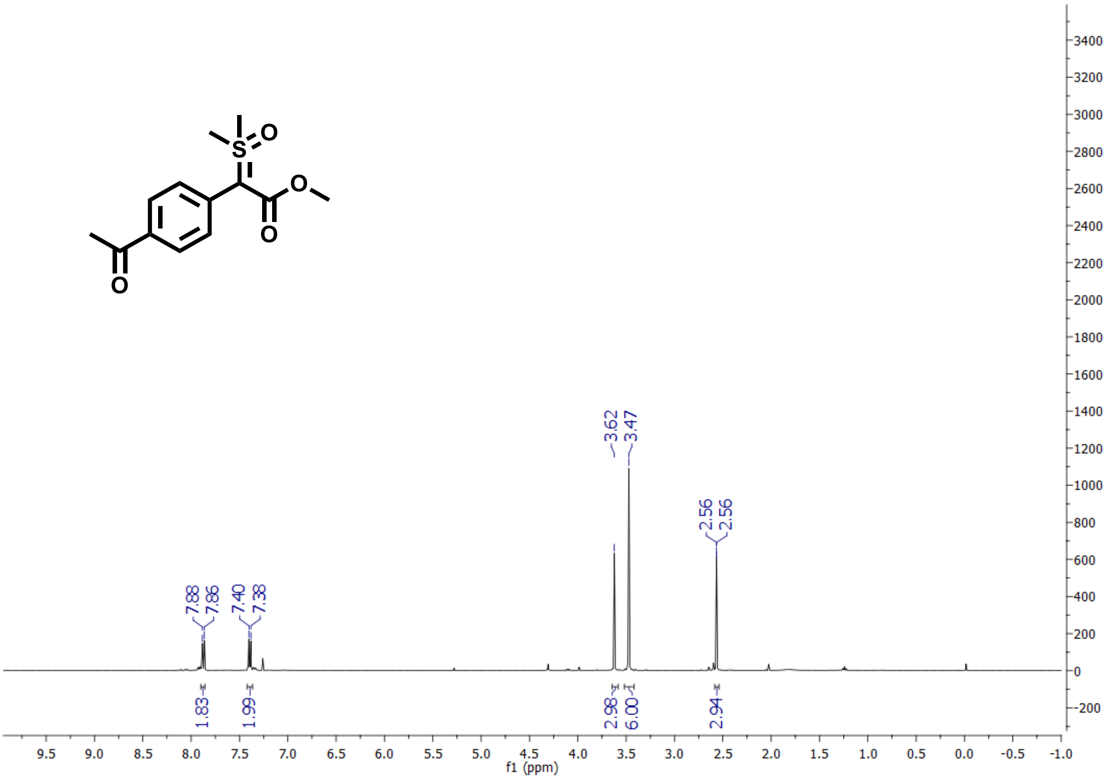

Methyl 2-(4-acetylphenyl)-2-(dimethyl(oxo)-λ6-sulfaneylidene)acetate ^{13}C NMR (126 MHz, $CDCl_3$)

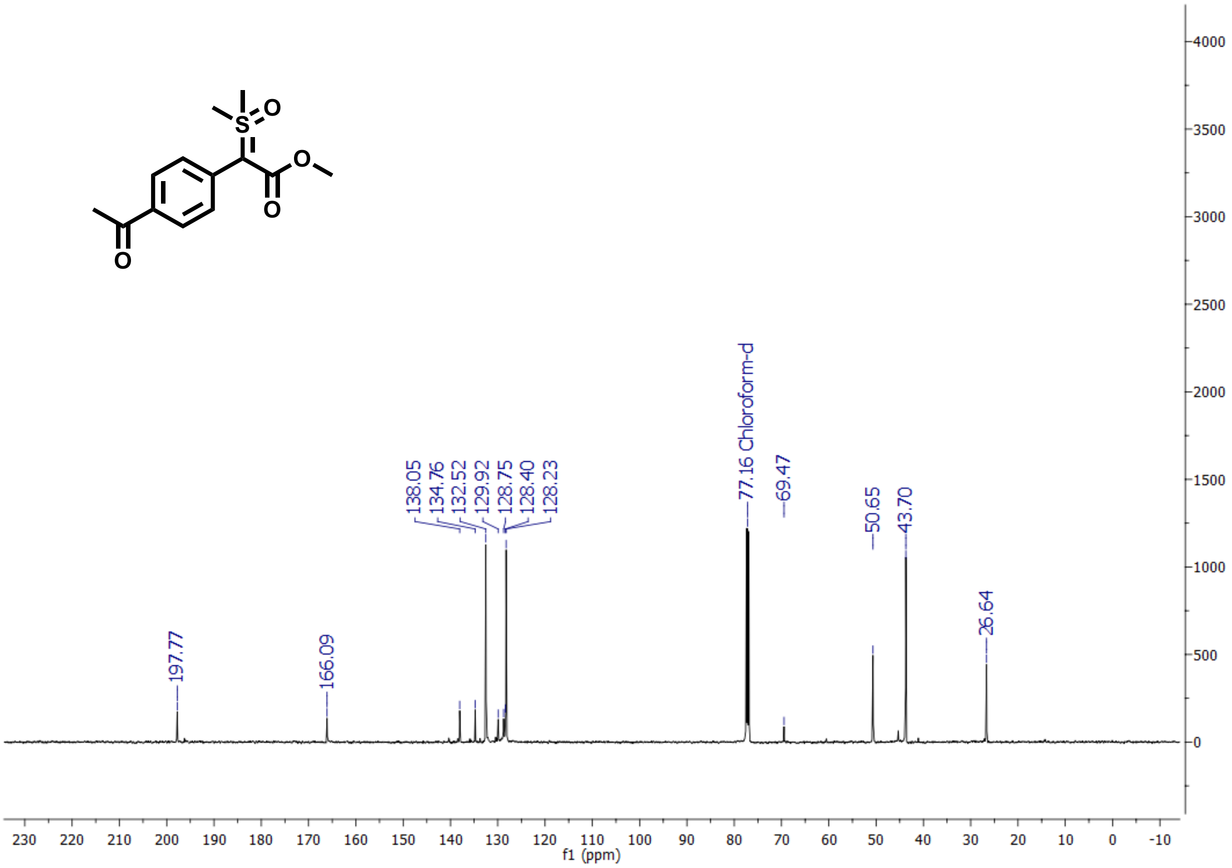

Methyl 4-(1-(dimethyl(oxo)- λ6-sulfaneylidene)-2-methoxy-2-oxoethyl)benzoate ^{1}H NMR (500 MHz, $CDCl_3$)

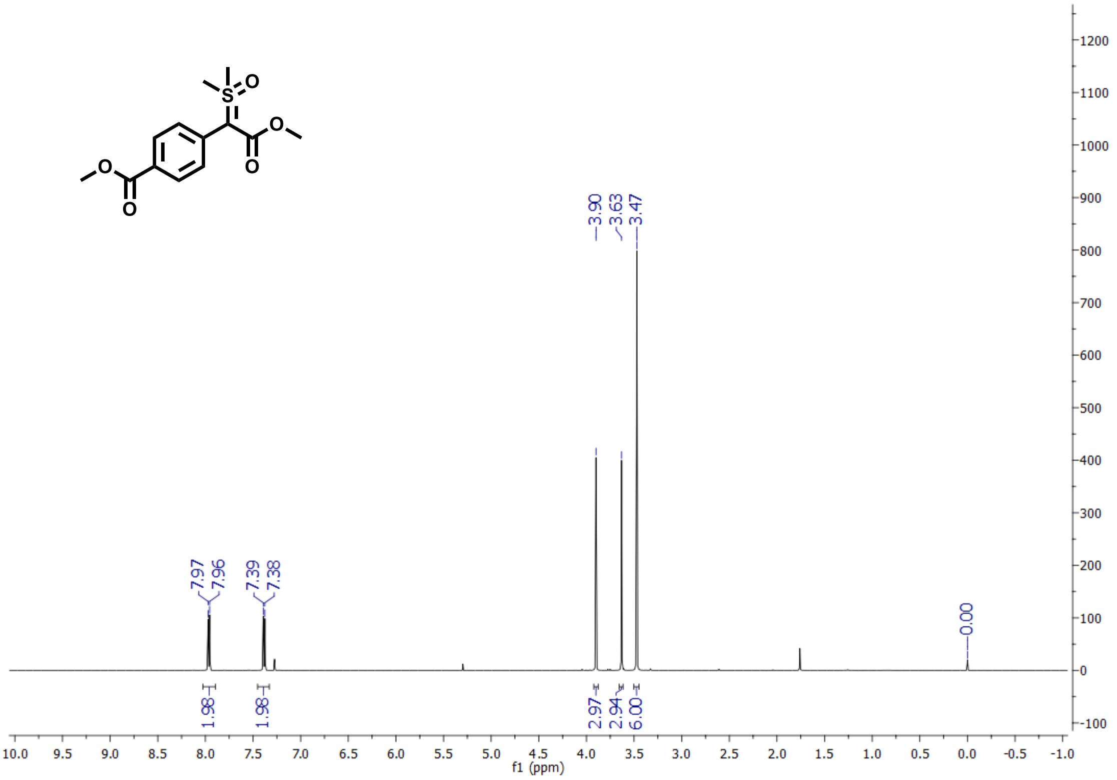

Methyl 4-(1-(dimethyl(oxo)- λ6-sulfaneylidene)-2-methoxy-2-oxoethyl)benzoate ^{13}C NMR (126 MHz, $CDCl_3$)

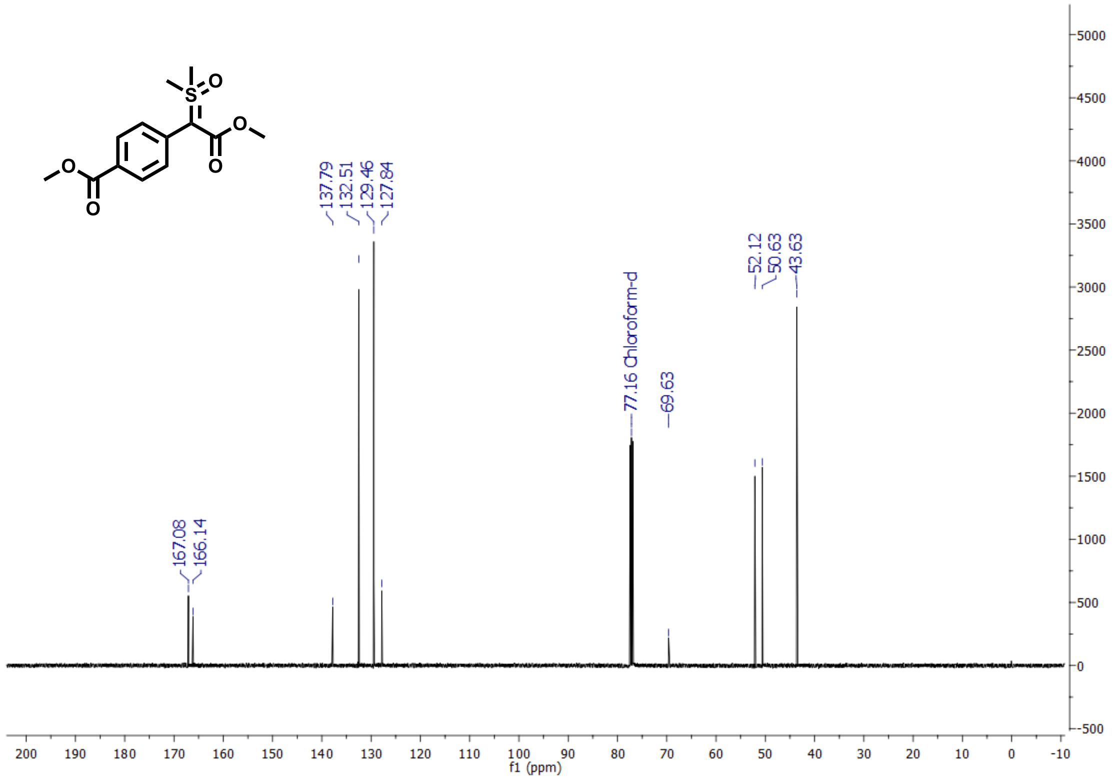

Methyl 2-(4-chlorophenyl)-2-(dimethyl(oxo)-λ6-sulfaneylidene)acetate ^{1}H NMR (400 MHz, $CDCl_3$)

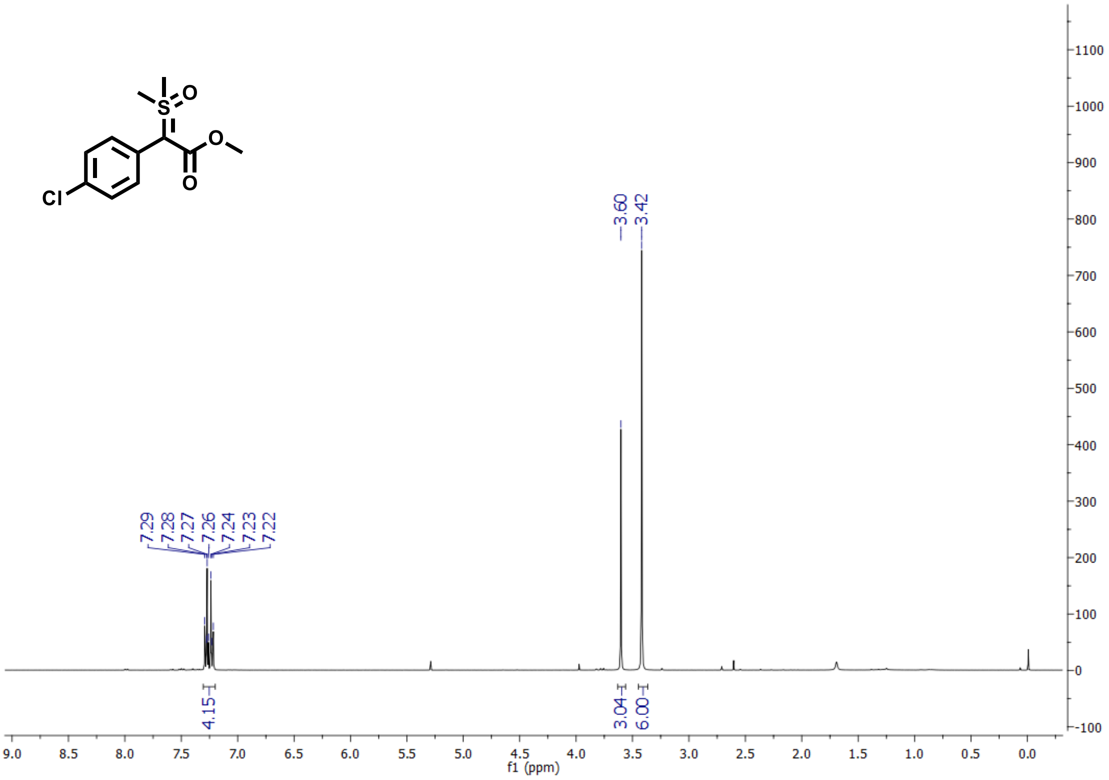

Methyl 2-(4-chlorophenyl)-2-(dimethyl(oxo)-λ6-sulfaneylidene)acetate ^{13}C NMR (126 MHz, $CDCl_3$)

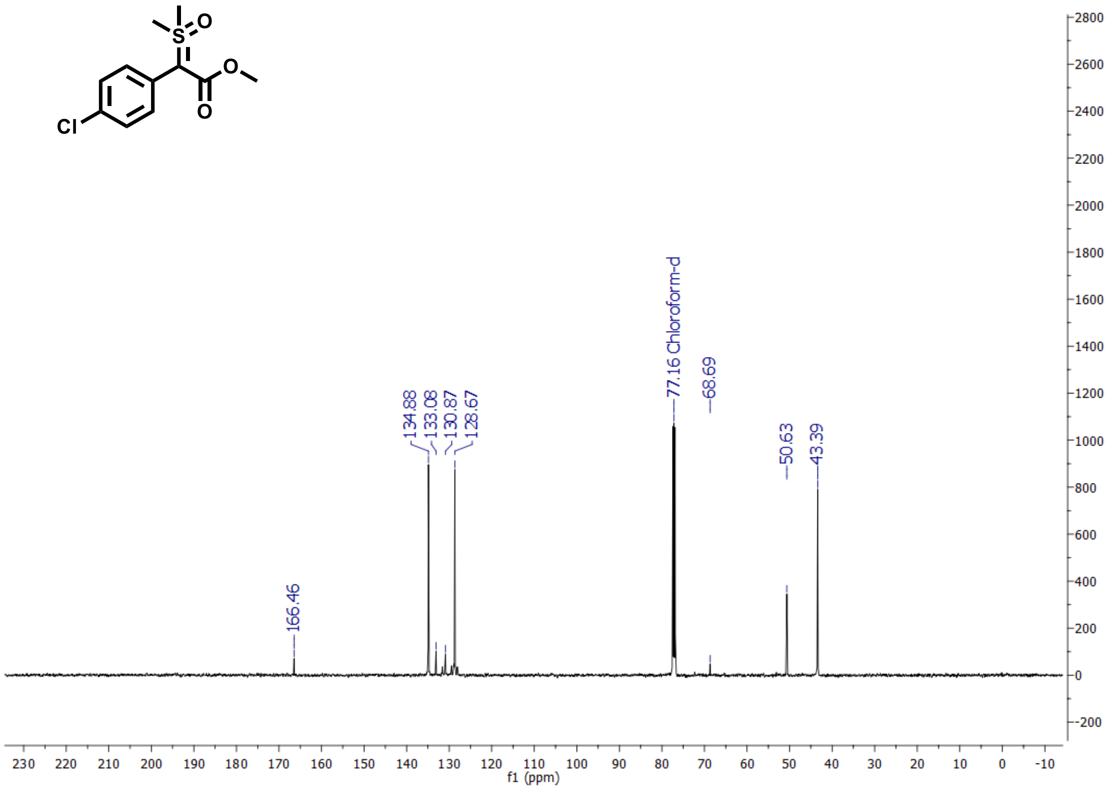

4-(1-(dimethyl(oxo)- λ6-sulfaneylidene)-2-methoxy-2-oxoethyl)phenyl benzoate ^{1}H NMR (400 MHz, $CDCl_3$)

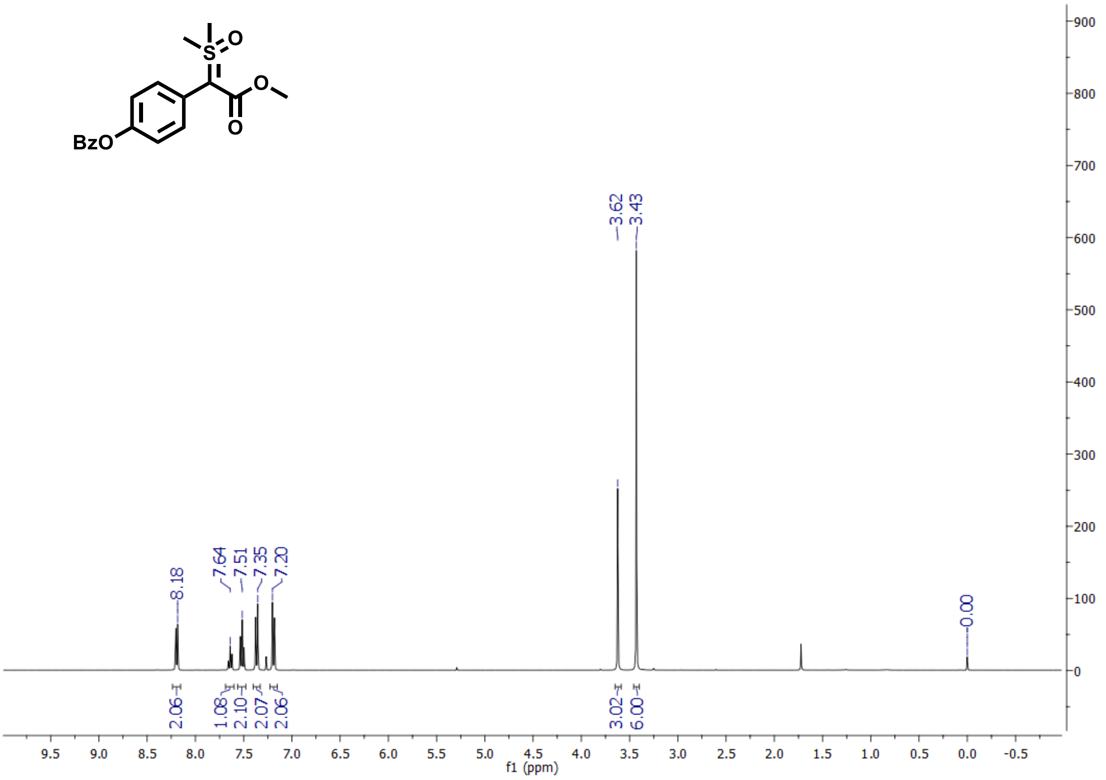

4-(1-(dimethyl(oxo)- λ6-sulfaneylidene)-2-methoxy-2-oxoethyl)phenyl benzoate ^{13}C NMR (400 MHz, $CDCl_3$)

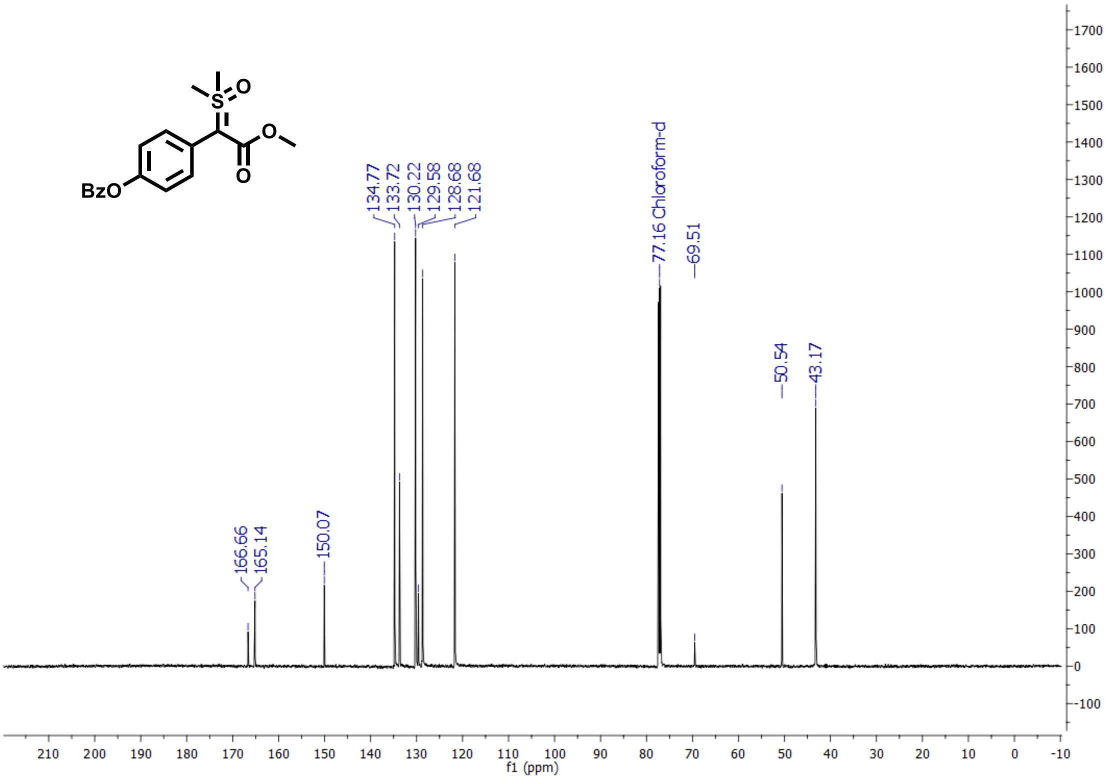

Methyl 2-(dimethyl(oxo)-λ6-sulfaneylidene)-2-(3,5-dimethylphenyl)acetate ^{1}H NMR (500 MHz, $CDCl_3$)

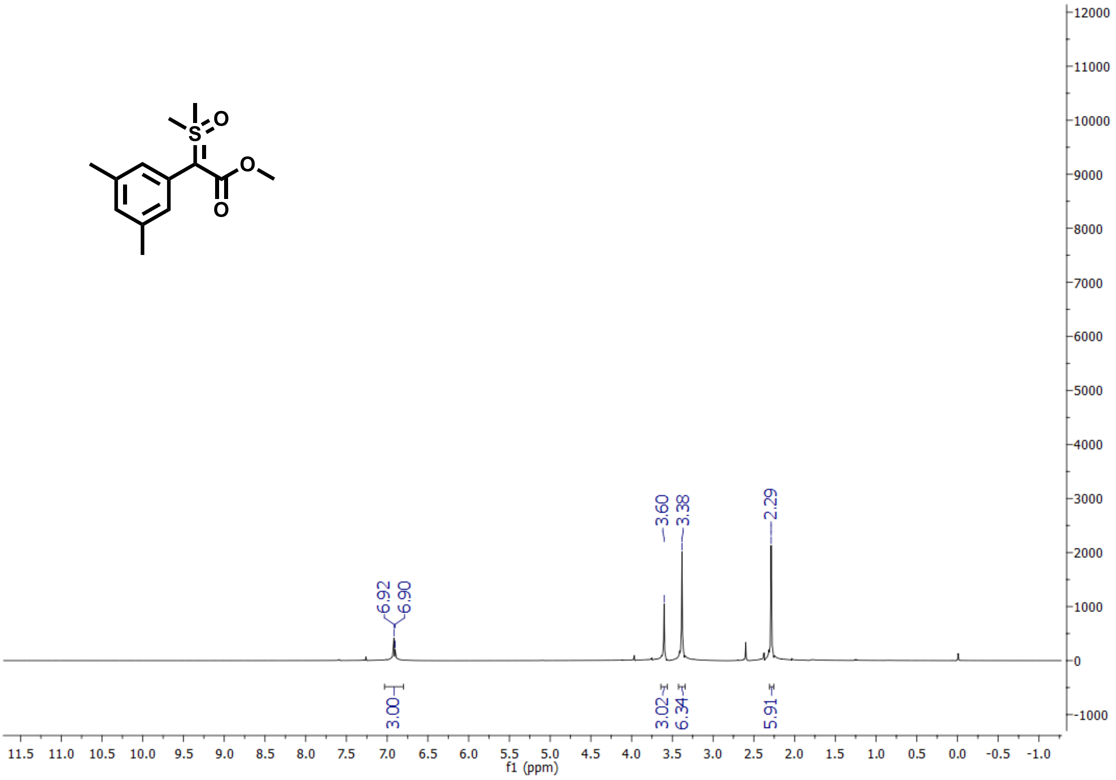

Methyl 2-(dimethyl(oxo)-λ6-sulfaneylidene)-2-(3,5-dimethylphenyl)acetate ^{13}C NMR (126 MHz, $CDCl_3$)

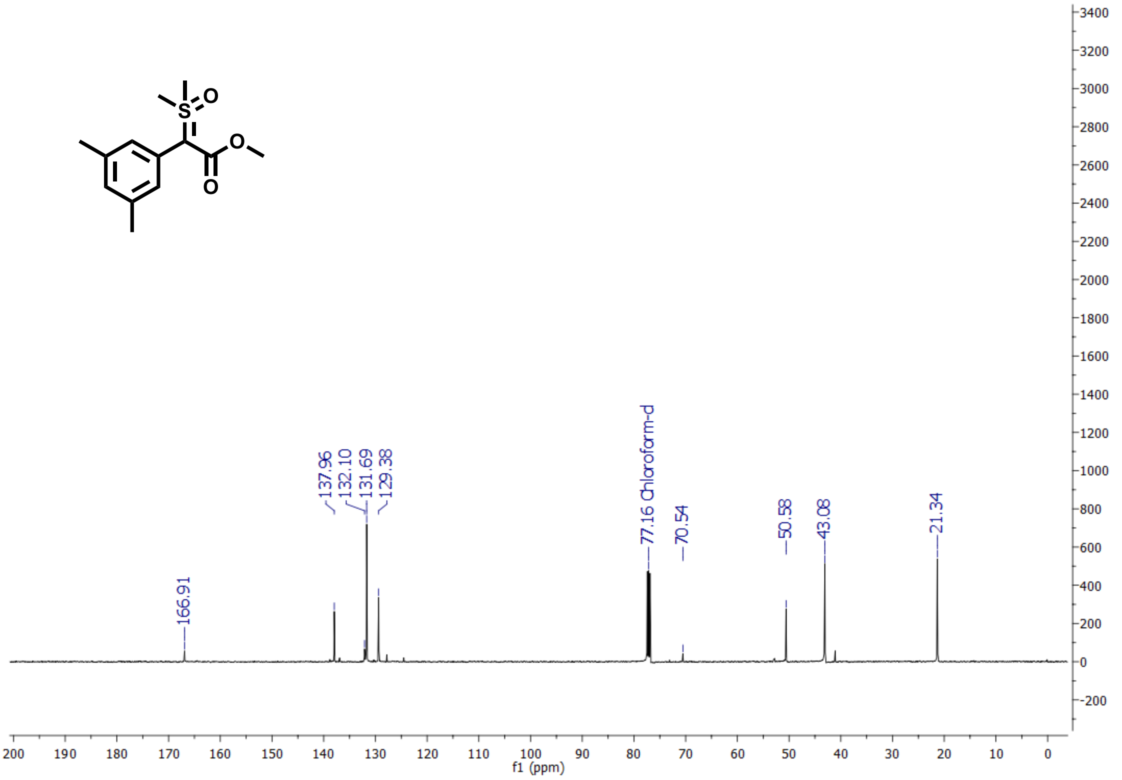

Methyl 2-(dimethyl(oxo)-λ6-sulfaneylidene)-2-(2-methoxyphenyl)acetate ^{1}H NMR (600 MHz, $CDCl_3$)

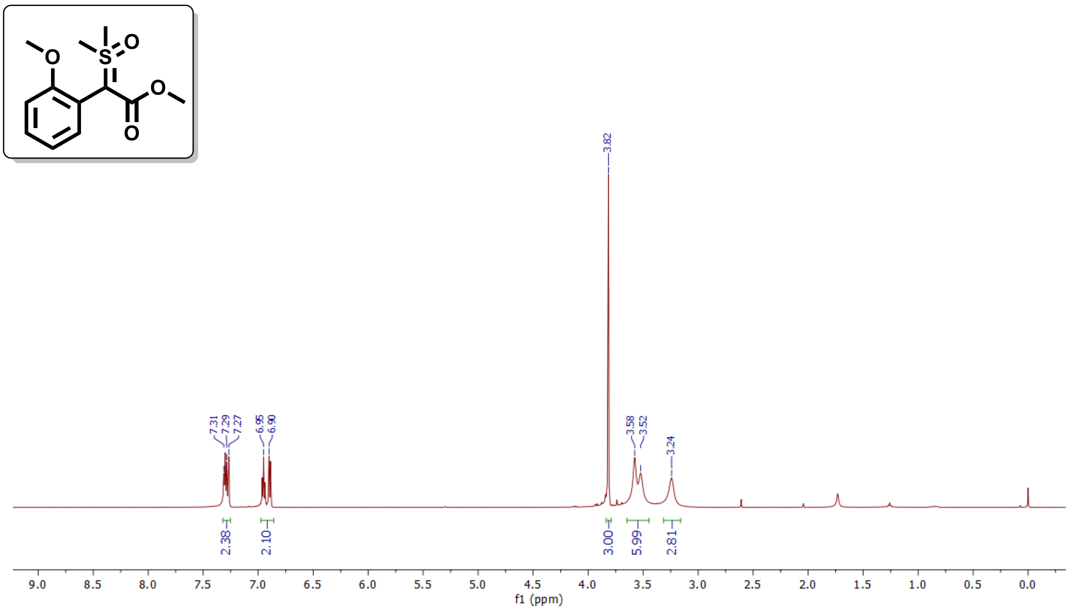

Methyl 2-(dimethyl(oxo)-λ6-sulfaneylidene)-2-(2-methoxyphenyl)acetate ^{1}H NMR (151 MHz, $CDCl_3$)

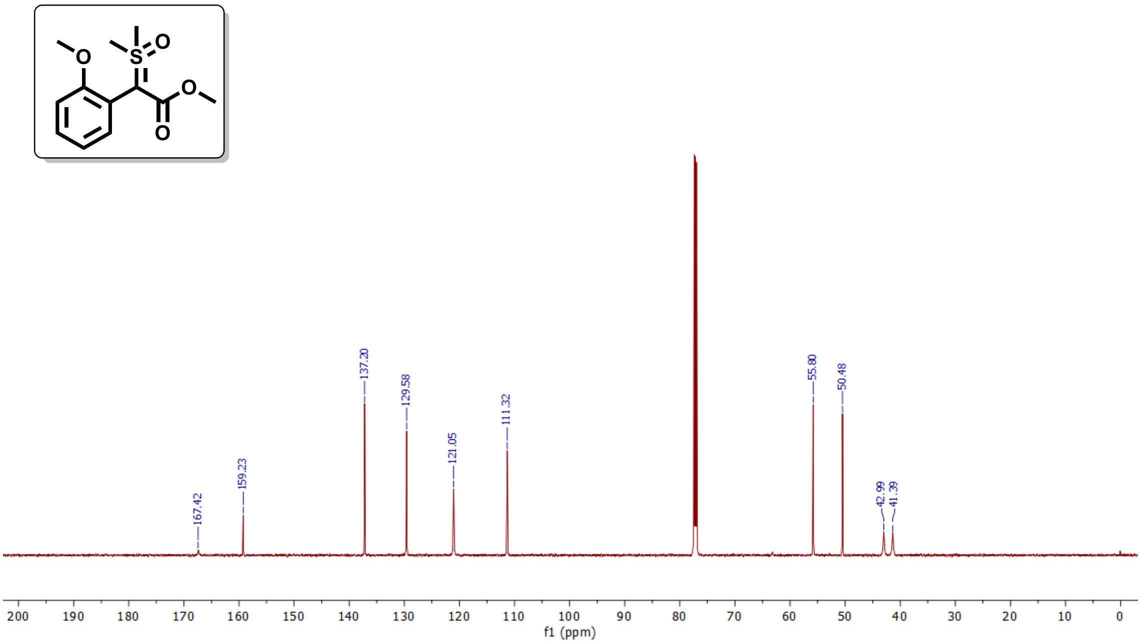

Methyl 2-(benzo[d][1,3]dioxol-5-yl)-2-(dimethyl(oxo)-λ6-sulfaneylidene)acetate ^{1}H NMR (600 MHz, $CDCl_3$)

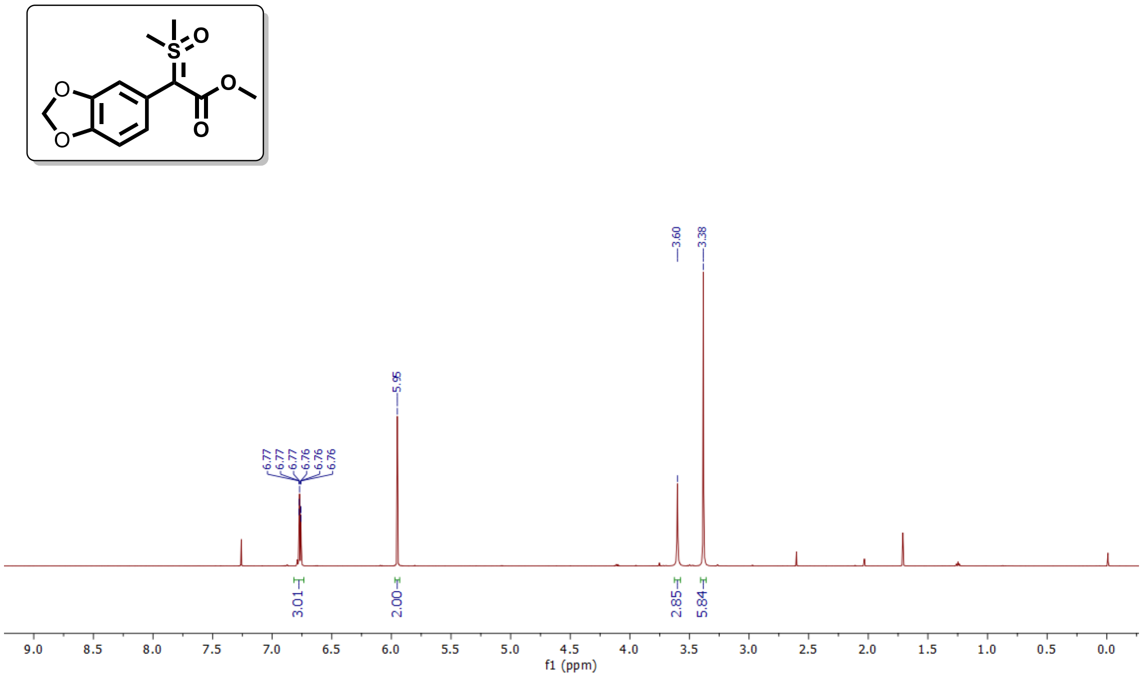

Methyl 2-(benzo[d][1,3]dioxol-5-yl)-2-(dimethyl(oxo)-λ6-sulfaneylidene)acetate ^{1}H NMR (600 MHz, $CDCl_3$)

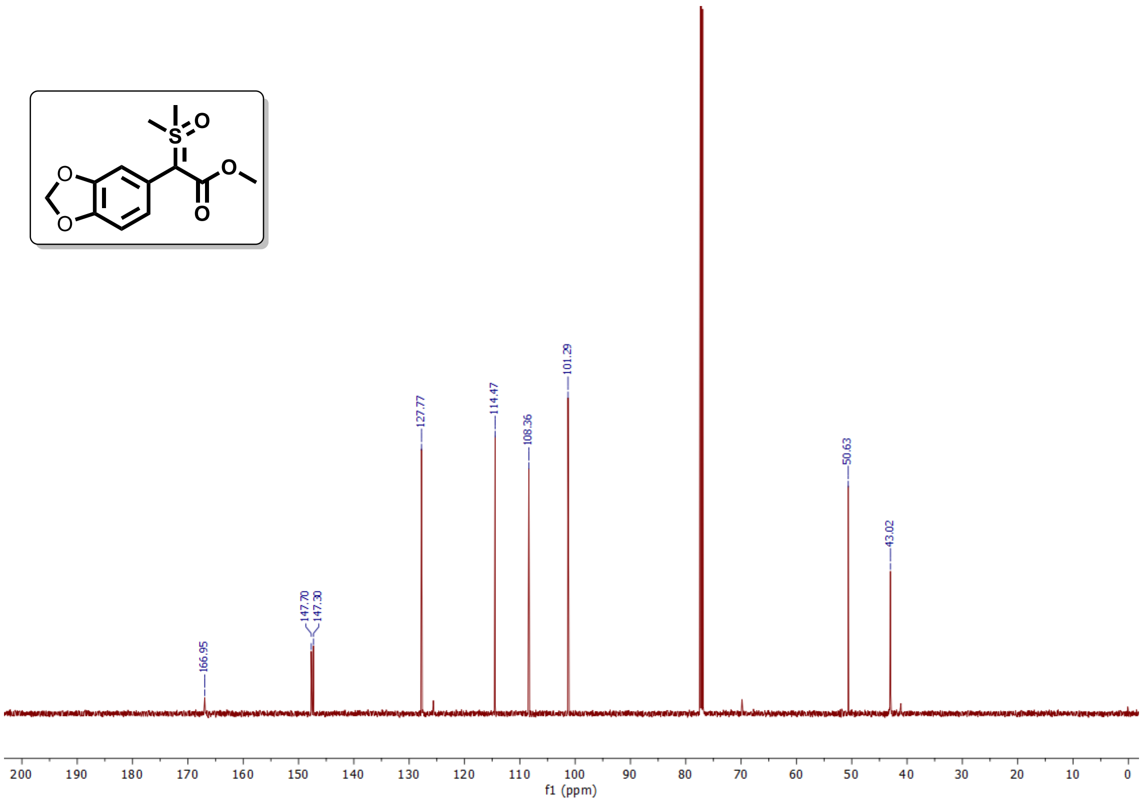

(S)-Methyl 2-phenyl-2-(phenylamino)acetate (**3**) ^{1}H NMR (500 MHz, $CDCl_3$)

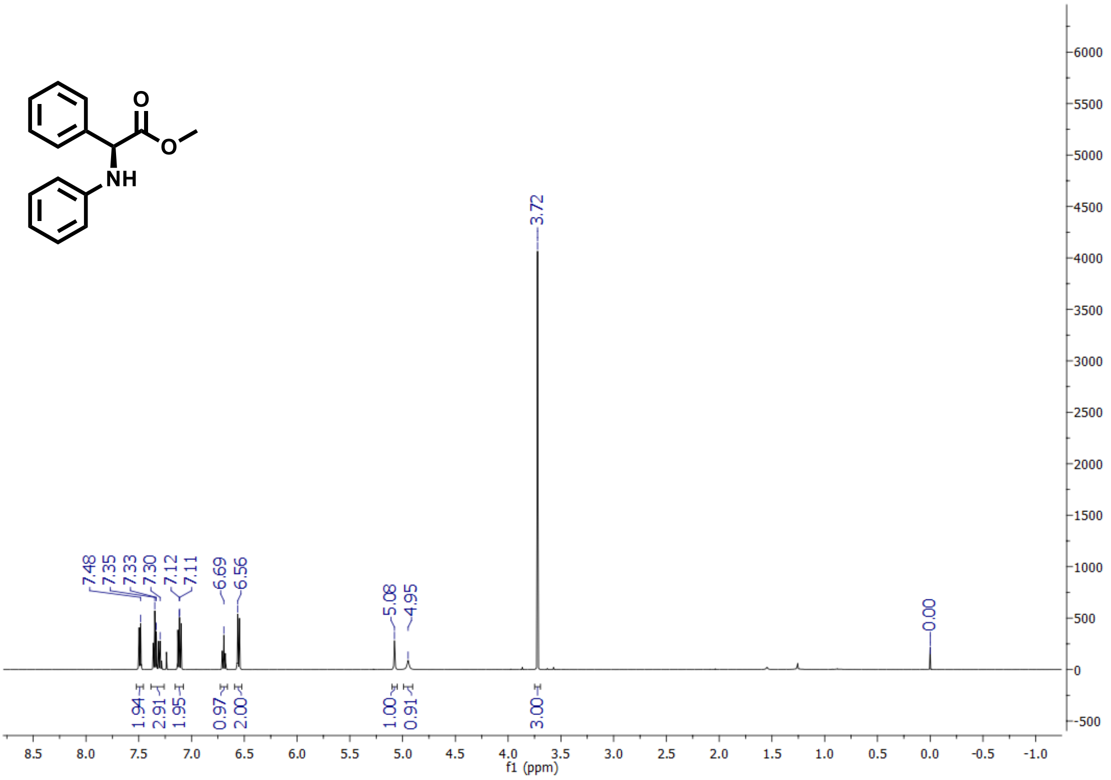

(S)-Methyl 2-phenyl-2-(phenylamino)acetate (**3**) ^{13}C NMR (101 MHz, $CDCl_3$)

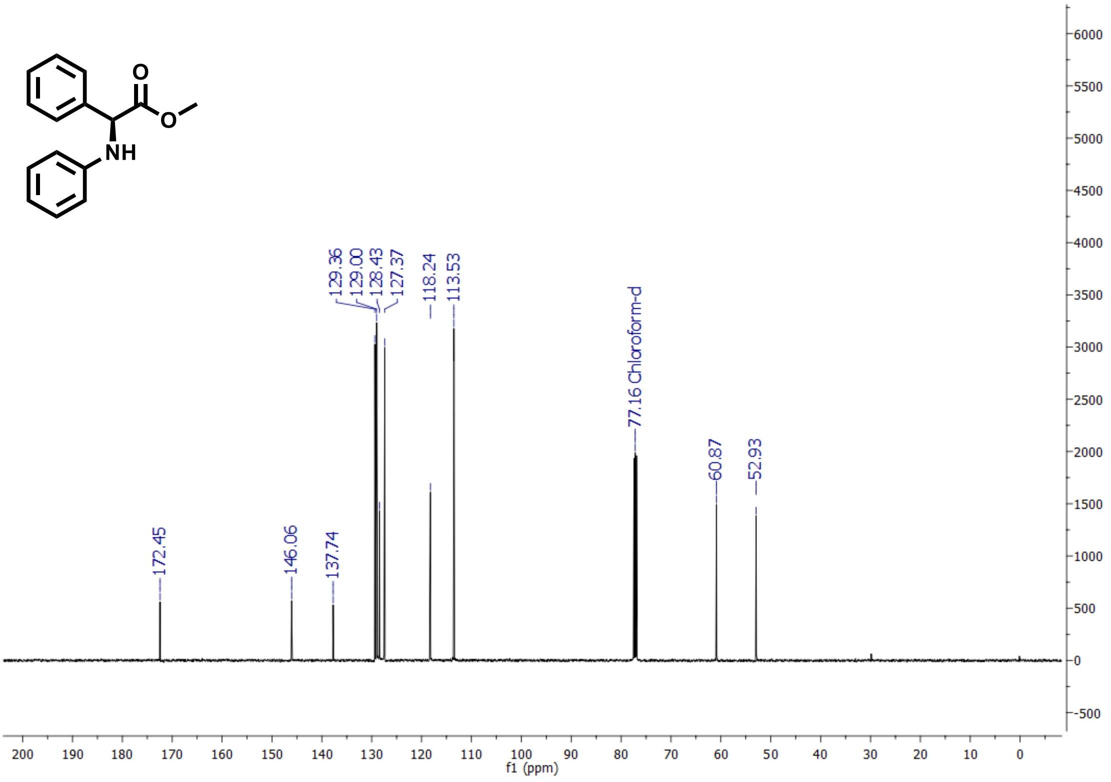

Phenyl 2-phenyl-2-(phenylamino)acetate (**4**) ^{1}H NMR (400 MHz, $CDCl_3$)

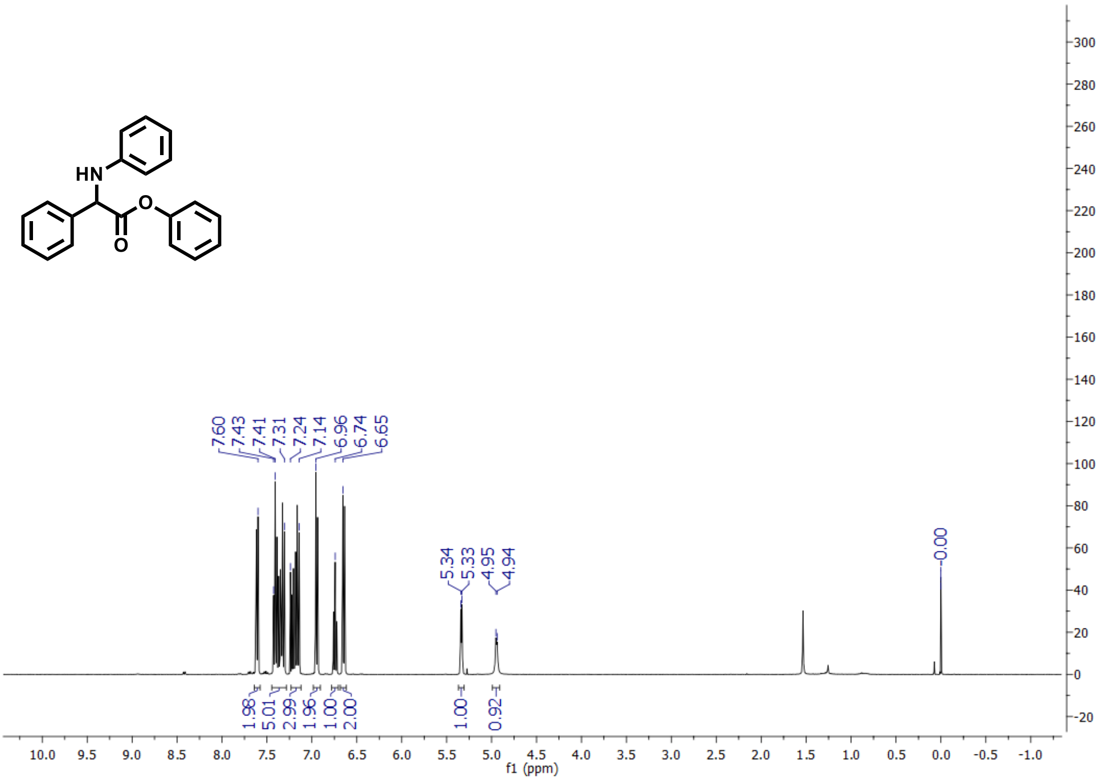

Phenyl 2-phenyl-2-(phenylamino)acetate (**4**) ^{13}C NMR (126 MHz, $CDCl_3$)

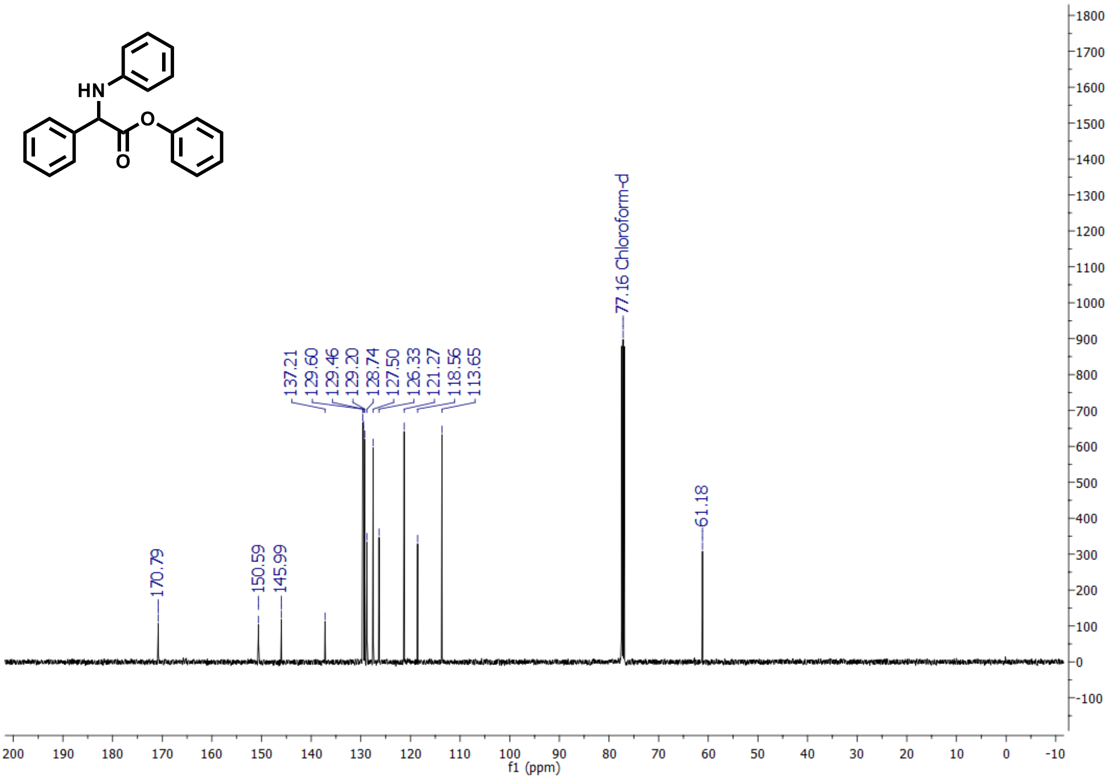

Benzyl 2-phenyl-2-(phenylamino)acetate (**5**) ^{1}H NMR (500 MHz, $CDCl_3$)

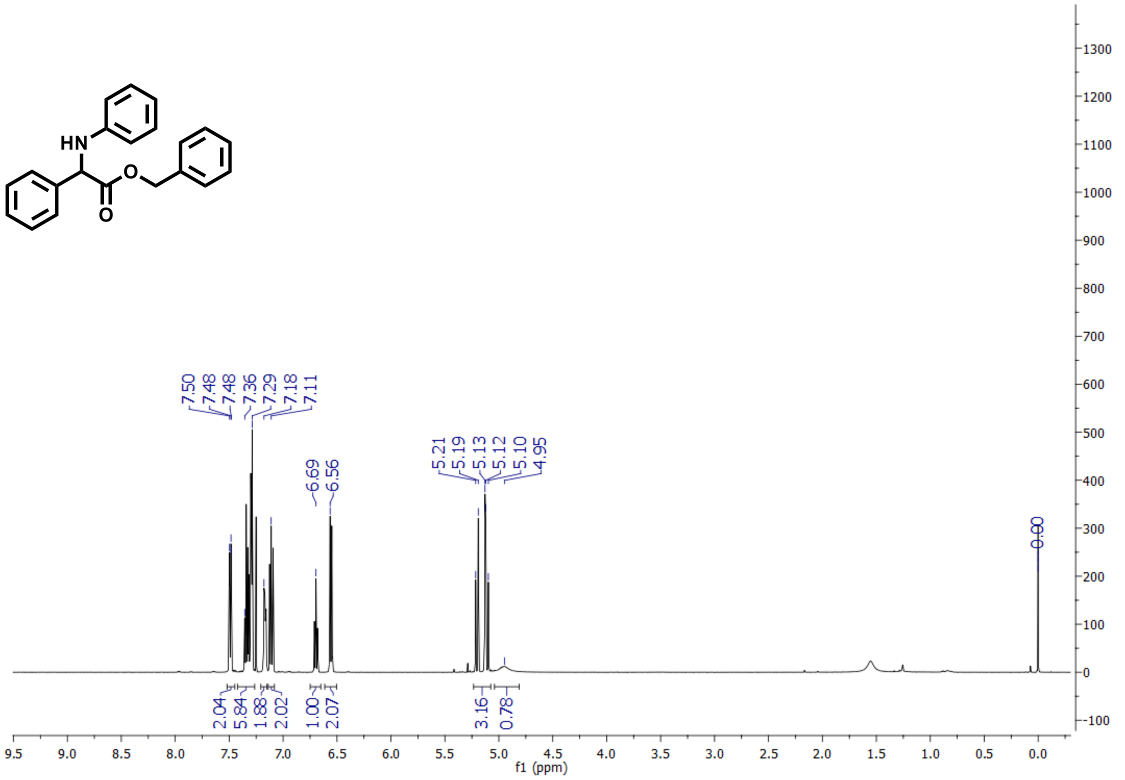

Benzyl 2-phenyl-2-(phenylamino)acetate (**5**) ^{13}C NMR (126 MHz, $CDCl_3$)

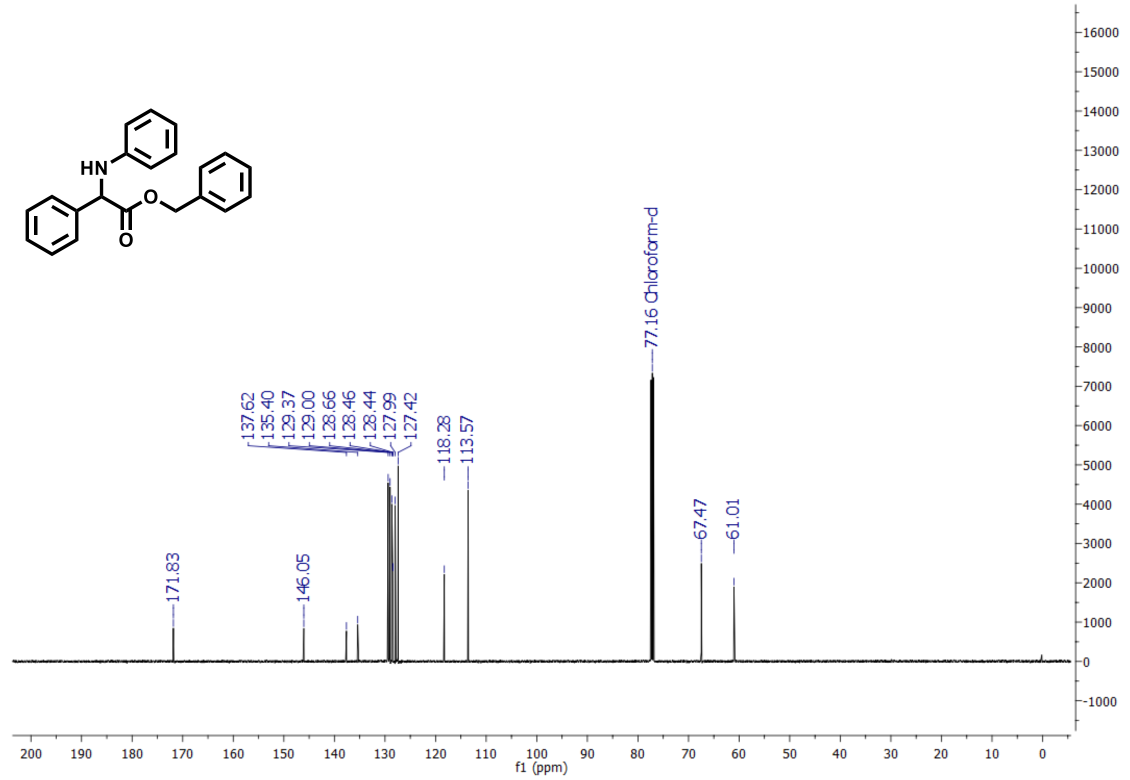

Tert-butyl 2-phenyl-2-(phenylamino)acetate (**6**) ^{1}H NMR (400 MHz, $CDCl_3$)

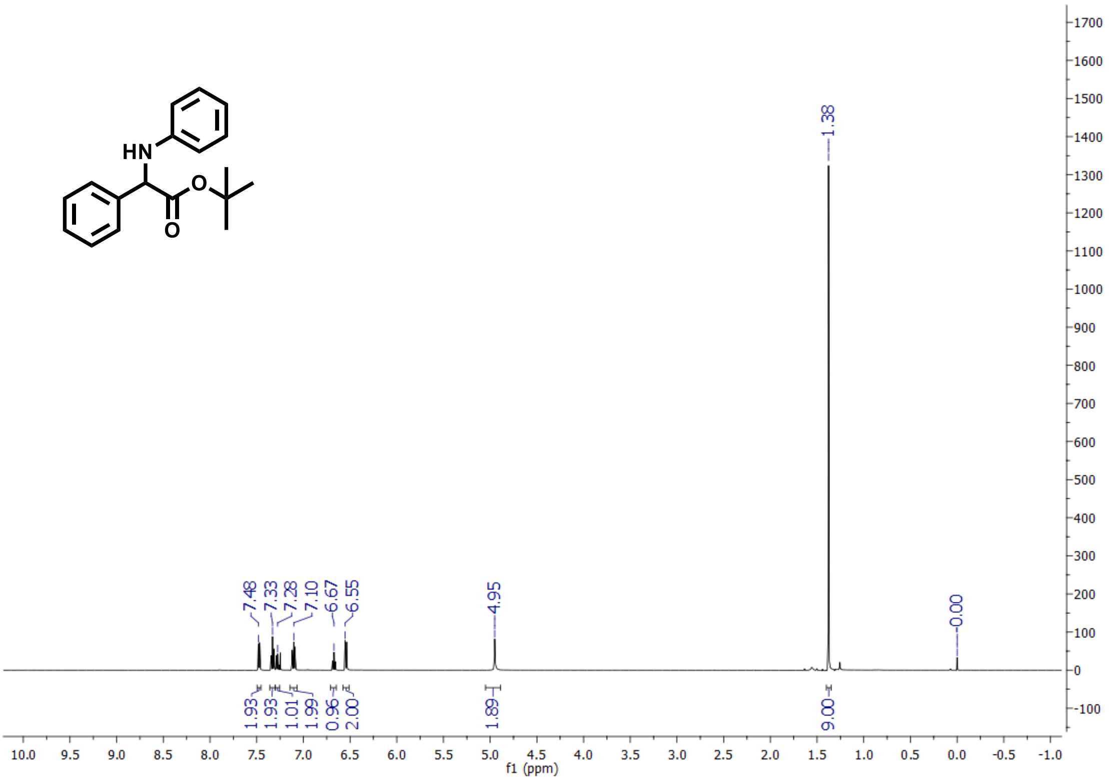

Tert-butyl 2-phenyl-2-(phenylamino)acetate (**6**) ^{1}H NMR (400 MHz, $CDCl_3$)

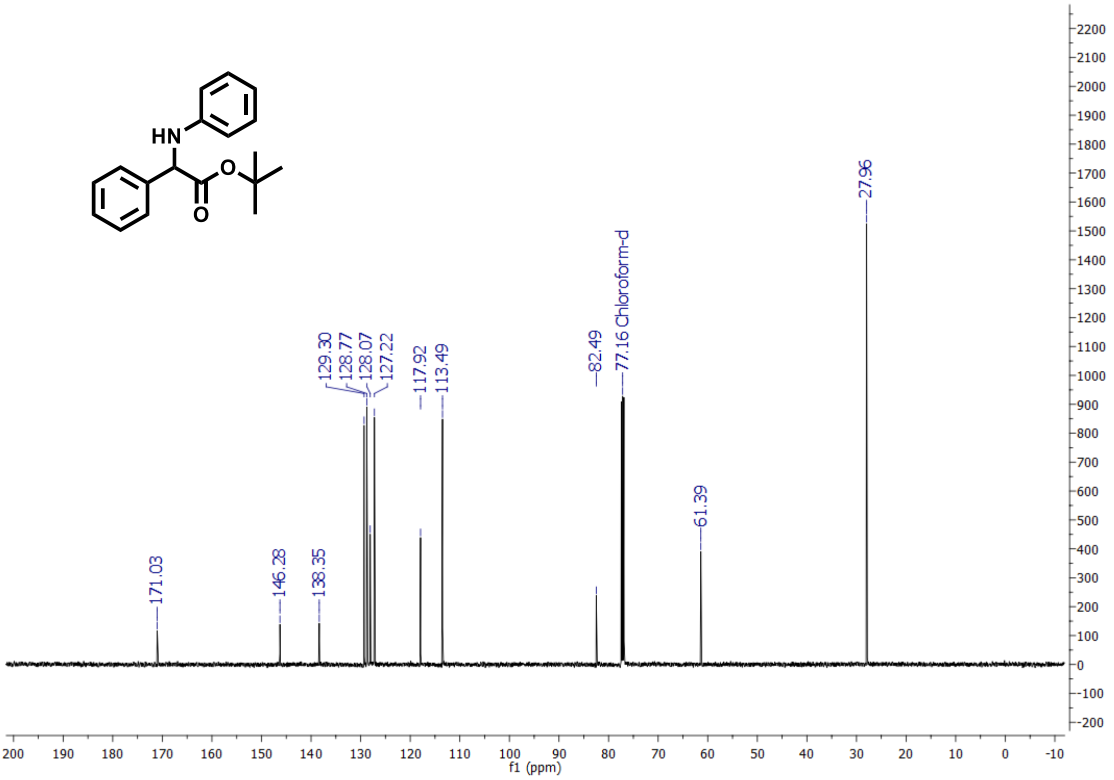

Methyl 2-((4-methoxyphenyl)amino)-2-phenylacetate (**7**) ^{1}H NMR (400 MHz, $CDCl_3$)

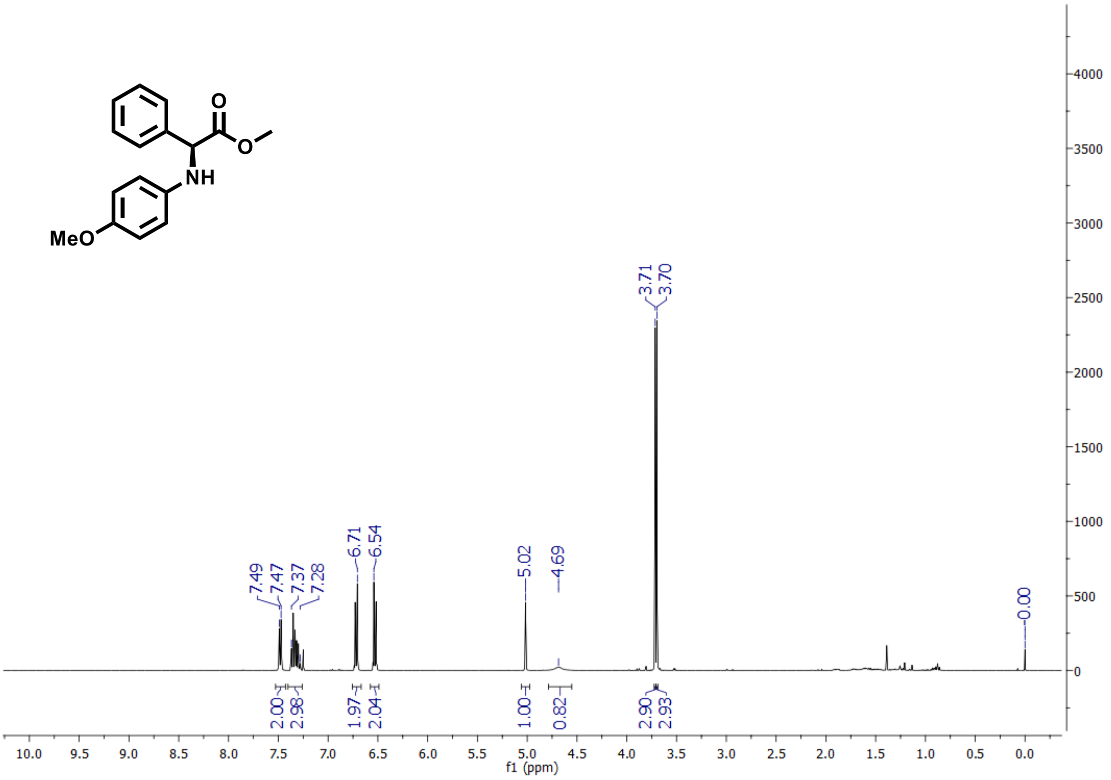

Methyl 2-((4-methoxyphenyl)amino)-2-phenylacetate (**7**) ^{13}C NMR (126 MHz, $CDCl_3$)

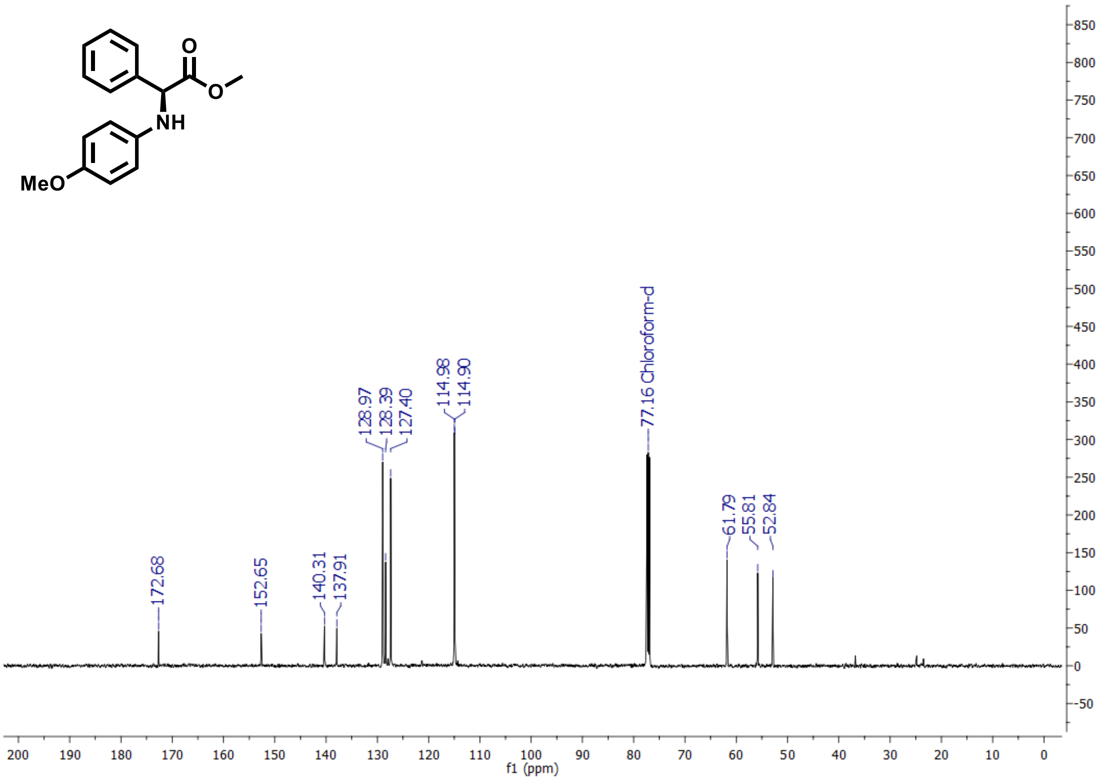

Methyl 2-((4-nitrophenyl)amino)-2-phenylacetate (**8**) ^{1}H NMR (400 MHz, $CDCl_3$)

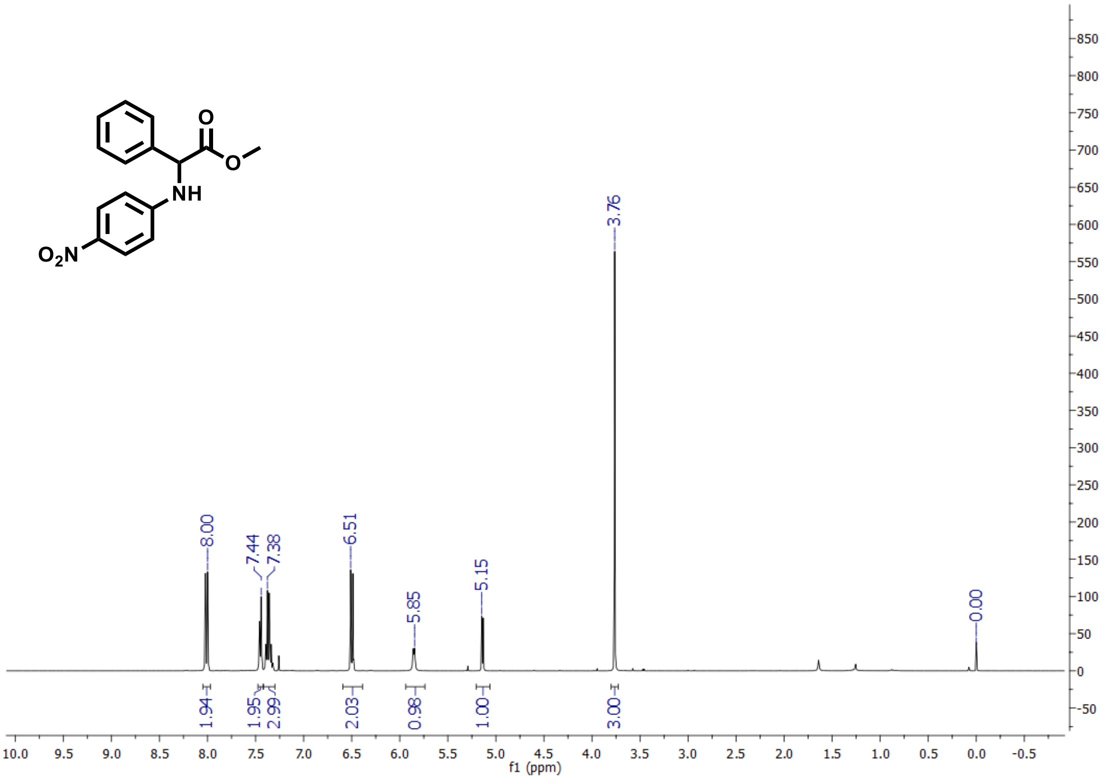

Methyl 2-((4-nitrophenyl)amino)-2-phenylacetate (**8**) ^{13}C NMR (126 MHz, $CDCl_3$)

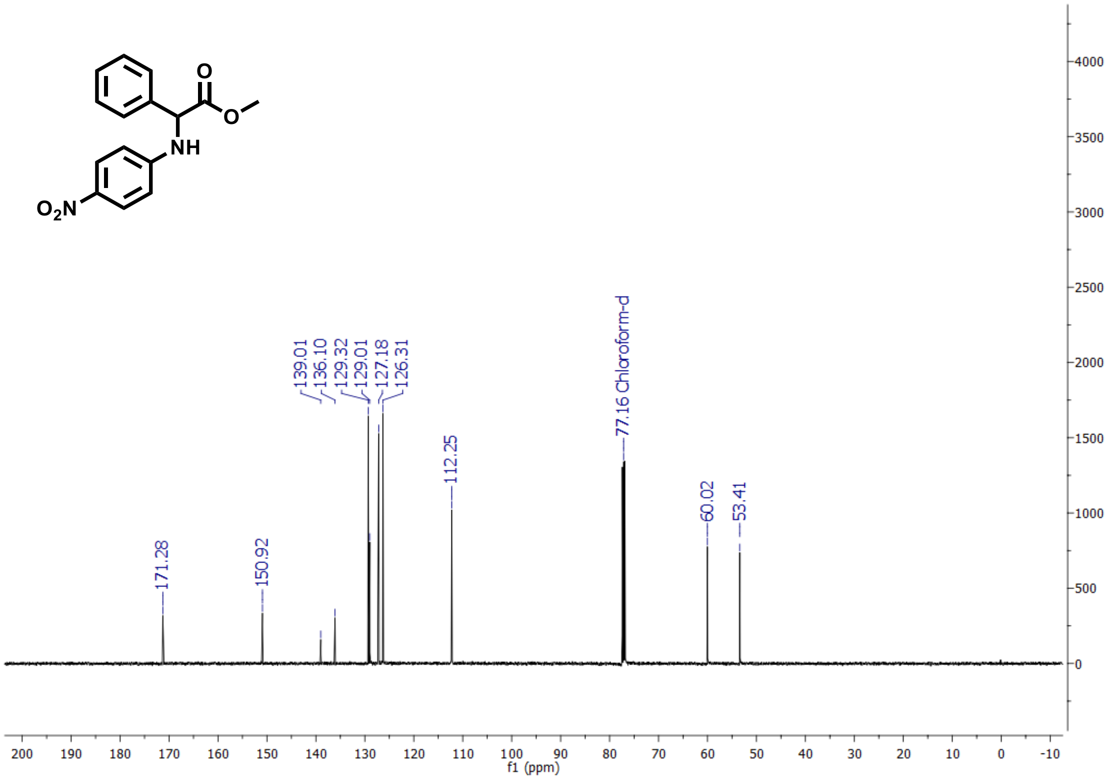

Methyl 2-phenyl-2-(p-tolylamino)acetate (**9**) ^{1}H NMR (400 MHz, CDCl$_3$)

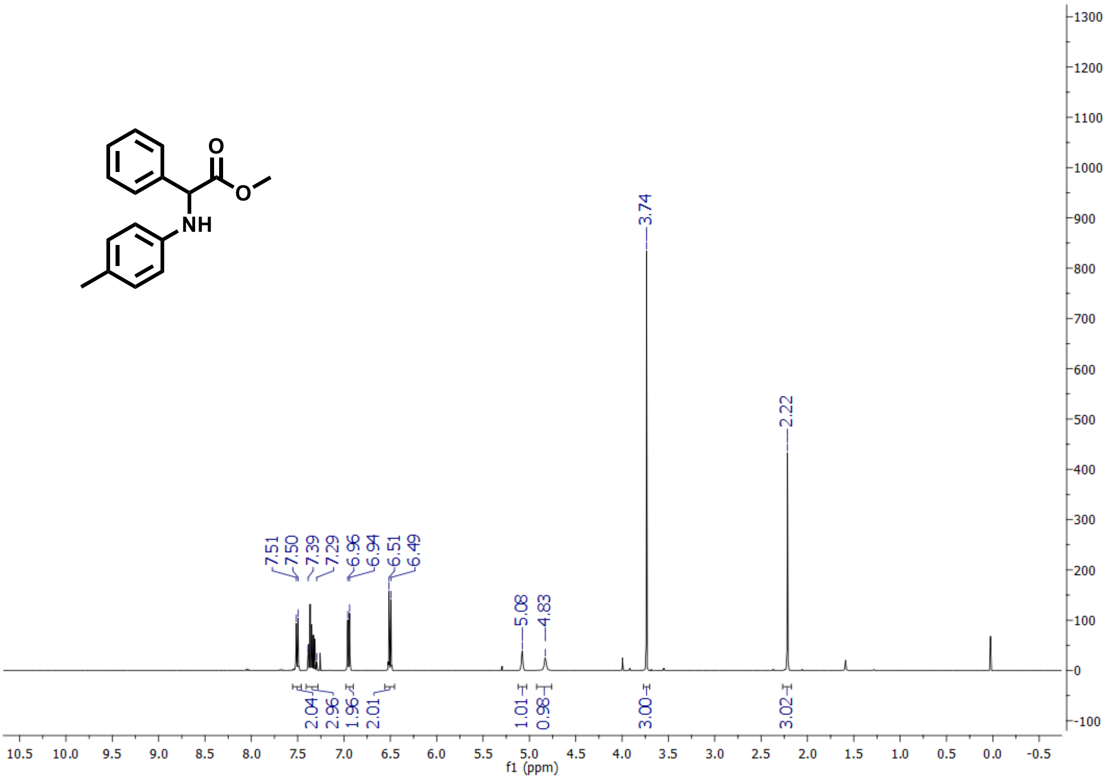

Methyl 2-phenyl-2-(p-tolylamino)acetate (**9**) ^{13}C NMR (101 MHz, CDCl$_3$)

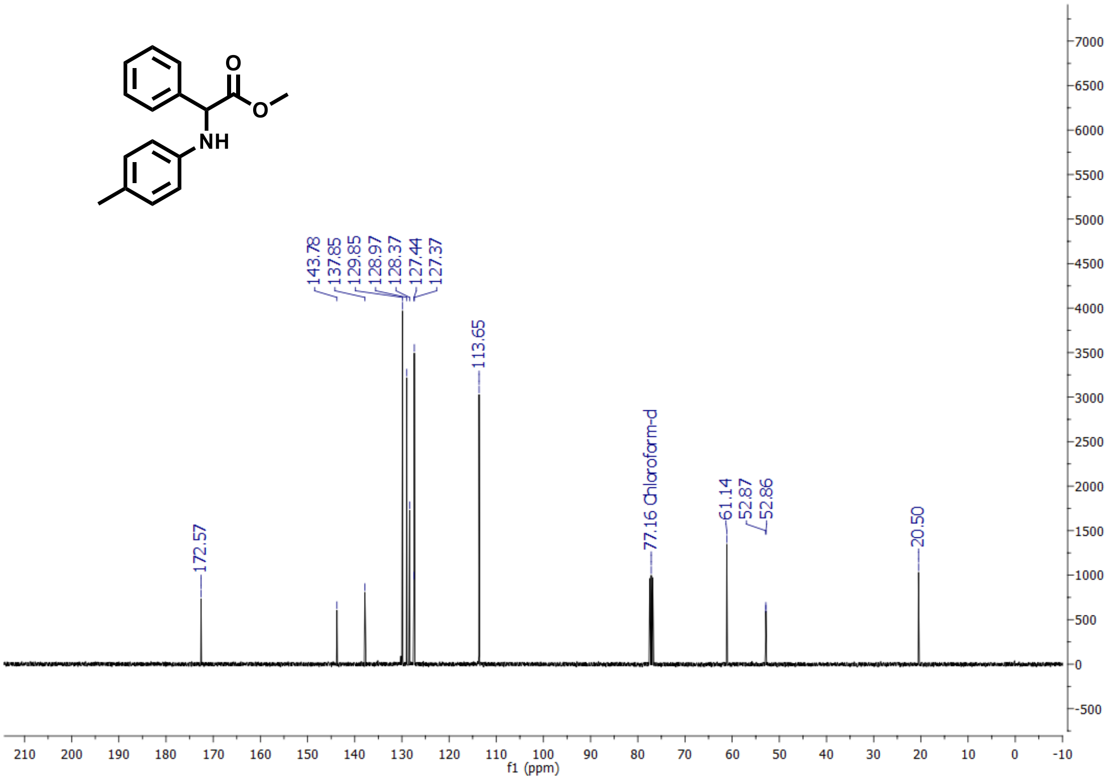

Methyl 2-((4-bromophenyl)amino)-2-phenylacetate (**10**) ^{1}H NMR (400 MHz, $CDCl_3$)

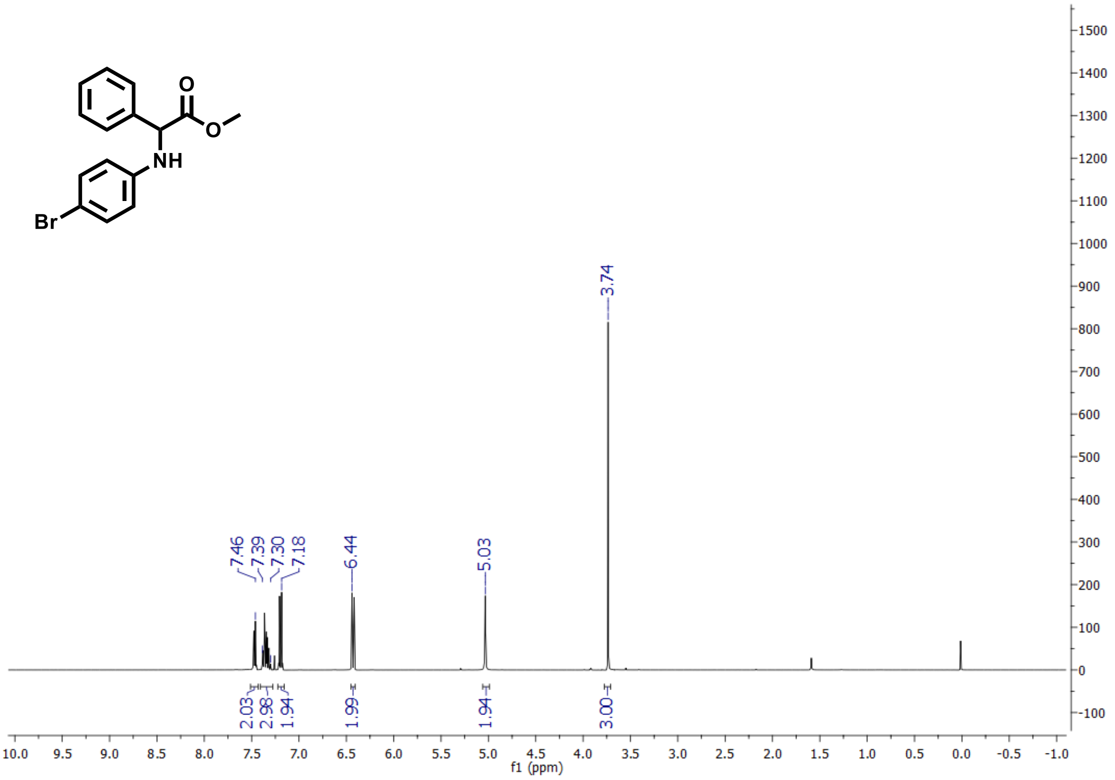

,

Methyl 2-((4-bromophenyl)amino)-2-phenylacetate (**10**) ^{13}C NMR (101 MHz, $CDCl_3$)

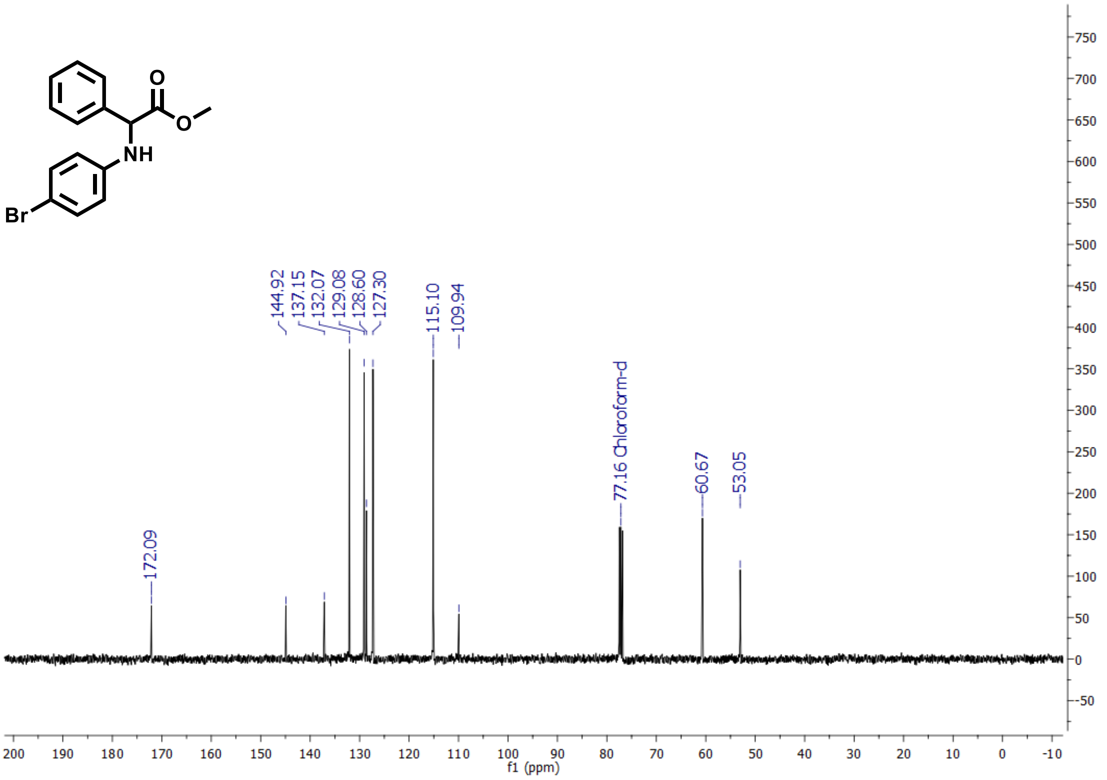

Methyl 2-((4-chlorophenyl)amino)-2-phenylacetate (**11**) ^{1}H NMR (400 MHz, $CDCl_3$)

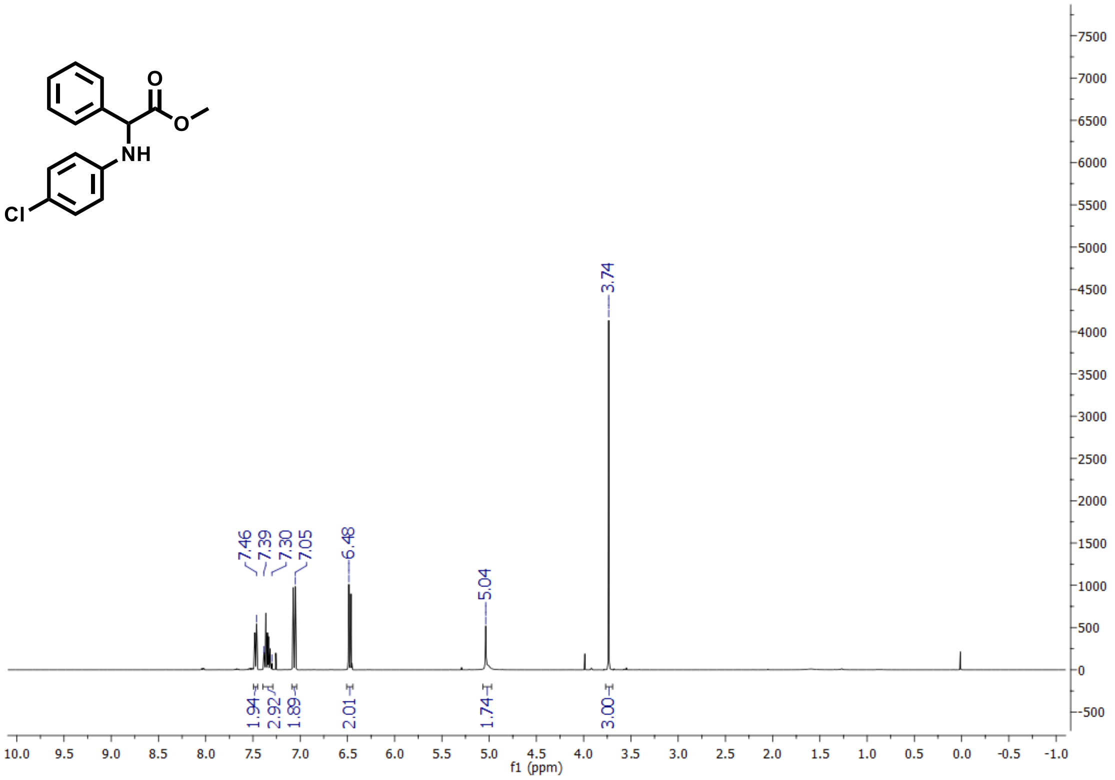

Methyl 2-((4-chlorophenyl)amino)-2-phenylacetate (**11**) ^{13}C NMR (101 MHz, $CDCl_3$)

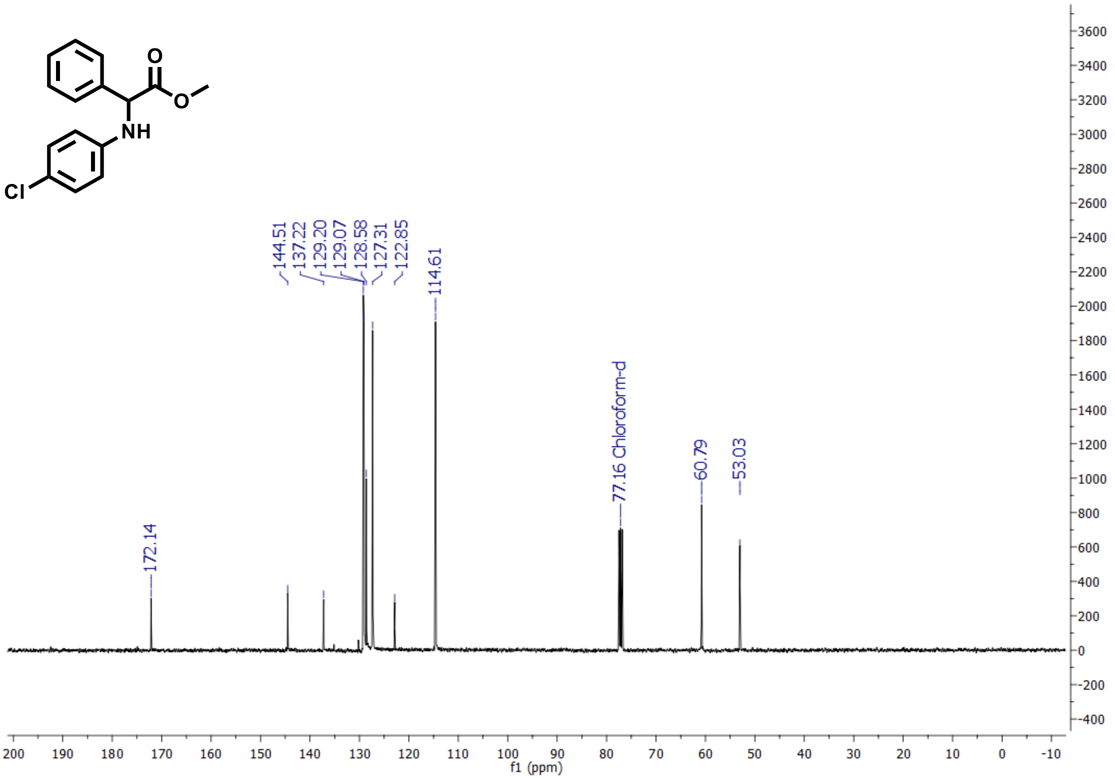

Methyl 2-((2-methoxyphenyl)amino)-2-phenylacetate (**12**) ^{1}H NMR (400 MHz, $CDCl_3$)

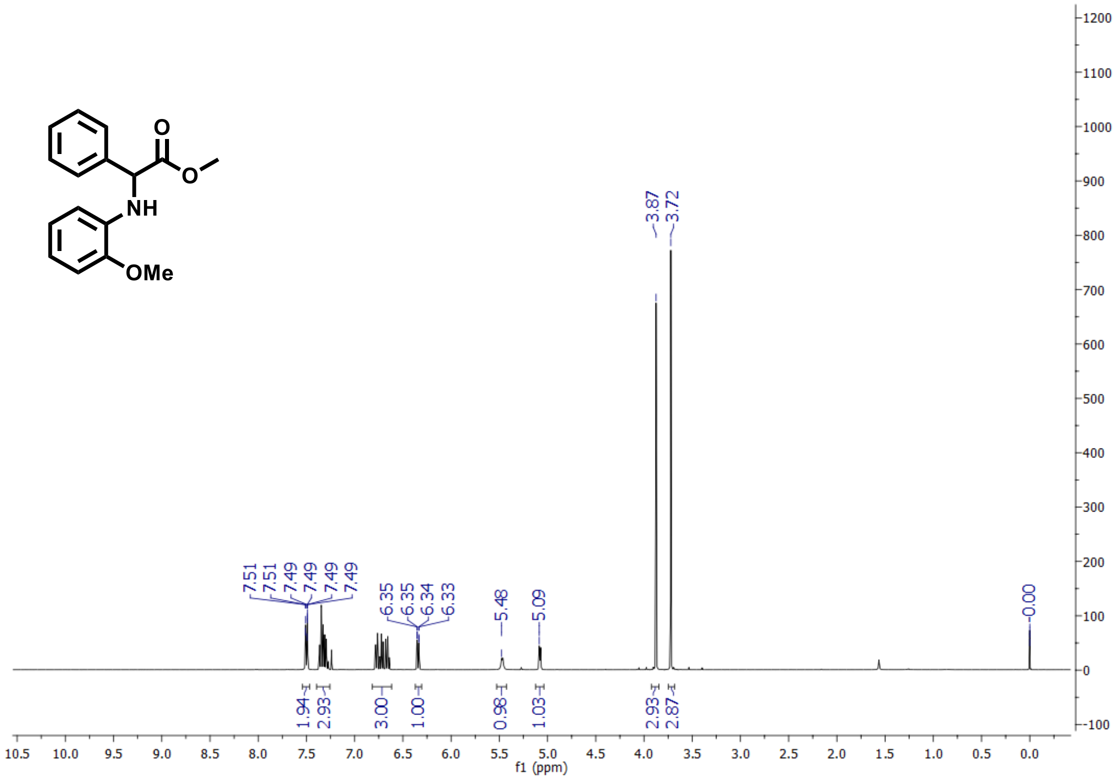

Methyl 2-((2-methoxyphenyl)amino)-2-phenylacetate (**12**) ^{13}C NMR (101 MHz, $CDCl_3$)

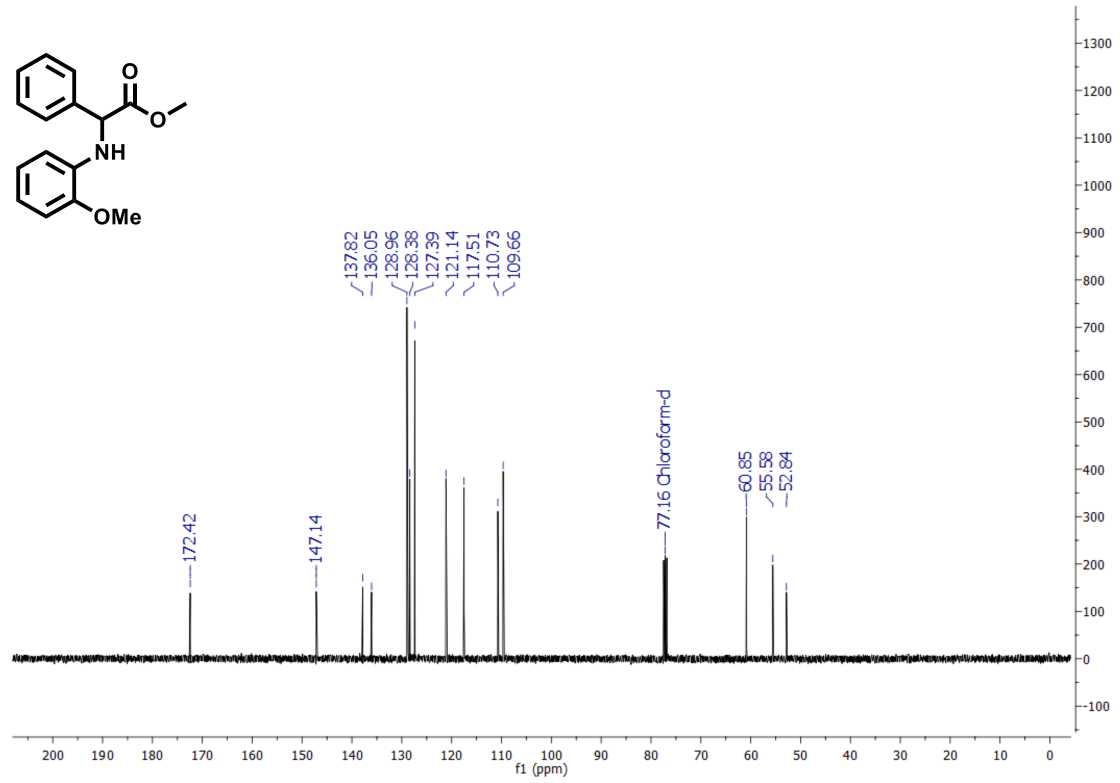

Methyl 2-phenyl-2-(o-tolylamino)acetate (**13**) ^{1}H NMR (400 MHz, $CDCl_3$)

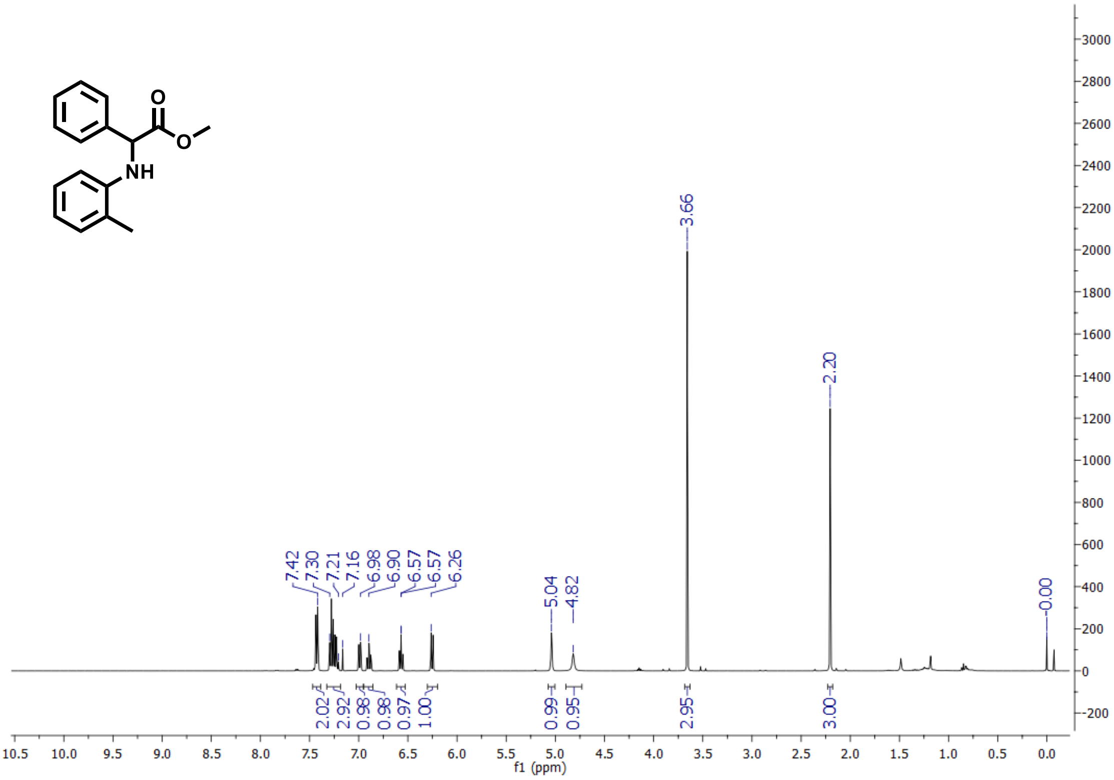

Methyl 2-phenyl-2-(o-tolylamino)acetate (**13**) ^{13}C NMR (126 MHz, $CDCl_3$)

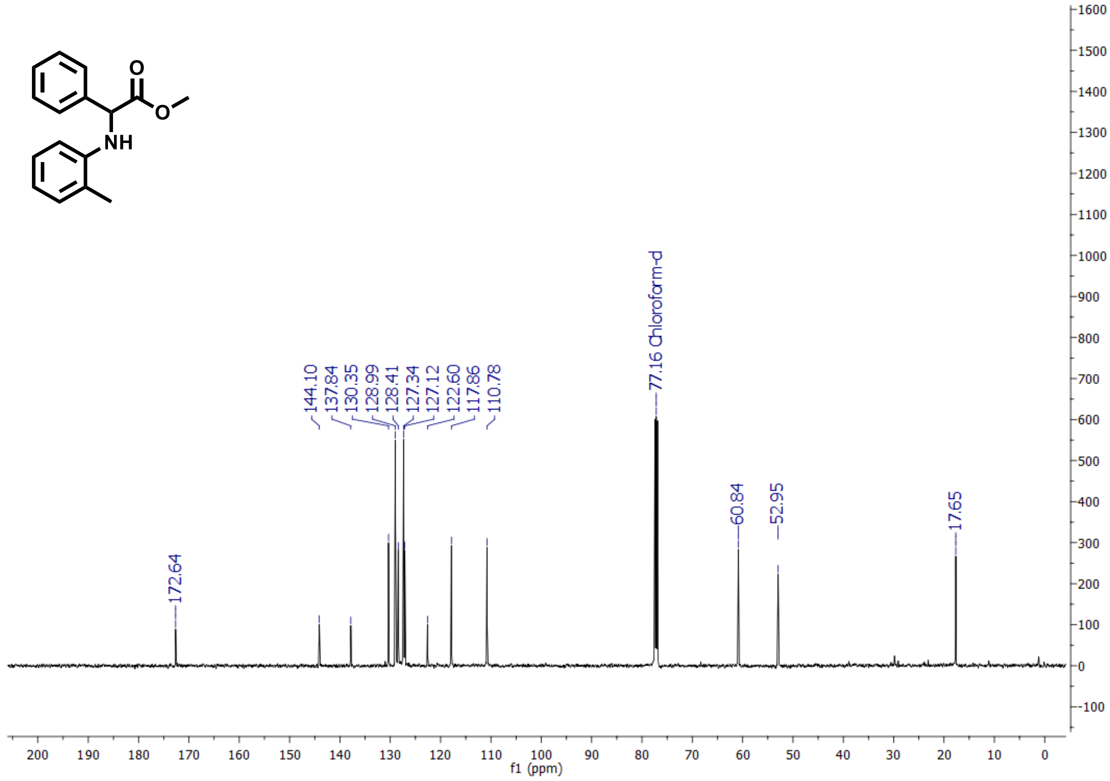

Methyl 2-((3-methoxyphenyl)amino)-2-phenylacetate (**14**) ^{1}H NMR (500 MHz, $CDCl_3$)

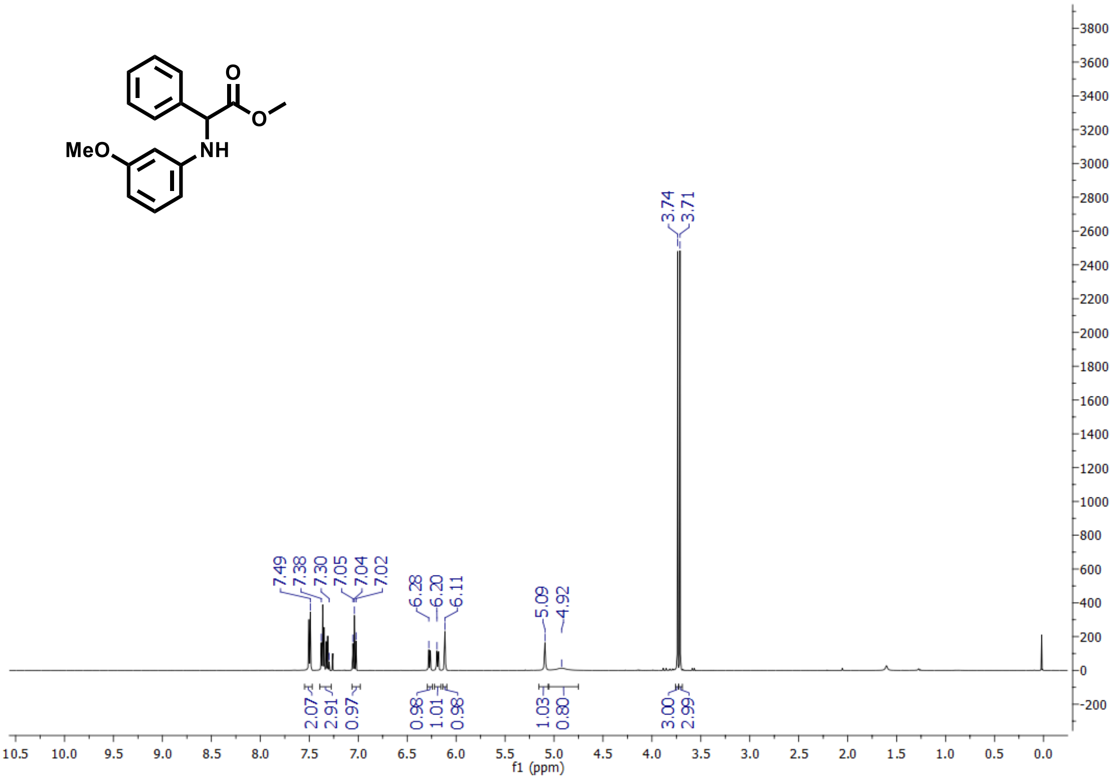

Methyl 2-((3-methoxyphenyl)amino)-2-phenylacetate (**14**) ^{13}C NMR (101 MHz, $CDCl_3$)

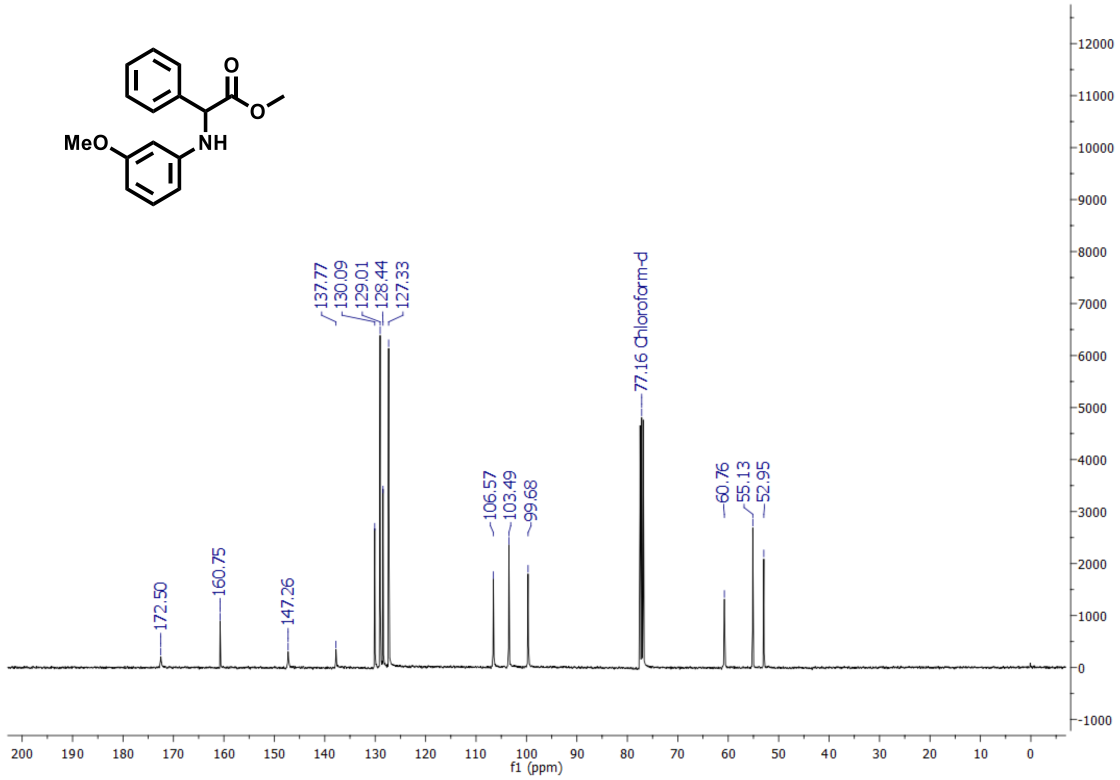

Methyl 2-phenyl-2-(m-tolylamino)acetate (**15**) ^{1}H NMR (500 MHz, $CDCl_3$)

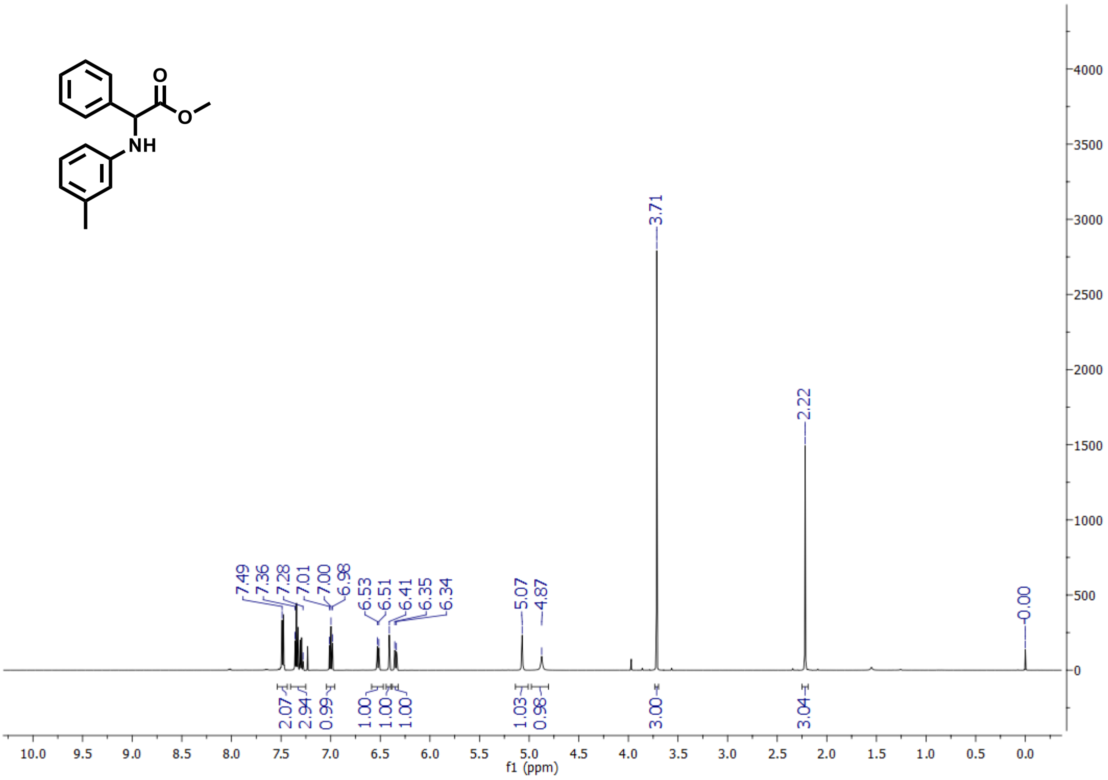

Methyl 2-phenyl-2-(m-tolylamino)acetate (**15**) ^{13}C NMR (126 MHz, $CDCl_3$)

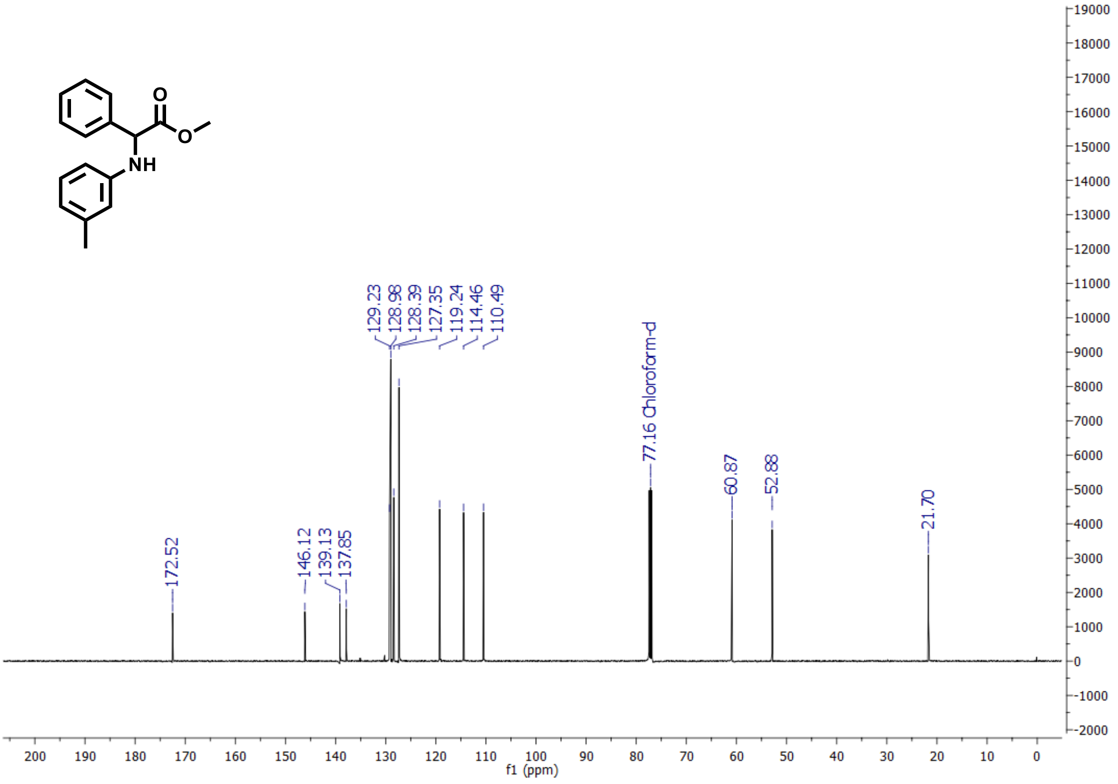

Methyl 2-((3-bromophenyl)amino)-2-phenylacetate (**16**) ^{1}H NMR (500 MHz, $CDCl_3$)

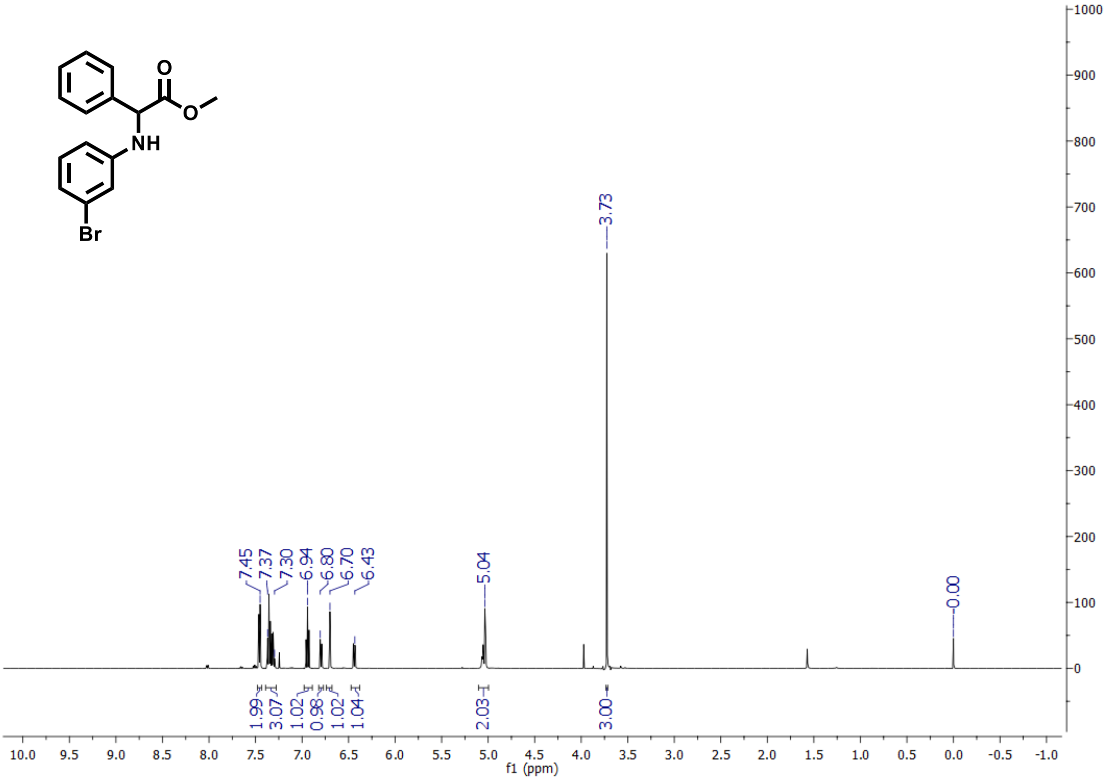

Methyl 2-((3-bromophenyl)amino)-2-phenylacetate (**16**) ^{13}C NMR (126 MHz, $CDCl_3$)

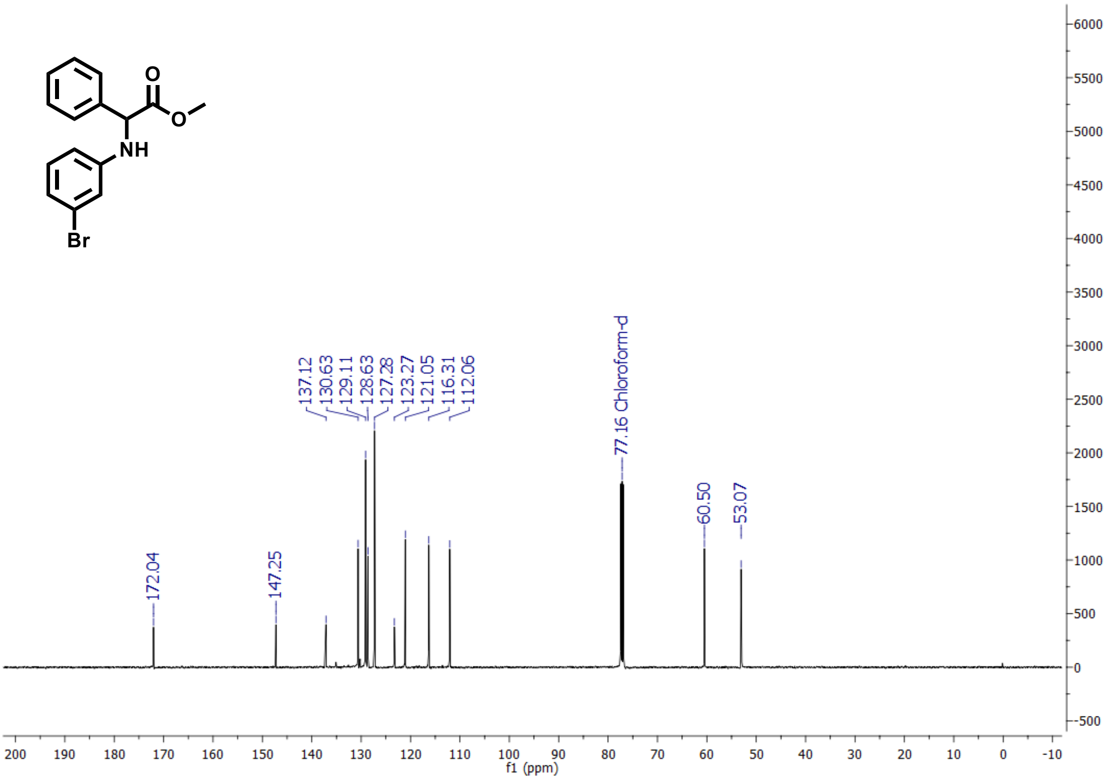

Methyl 2-phenyl-2-((3-(trifluoromethyl)phenyl)amino)acetate (**17**) ^{1}H NMR (500 MHz, $CDCl_3$)

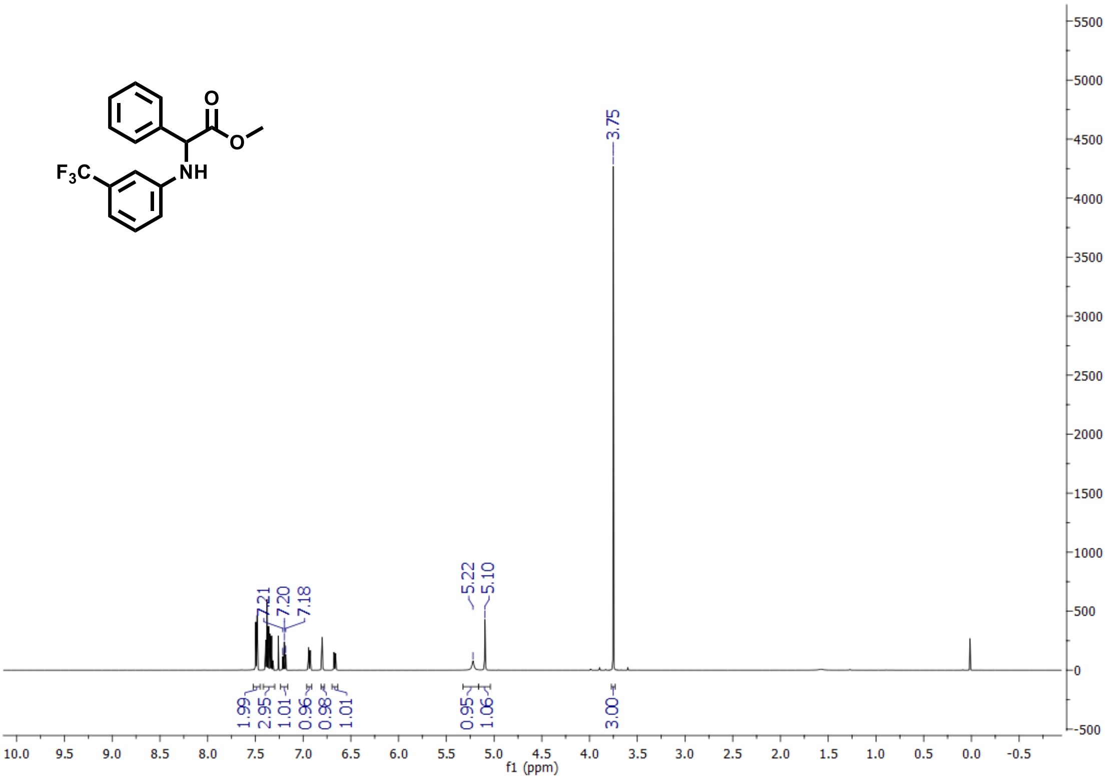

Methyl 2-phenyl-2-((3-(trifluoromethyl)phenyl)amino)acetate (**17**) ^{13}C NMR (101 MHz, $CDCl_3$)

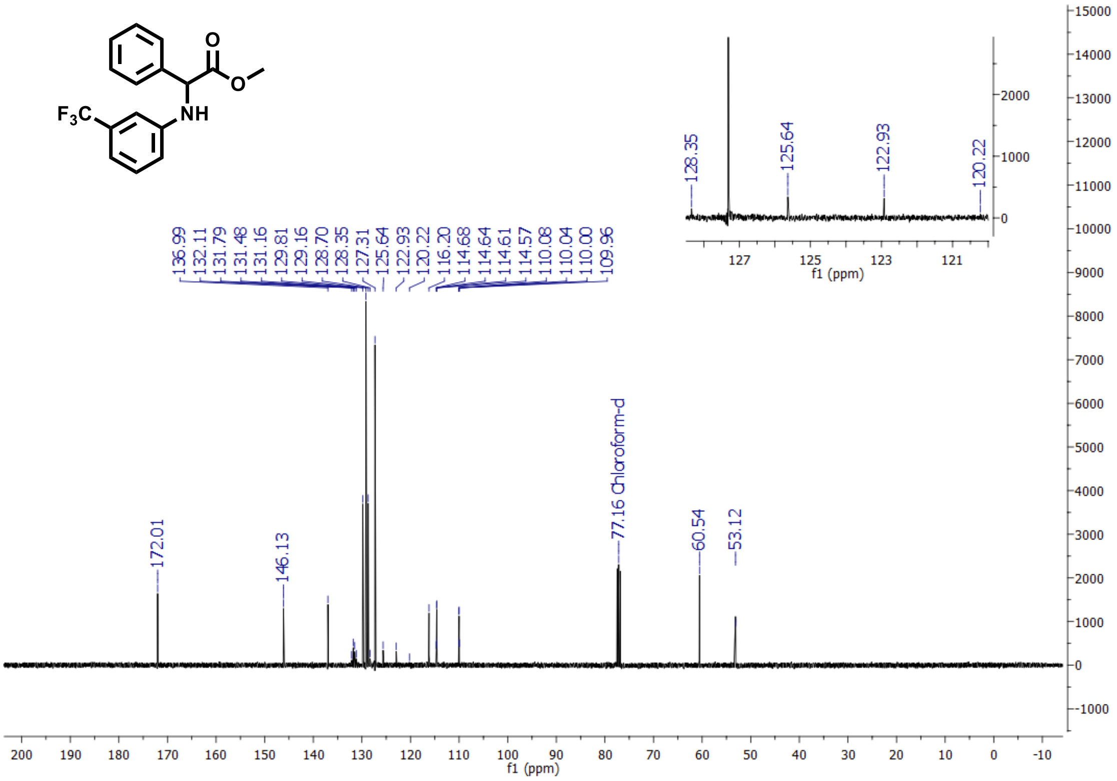

Methyl 2-((3-chlorophenyl)amino)-2-phenylacetate (**18**) ^{1}H NMR (400 MHz, $CDCl_3$)

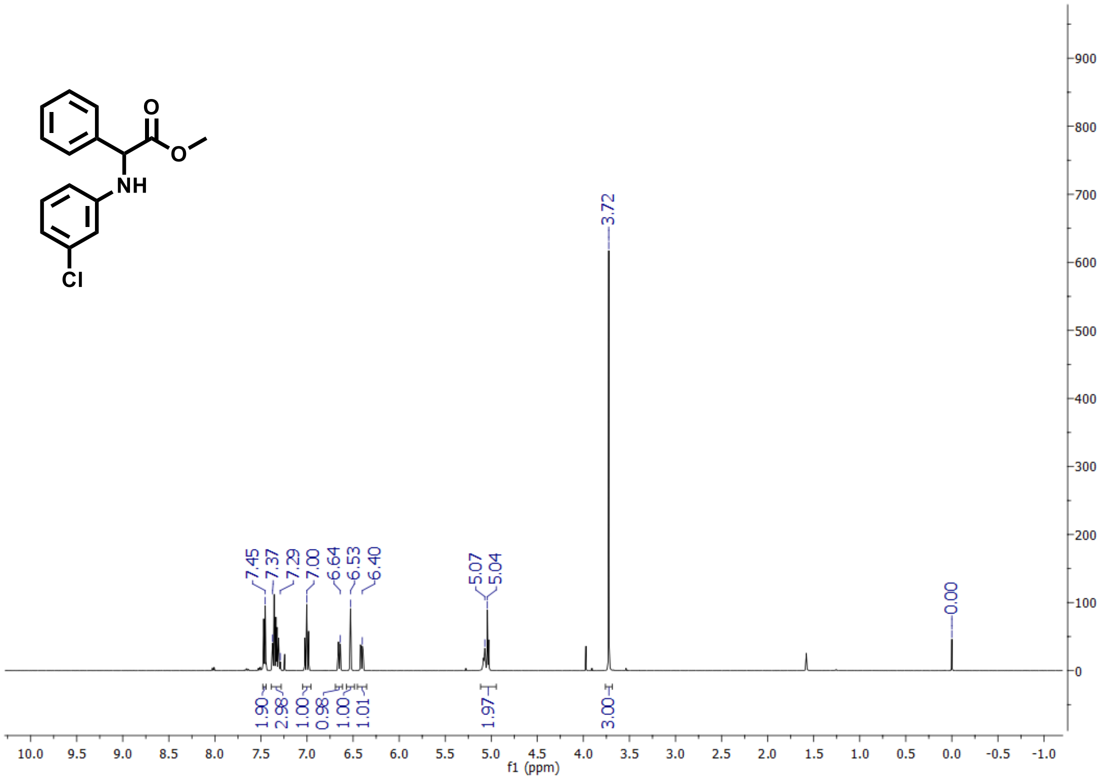

Methyl 2-((3-chlorophenyl)amino)-2-phenylacetate (**18**) ^{13}C NMR (101 MHz, $CDCl_3$)

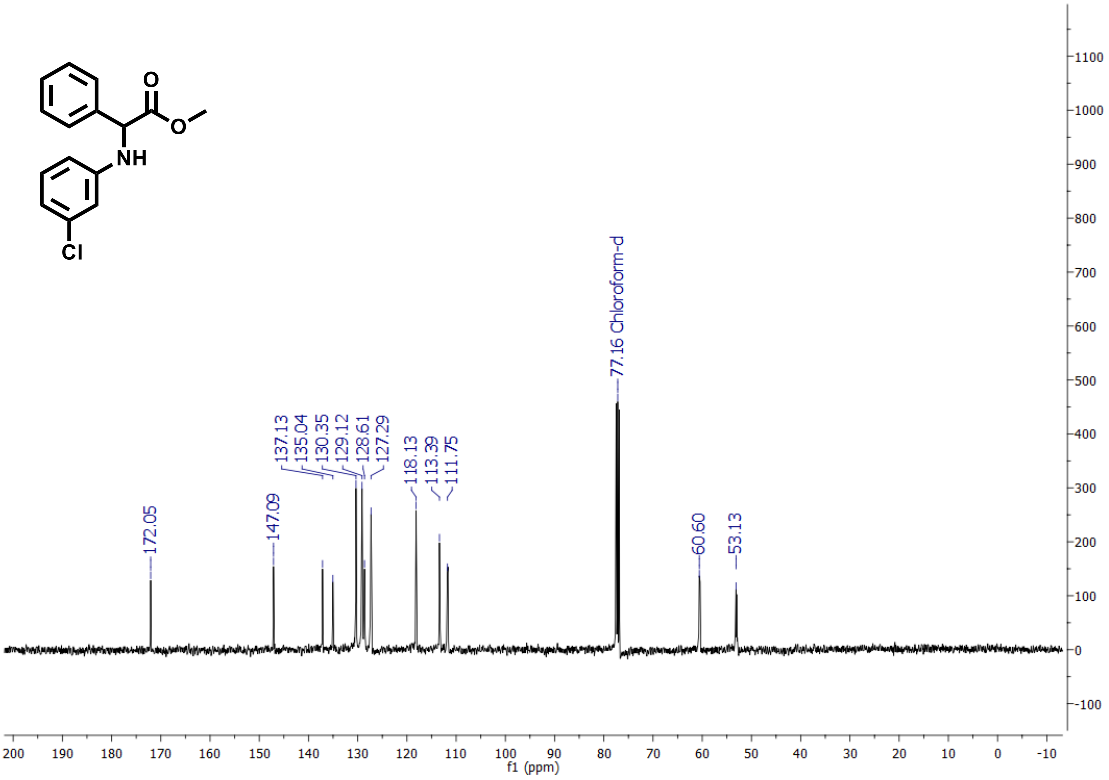

Methyl 2-(naphthalen-1-ylamino)-2-phenylacetate (**19**) ^{1}H NMR (400 MHz, $CDCl_3$)

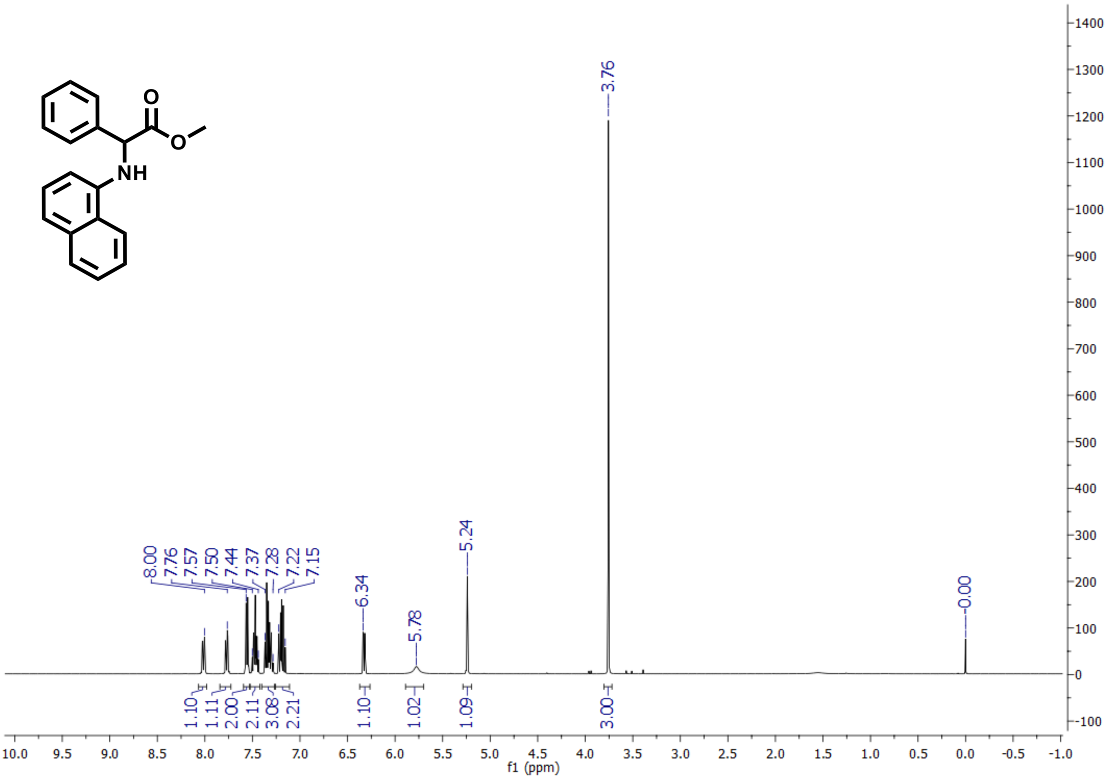

Methyl 2-(naphthalen-1-ylamino)-2-phenylacetate (**19**) ^{13}C NMR (101 MHz, $CDCl_3$)

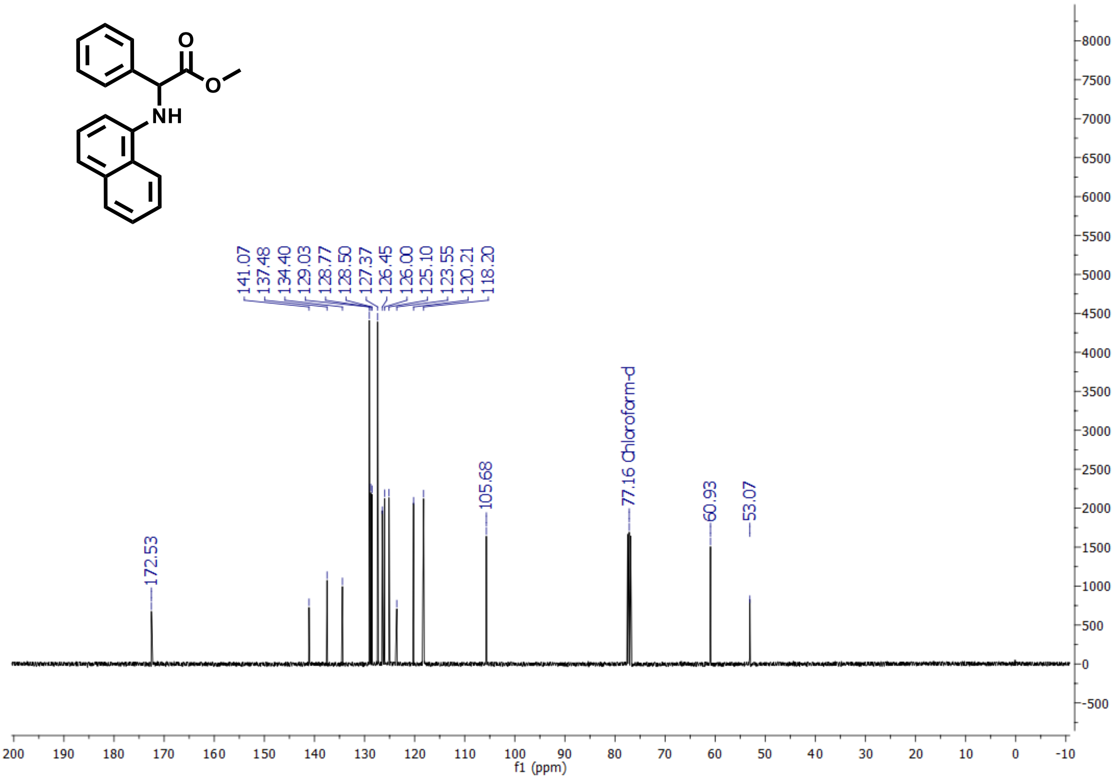

Methyl 2-((3-chloro-2-methylphenyl)amino)-2-phenylacetate (**20**) ^{1}H NMR (400 MHz, $CDCl_3$)

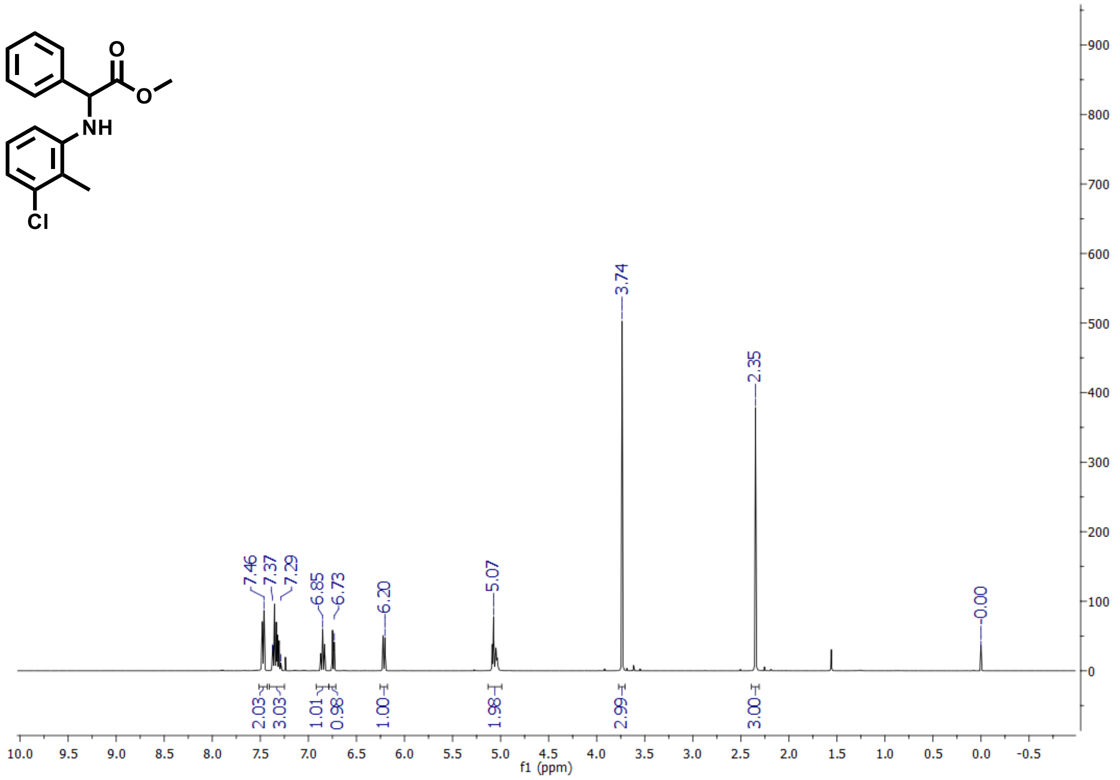

Methyl 2-((3-chloro-2-methylphenyl)amino)-2-phenylacetate (**20**) ^{13}C NMR (101 MHz, $CDCl_3$)

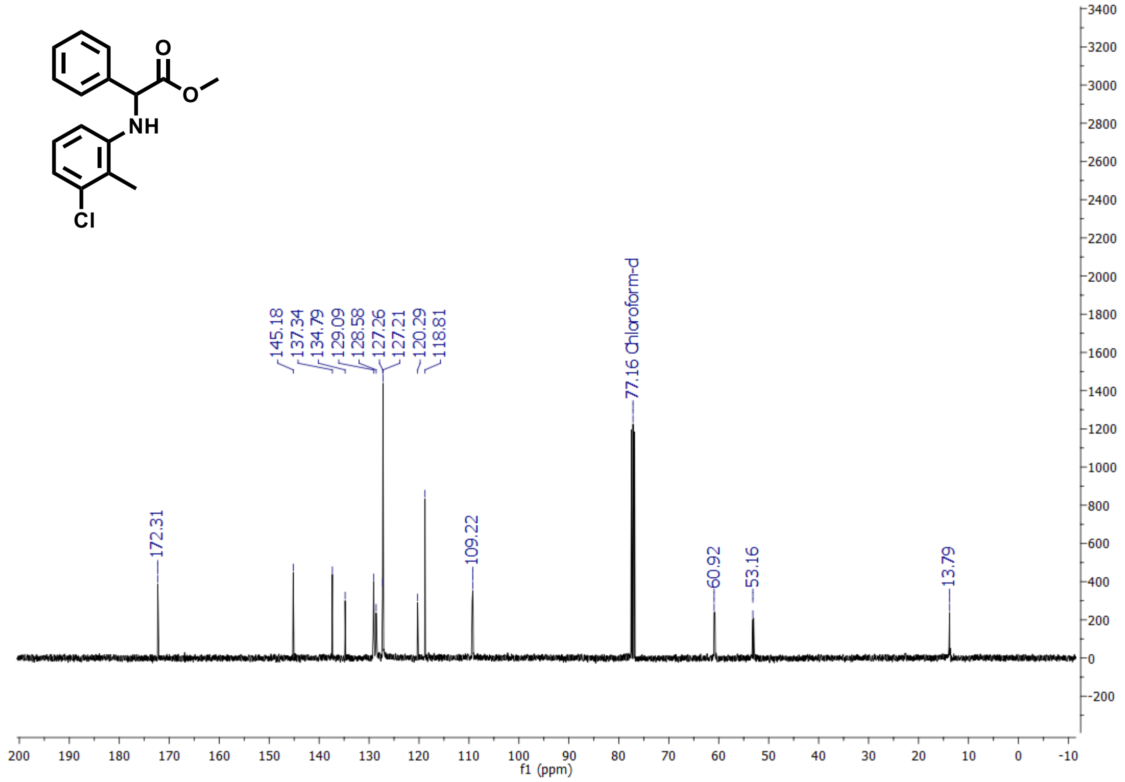

Methyl 2-(benzo[d][1,3]dioxol-5-ylamino)-2-phenylacetate (**21**) ^{1}H NMR (400 MHz, $CDCl_3$)

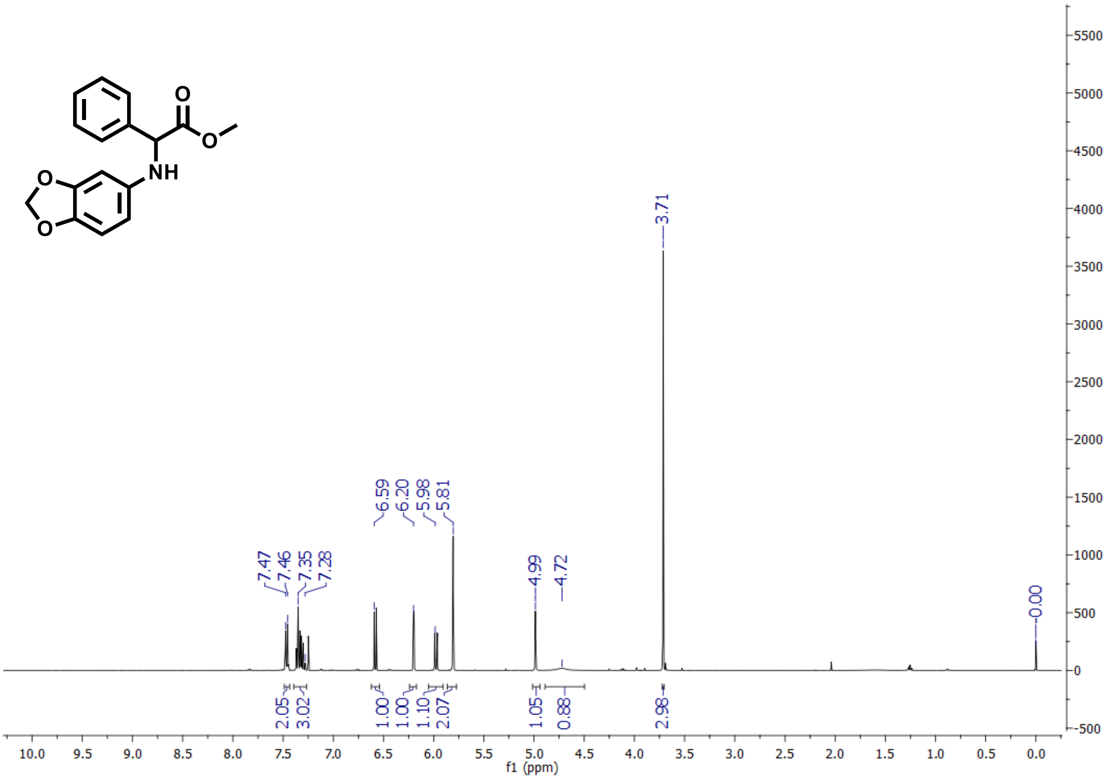

Methyl 2-(benzo[d][1,3]dioxol-5-ylamino)-2-phenylacetate (**21**) ^{13}C NMR (126 MHz, $CDCl_3$)

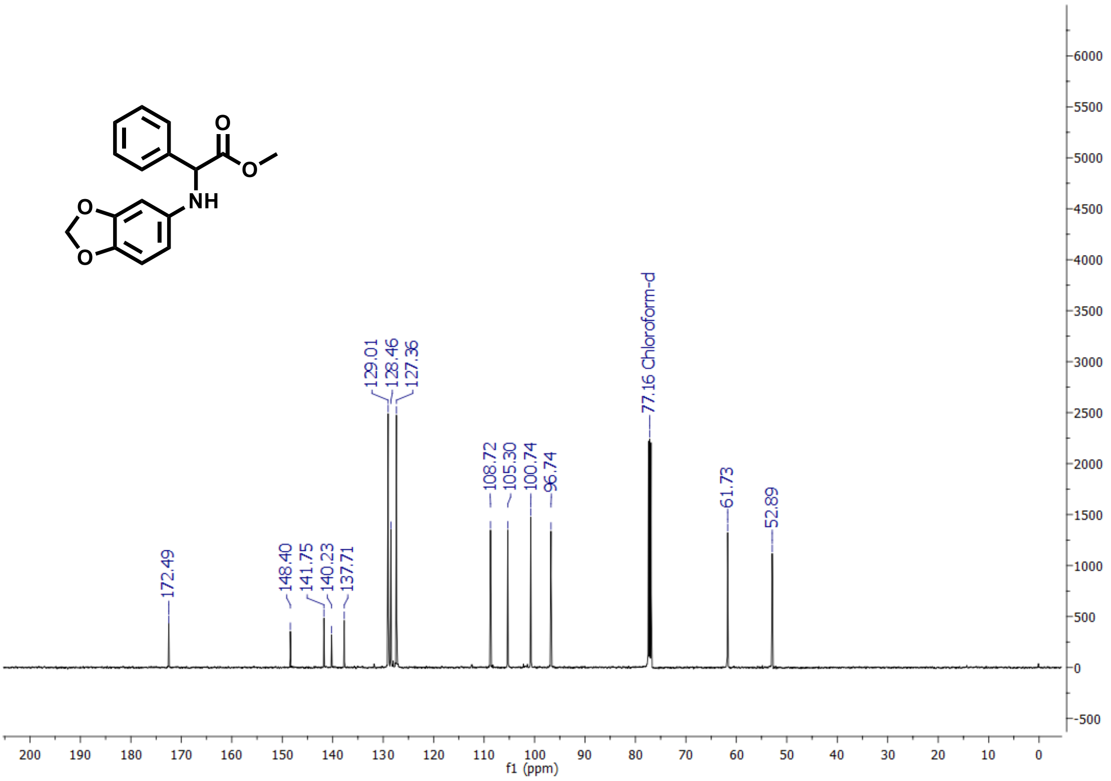

Methyl 2-((3,5-dimethoxyphenyl)amino)-2-phenylacetate (**22**) ^{1}H NMR (400 MHz, $CDCl_3$)

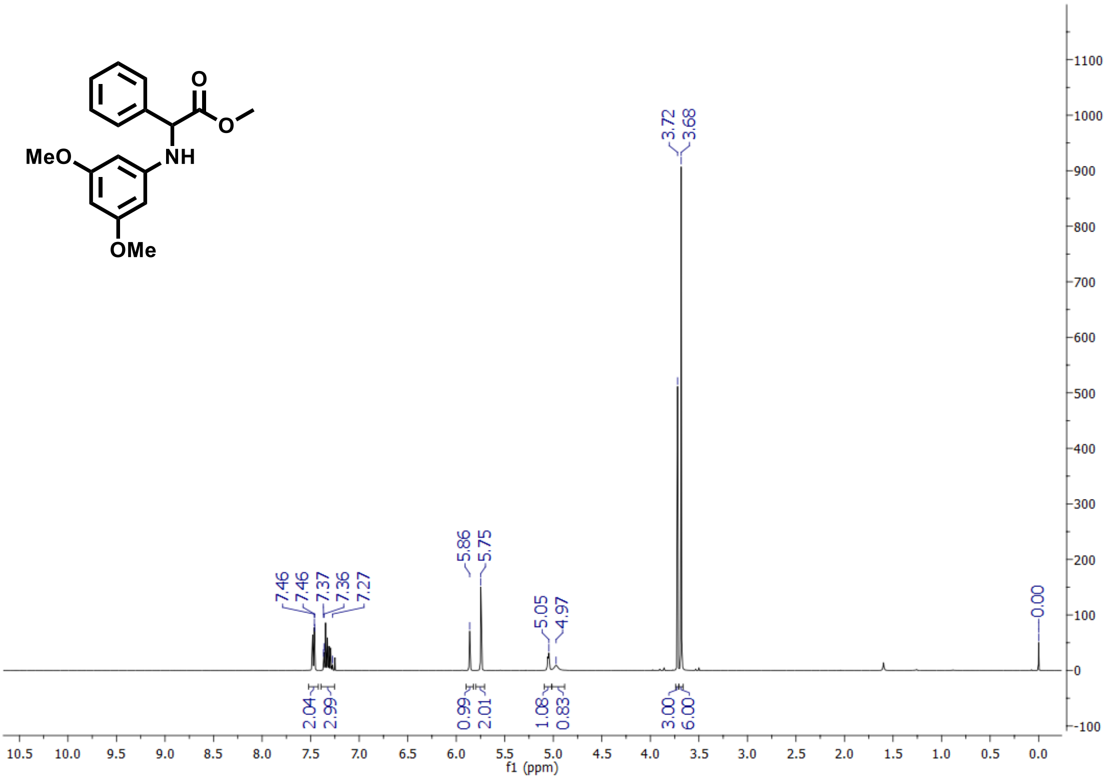

Methyl 2-((3,5-dimethoxyphenyl)amino)-2-phenylacetate (**22**) ^{13}C NMR (126 MHz, $CDCl_3$)

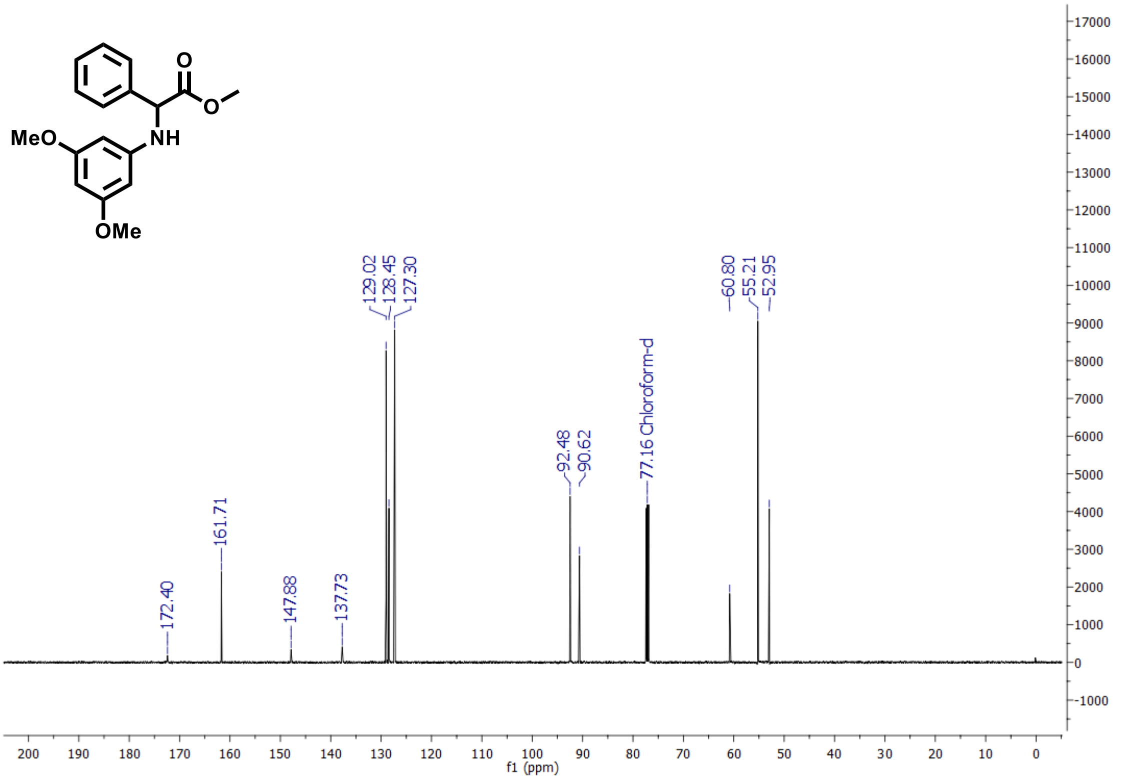

Methyl 2-((3-chloro-4-methoxyphenyl)amino)-2-phenylacetate (**23**) ^{1}H NMR (500 MHz, $CDCl_3$)

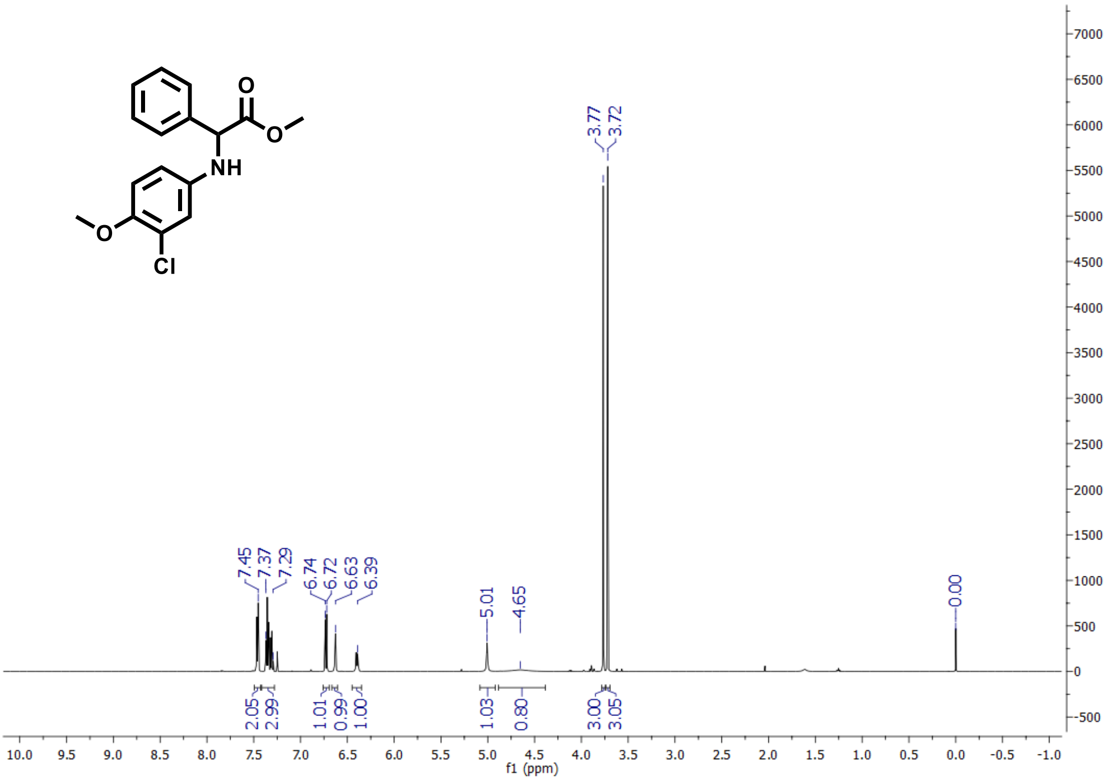

Methyl 2-((3-chloro-4-methoxyphenyl)amino)-2-phenylacetate (**23**) ^{13}C NMR (126 MHz, $CDCl_3$)

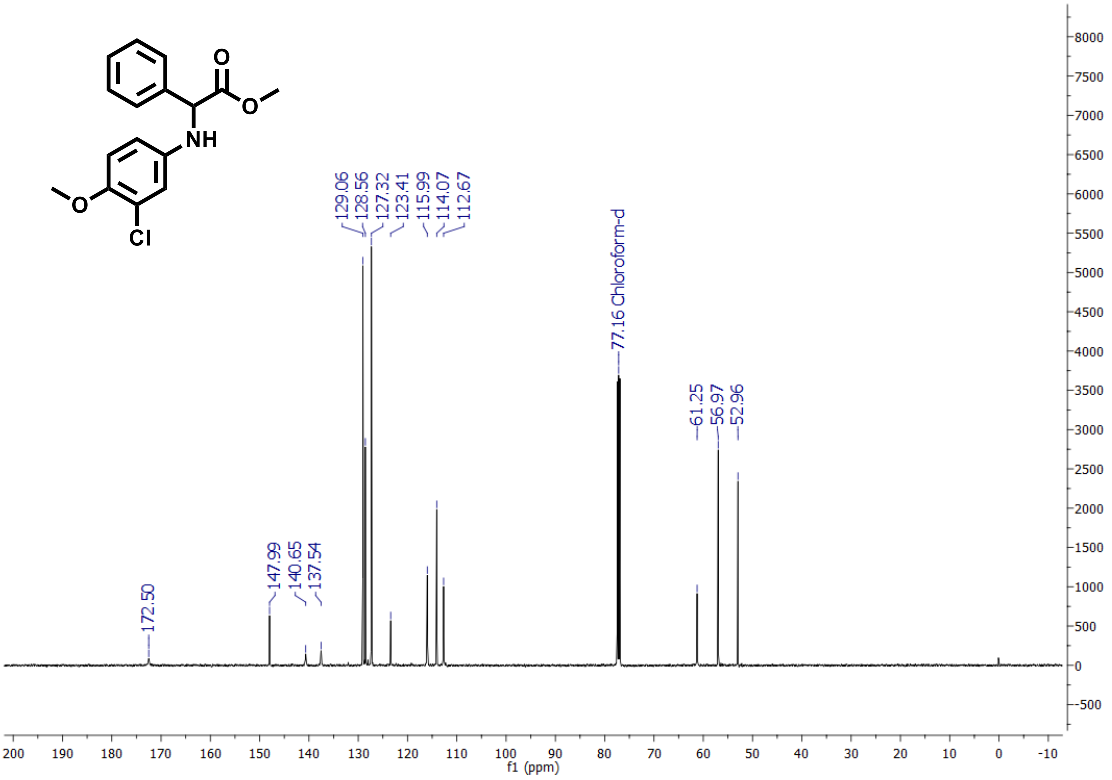

Methyl 2-(methyl(phenyl)amino)-2-phenylacetate (**24**) ^{1}H NMR (400 MHz, $CDCl_3$)

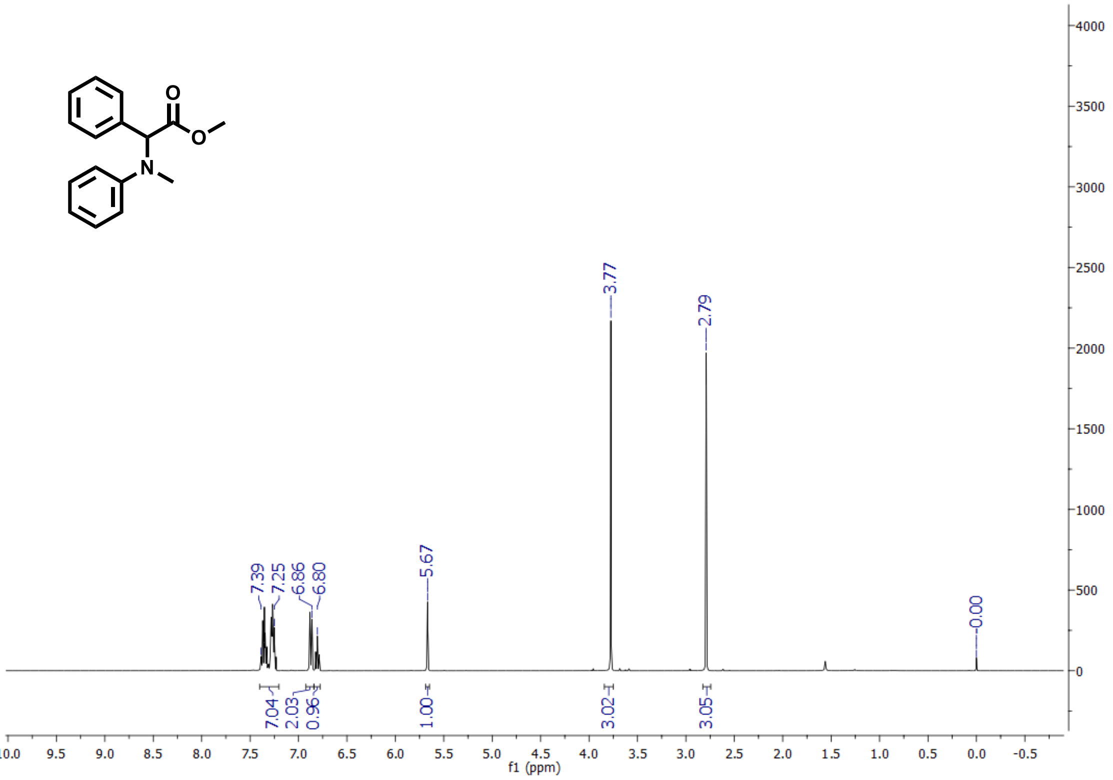

Methyl 2-(methyl(phenyl)amino)-2-phenylacetate (**24**) ^{13}C NMR (126 MHz, $CDCl_3$)

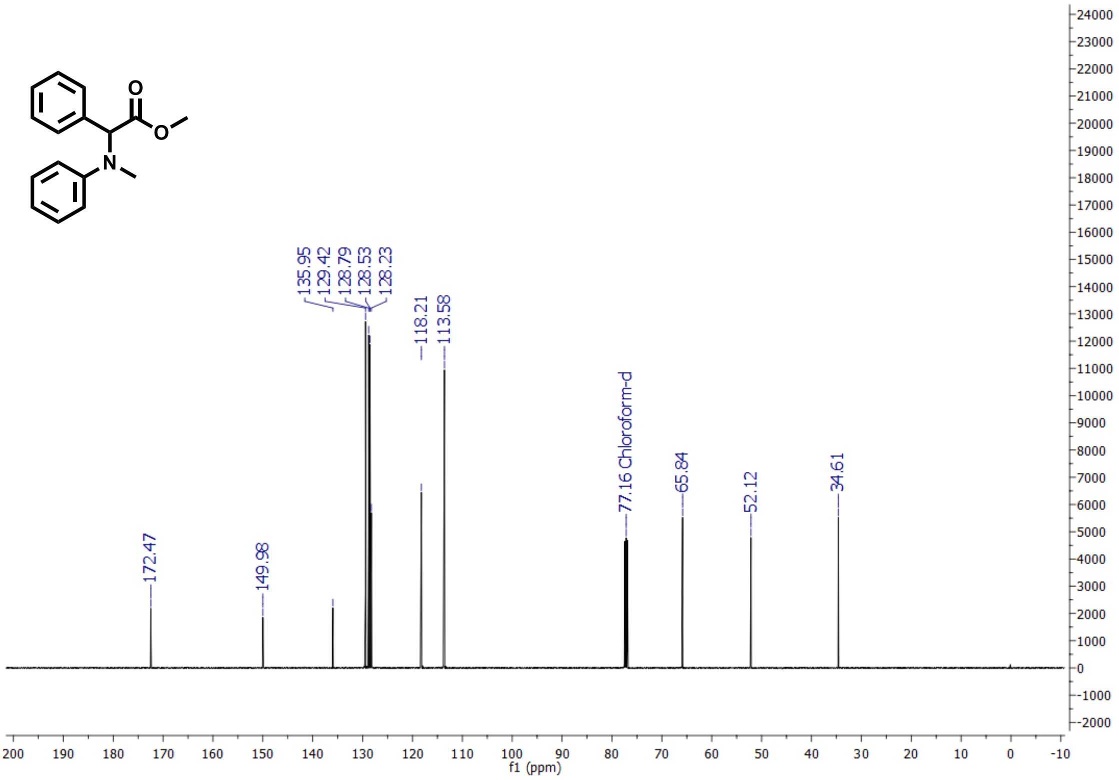

Methyl 2-(methyl(p-tolyl)amino)-2-phenylacetate (**25**) ^{1}H NMR (600 MHz, $CDCl_3$)

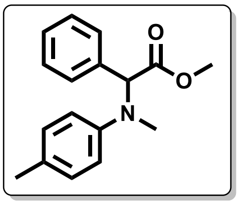

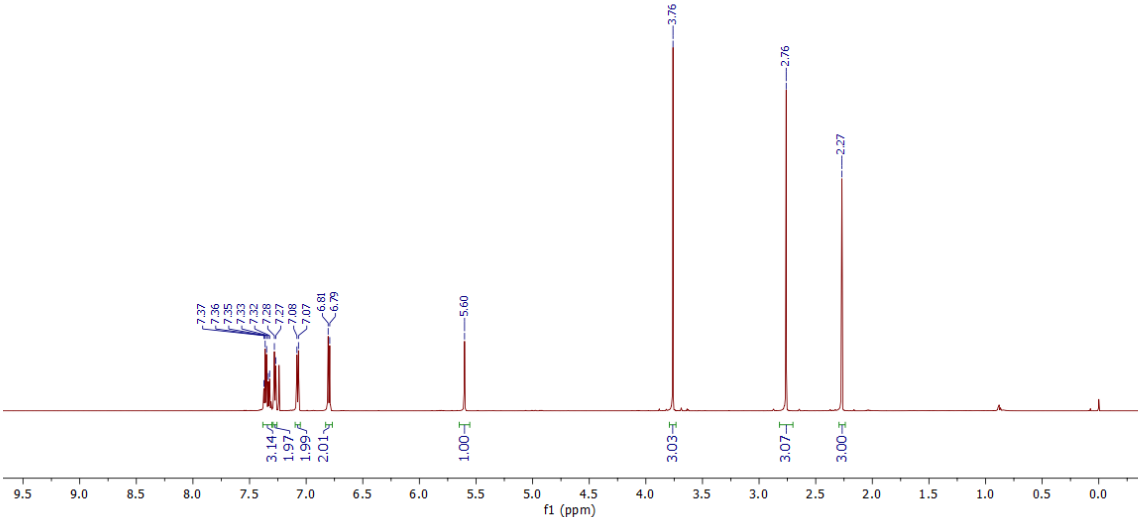

Methyl 2-(methyl(p-tolyl)amino)-2-phenylacetate (**25**) ^{13}C NMR (151 MHz, $CDCl_3$)

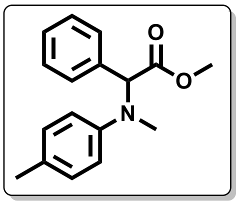

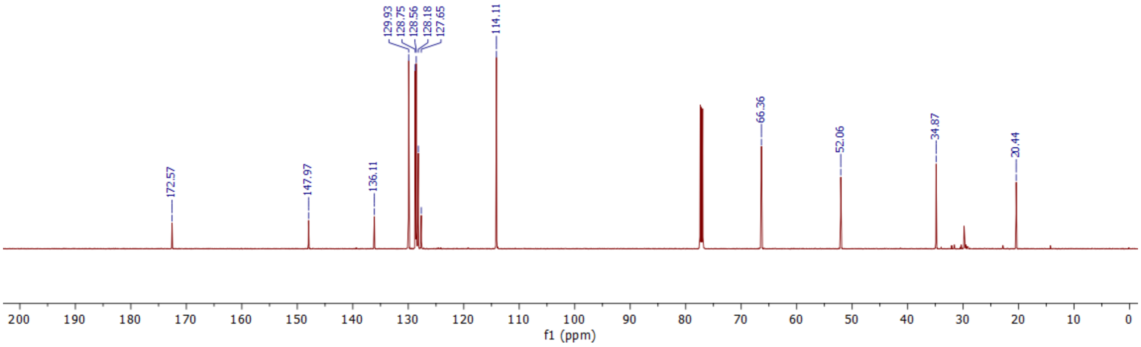

Methyl 2-((4-bromophenyl)(methyl)amino)-2-phenylacetate (**26**) ^{1}H NMR (600 MHz, $CDCl_3$)

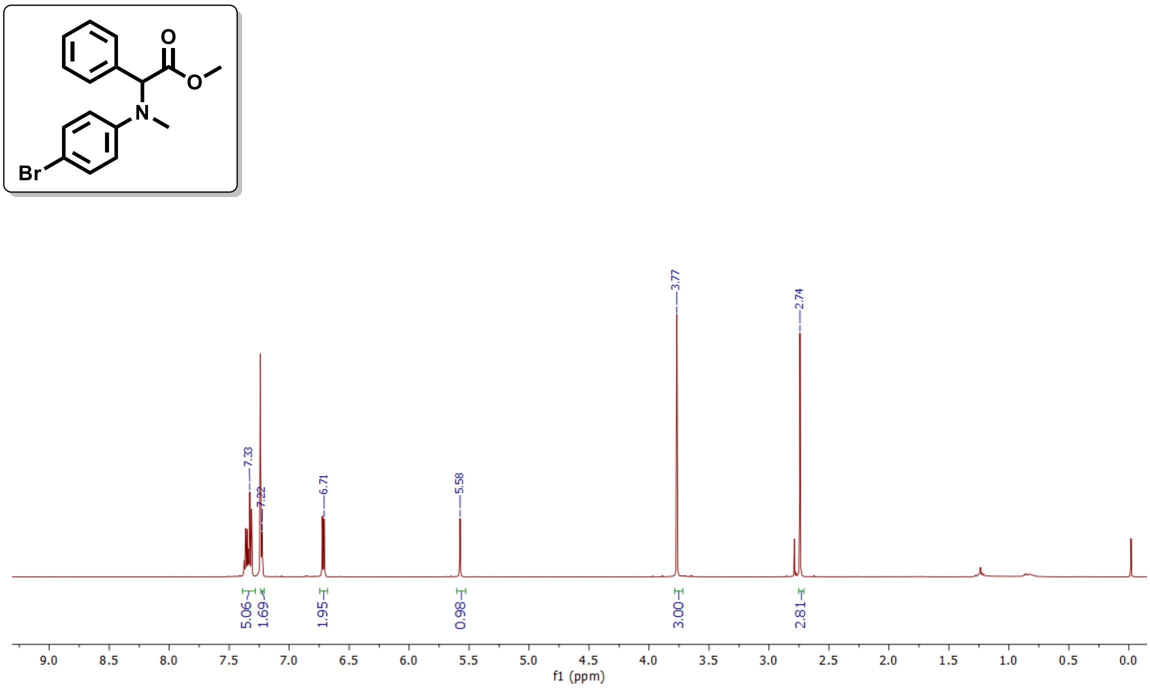

Methyl 2-((4-bromophenyl)(methyl)amino)-2-phenylacetate (**26**) ^{13}C NMR (151 MHz, $CDCl_3$)

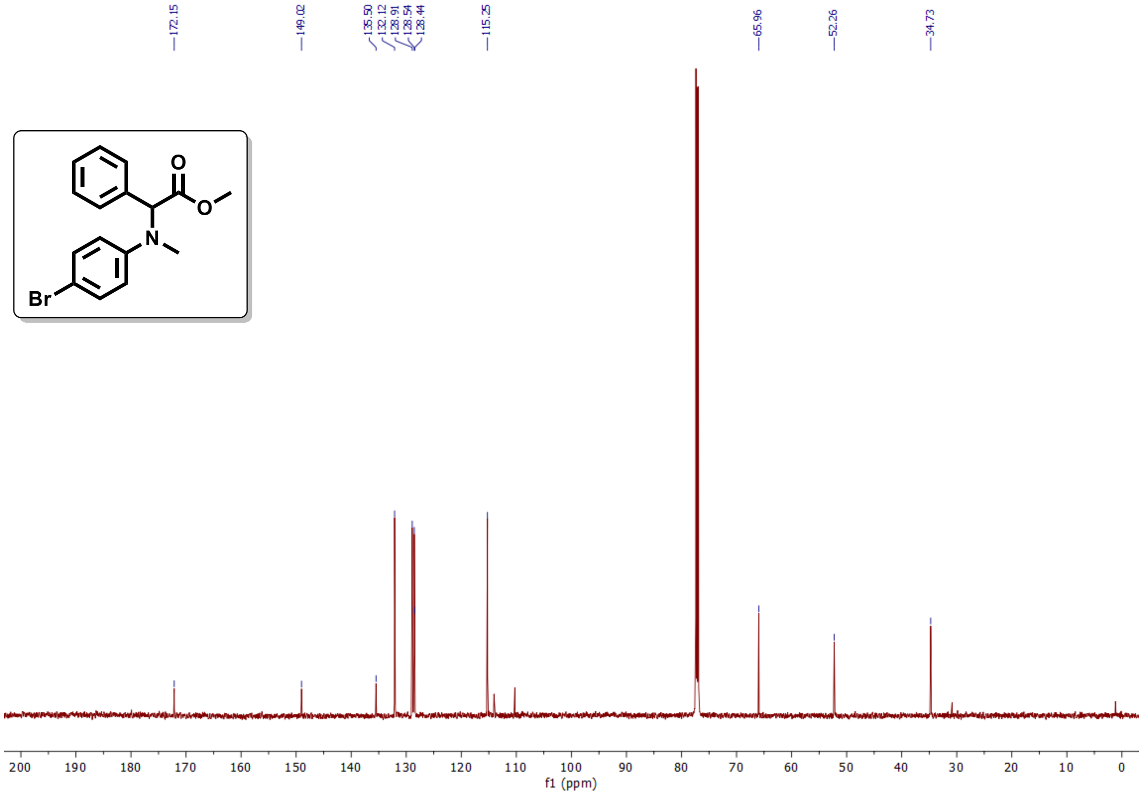

Methyl 2-((3-bromophenyl)(methyl)amino)-2-phenylacetate (**27**) ^{1}H NMR (600 MHz, $CDCl_3$)

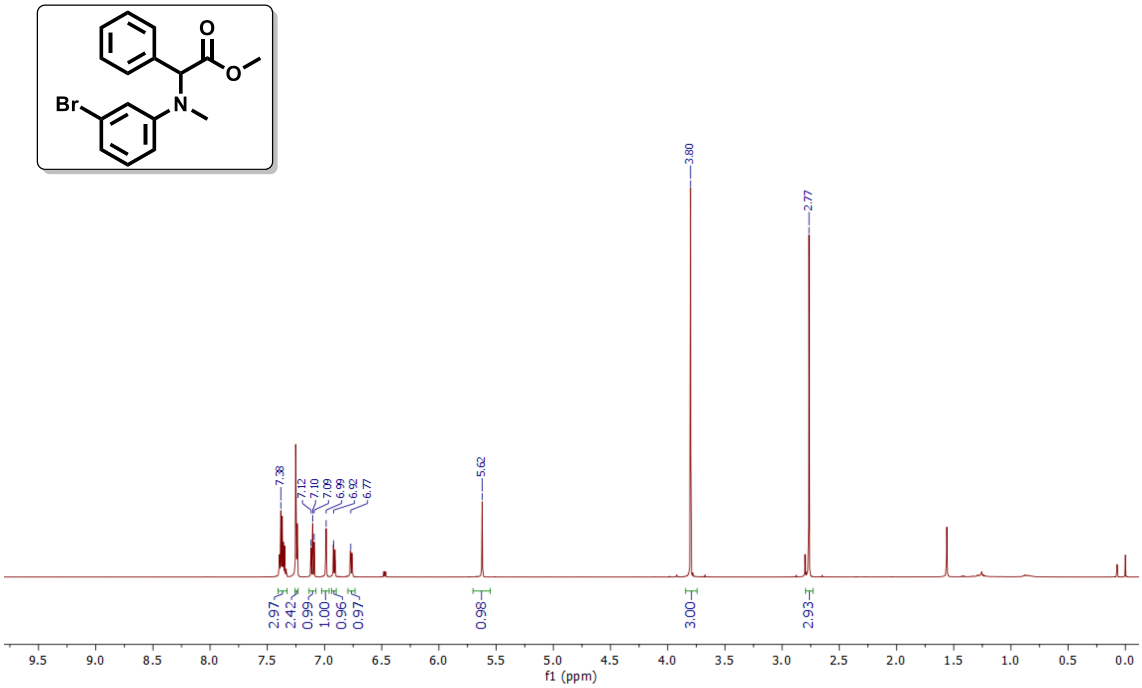

Methyl 2-((3-bromophenyl)(methyl)amino)-2-phenylacetate (**27**) ^{13}C NMR (151 MHz, $CDCl_3$)

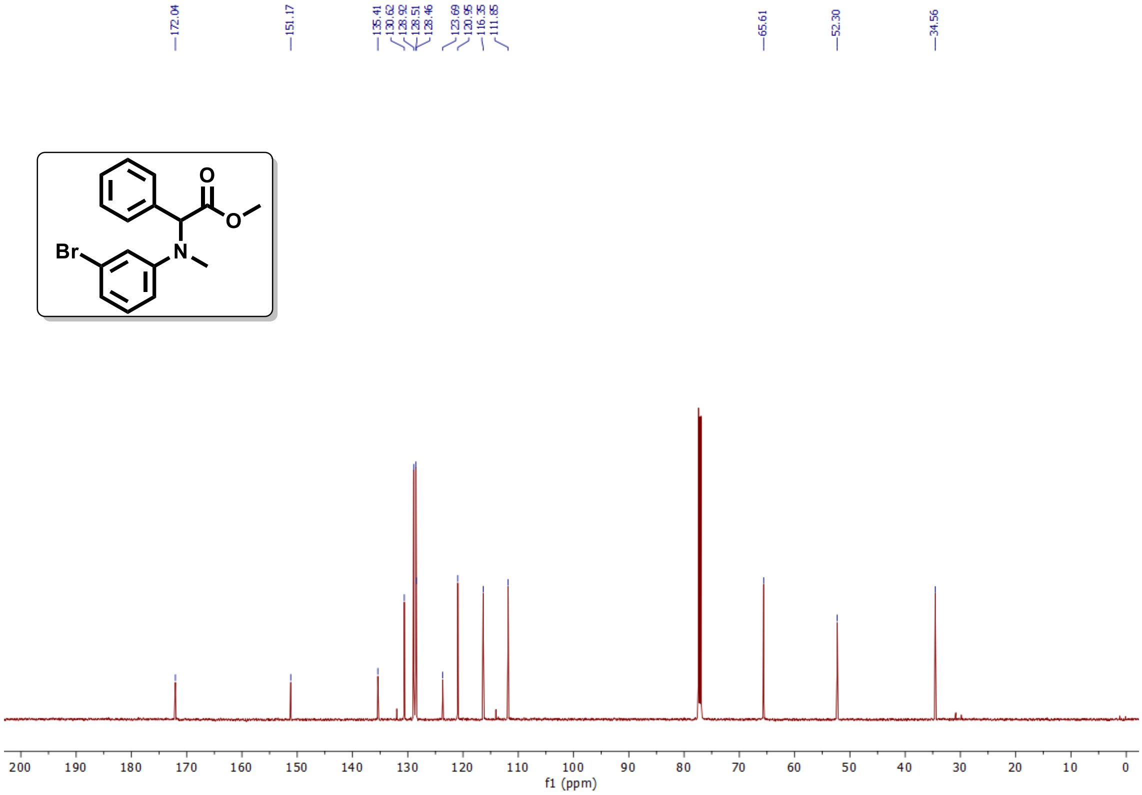

Methyl 2-(4-acetylphenyl)-2-(methyl(phenyl)amino)acetate (**28**) ^{1}H NMR (600 MHz, $CDCl_3$)

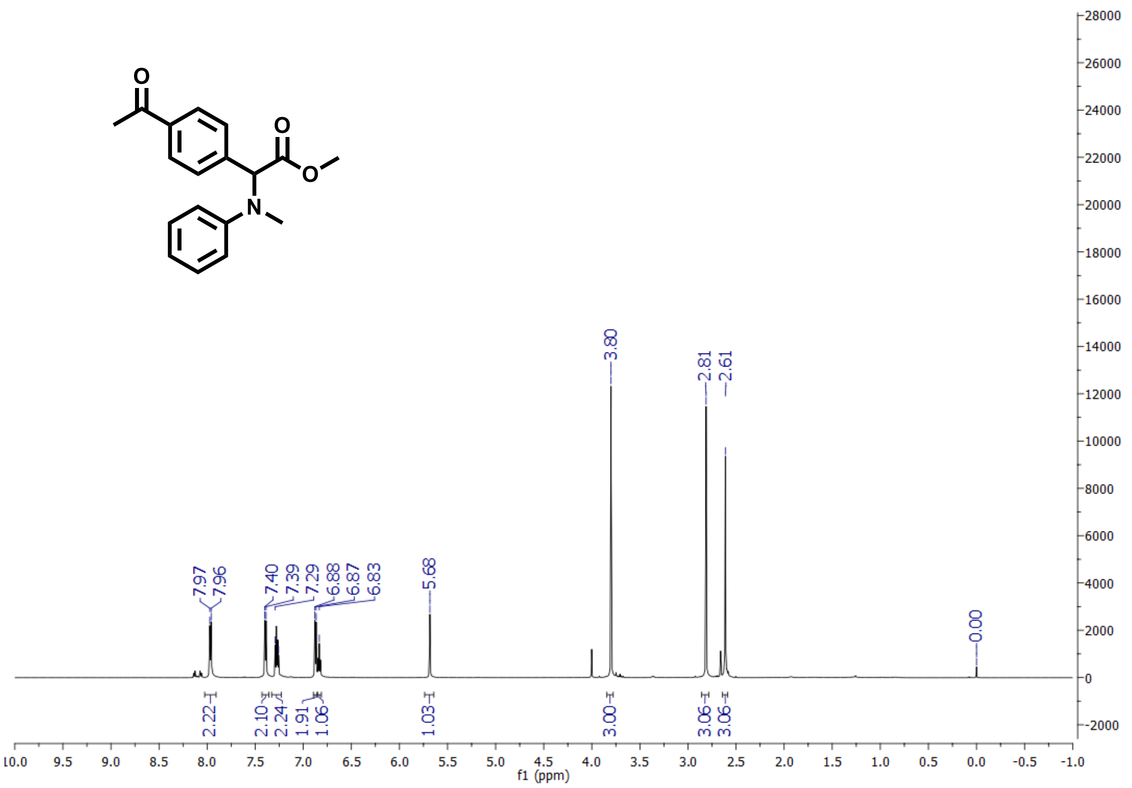

Methyl 2-(4-acetylphenyl)-2-(methyl(phenyl)amino)acetate (**28**) ^{13}C NMR (151 MHz, $CDCl_3$)

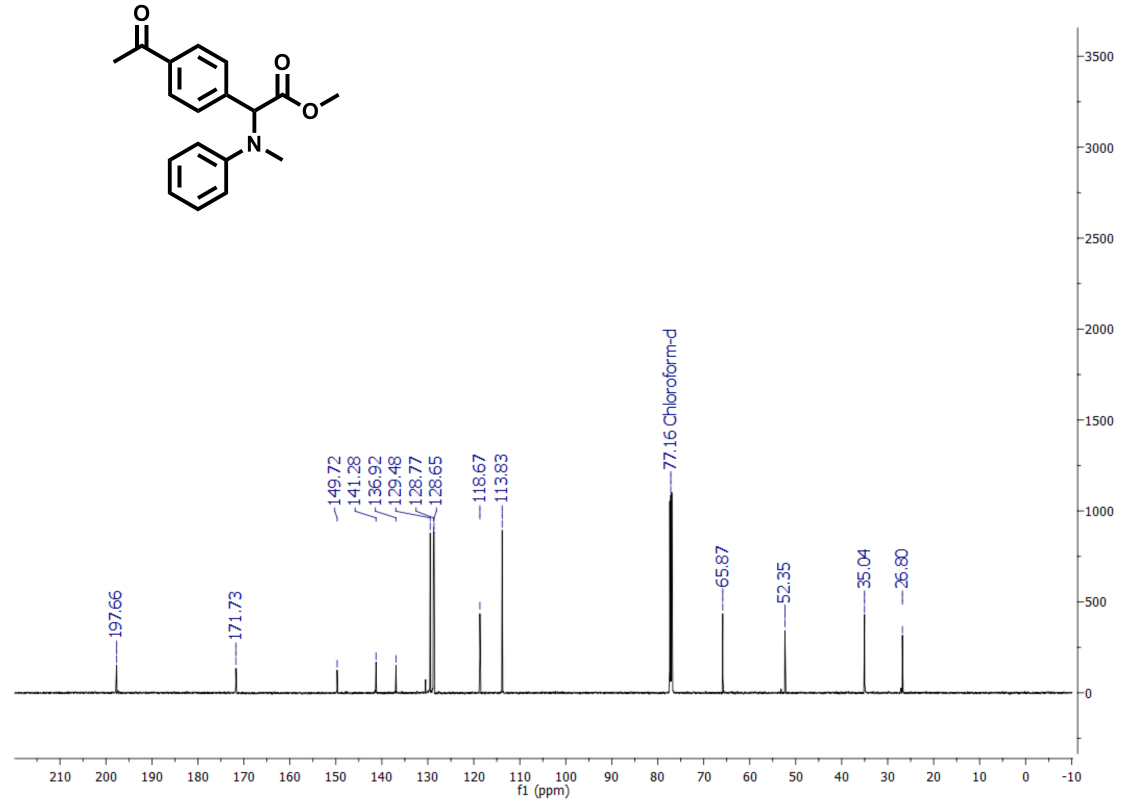

Methyl 4-(2-methoxy-1-(methyl(phenyl)amino)-2-oxoethyl)benzoate (**29**) ^{1}H NMR (600 MHz, $CDCl_3$)

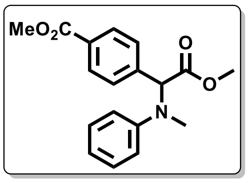

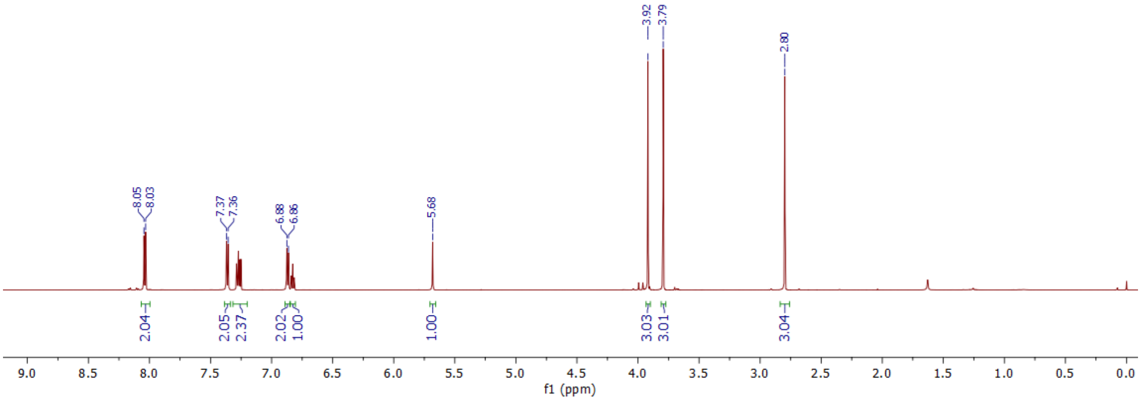

Methyl 4-(2-methoxy-1-(methyl(phenyl)amino)-2-oxoethyl)benzoate (**29**) ^{13}C NMR (151 MHz, $CDCl_3$)

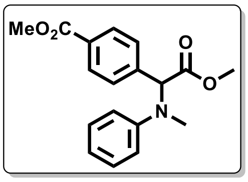

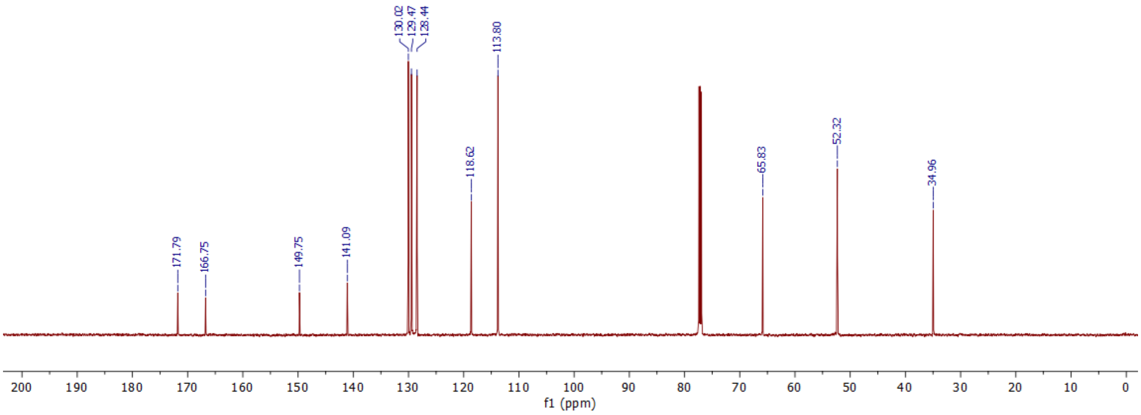

Methyl 2-(4-chlorophenyl)-2-(methyl(phenyl)amino)acetate (**30**) ^{1}H NMR (600 MHz, $CDCl_3$)

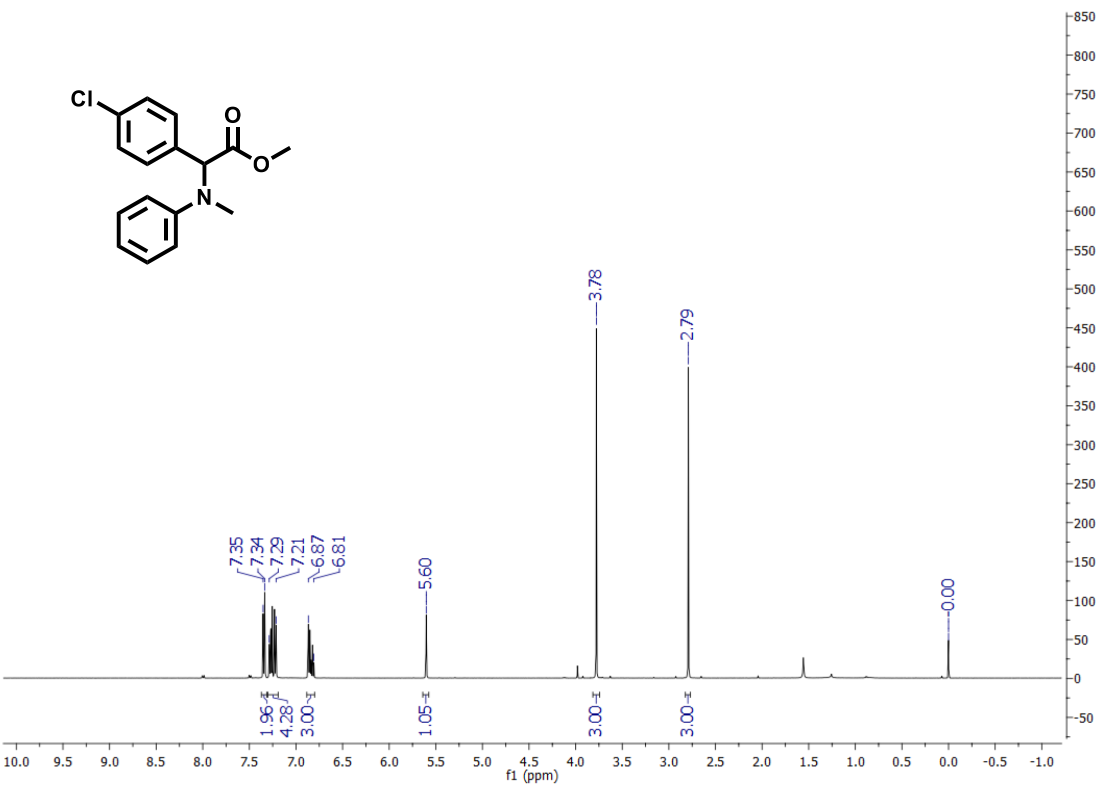

Methyl 2-(4-chlorophenyl)-2-(methyl(phenyl)amino)acetate (**30**) ^{13}C NMR (151 MHz, $CDCl_3$)

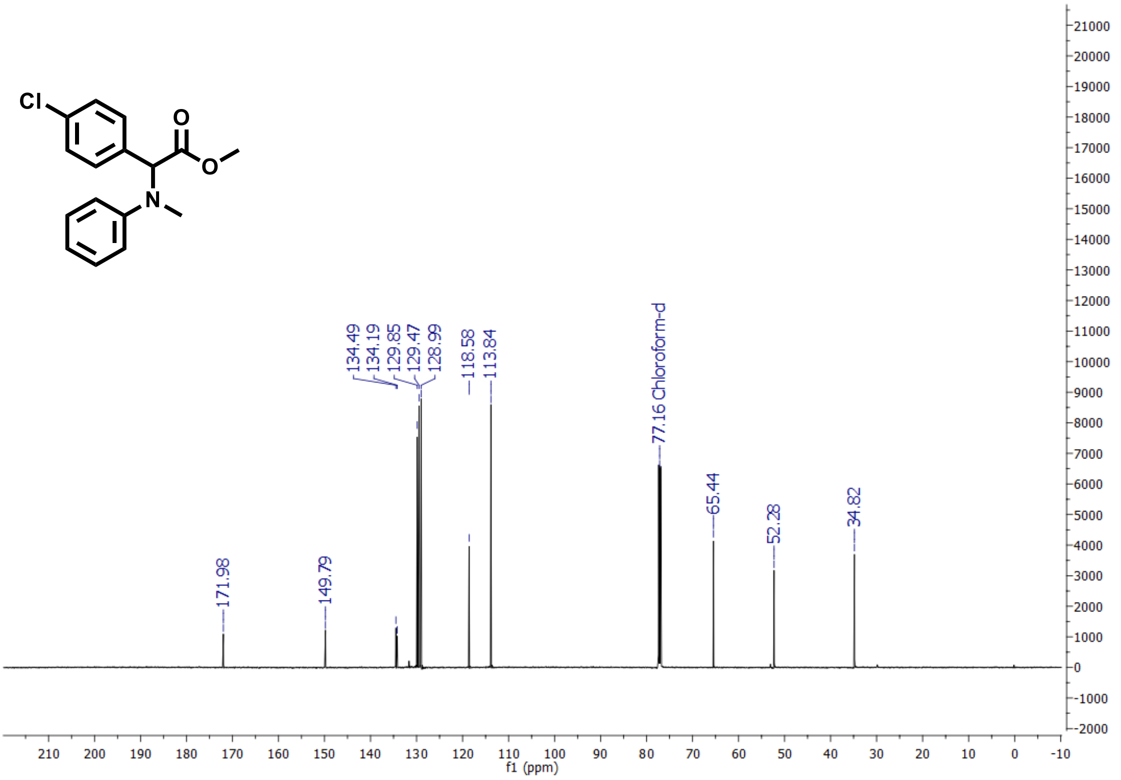

4-(2-methoxy-1-(methyl(phenyl)amino)-2-oxoethyl)phenyl benzoate (**31**) ^{1}H NMR (400 MHz, $CDCl_3$)

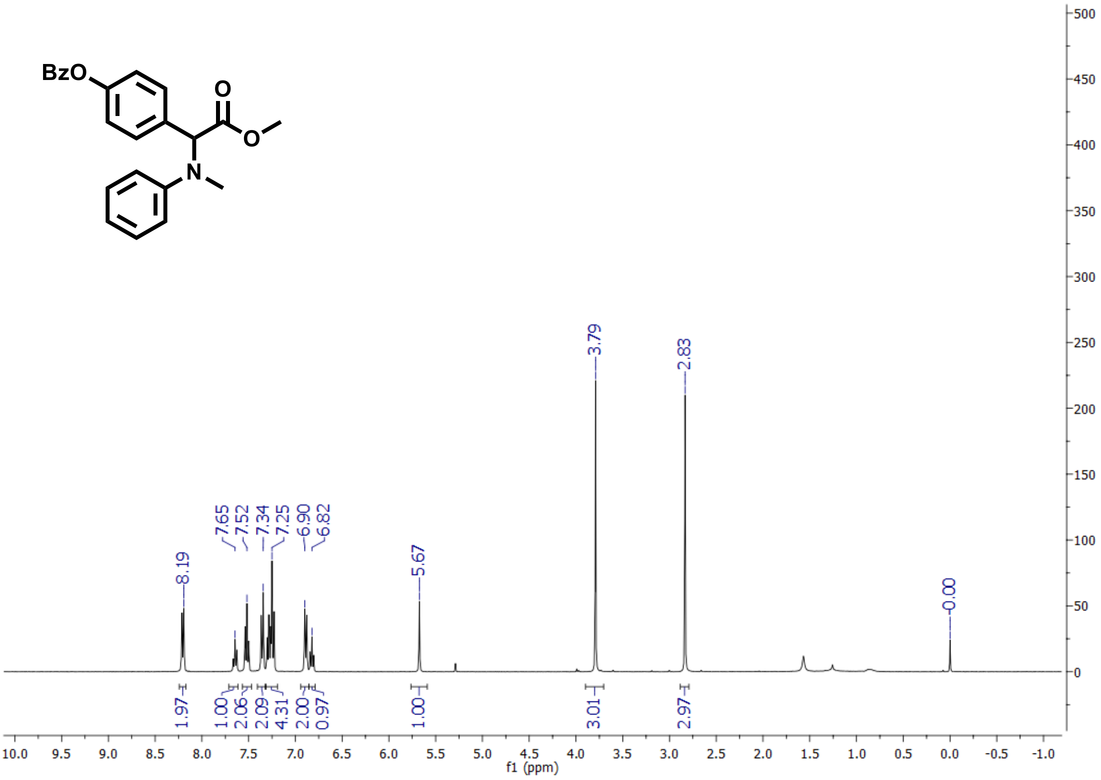

4-(2-methoxy-1-(methyl(phenyl)amino)-2-oxoethyl)phenyl benzoate (**31**) ^{13}C NMR (151 MHz, $CDCl_3$)

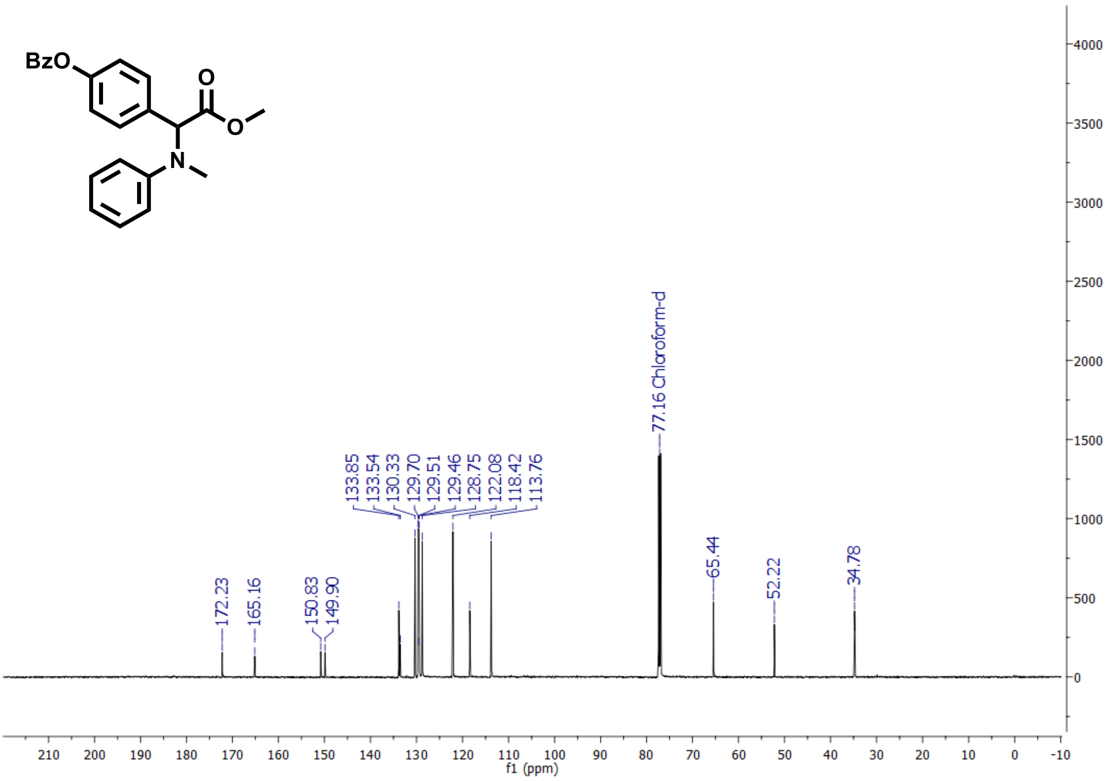

Methyl 2-(3,5-dimethylphenyl)-2-(methyl(phenyl)amino)acetate (**32**) ^{1}H NMR (600 MHz, $CDCl_3$)

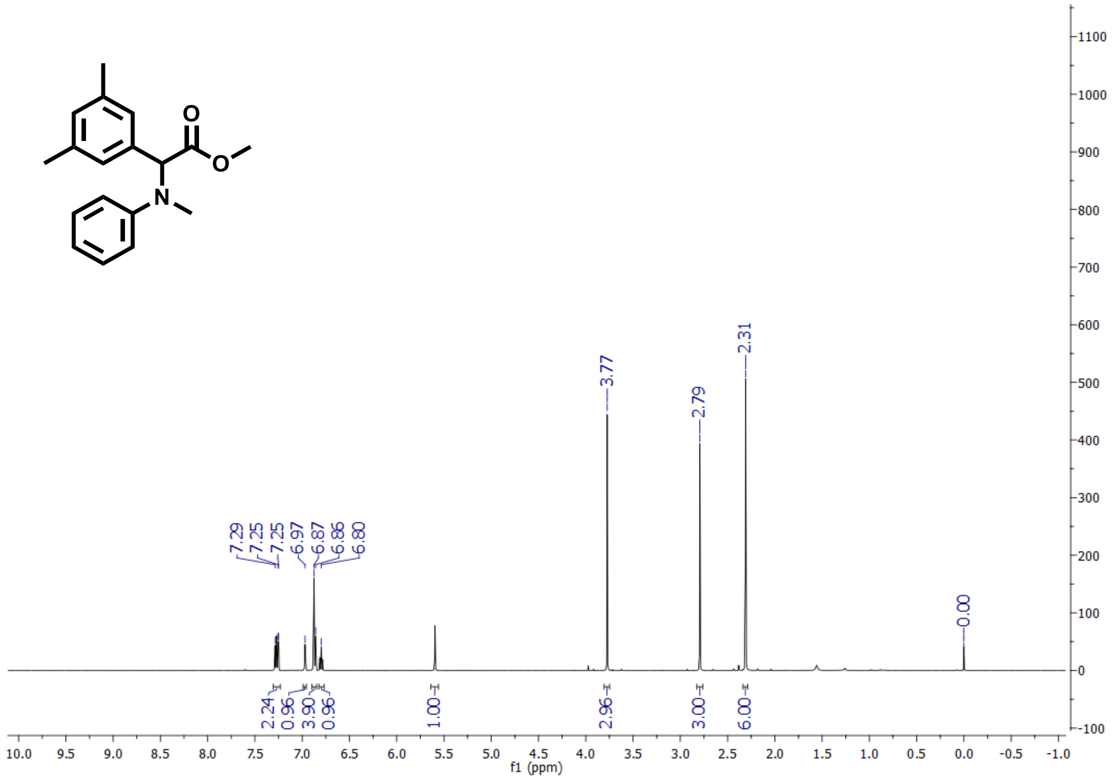

Methyl 2-(3,5-dimethylphenyl)-2-(methyl(phenyl)amino)acetate (**32**) ^{13}C NMR (151 MHz, $CDCl_3$)

,

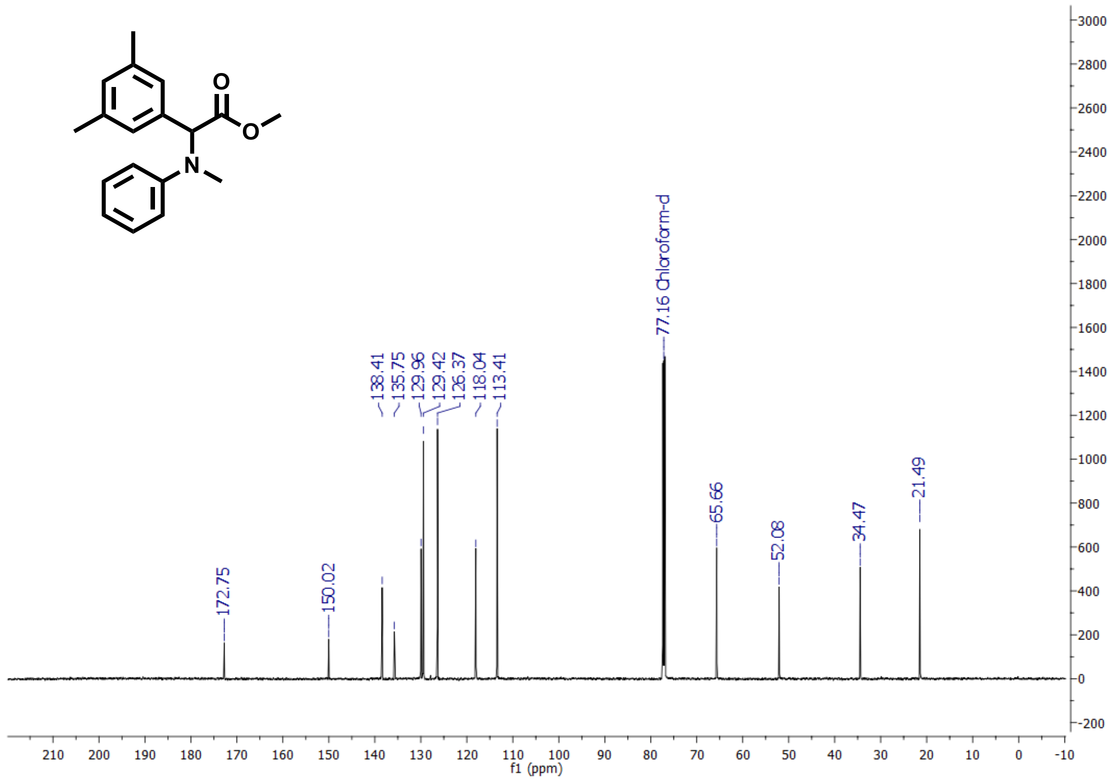

Methyl 2-(2-methoxyphenyl)-2-(methyl(phenyl)amino)acetate (**33**) ^{1}H NMR (600 MHz, $CDCl_3$)

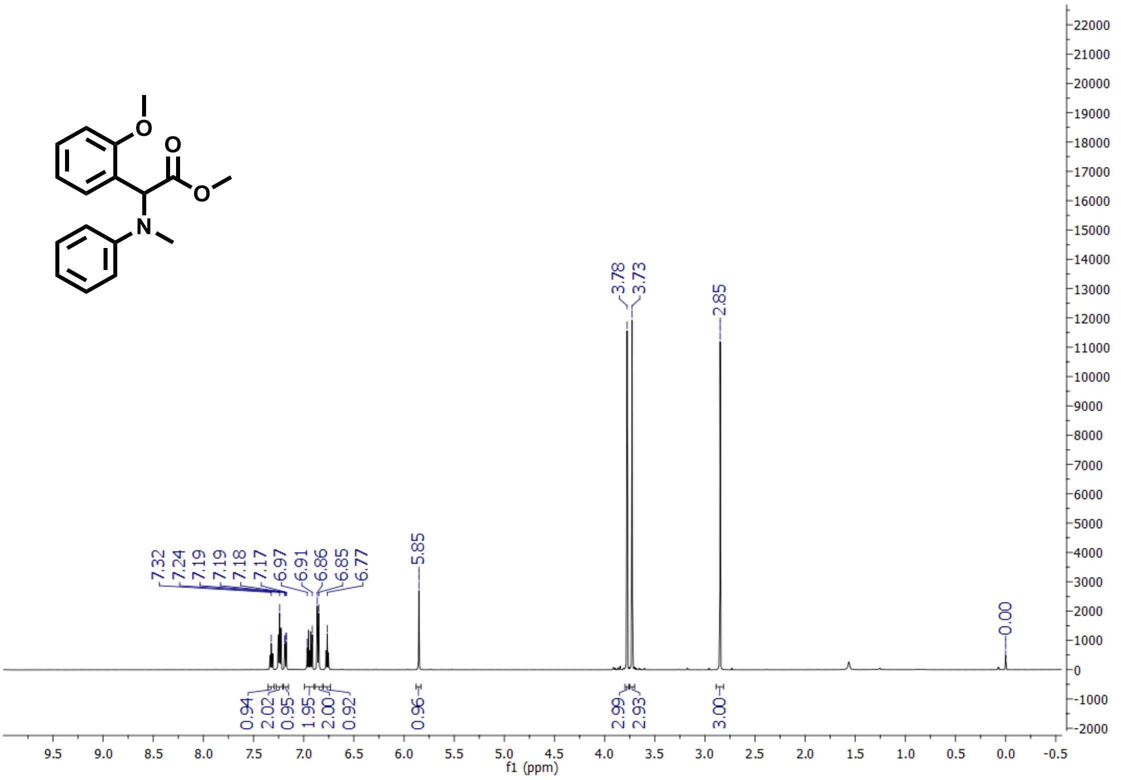

Methyl 2-(2-methoxyphenyl)-2-(methyl(phenyl)amino)acetate (**33**) ^{13}C NMR (151 MHz, $CDCl_3$)

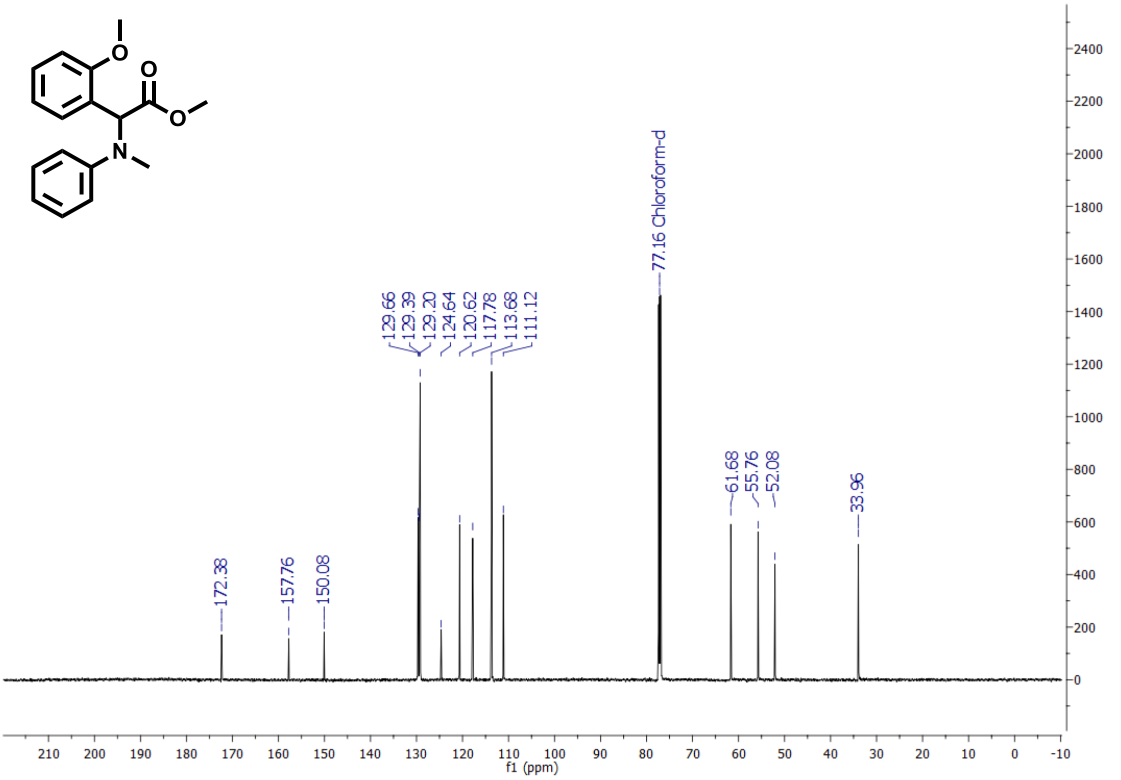

Methyl 2-(benzo[d][1,3]dioxol-5-yl)-2-((4-bromophenyl)(methyl)amino)acetate (**34**) ^{1}H NMR (600 MHz, $CDCl_3$)

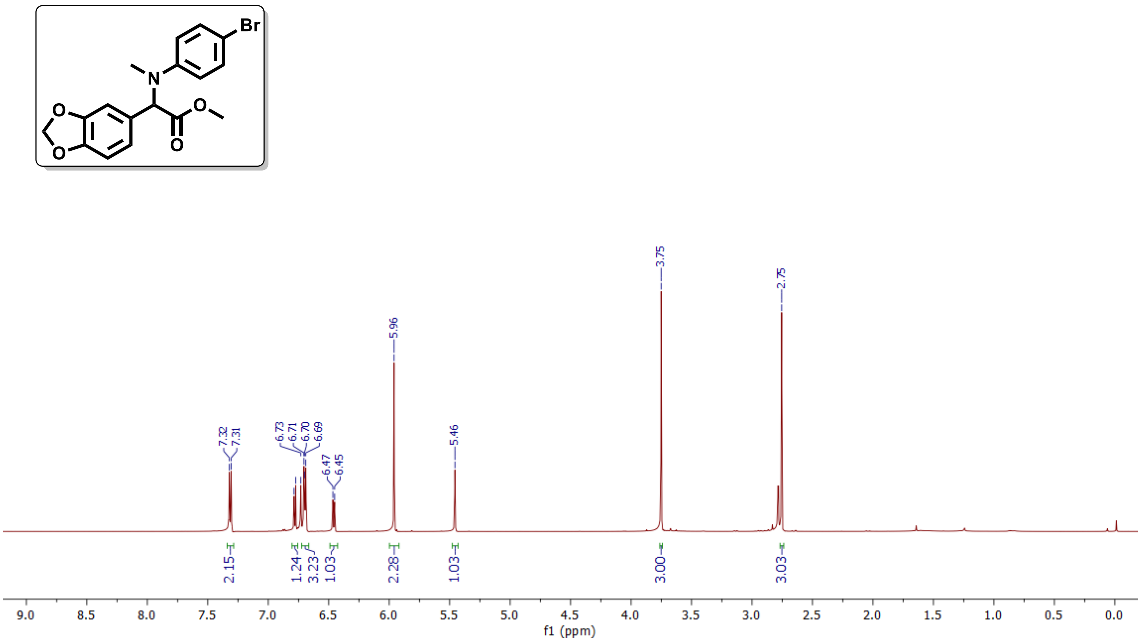

Methyl 2-(benzo[d][1,3]dioxol-5-yl)-2-((4-bromophenyl)(methyl)amino)acetate (**34**) ^{13}C NMR (151 MHz, $CDCl_3$)

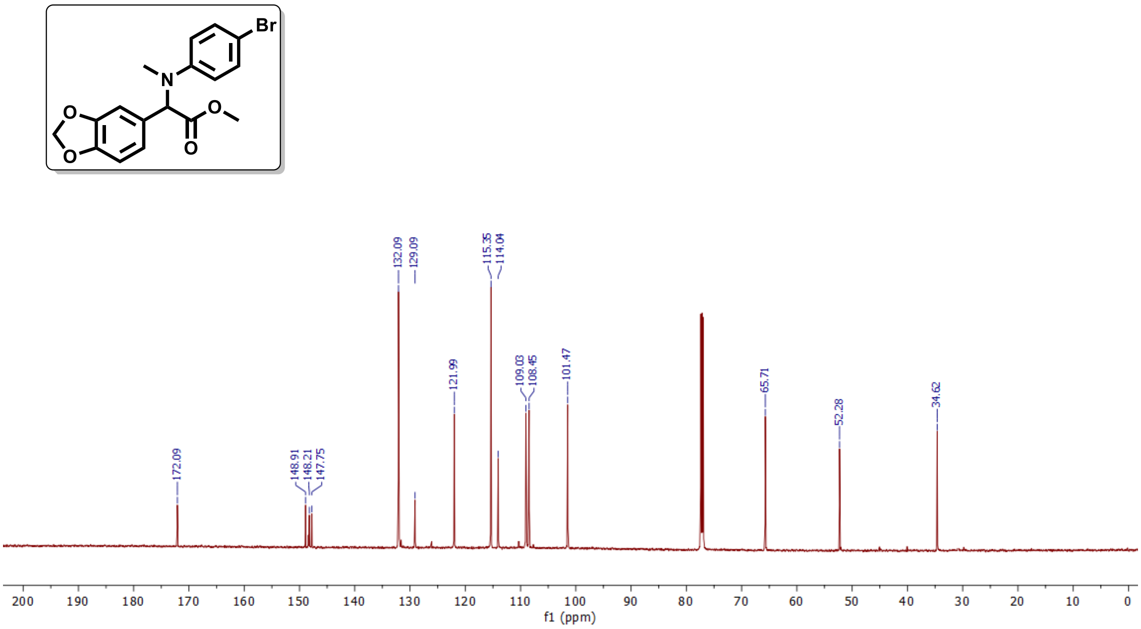

Methyl 2-((4-bromophenyl)(methyl)amino)-2-(2-methoxyphenyl)acetate (**35**) ^{1}H NMR (600 MHz, $CDCl_3$)

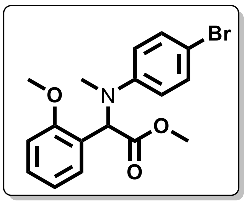

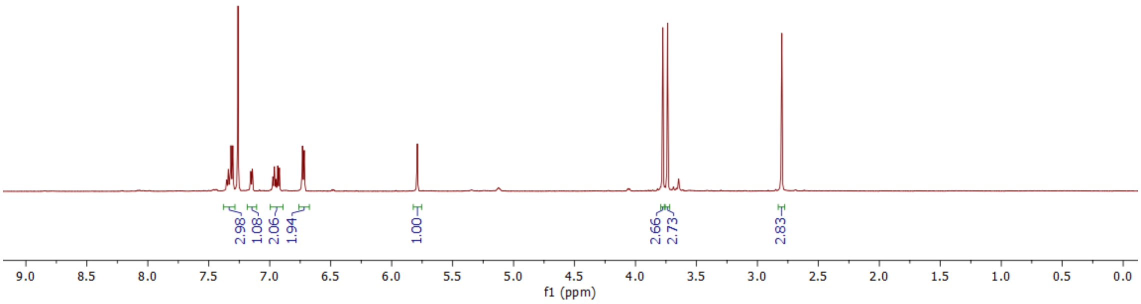

Methyl 2-((4-bromophenyl)(methyl)amino)-2-(2-methoxyphenyl)acetate (**35**) ^{13}CNMR (151 MHz, $CDCl_3$)

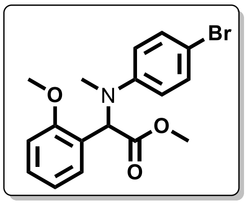

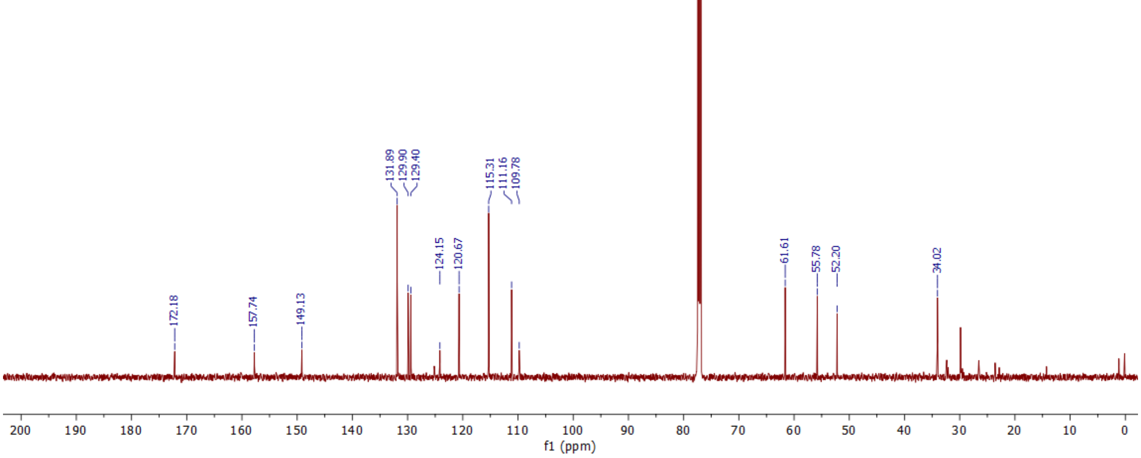

Methyl 2-(benzo[d][1,3]dioxol-5-yl)-2-(methyl(p-tolyl)amino)acetate (**36**) ^{1}H NMR (600 MHz, $CDCl_3$)

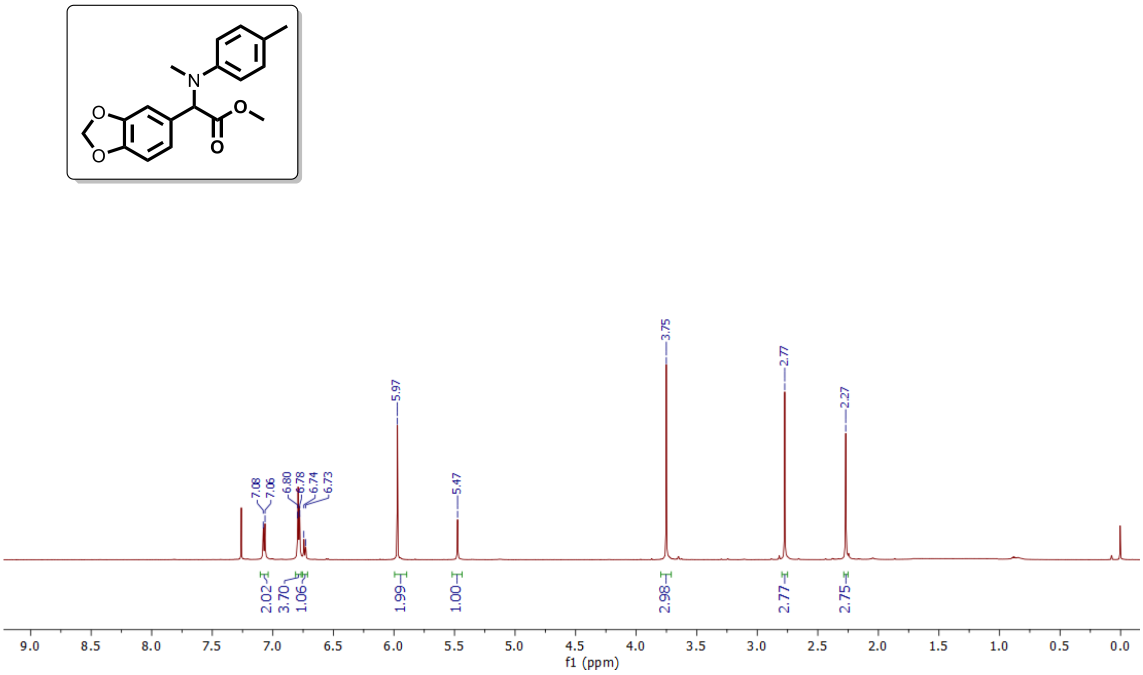

Methyl 2-(benzo[d][1,3]dioxol-5-yl)-2-(methyl(p-tolyl)amino)acetate (**36**) ^{13}C NMR (151 MHz, $CDCl_3$)

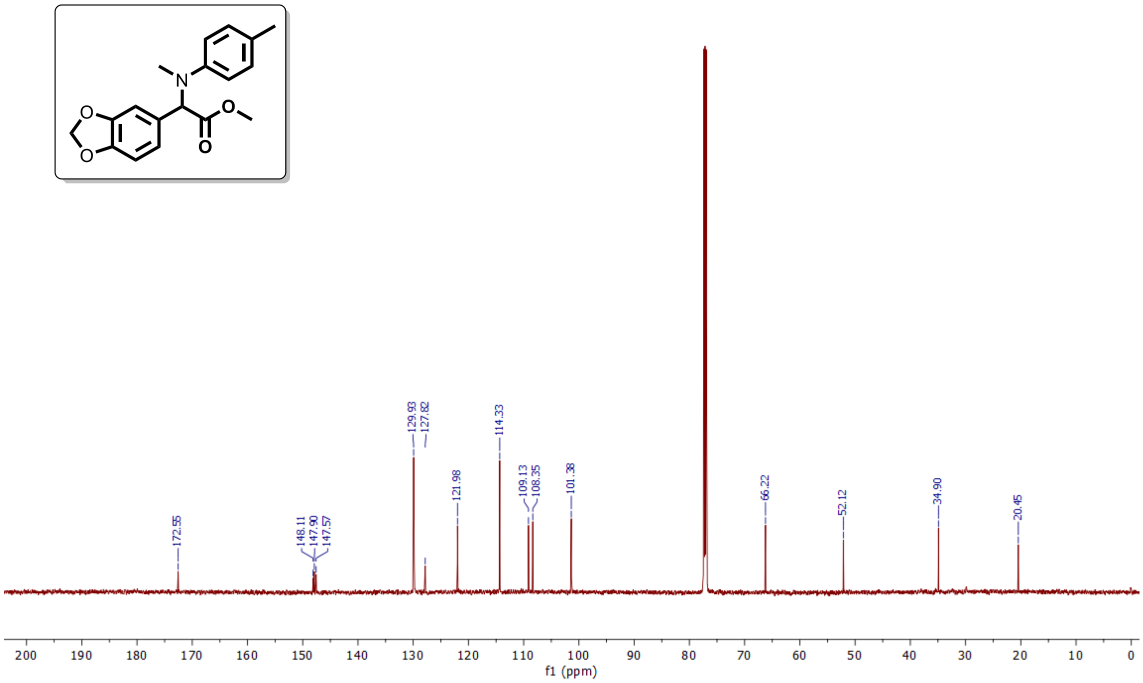

Methyl 2-((4-bromophenyl)amino)-2-(2-methoxyphenyl)acetate (**37**) ^{1}H NMR (600 MHz, $CDCl_3$)

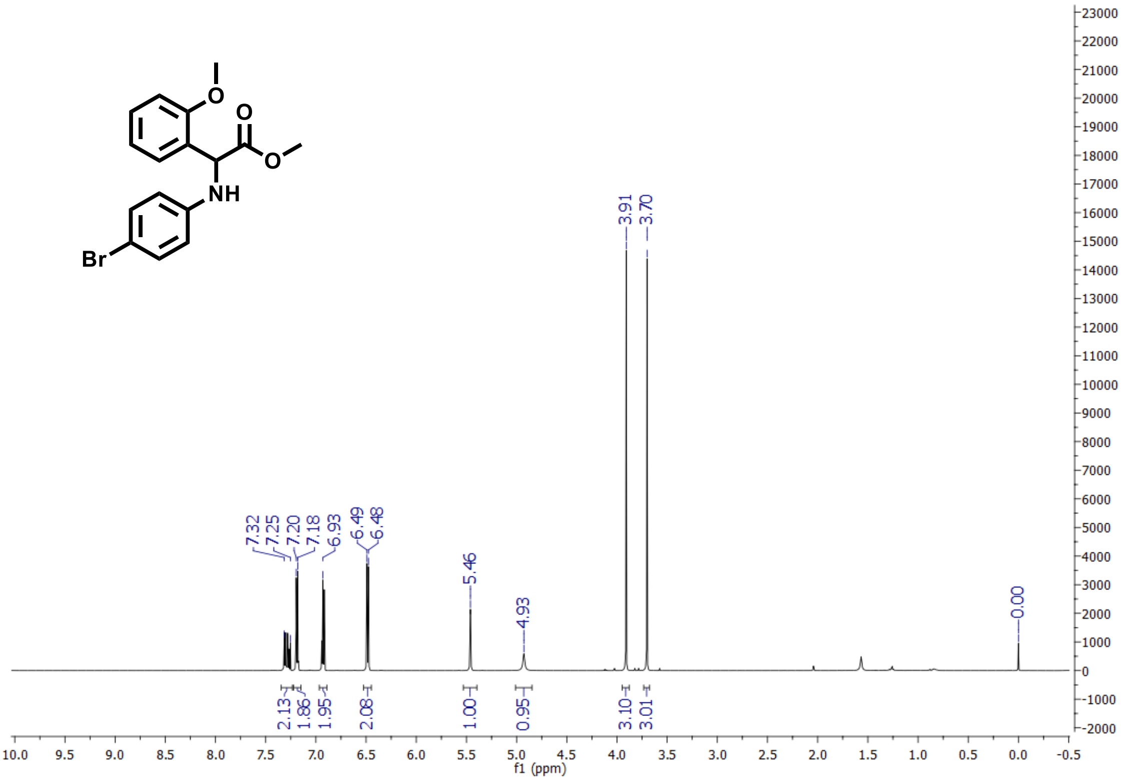

Methyl 2-((4-bromophenyl)amino)-2-(2-methoxyphenyl)acetate (**37**) ^{13}C NMR (151 MHz, $CDCl_3$)

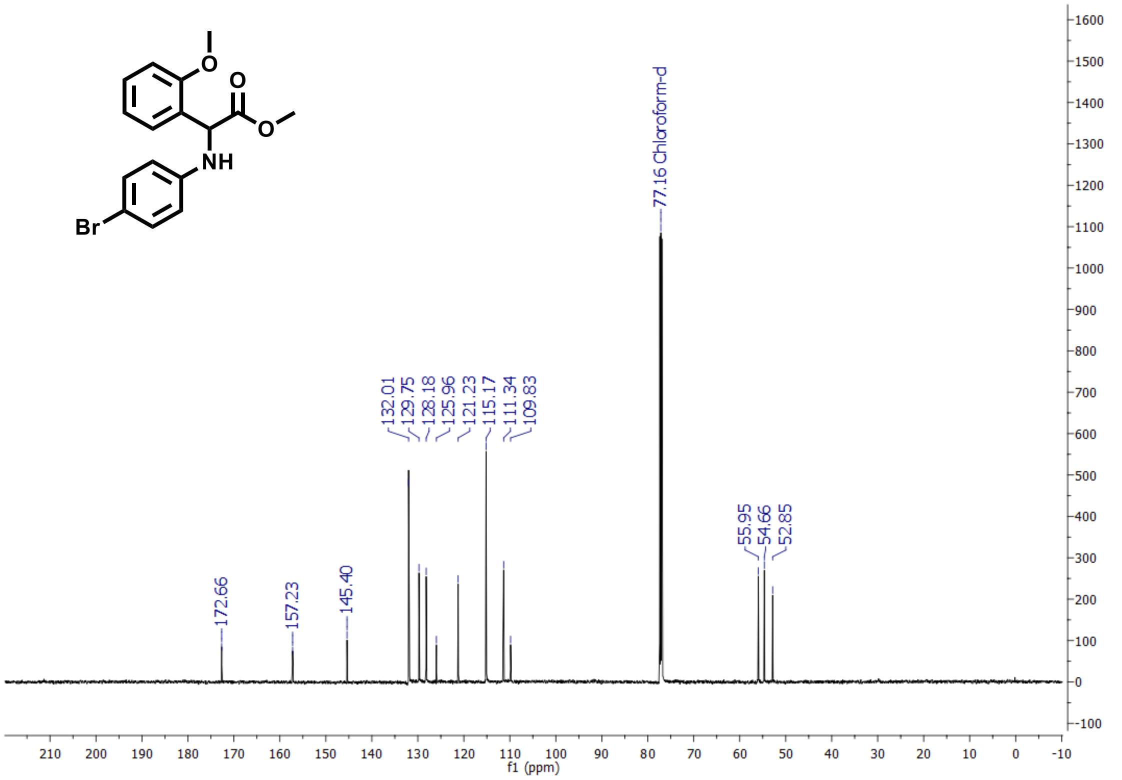

Methyl 2-((4-bromophenyl)amino)-2-(3,5-dimethylphenyl)acetate (**38**) ^{1}H NMR (400 MHz, $CDCl_3$)

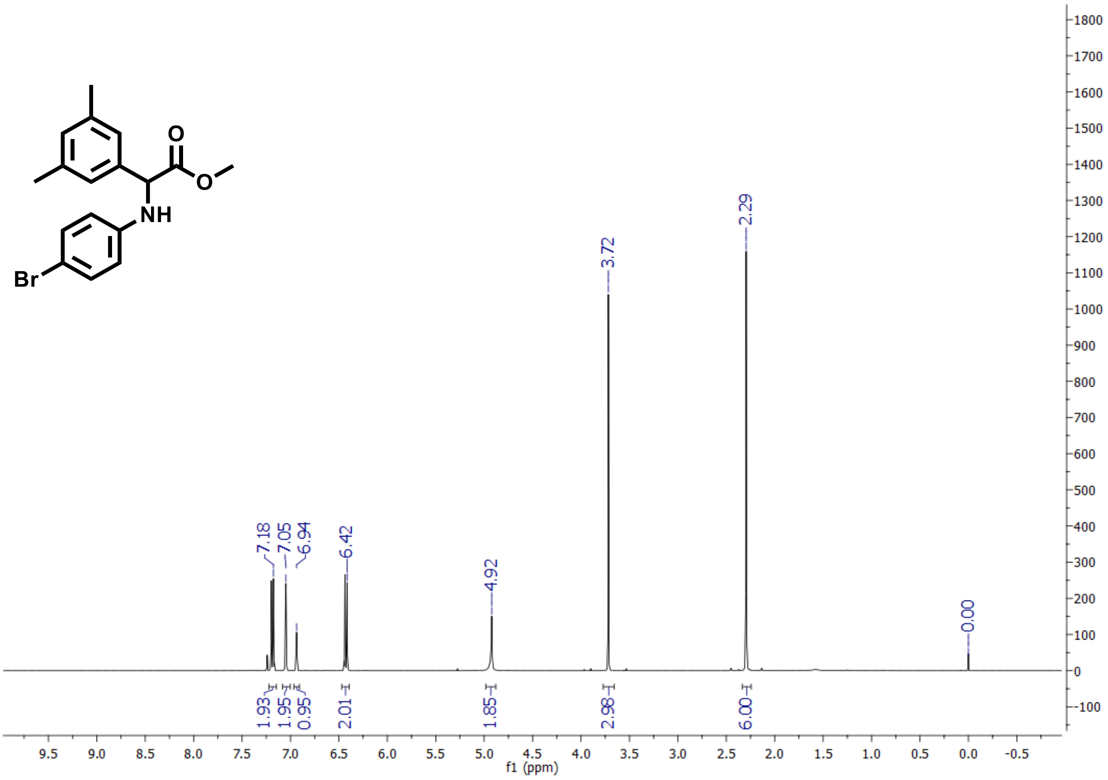

Methyl 2-((4-bromophenyl)amino)-2-(3,5-dimethylphenyl)acetate (**38**) ^{13}C NMR (101 MHz, $CDCl_3$)

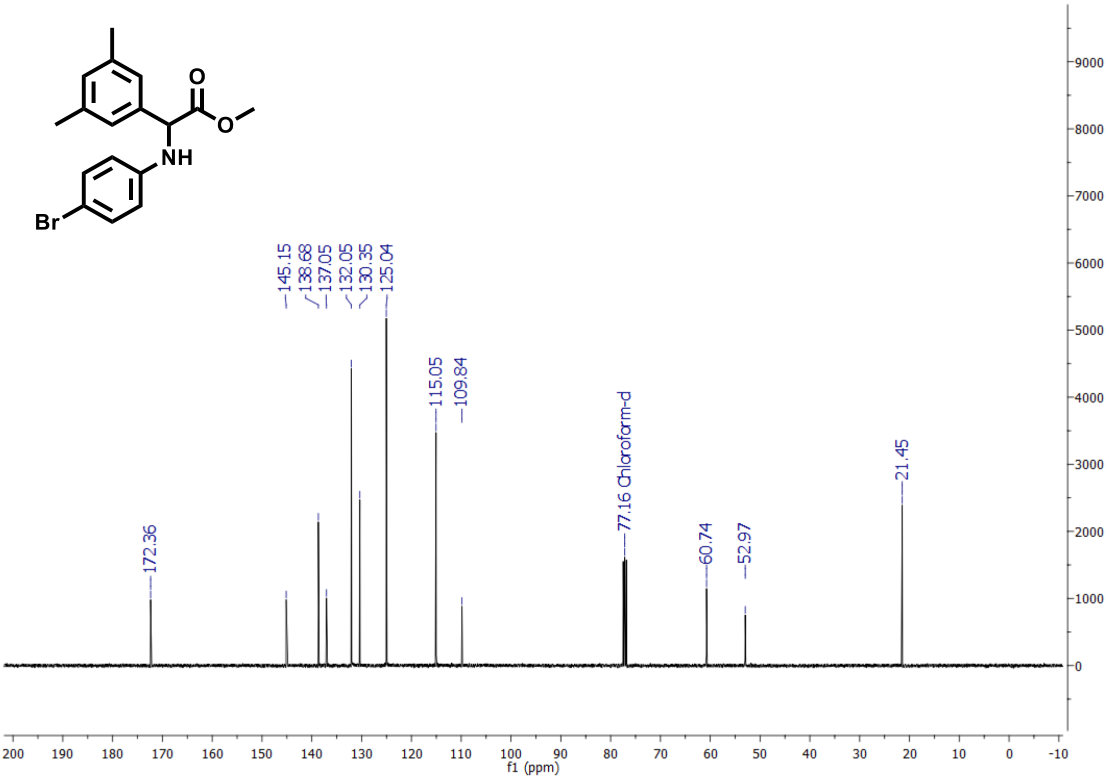

Methyl 2-((4-bromophenyl)amino)-2-(4-chlorophenyl)acetate (**39**) ^{1}H NMR (400 MHz, $CDCl_3$)

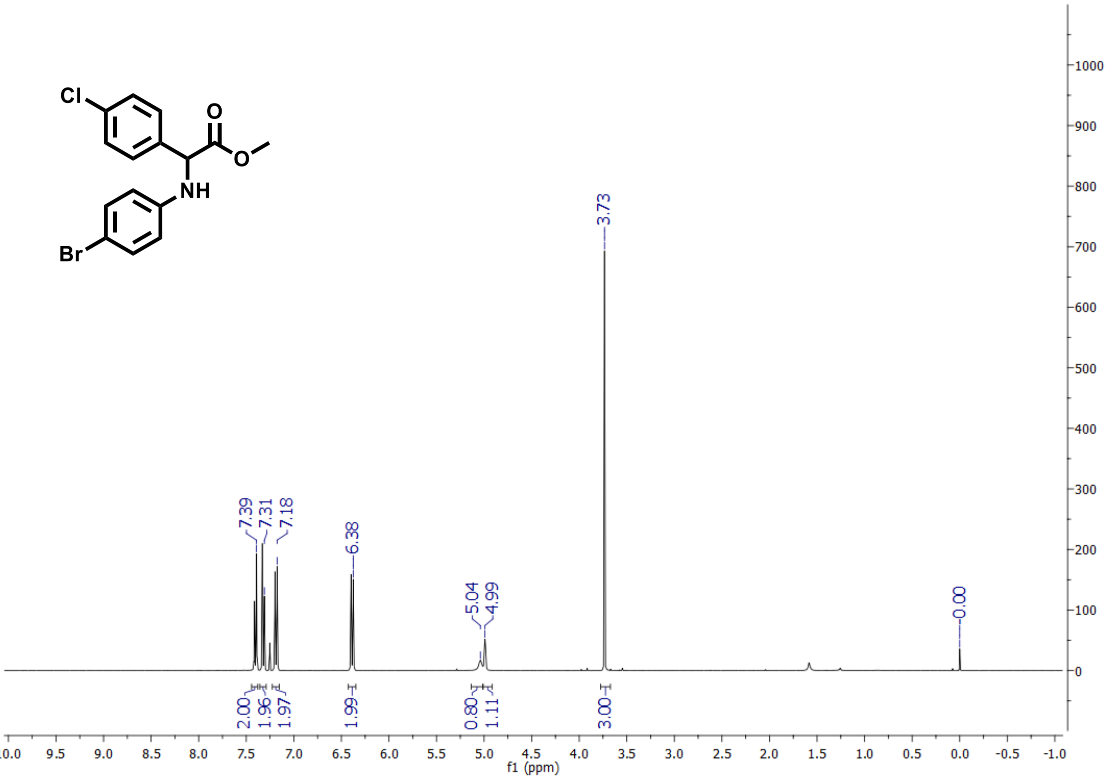

Methyl 2-((4-bromophenyl)amino)-2-(4-chlorophenyl)acetate (**39**) ^{13}C NMR (101 MHz, $CDCl_3$)

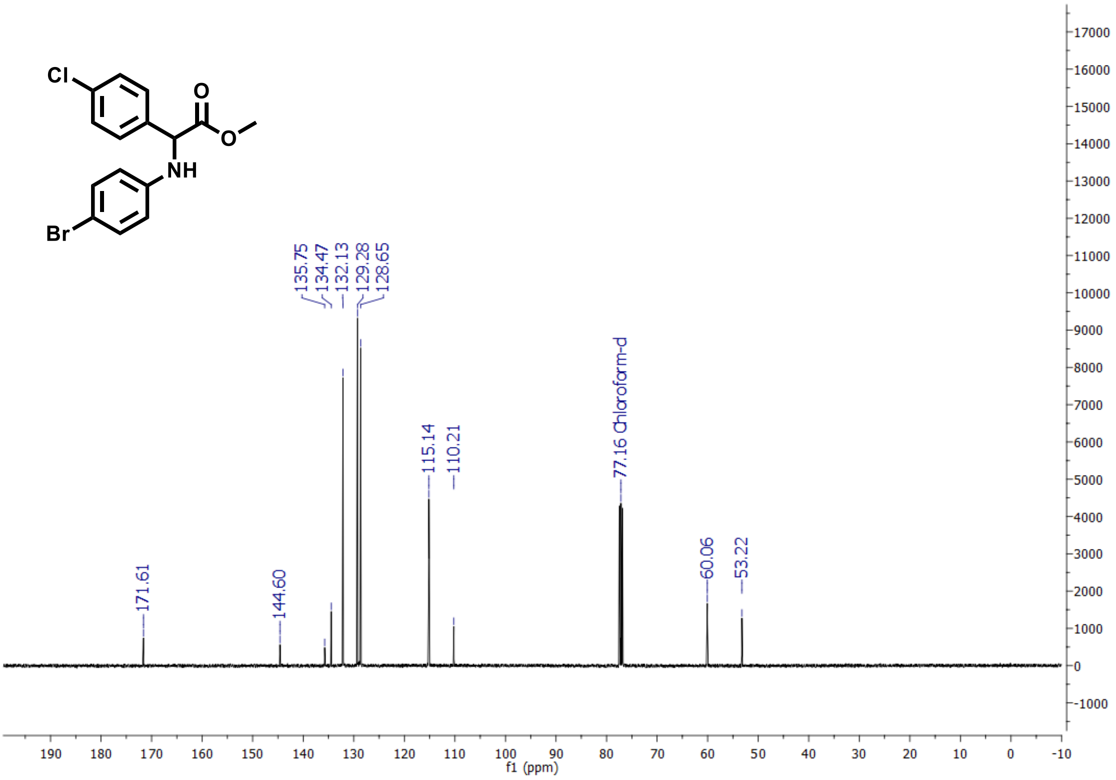

2-((4-chlorophenyl)amino)-2-phenylacetic acid (**40**) ^{1}H NMR (500 MHz, DMSO-d_6)

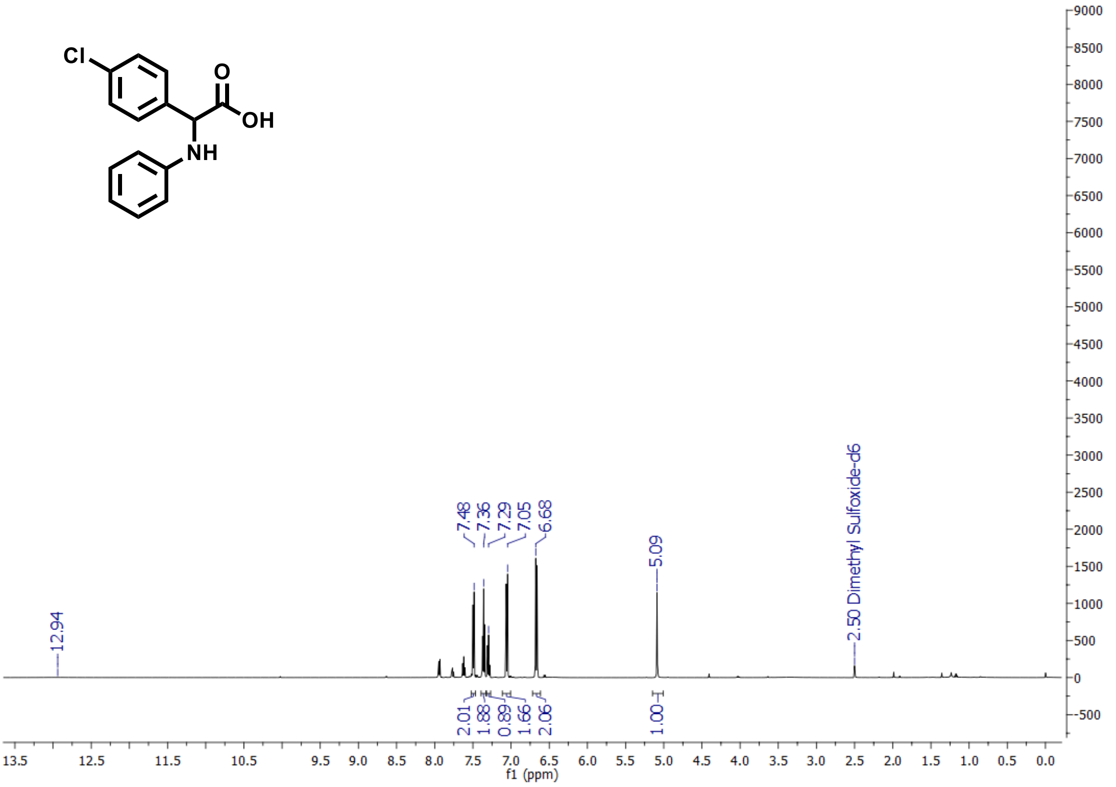

2-((4-chlorophenyl)amino)-2-phenylacetic acid (**40**) ^{1}H NMR (126 MHz, DMSO-d_6)

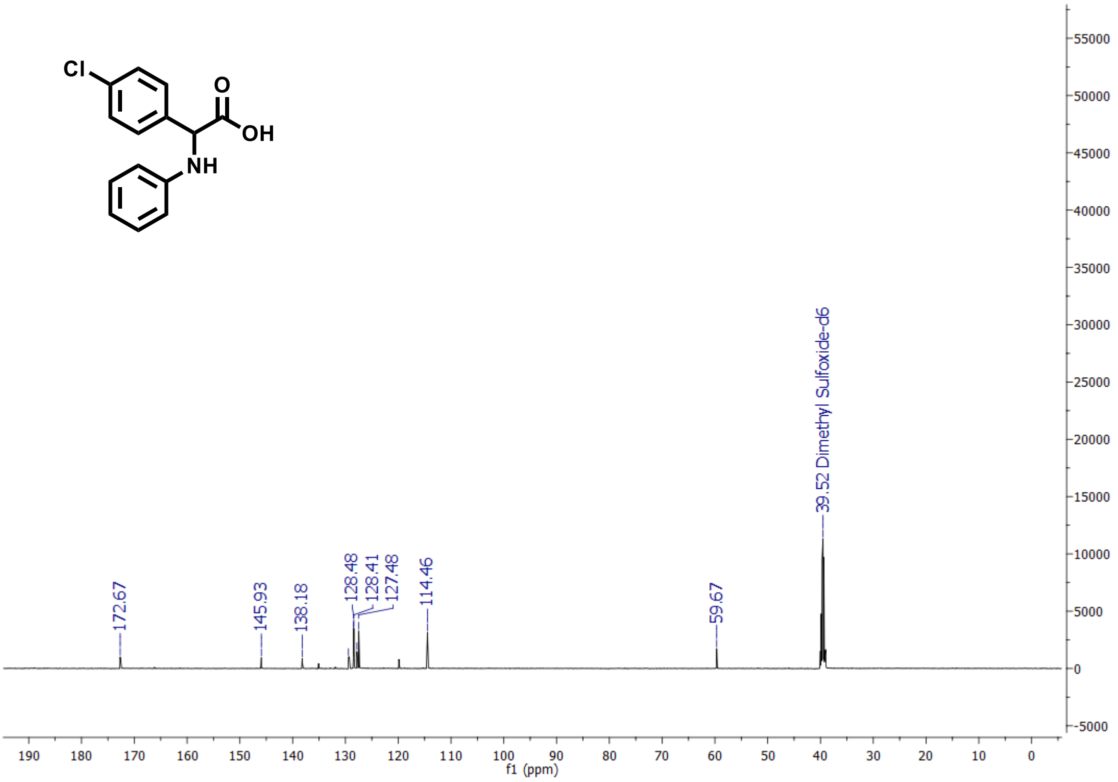

7. HPLC Chromatograms

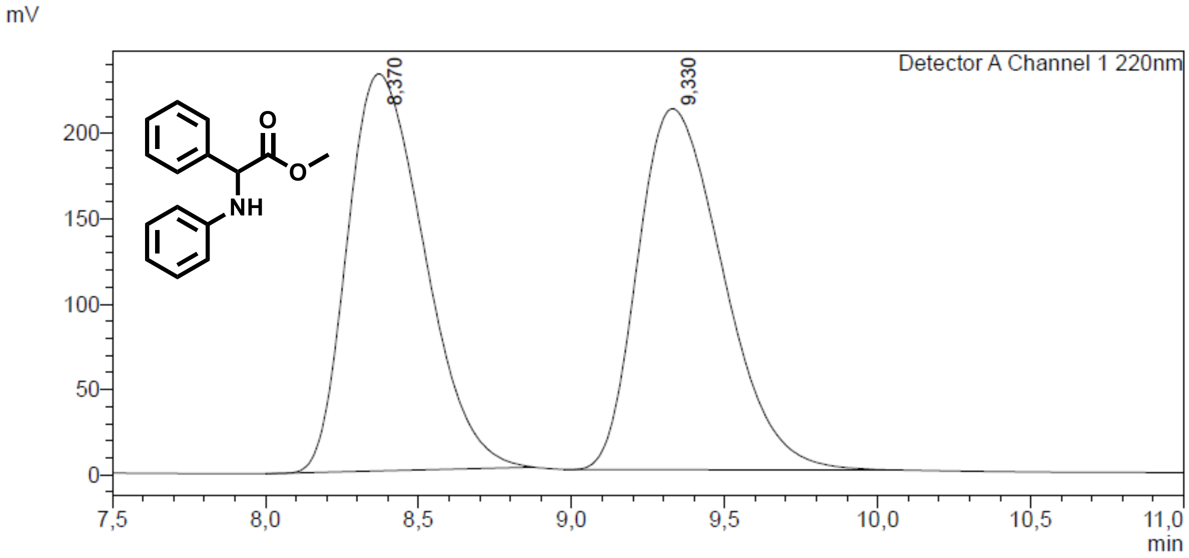

Peak#	Ret. Time	Area	Height	Conc.	Unit	Mark	Name
1	8,370	4060697	232226	49,770		M	
2	9,330	4098151	211142	50,230		M	
Total		8158848	443368				

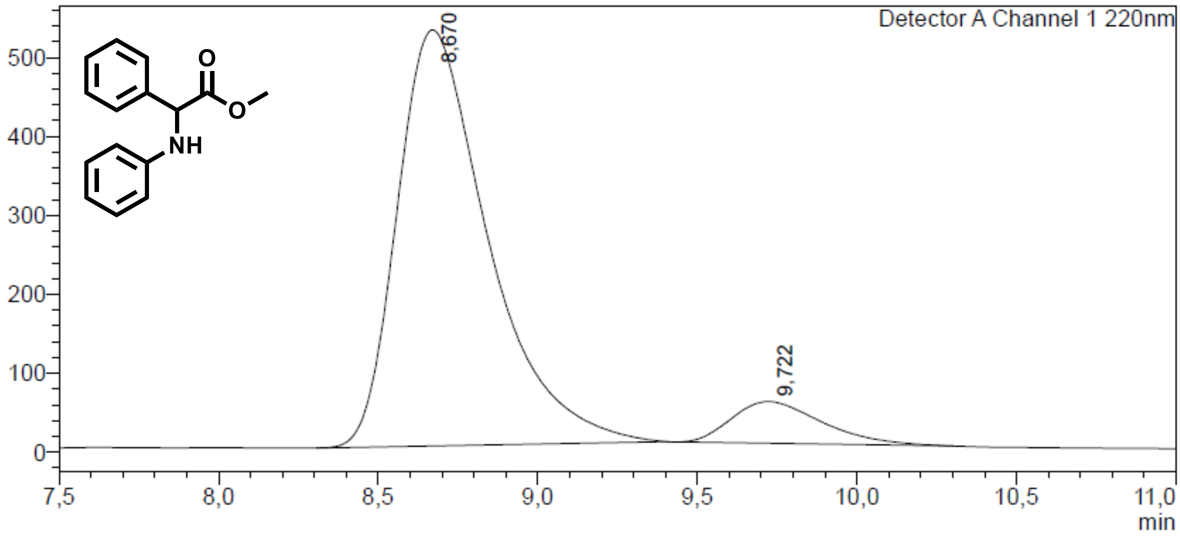

Peak#	Ret. Time	Area	Height	Conc.	Unit	Mark	Name
1	8,670	10444775	526896	90,967		M	
2	9,722	1037152	52587	9,033		M	
Total		11481927	579484				

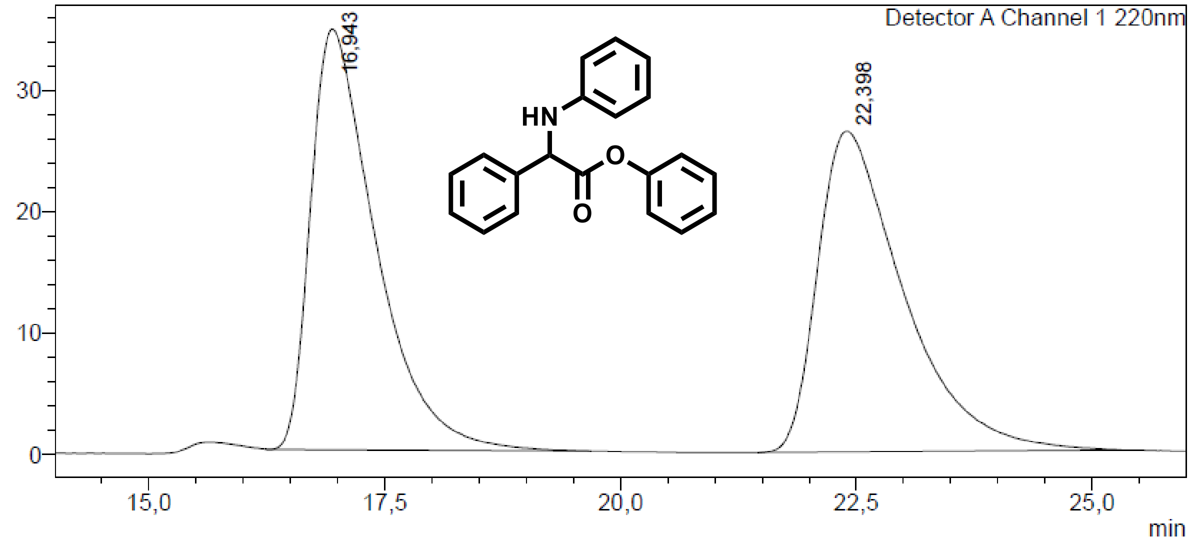

Peak#	Ret. Time	Area	Height	Conc.	Unit	Mark	Name
1	16,943	1664468	34620	49,842		M	
2	22,398	1675038	26400	50,158		M	
Total		3339506	61020				

,

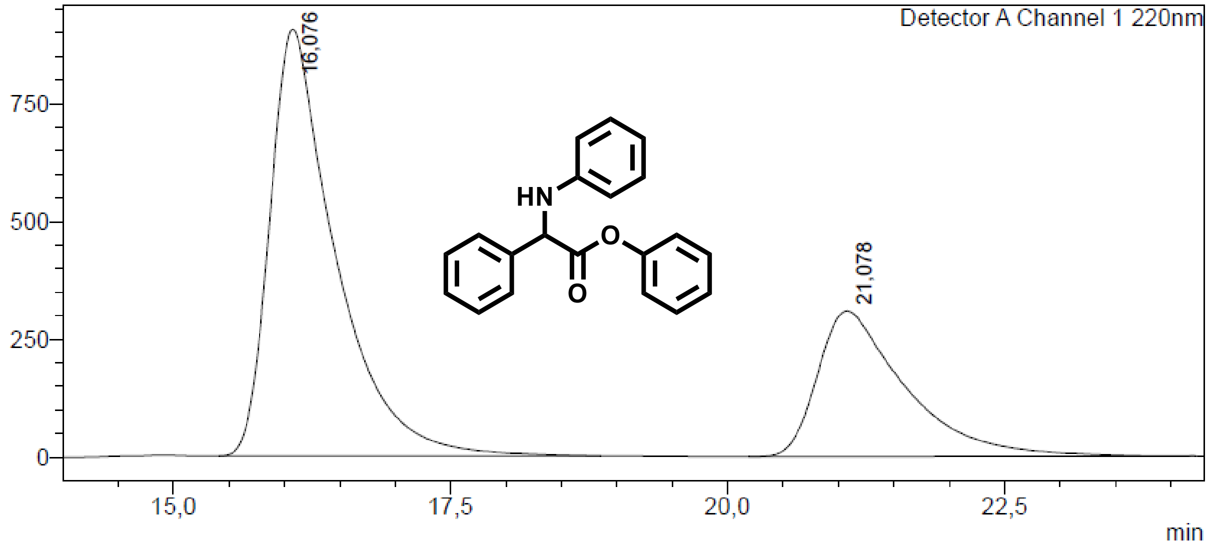

Peak#	Ret. Time	Area	Height	Conc.	Unit	Mark	Name
1	16,076	37396264	904860	68,568		M	
2	21,078	17142686	308933	31,432		M	
Total		54538950	1213793				

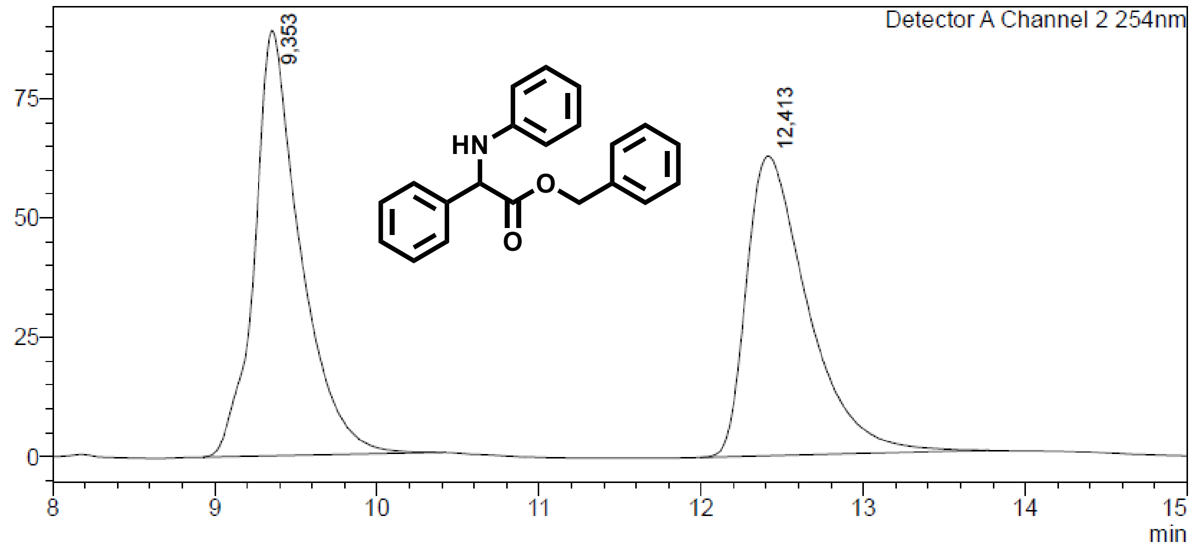

Detector A Channel 2 254nm

Peak#	Ret. Time	Area	Height	Conc.	Unit	Mark	Name
1	9,353	1760348	88939	51,493		M	
2	12,413	1658288	62732	48,507		M	
Total		3418636	151670				

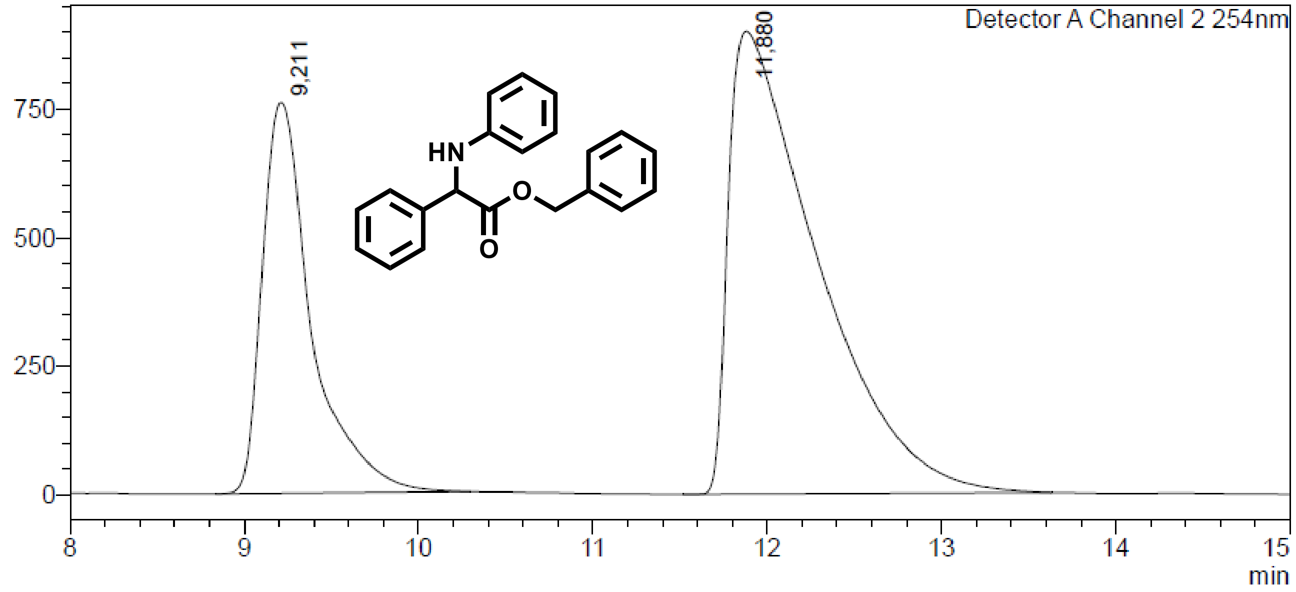

Detector A Channel 2 254nm

Peak#	Ret. Time	Area	Height	Conc.	Unit	Mark	Name
1	9,211	14871344	760920	31,804		M	
2	11,880	31887803	900295	68,196		M	
Total		46759147	1661215				

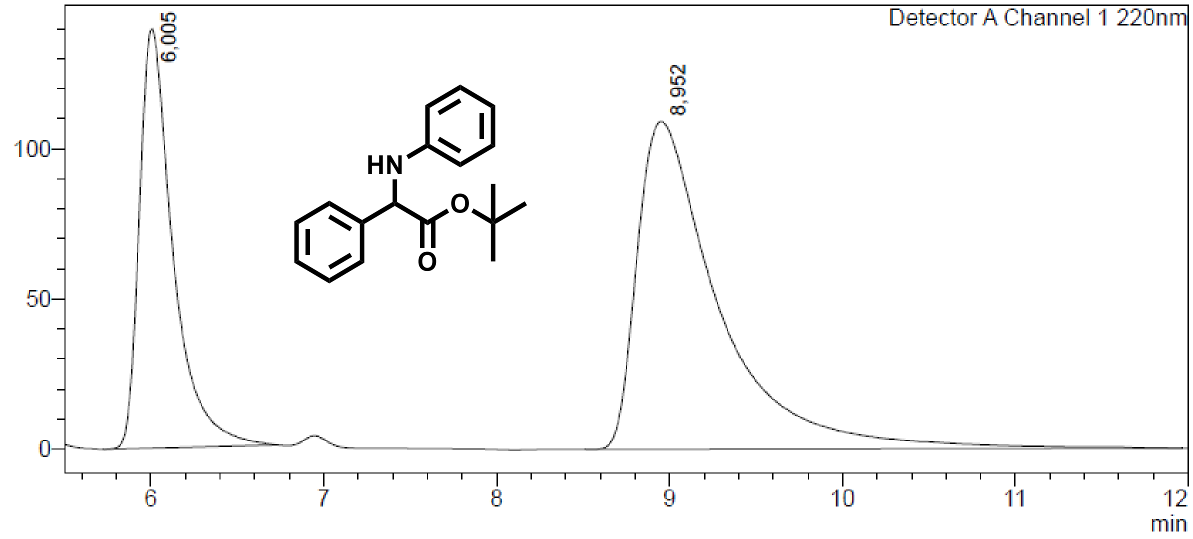

Peak#	Ret. Time	Area	Height	Conc.	Unit	Mark	Name
1	5,936	33134292	2301707	49,984		M	
2	8,827	33154943	920590	50,016		M	
Total		66289235	3222297				

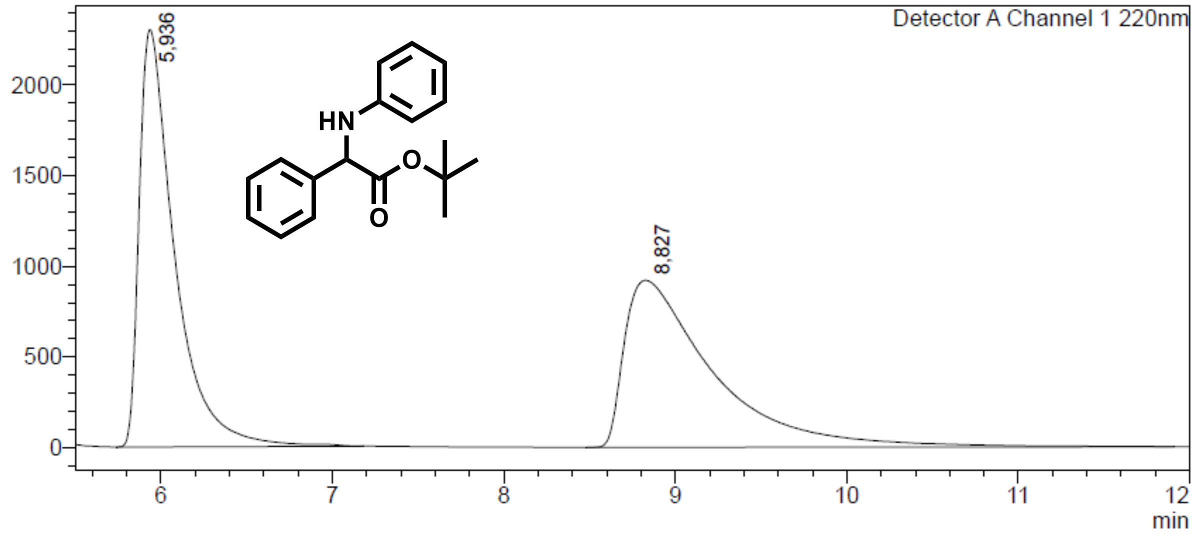

Peak#	Ret. Time	Area	Height	Conc.	Unit	Mark	Name
1	6,005	1889792	139620	34,370		M	
2	8,952	3608540	109220	65,630		M	
Total		5498332	248840				

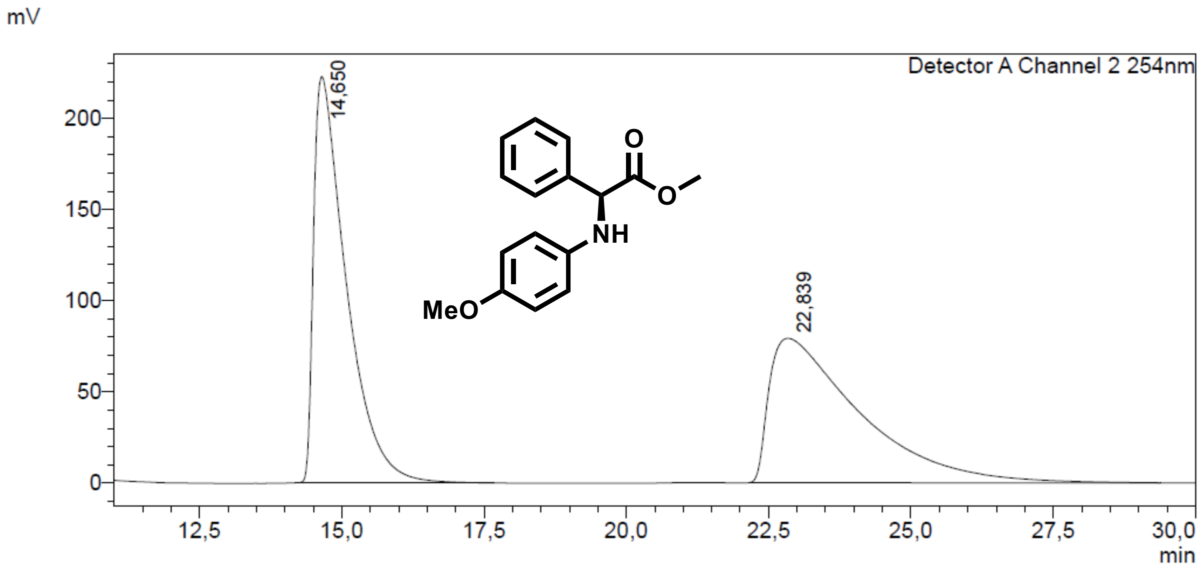

Detector A Channel 2 254nm

Peak#	Ret. Time	Area	Height	Conc.	Unit	Mark	Name
1	14,650	8896887	222932	50,401		M	
2	22,839	8755229	79189	49,599		M	
Total		17652116	302120				

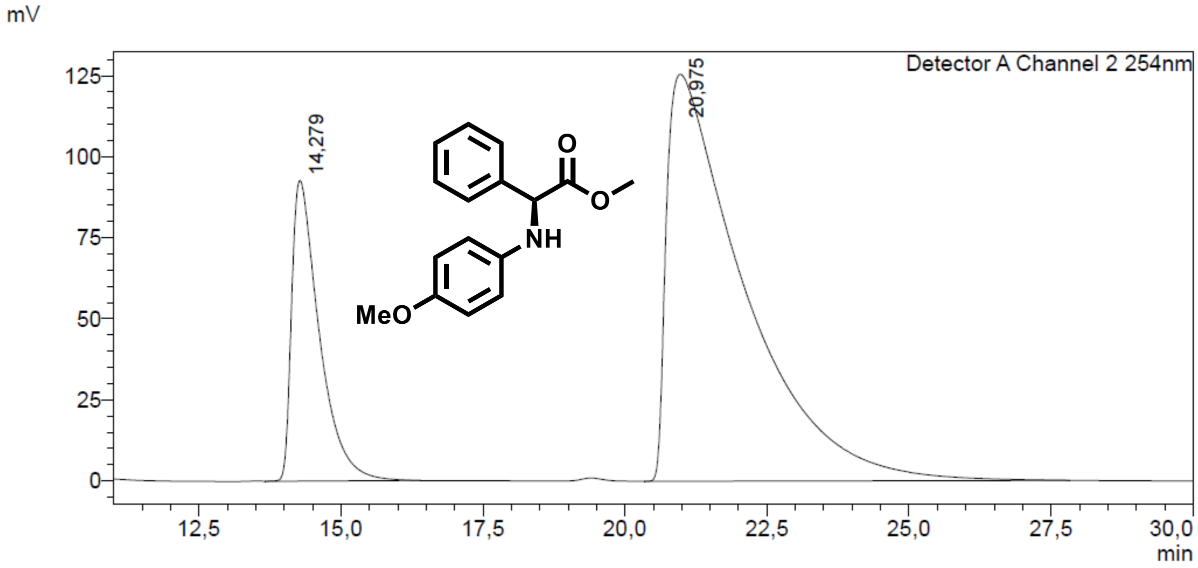

Detector A Channel 2 254nm

Peak#	Ret. Time	Area	Height	Conc.	Unit	Mark	Name
1	14,279	3152445	92729	20,864		M	
2	20,975	11957171	125523	79,136		M	
Total		15109616	218253				

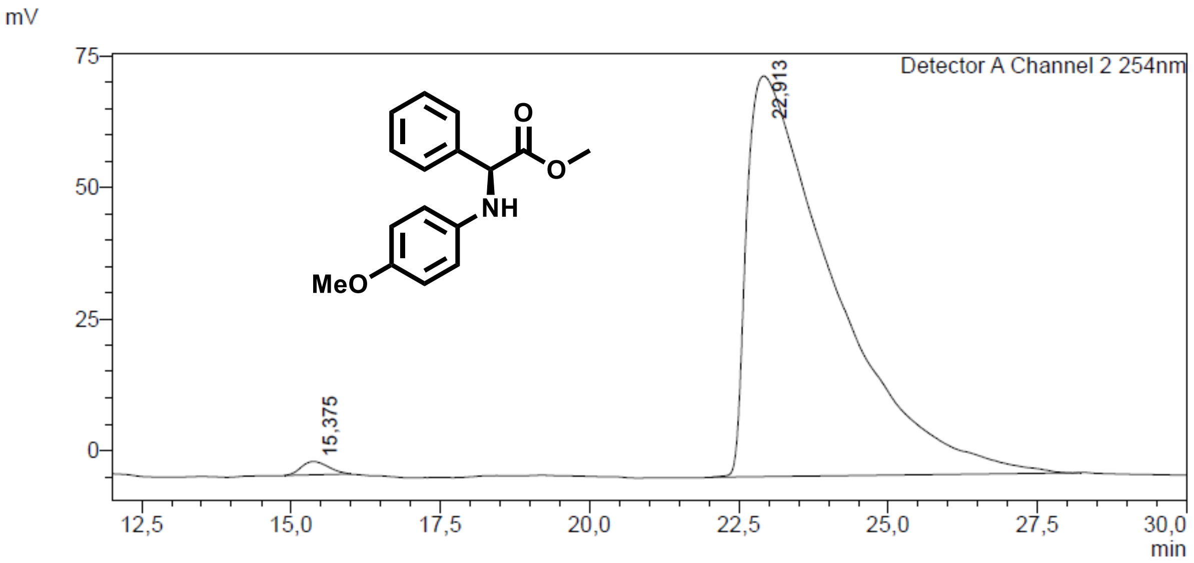

Detector A Channel 2 254nm

Peak#	Ret. Time	Area	Height	Conc.	Unit	Mark	Name
1	15,375	81016	2542	1,037		M	
2	22,913	7729073	76124	98,963		M	
Total		7810089	78666				

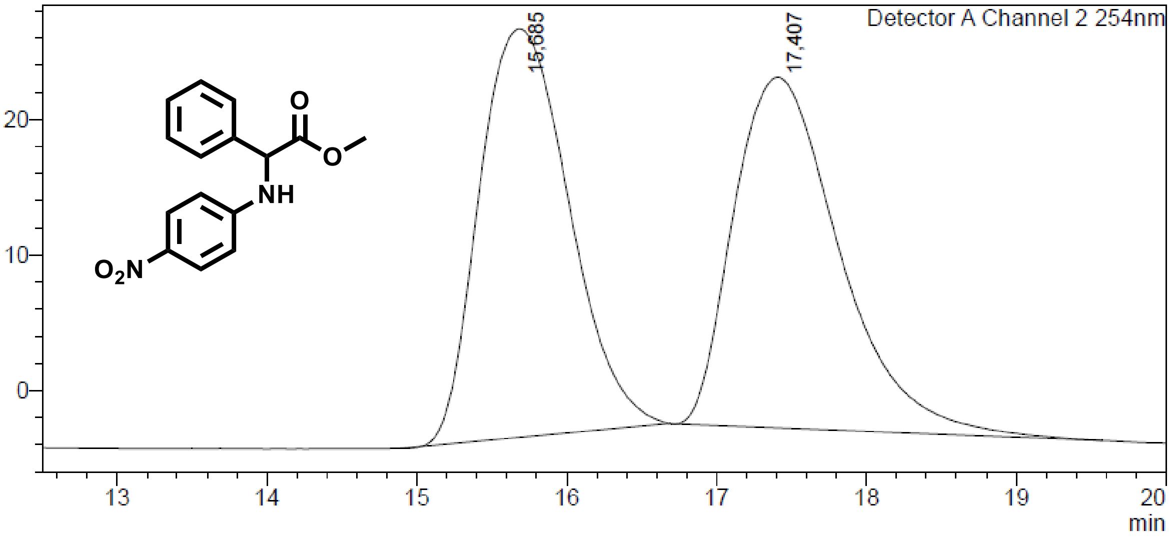

Detector A Channel 2 254nm

Peak#	Ret. Time	Area	Height	Conc.	Unit	Mark	Name
1	15,685	1257284	30116	49,691		M	
2	17,407	1272925	25852	50,309		M	
Total		2530209	55968				

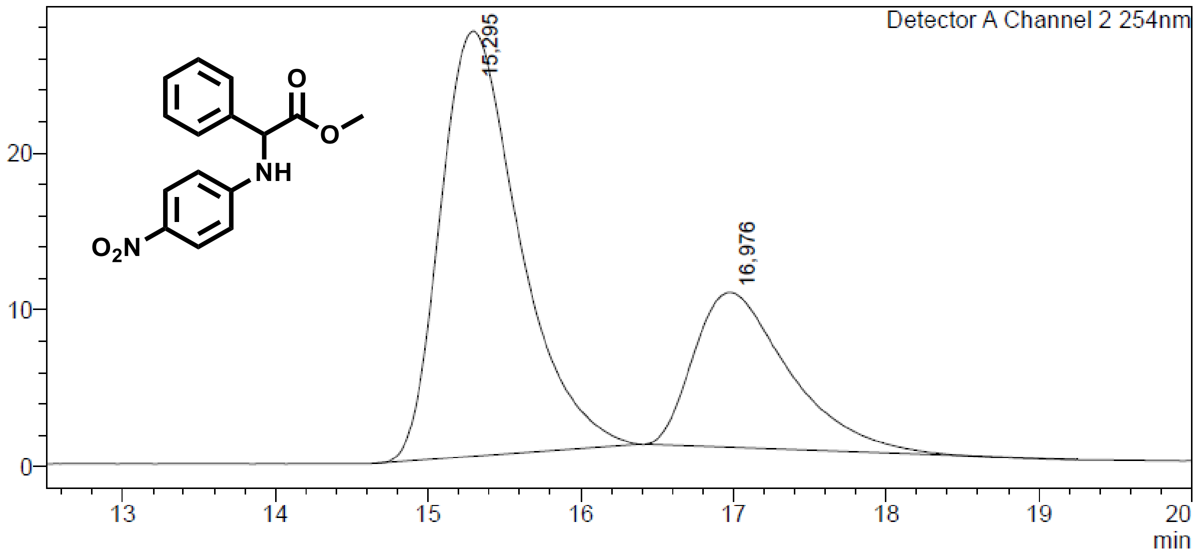

Detector A Channel 2 254nm

Peak#	Ret. Time	Area	Height	Conc.	Unit	Mark	Name
1	15,295	974049	27107	69,400		M	
2	16,976	429470	9880	30,600		M	
Total		1403519	36986				

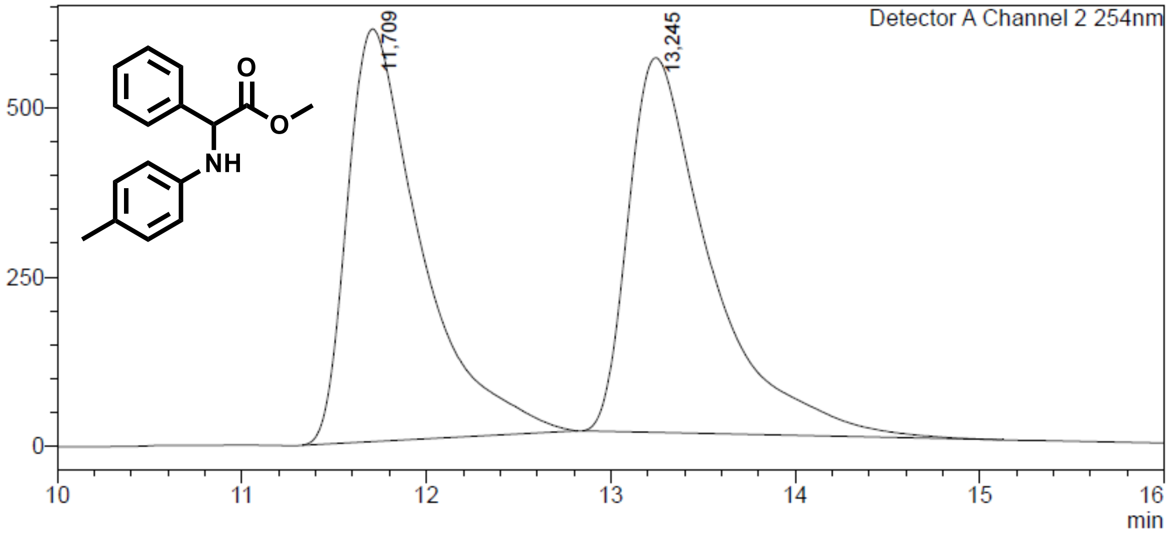

Detector A Channel 2 254nm

Peak#	Ret. Time	Area	Height	Conc.	Unit	Mark	Name
1	11,709	16963708	609870	49,601		M	
2	13,245	17236854	553662	50,399		M	
Total		34200562	1163532				

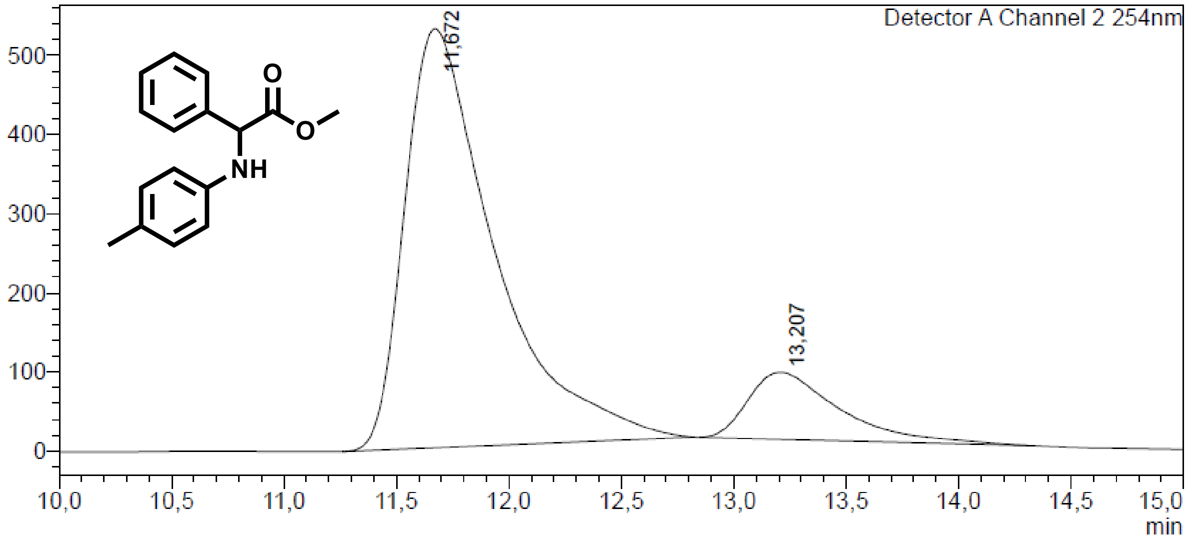

Detector A Channel 2 254nm

Peak#	Ret. Time	Area	Height	Conc.	Unit	Mark	Name
1	11,672	14727440	529283	86,058		M	
2	13,207	2385863	84852	13,942		M	
Total		17113303	614135				

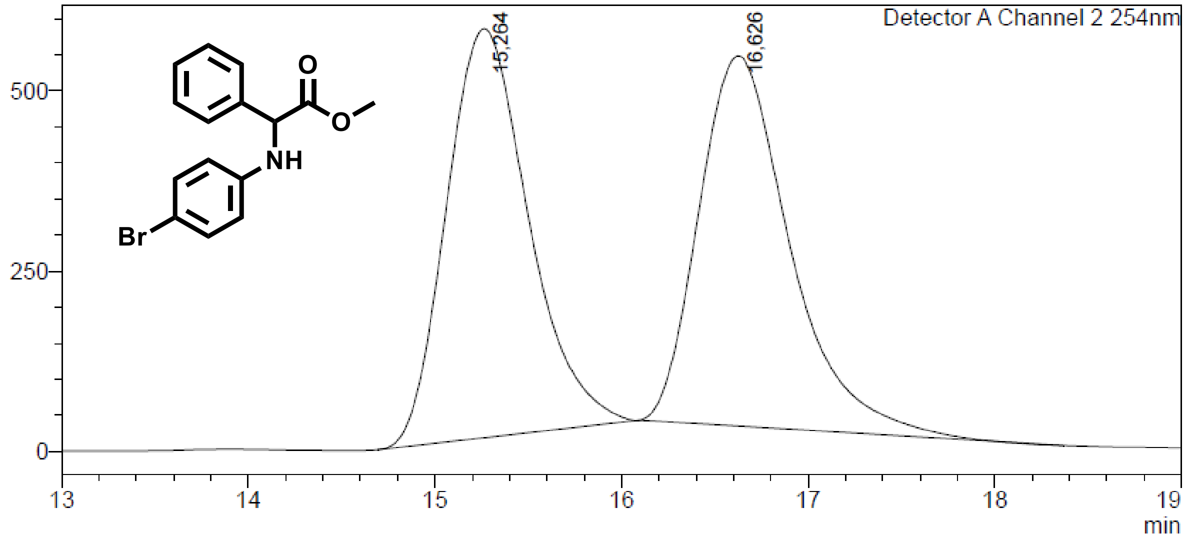

Detector A Channel 2 254nm

Peak#	Ret. Time	Area	Height	Conc.	Unit	Mark	Name
1	15,264	17363738	567166	49,910		M	
2	16,626	17426636	513528	50,090		M	
Total		34790374	1080693				

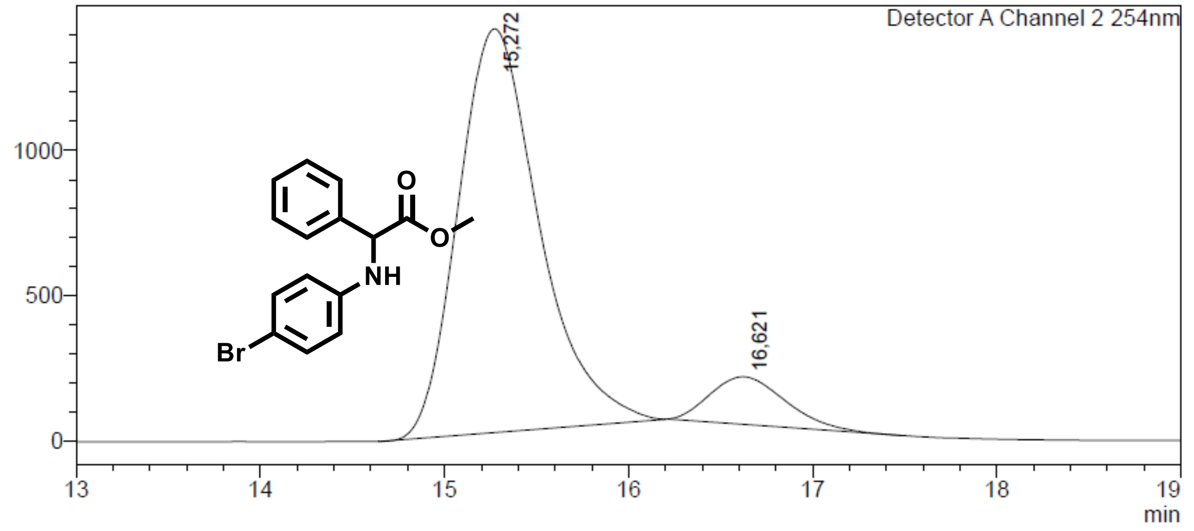

Detector A Channel 2 254nm

Peak#	Ret. Time	Area	Height	Conc.	Unit	Mark	Name
1	15,272	42521434	1387774	90,093		M	
2	16,621	4675814	163200	9,907		M	
Total		47197247	1550974				

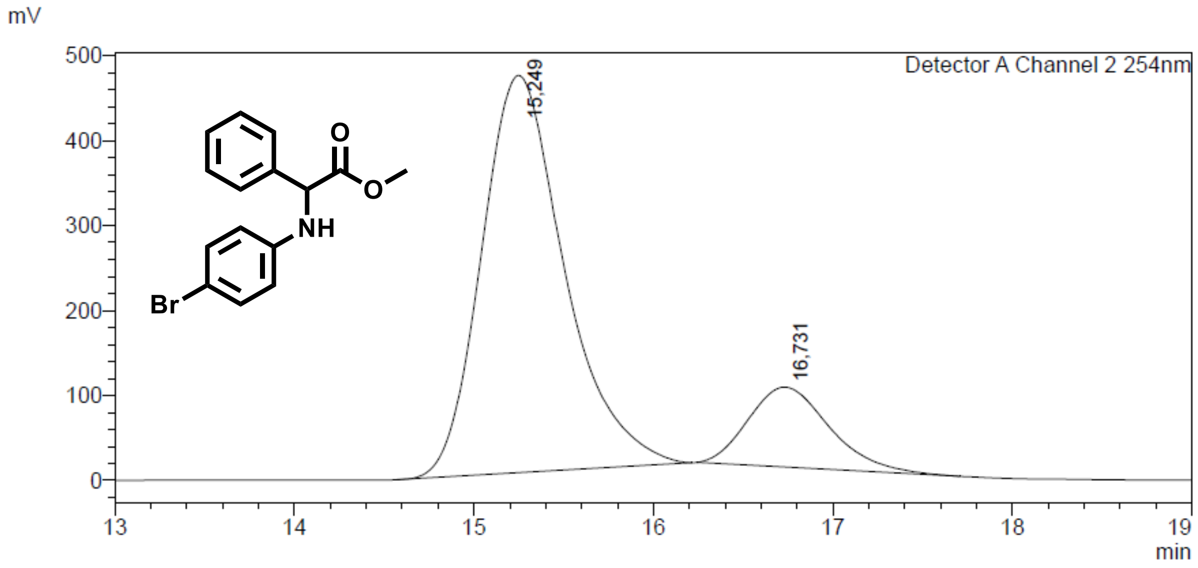

Detector A Channel 2 254nm

Peak#	Ret. Time	Area	Height	Conc.	Unit	Mark	Name
1	15,249	15013704	467415	83,543		M	
2	16,731	2957438	93961	16,457		M	
Total		17971142	561375				

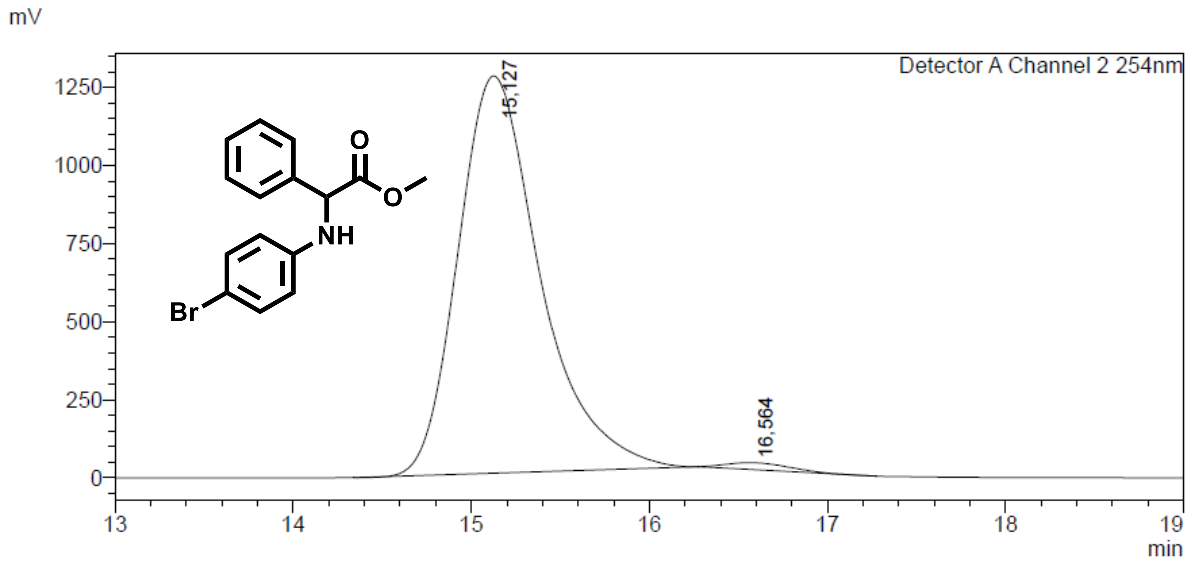

Detector A Channel 2 254nm

Peak#	Ret. Time	Area	Height	Conc.	Unit	Mark	Name
1	15,127	40835472	1271474	98,733		M	
2	16,564	523898	21280	1,267		M	
Total		41359370	1292754				

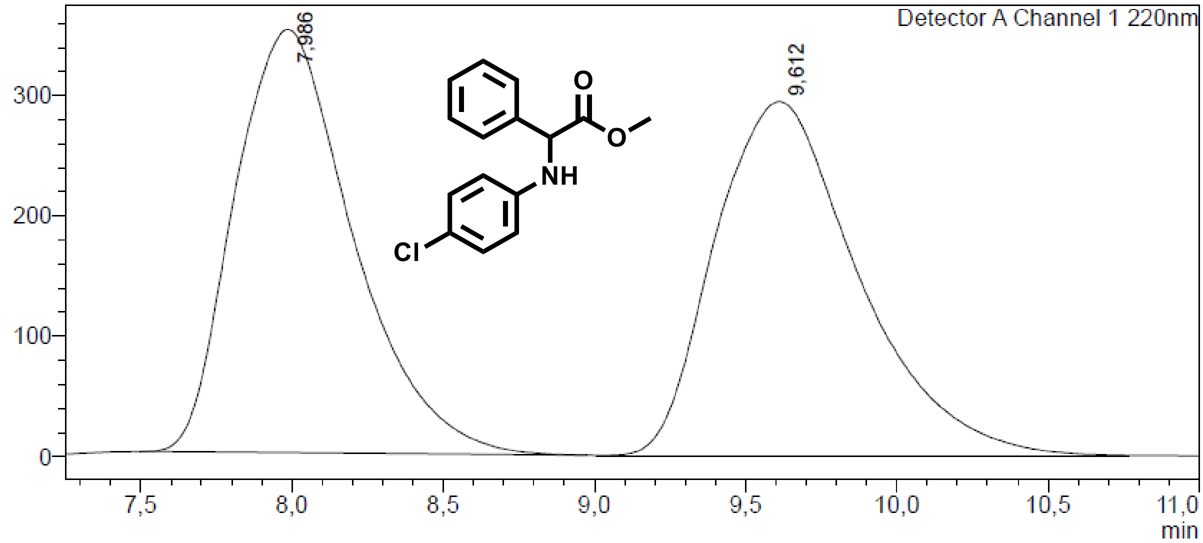

Peak#	Ret. Time	Area	Height	Conc.	Unit	Mark	Name
1	7,986	9599671	351724	49,700		M	
2	9,612	9715397	294056	50,300		M	
Total		19315069	645781				

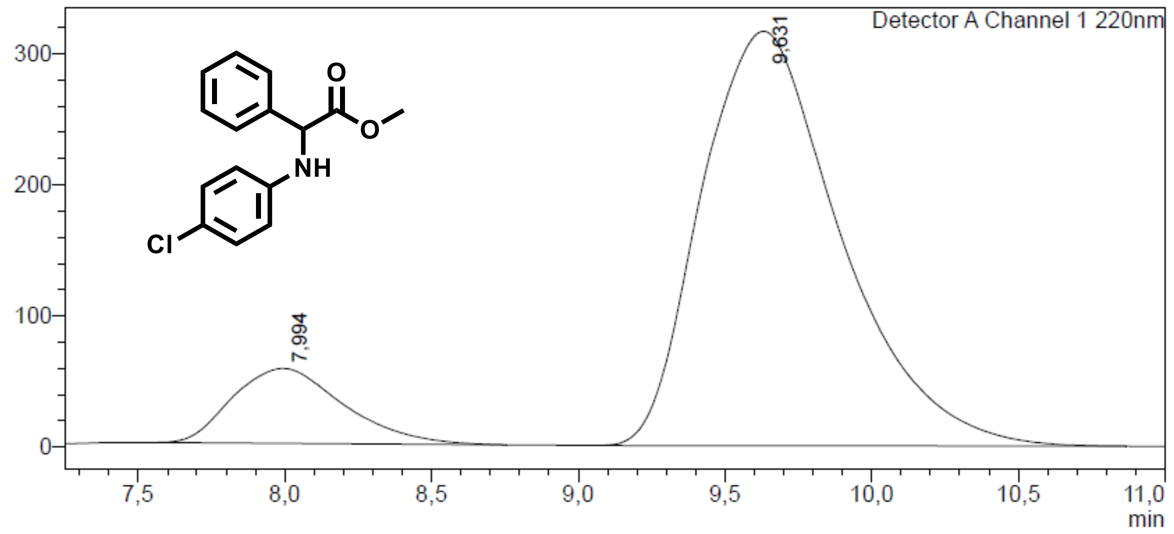

Peak#	Ret. Time	Area	Height	Conc.	Unit	Mark	Name
1	7,994	1500789	57092	12,553		M	
2	9,631	10454800	316059	87,447		M	
Total		11955589	373150				

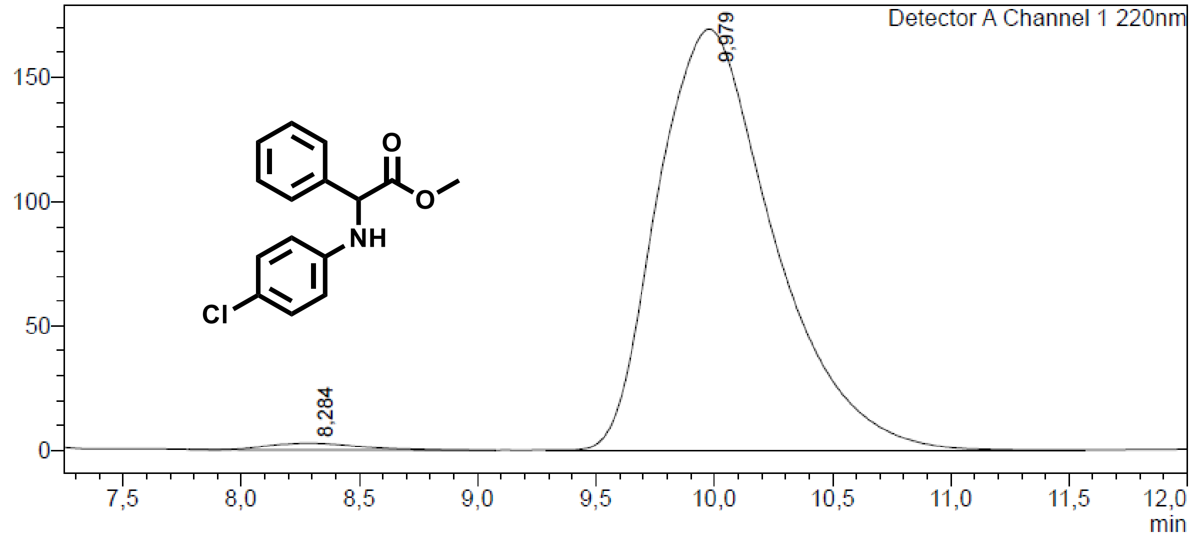

Peak#	Ret. Time	Area	Height	Conc.	Unit	Mark	Name
1	8,284	69442	2678	1,186		M	
2	9,979	5785629	169328	98,814		M	
Total		5855071	172006				

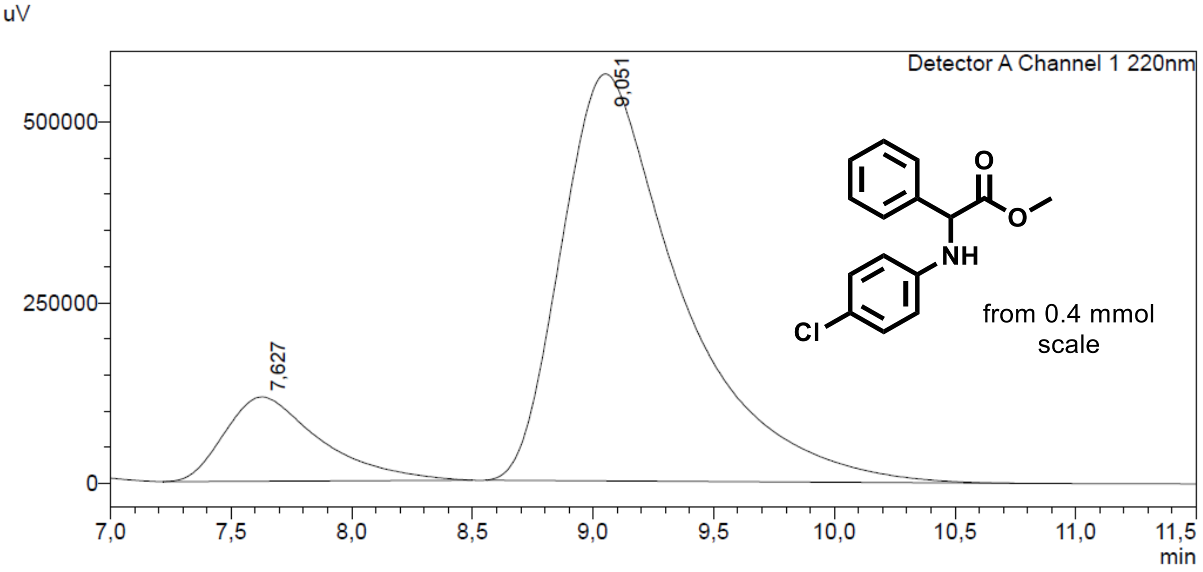

Peak#	Ret. Time	Area	Height	Conc.	Unit	Mark	Name
1	7,627	3209634	116457	14,163		M	
2	9,051	19452990	562406	85,837		M	
Total		22662623	678862				

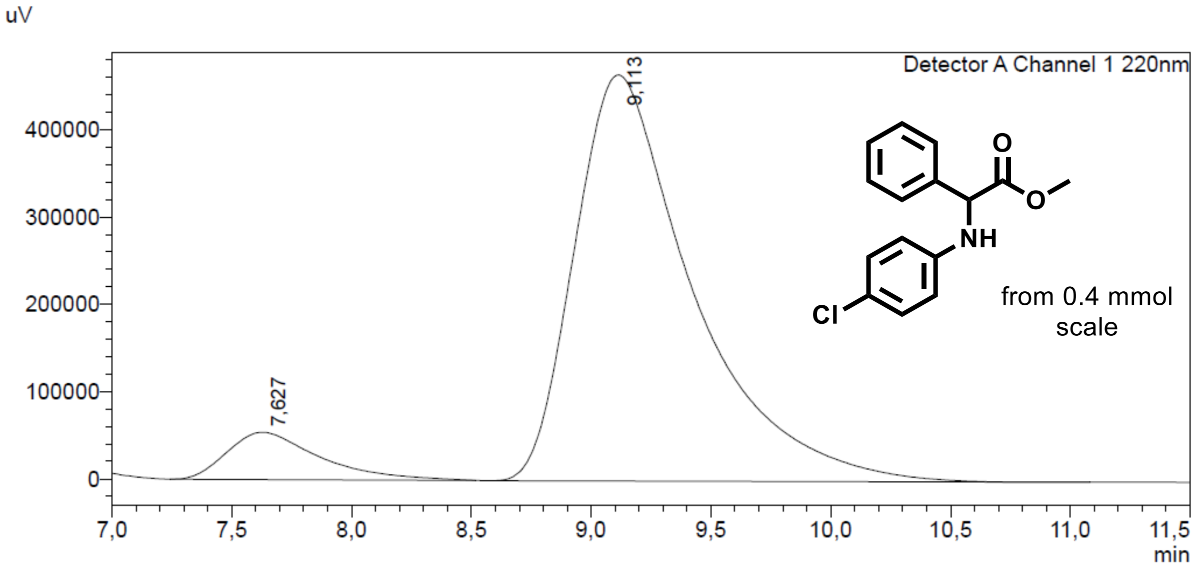

Peak#	Ret. Time	Area	Height	Conc.	Unit	Mark	Name
1	7,627	1450193	54190	8,315		M	
2	9,113	15990112	465404	91,685		M	
Total		17440306	519595				

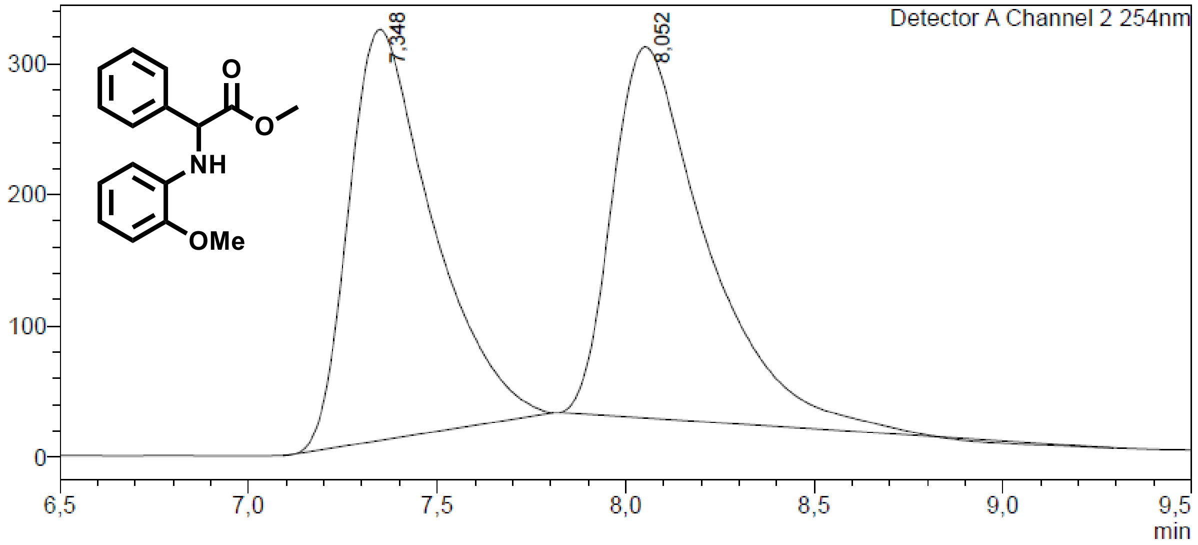

Detector A Channel 2 254nm

Peak#	Ret. Time	Area	Height	Conc.	Unit	Mark	Name
1	7,348	4871139	313142	49,591		M	
2	8,052	4951440	282904	50,409		M	
Total		9822579	596046				

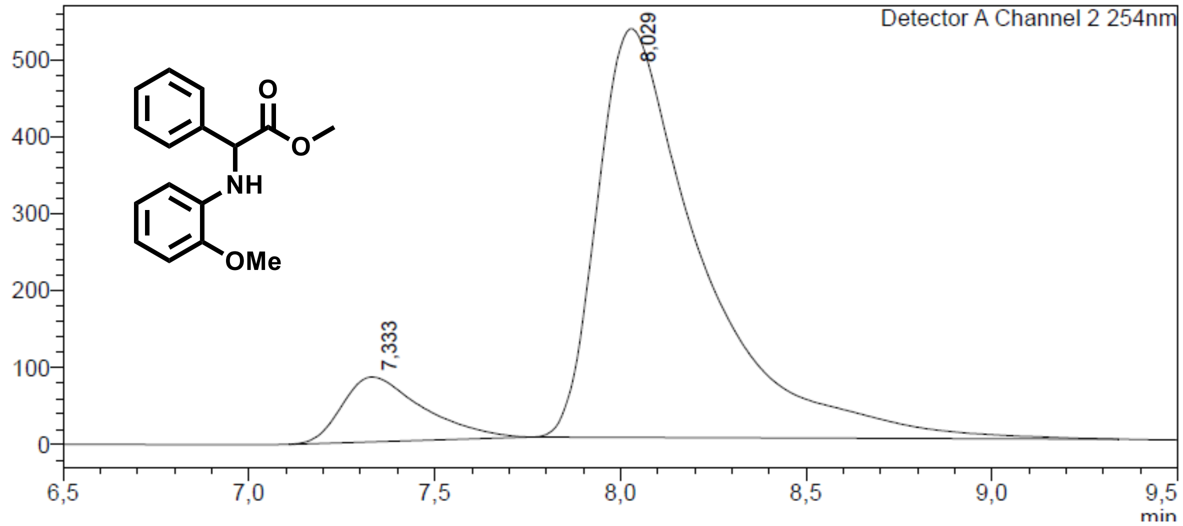

Detector A Channel 2 254nm

Peak#	Ret. Time	Area	Height	Conc.	Unit	Mark	Name
1	7,333	1270789	84259	10,886		M	
2	8,029	10402723	530463	89,114		M	
Total		11673511	614723				

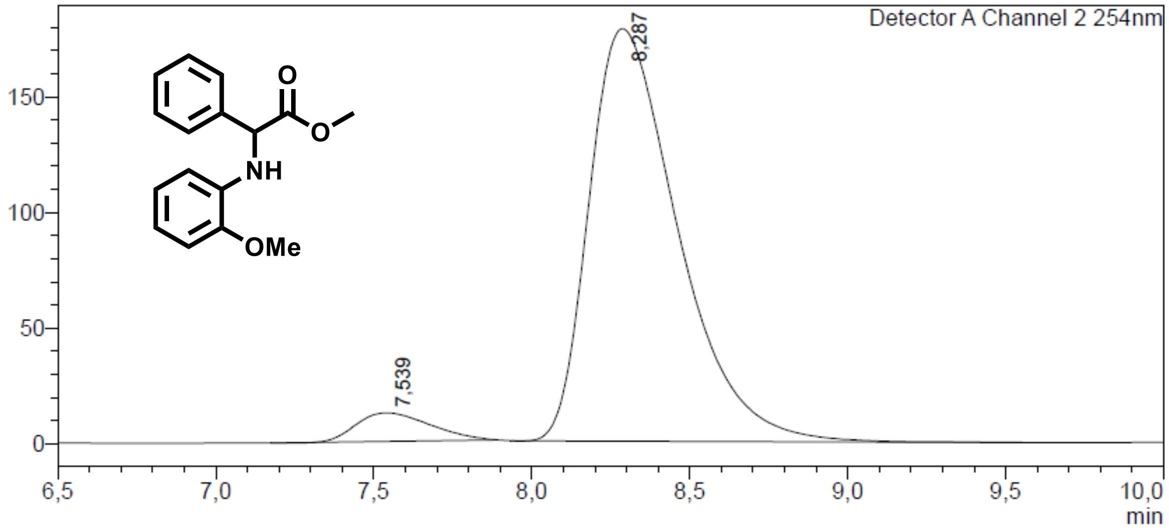

Detector A Channel 2 254nm

Peak#	Ret. Time	Area	Height	Conc.	Unit	Mark	Name
1	7,539	201705	12372	5,469		M	
2	8,287	3486527	178315	94,531		M	
Total		3688233	190687				

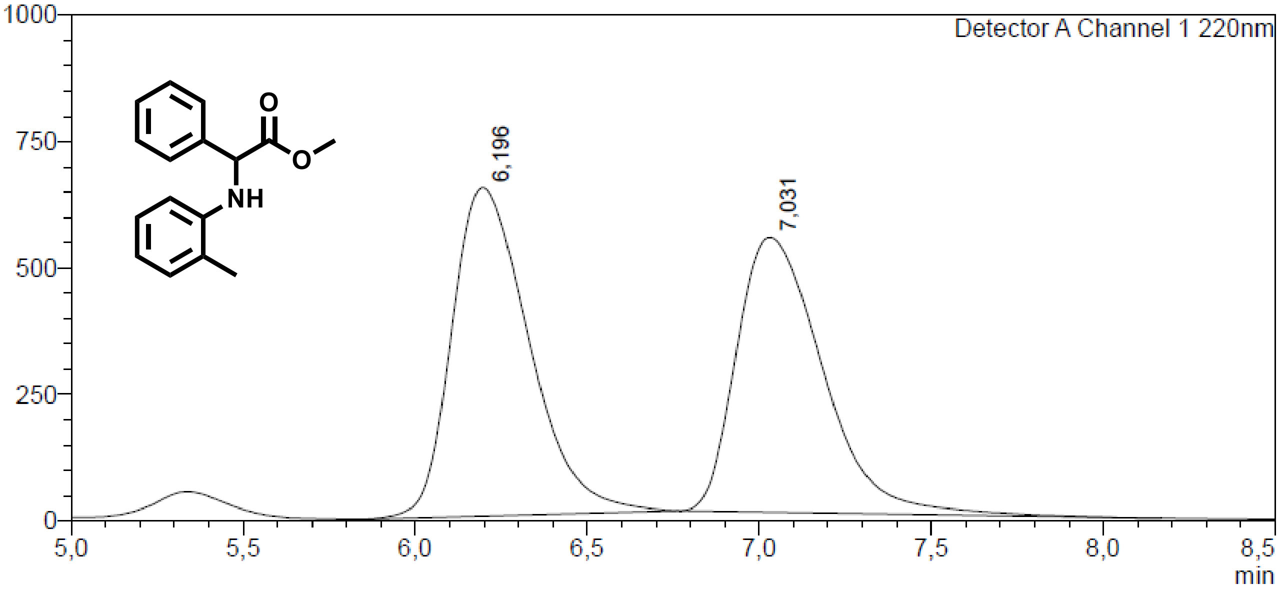

Peak#	Ret. Time	Area	Height	Conc.	Unit	Mark	Name
1	6,196	10159712	648896	51,731		M	
2	7,031	9479830	542918	48,269		M	
Total		19639542	1191814				

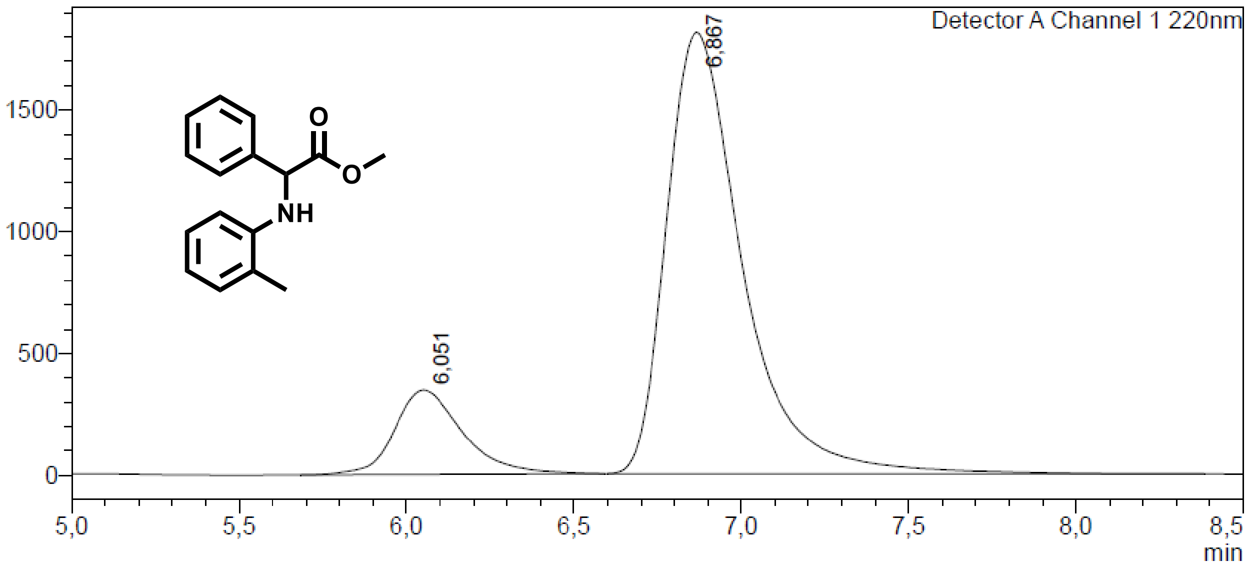

Peak#	Ret. Time	Area	Height	Conc.	Unit	Mark	Name
1	6,051	4895612	344952	14,592		M	
2	6,867	28655374	1811282	85,408		M	
Total		33550985	2156234				

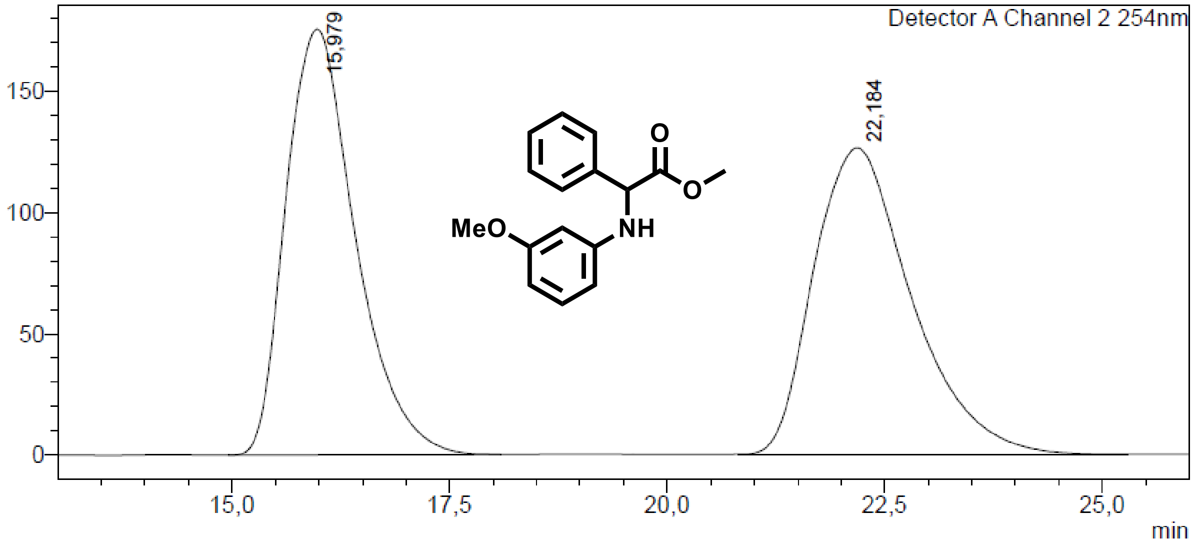

Detector A Channel 2 254nm

Peak#	Ret. Time	Area	Height	Conc.	Unit	Mark	Name
1	15,979	9778536	175673	49,756		M	
2	22,184	9874559	126353	50,244		M	
Total		19653095	302025				

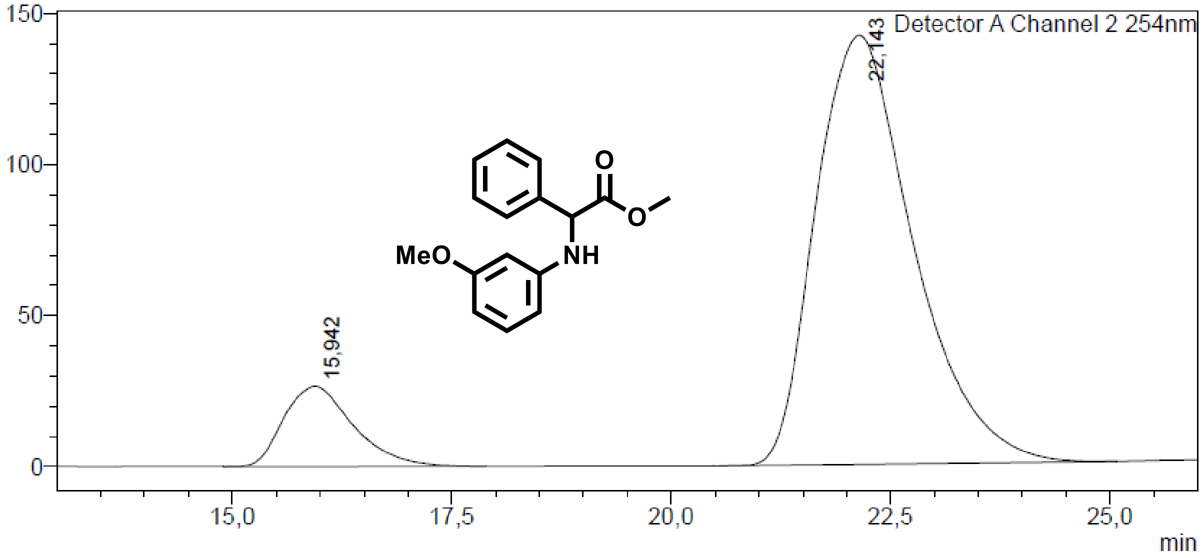

Detector A Channel 2 254nm

Peak#	Ret. Time	Area	Height	Conc.	Unit	Mark	Name
1	15,942	1462092	26519	11,726		M	
2	22,143	11007019	142013	88,274		M	
Total		12469111	168531				

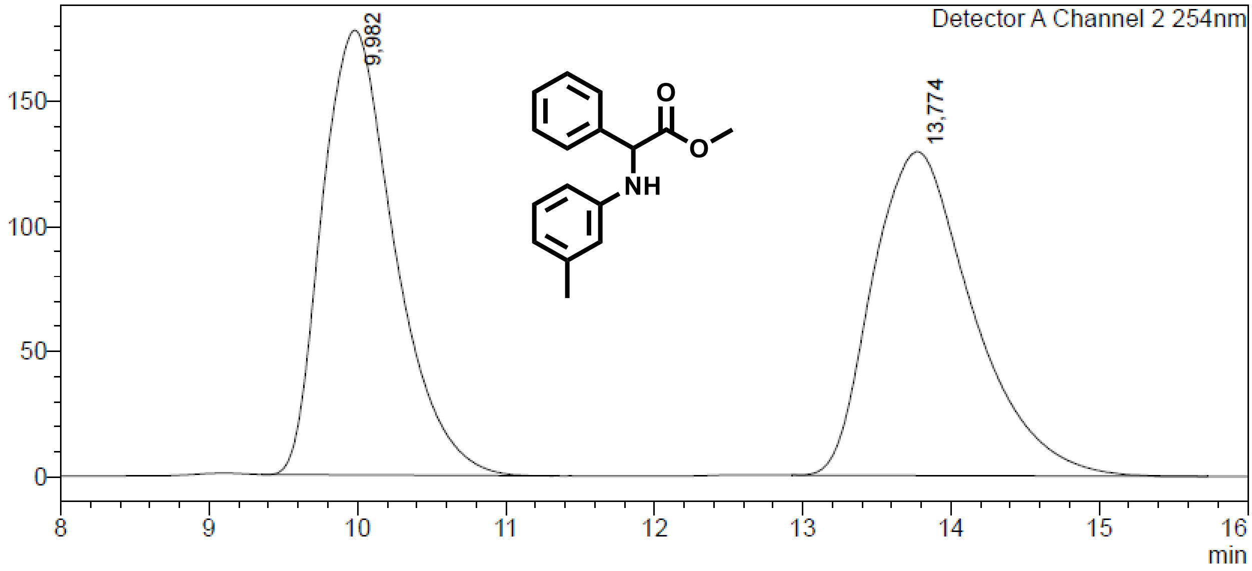

Detector A Channel 2 254nm

Peak#	Ret. Time	Area	Height	Conc.	Unit	Mark	Name
1	9,982	6051285	177266	49,868		M	
2	13,774	6083297	129111	50,132		M	
Total		12134582	306377				

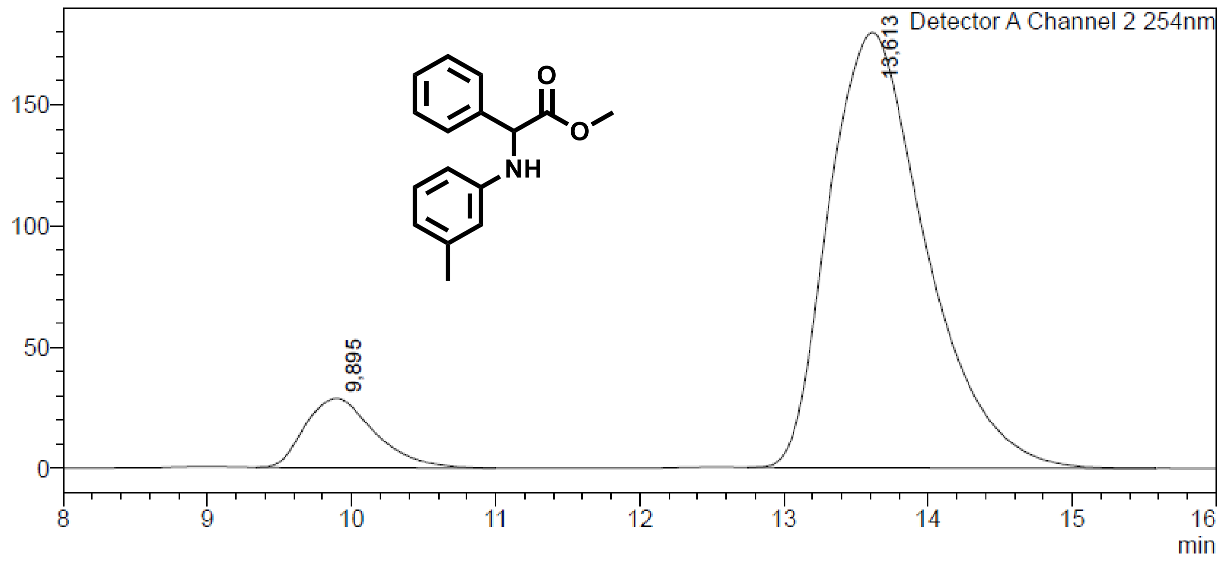

Detector A Channel 2 254nm

Peak#	Ret. Time	Area	Height	Conc.	Unit	Mark	Name
1	9,895	960617	28703	10,254		M	
2	13,613	8407283	179457	89,746		M	
Total		9367901	208160				

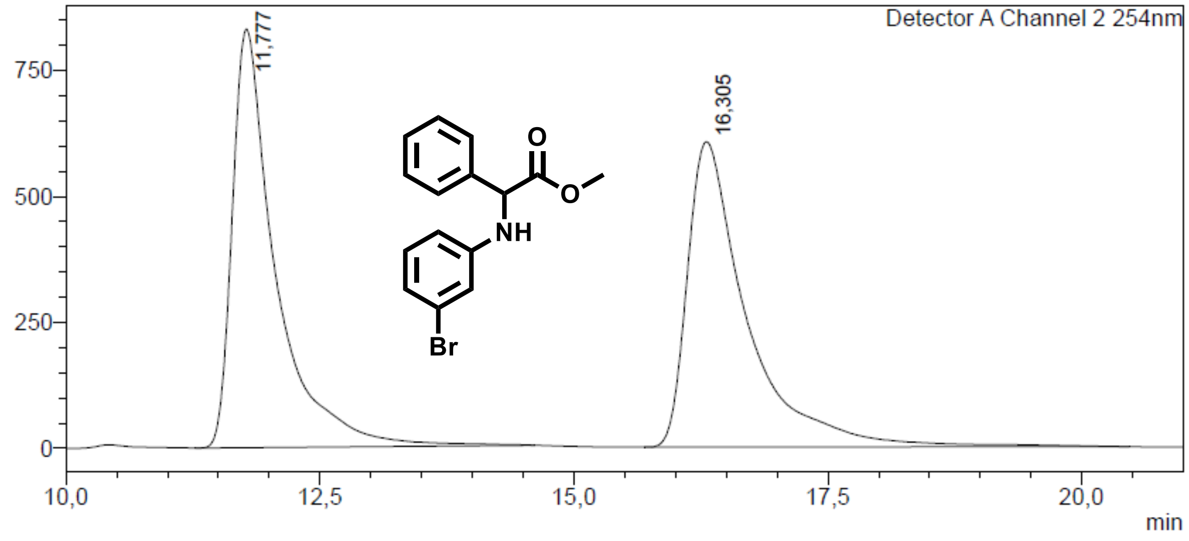

Detector A Channel 2 254nm

Peak#	Ret. Time	Area	Height	Conc.	Unit	Mark	Name
1	11,777	24706651	830770	49,838		M	
2	16,305	24867314	606535	50,162		M	
Total		49573965	1437305				

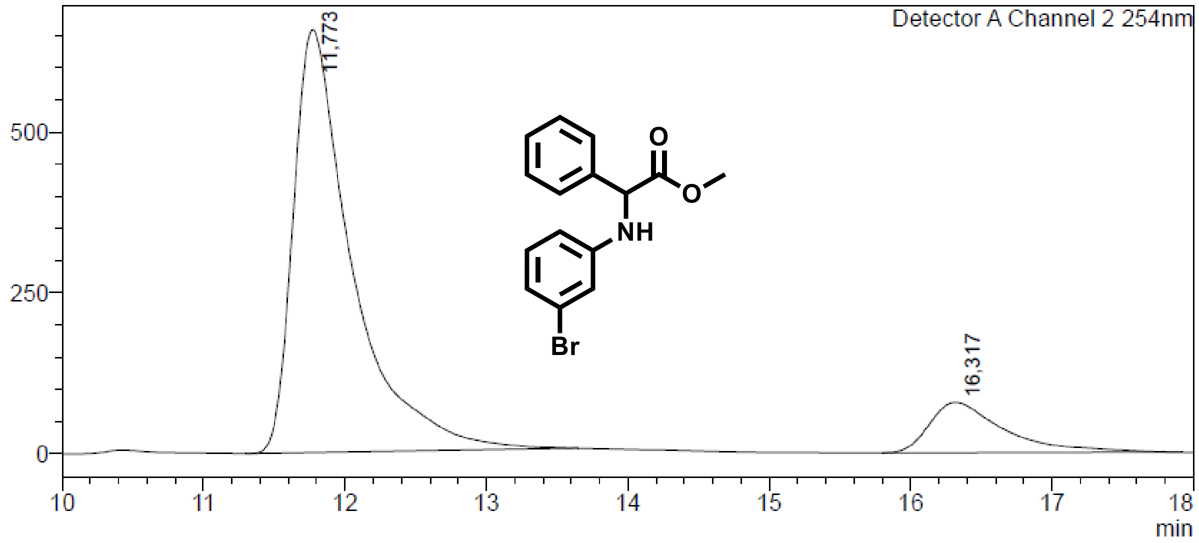

Detector A Channel 2 254nm

Peak#	Ret. Time	Area	Height	Conc.	Unit	Mark	Name
1	11,773	19059174	657582	86,860		M	
2	16,317	2883186	78167	13,140		M	
Total		21942361	735748				

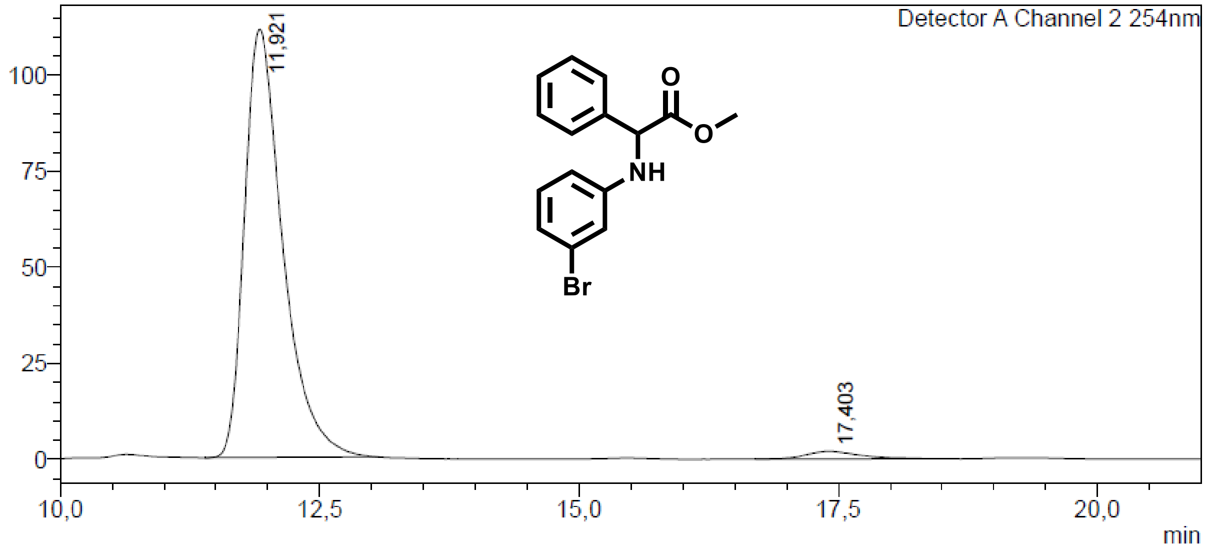

Detector A Channel 2 254nm

Peak#	Ret. Time	Area	Height	Conc.	Unit	Mark	Name
1	11,921	2875592	111498	97,601		M	
2	17,403	70681	2004	2,399		M	
Total		2946274	113502				

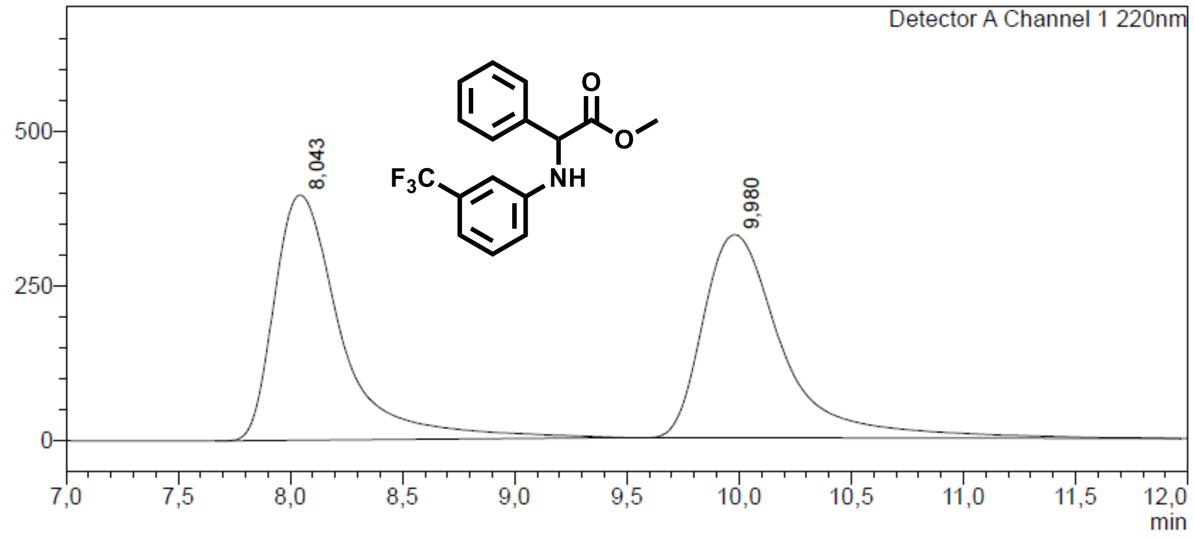

Peak#	Ret. Time	Area	Height	Conc.	Unit	Mark	Name
1	8,043	8264411	395734	50,313		M	
2	9,980	8161483	327792	49,687		M	
Total		16425894	723526				

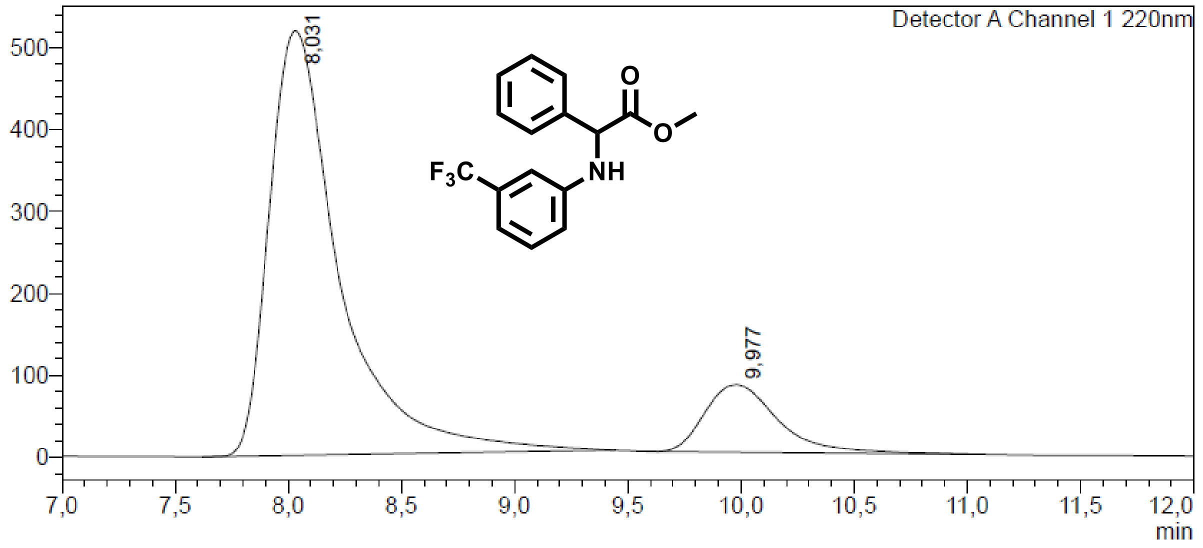

Peak#	Ret. Time	Area	Height	Conc.	Unit	Mark	Name
1	8,031	11266706	518547	85,890		M	
2	9,977	1850931	82553	14,110		M	
Total		13117637	601100				

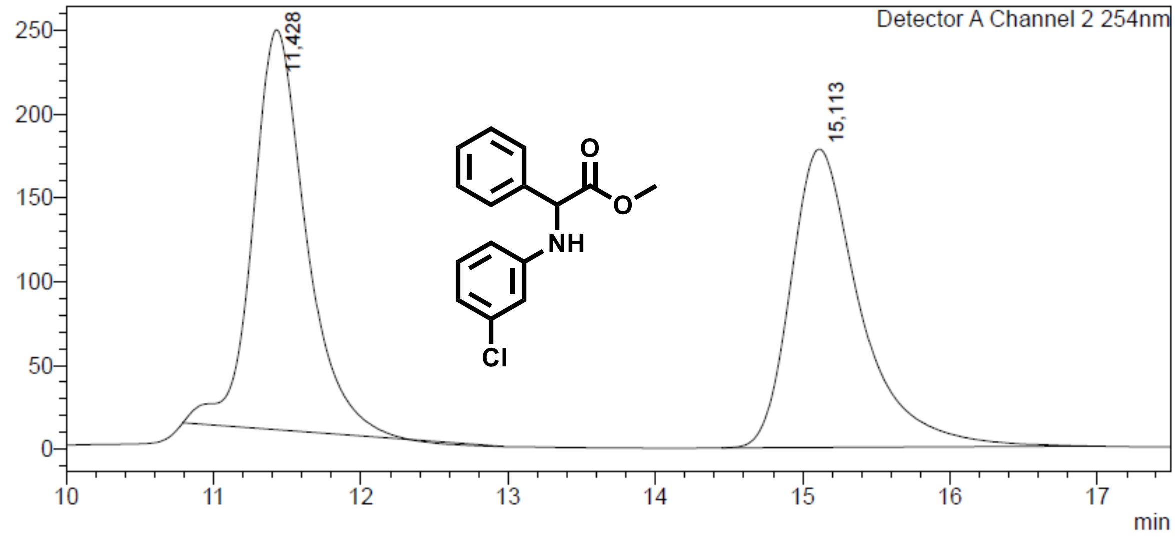

Detector A Channel 2 254nm

Peak#	Ret. Time	Area	Height	Conc.	Unit	Mark	Name
1	11,428	6026947	238613	50,757		M	
2	15,113	5847140	177944	49,243		M	
Total		11874087	416557				

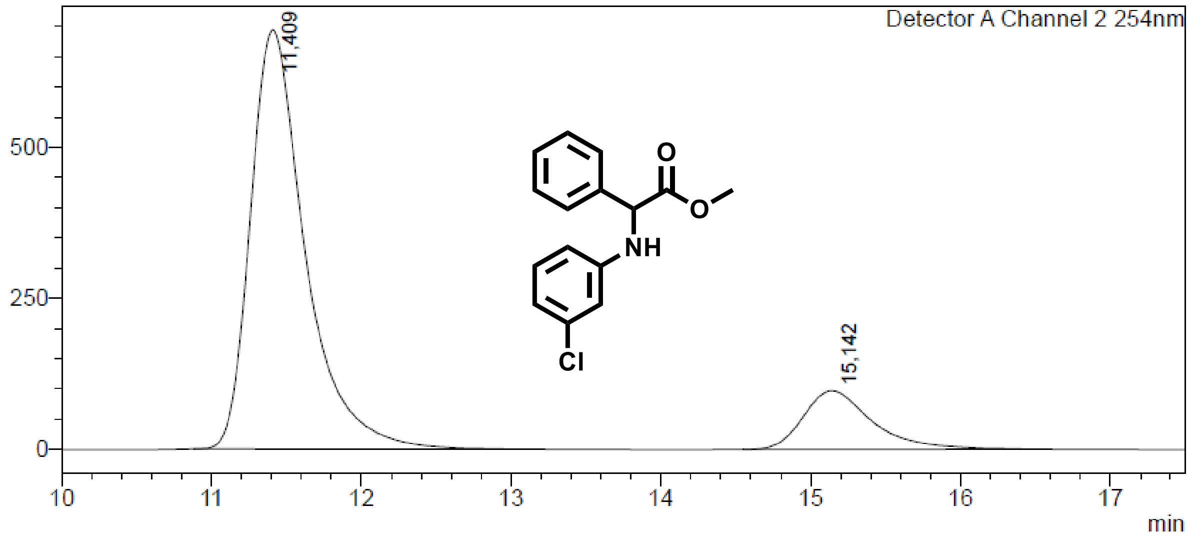

Detector A Channel 2 254nm

Peak#	Ret. Time	Area	Height	Conc.	Unit	Mark	Name
1	11,409	17597709	693852	84,965		M	
2	15,142	3113906	96872	15,035		M	
Total		20711616	790724				

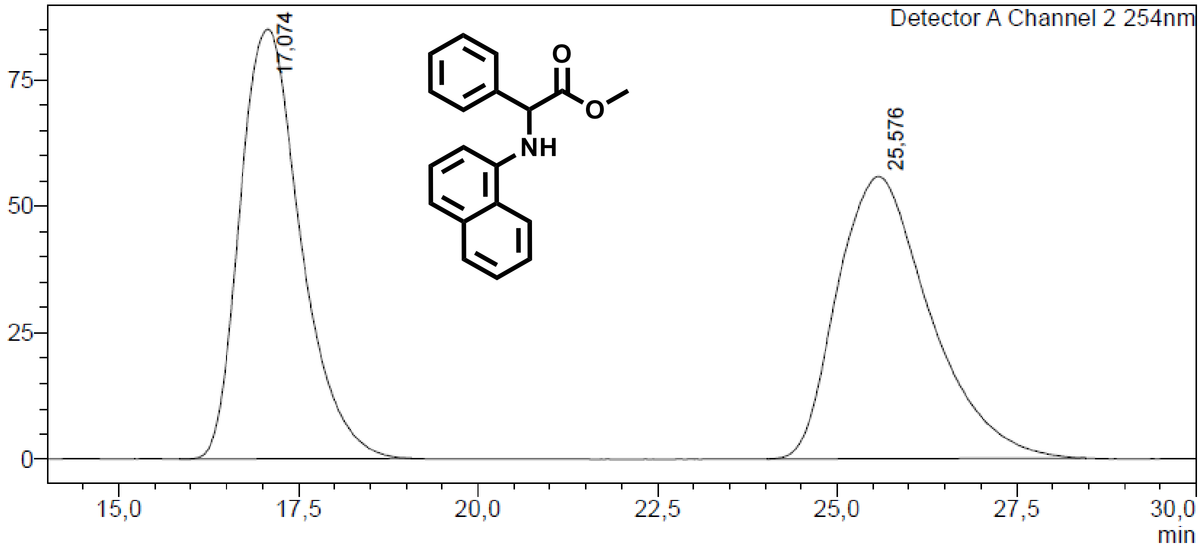

Detector A Channel 2 254nm

Peak#	Ret. Time	Area	Height	Conc.	Unit	Mark	Name
1	17,074	5000797	84832	49,977		M	
2	25,576	5005409	55696	50,023		M	
Total		10006206	140527				

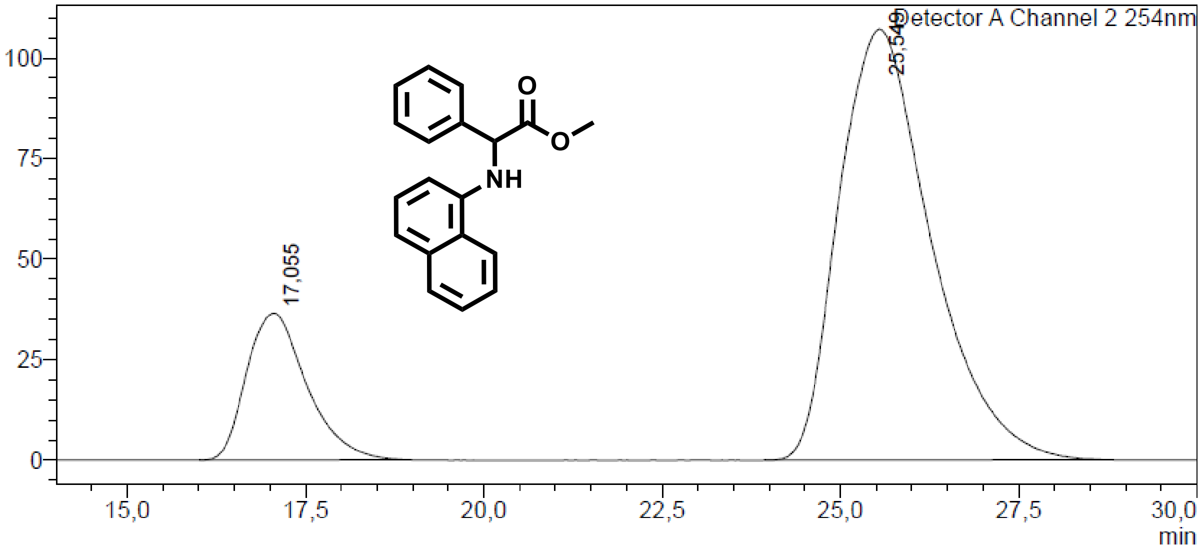

Detector A Channel 2 254nm

Peak#	Ret. Time	Area	Height	Conc.	Unit	Mark	Name
1	17,055	2143181	36474	18,199		M	
2	25,549	9633003	107112	81,801		M	
Total		11776185	143586				

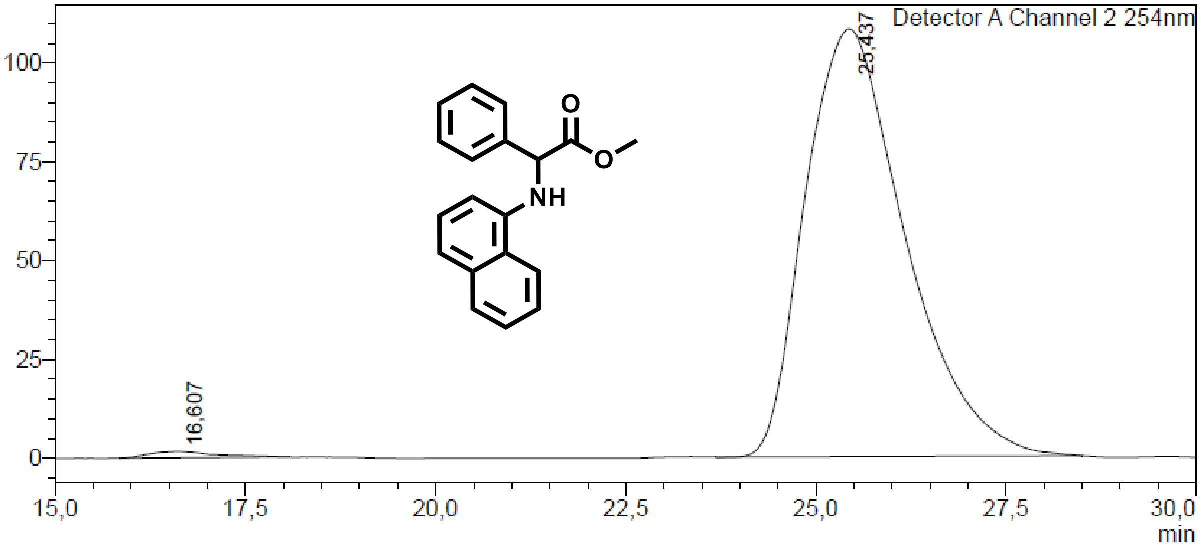

Detector A Channel 2 254nm

Peak#	Ret. Time	Area	Height	Conc.	Unit	Mark	Name
1	16,607	94331	1605	0,945		M	
2	25,437	9892256	108131	99,055		M	
Total		9986587	109736				

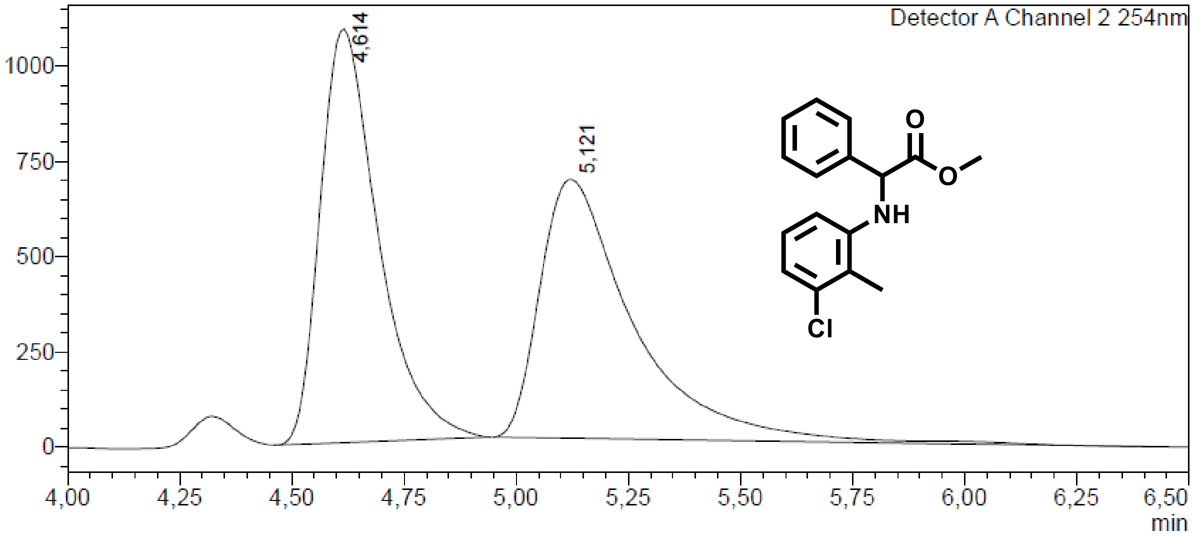

Detector A Channel 2 254nm

Peak#	Ret. Time	Area	Height	Conc.	Unit	Mark	Name
1	4,614	9787747	1086282	49,722		M	
2	5,121	9897184	679472	50,278		M	
Total		19684932	1765754				

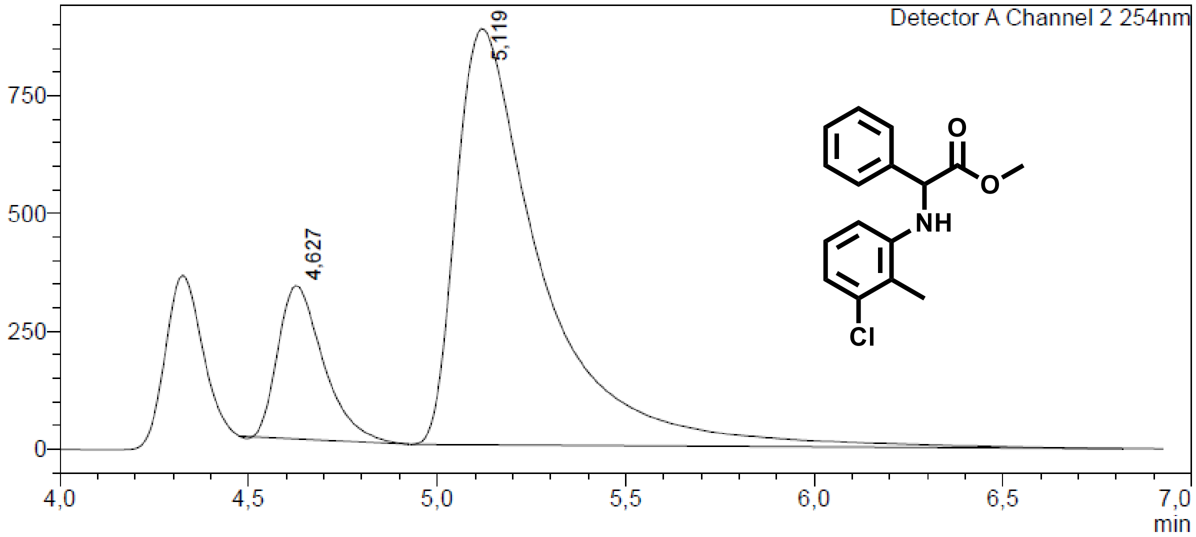

Detector A Channel 2 254nm

Peak#	Ret. Time	Area	Height	Conc.	Unit	Mark	Name
1	4,627	2726463	325211	16,301		M	
2	5,119	13999513	883103	83,699		M	
Total		16725976	1208314				

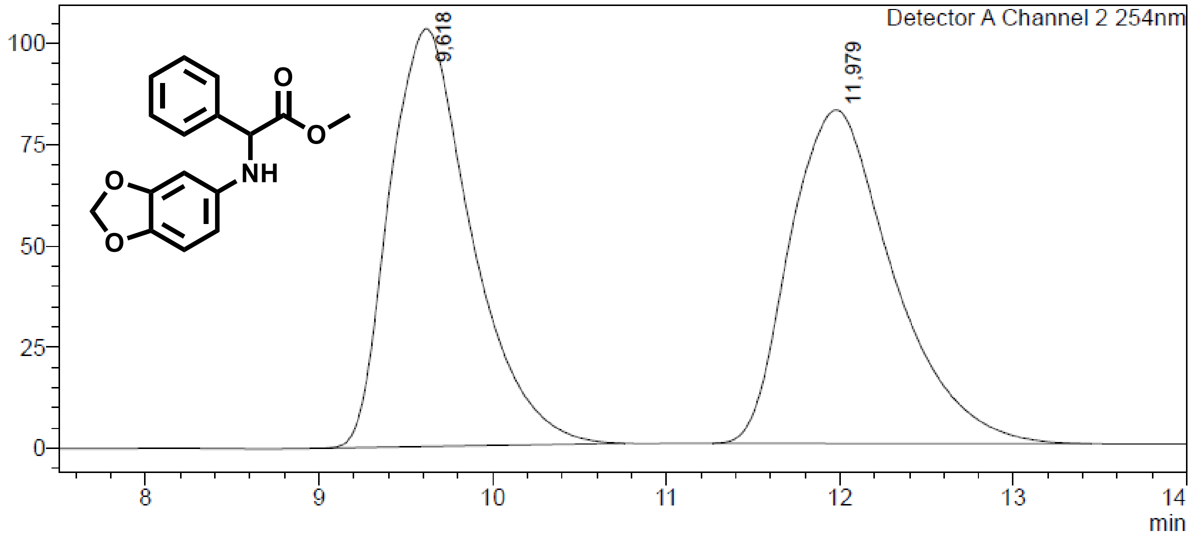

Detector A Channel 2 254nm

Peak#	Ret. Time	Area	Height	Conc.	Unit	Mark	Name
1	9,618	3400774	103127	49,875		M	
2	11,979	3417839	82350	50,125		M	
Total		6818613	185477				

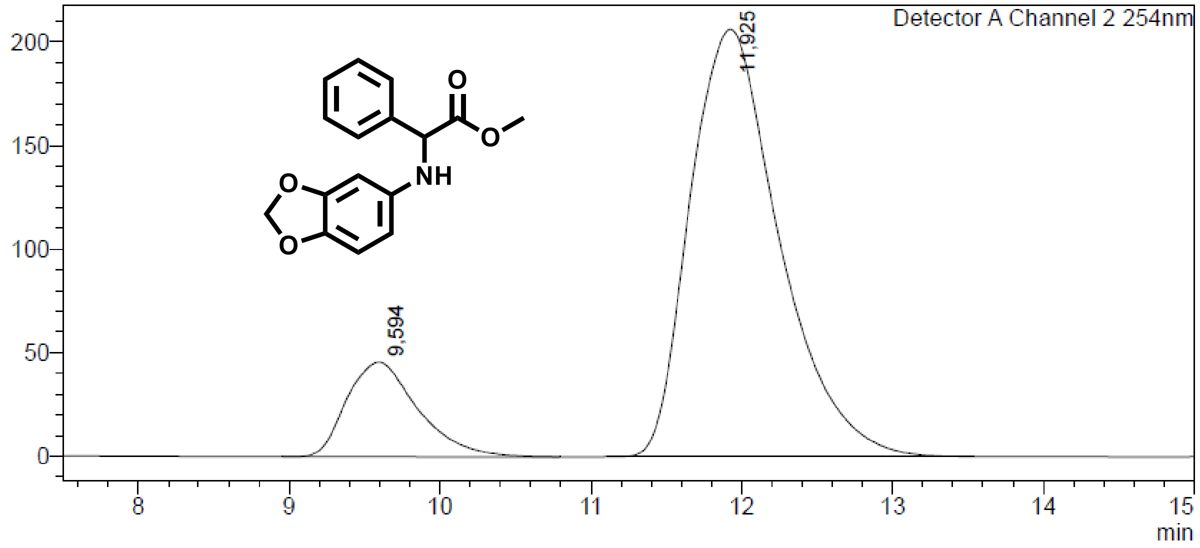

Detector A Channel 2 254nm

Peak#	Ret. Time	Area	Height	Conc.	Unit	Mark	Name
1	9,594	1495746	45592	14,996		M	
2	11,925	8478693	206153	85,004		M	
Total		9974439	251745				

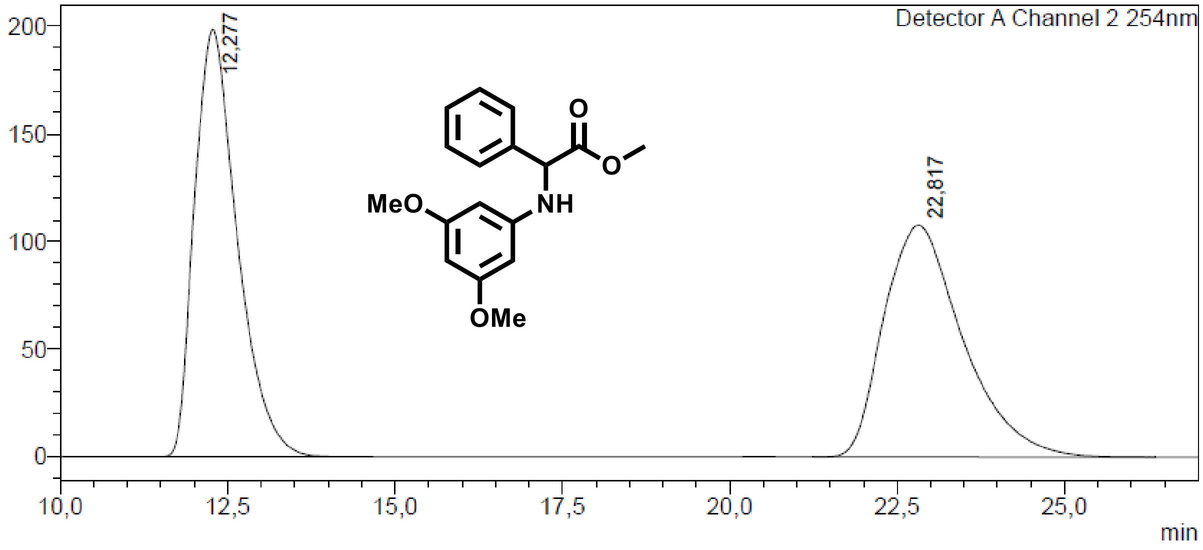

Detector A Channel 2 254nm

Peak#	Ret. Time	Area	Height	Conc.	Unit	Mark	Name
1	12,277	8830711	198421	49,977		M	
2	22,817	8839008	107571	50,023		M	
Total		17669719	305992				

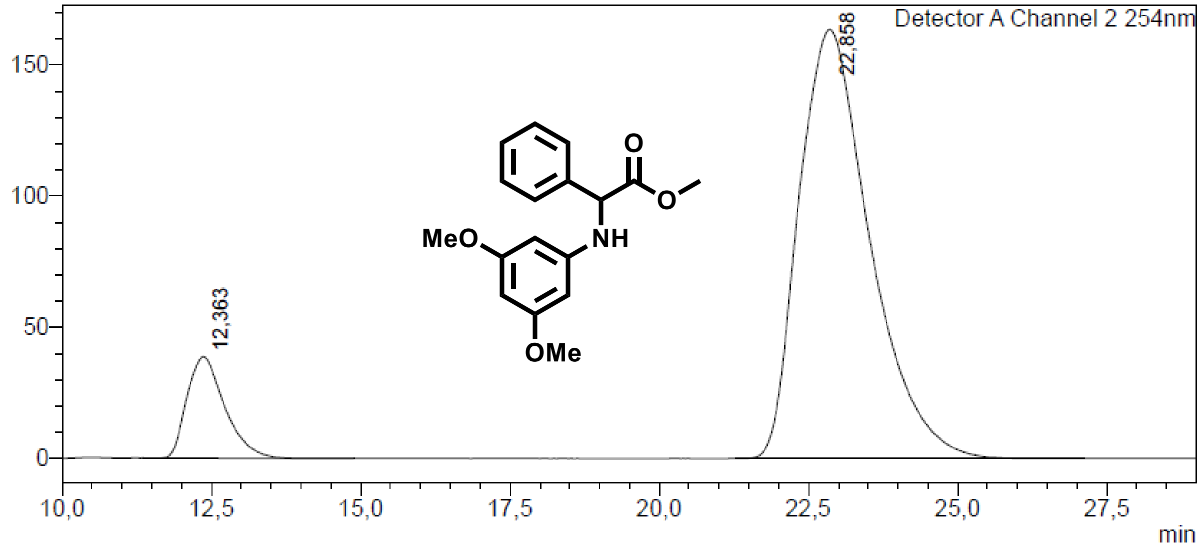

Detector A Channel 2 254nm

Peak#	Ret. Time	Area	Height	Conc.	Unit	Mark	Name
1	12,363	1698067	38663	11,163		M	
2	22,858	13514138	163548	88,837		M	
Total		15212205	202211				

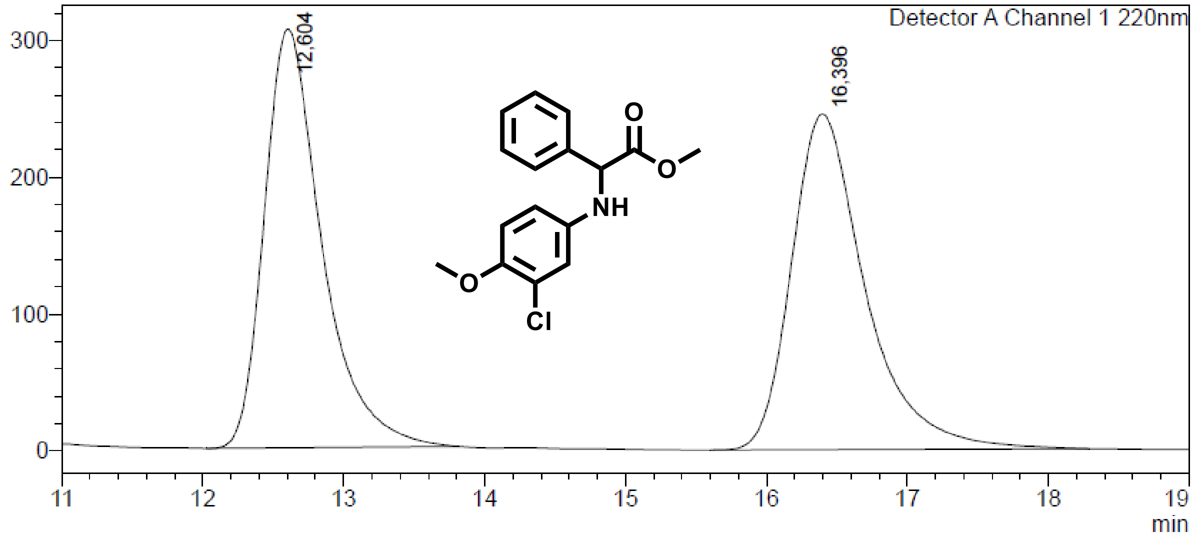

Peak#	Ret. Time	Area	Height	Conc.	Unit	Mark	Name
1	12,604	8696068	305769	49,371		M	
2	16,396	8917764	245005	50,629		M	
Total		17613832	550774				

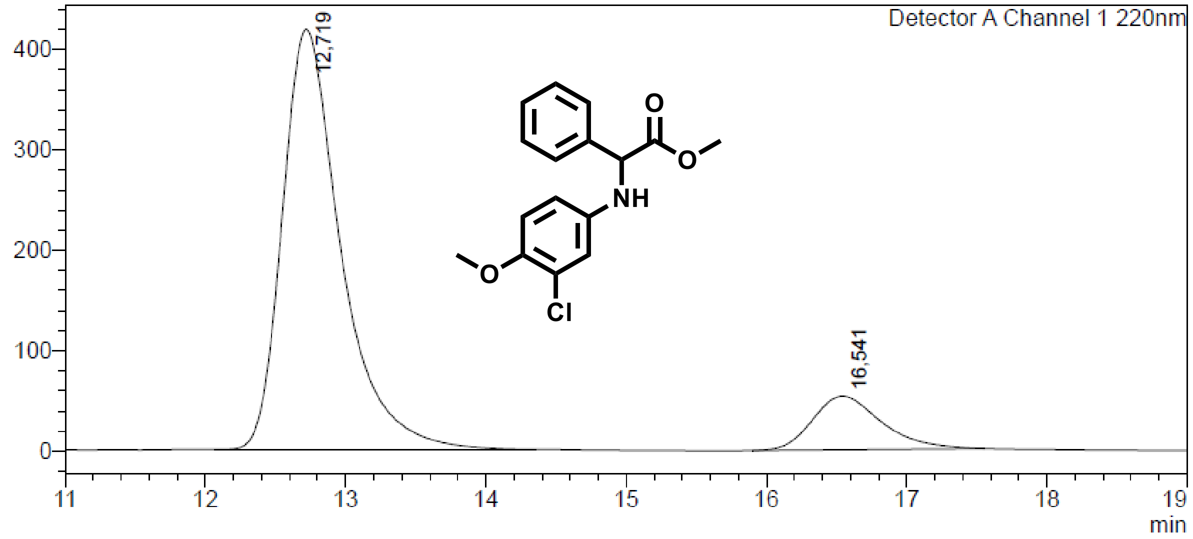

Peak#	Ret. Time	Area	Height	Conc.	Unit	Mark	Name
1	12,719	12118286	418440	86,818		M	
2	16,541	1840013	53514	13,182		M	
Total		13958299	471954				

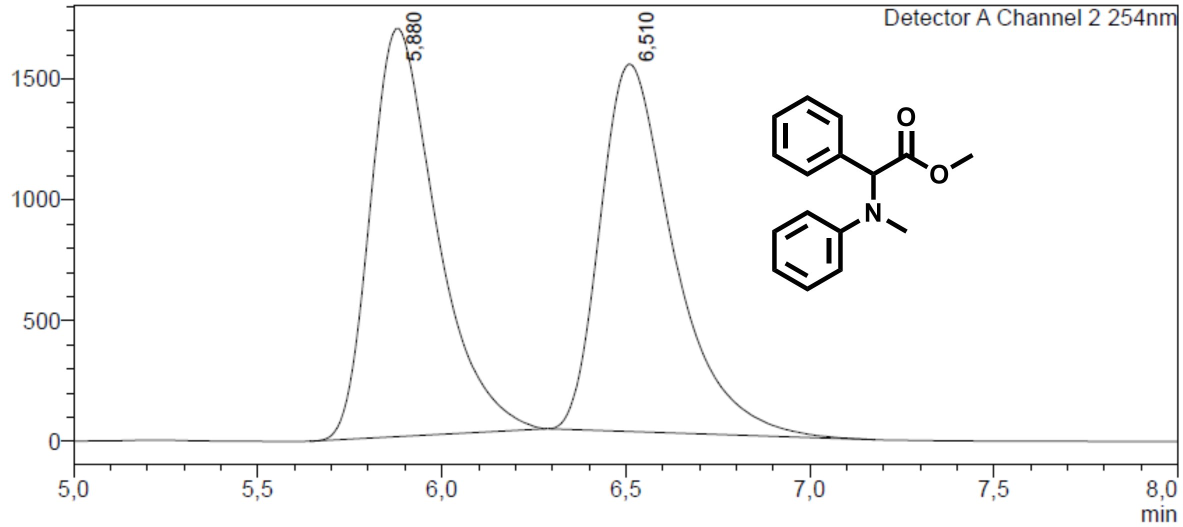

Detector A Channel 2 254nm

Peak#	Ret. Time	Area	Height	Conc.	Unit	Mark	Name
1	5,880	21194095	1687950	49,978		M	
2	6,510	21212398	1520524	50,022		M	
Total		42406493	3208474				

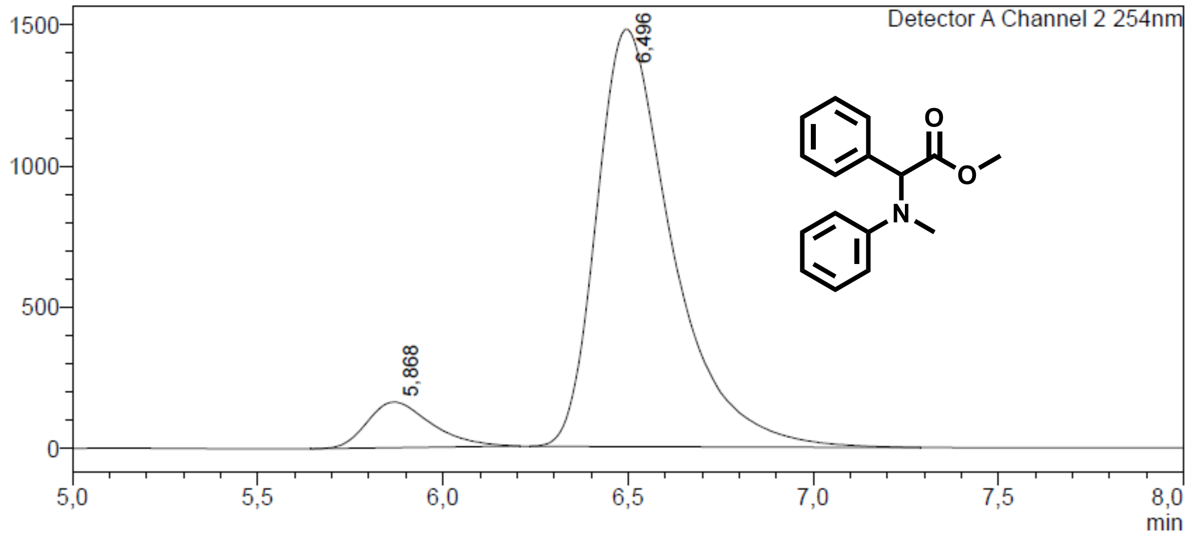

Detector A Channel 2 254nm

Peak#	Ret. Time	Area	Height	Conc.	Unit	Mark	Name
1	5,868	1948638	161703	8,466		M	
2	6,496	21069727	1475257	91,534		M	
Total		23018365	1636960				

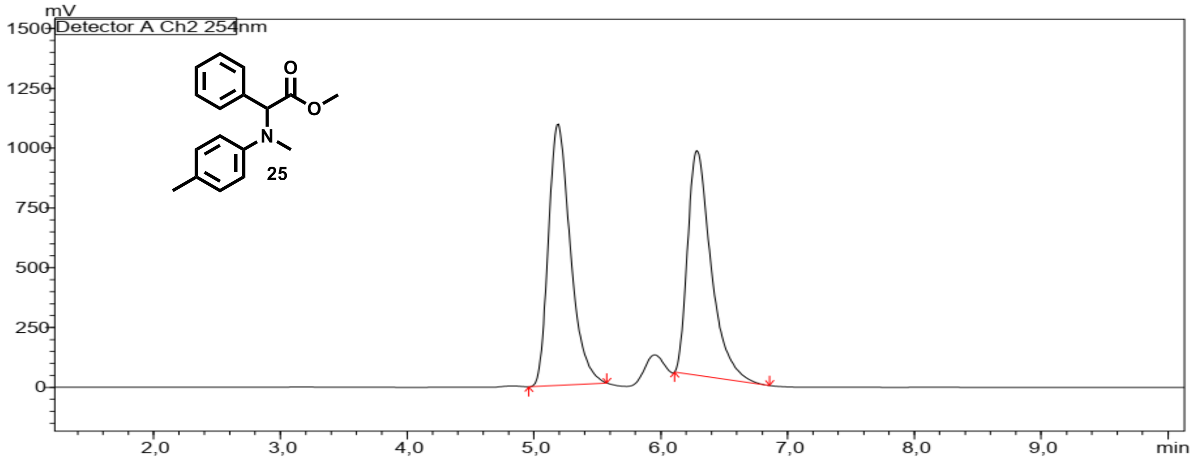

Detector A Channel 2 254nm

Peak#	Ret. Time	Area	Height	Area%
1	5,188	13186998	1092214	52,185
2	6,285	12082753	939087	47,815

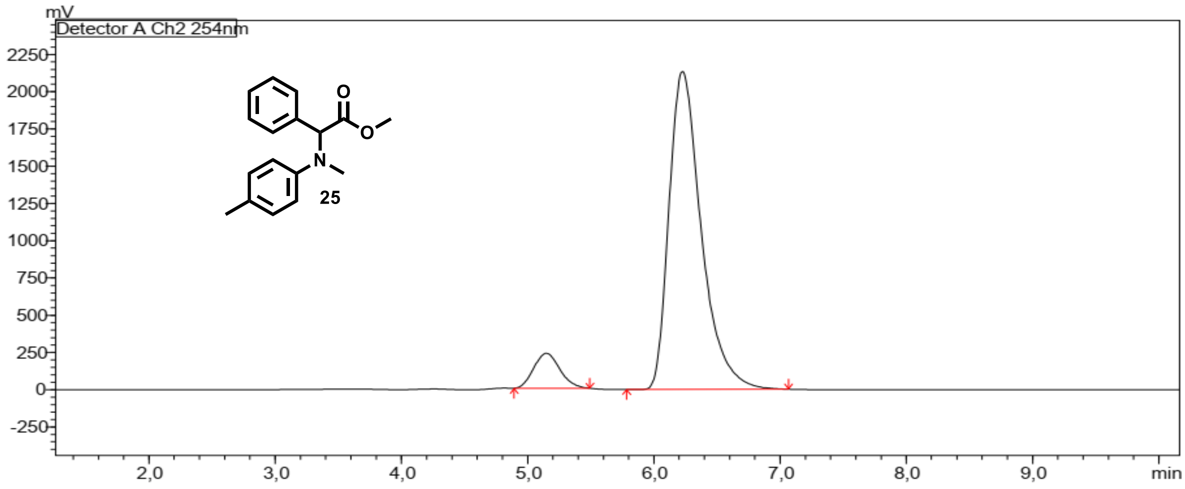

Detector A Channel 2 254nm

Peak#	Ret. Time	Area	Height	Area%
1	5,148	3396223	235359	8,227
2	6,227	37885137	2134443	91,773

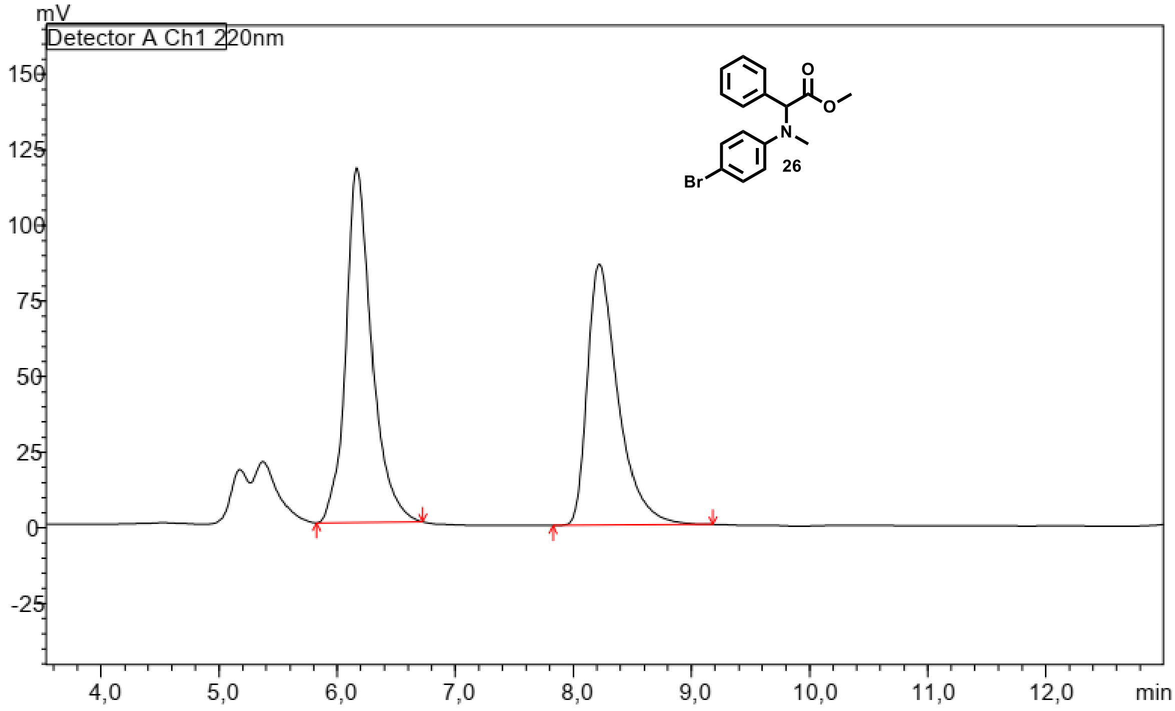

Detector A Channel 1 220nm

Peak#	Ret. Time	Area	Height	Area%
1	6,168	1792991	117227	53,240
2	8,220	1574747	86351	46,760

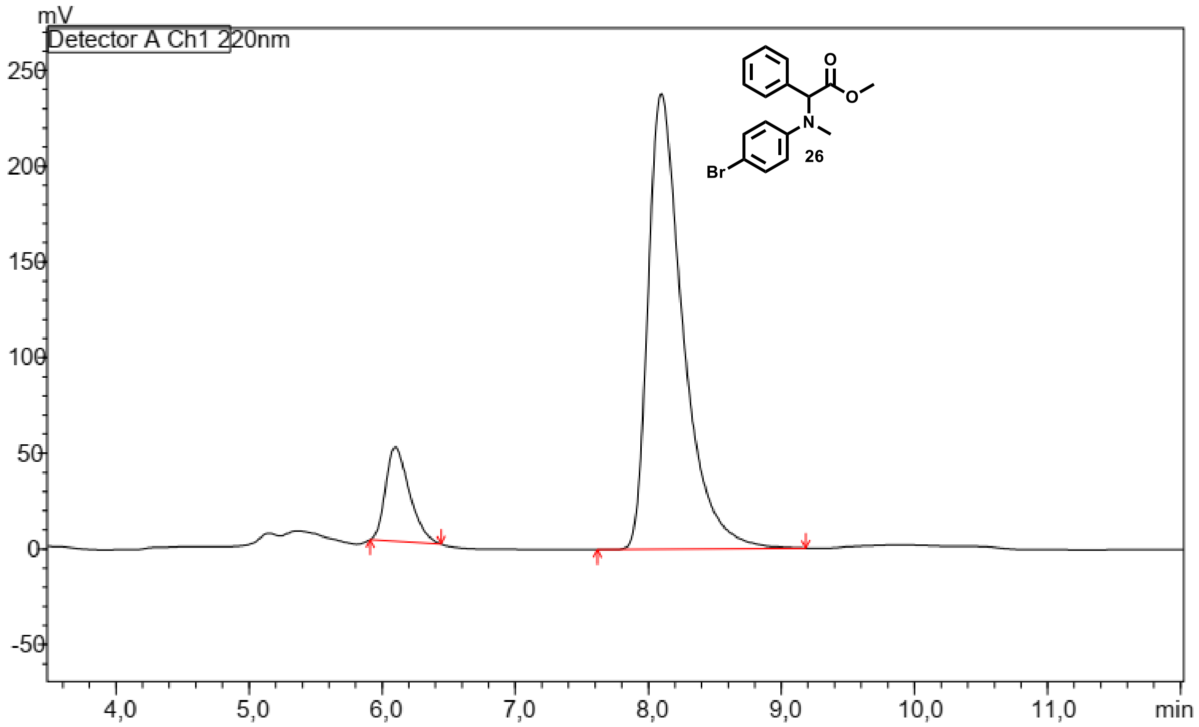

Detector A Channel 1 220nm

Peak#	Ret. Time	Area	Height	Area%
1	6,098	615394	49068	12,441
2	8,097	4330949	237714	87,559

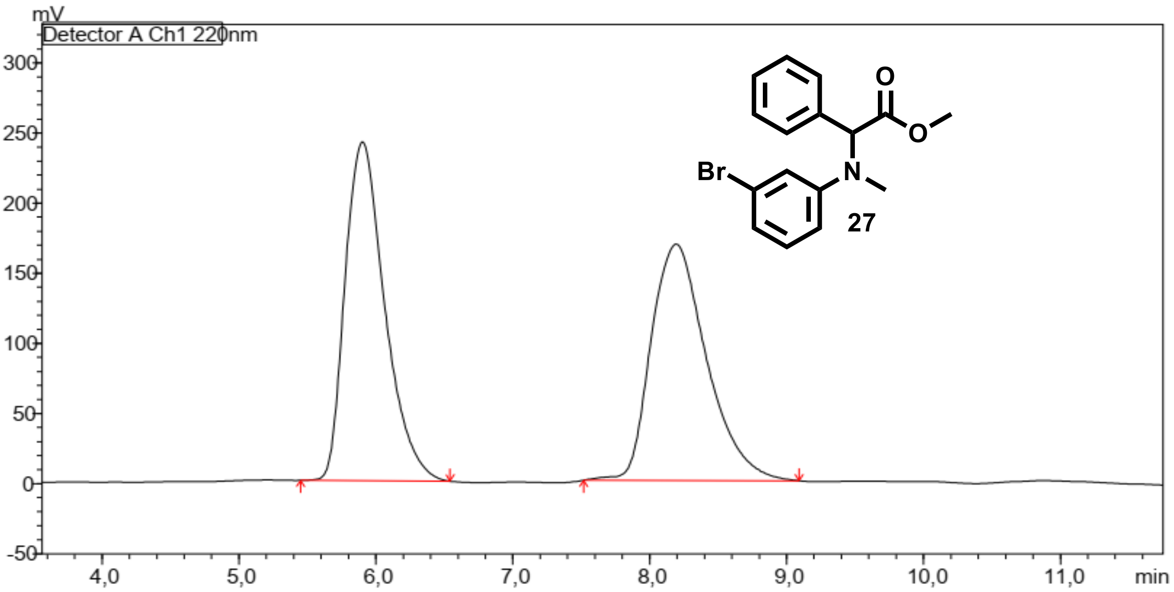

Detector A Channel 1 220nm

Peak#	Ret. Time	Area	Height	Conc.	Area%
1	5,902	4875694	241580	50,611	50,611
2	8,193	4757971	168570	49,389	49,389

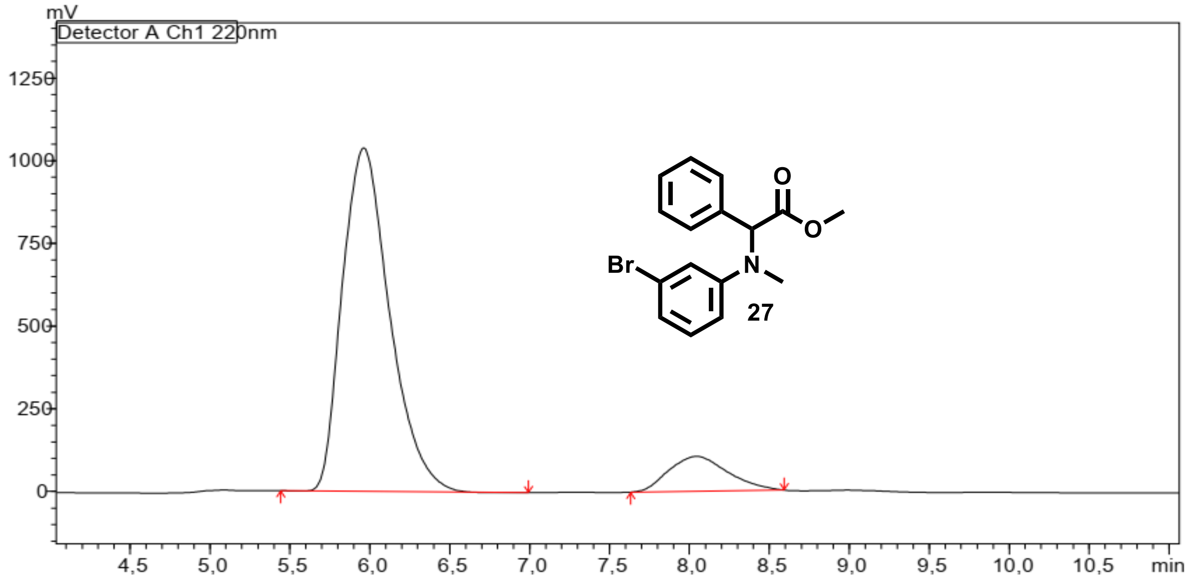

Detector A Channel 1 220nm

Peak#	Ret. Time	Area	Height	Conc.	Area%
1	5,963	21360835	1037524	88,749	88,749
2	8,044	2708075	105322	11,251	11,251

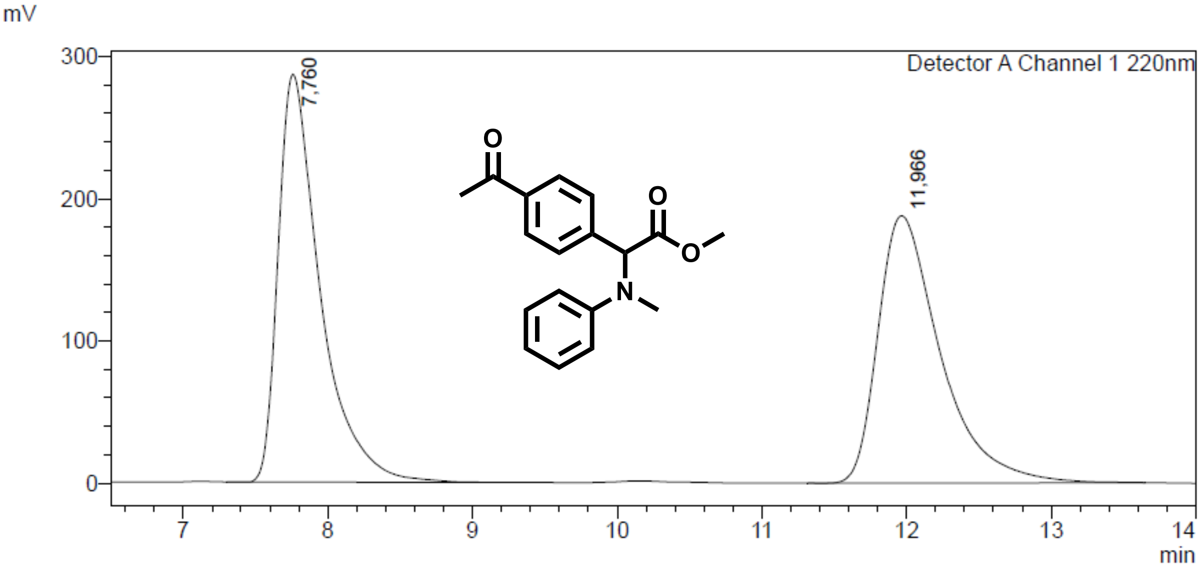

Peak#	Ret. Time	Area	Height	Conc.	Unit	Mark	Name
1	7,760	5780923	286959	50,619		M	
2	11,966	5639566	188109	49,381		M	
Total		11420489	475068				

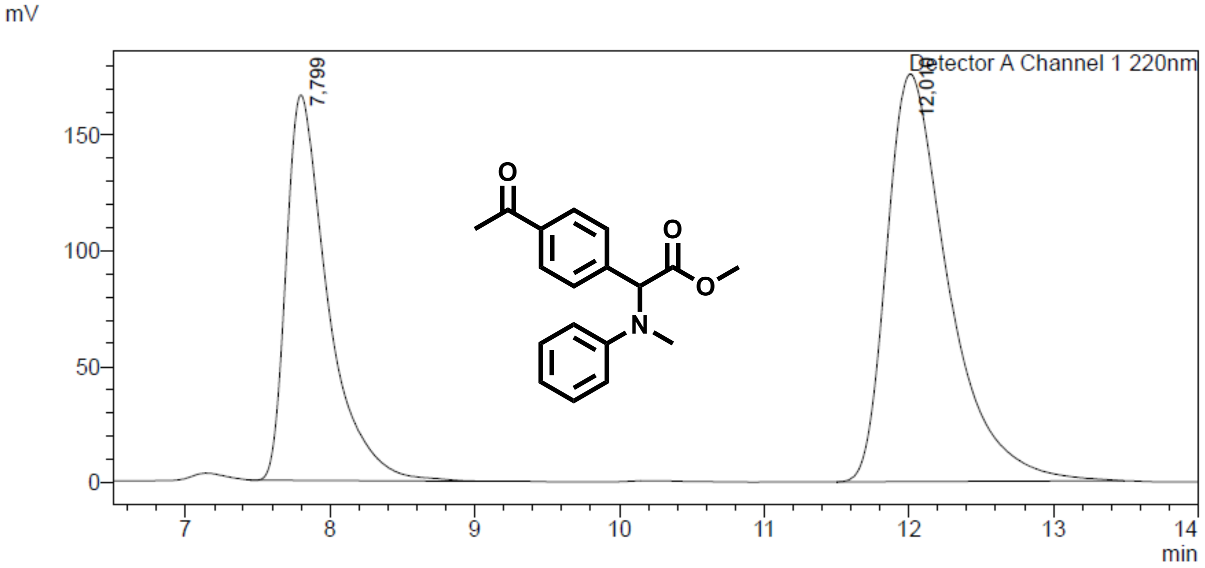

Peak#	Ret. Time	Area	Height	Conc.	Unit	Mark	Name
1	7,799	3328985	166458	38,975		M	
2	12,010	5212387	175960	61,025		M	
Total		8541372	342418				

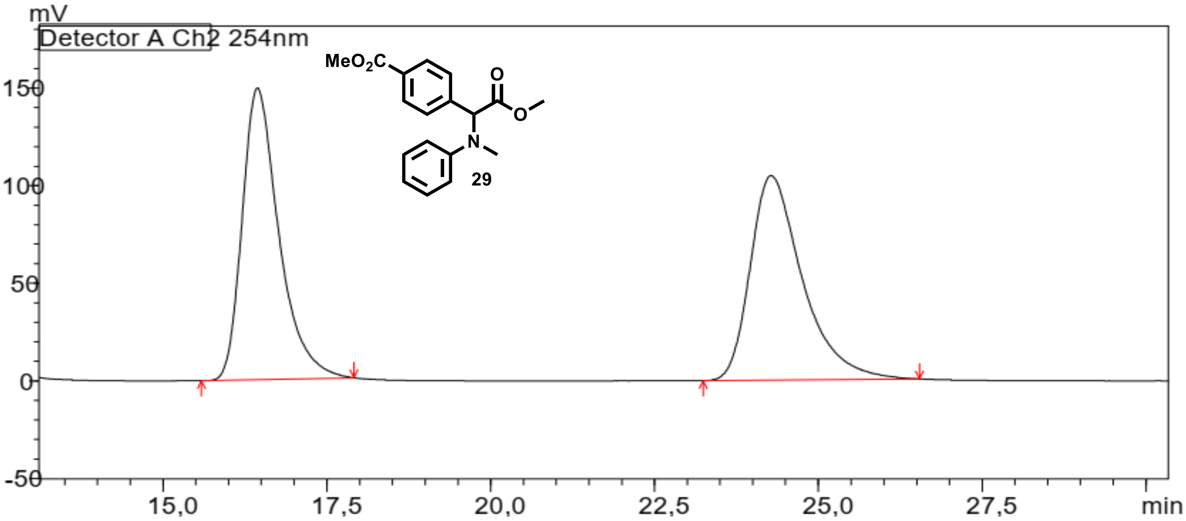

Detector A Channel 2 254nm

Peak#	Ret. Time	Area	Height	Conc.
1	16,440	5820466	149404	50,025
2	24,279	5814555	104830	49,975

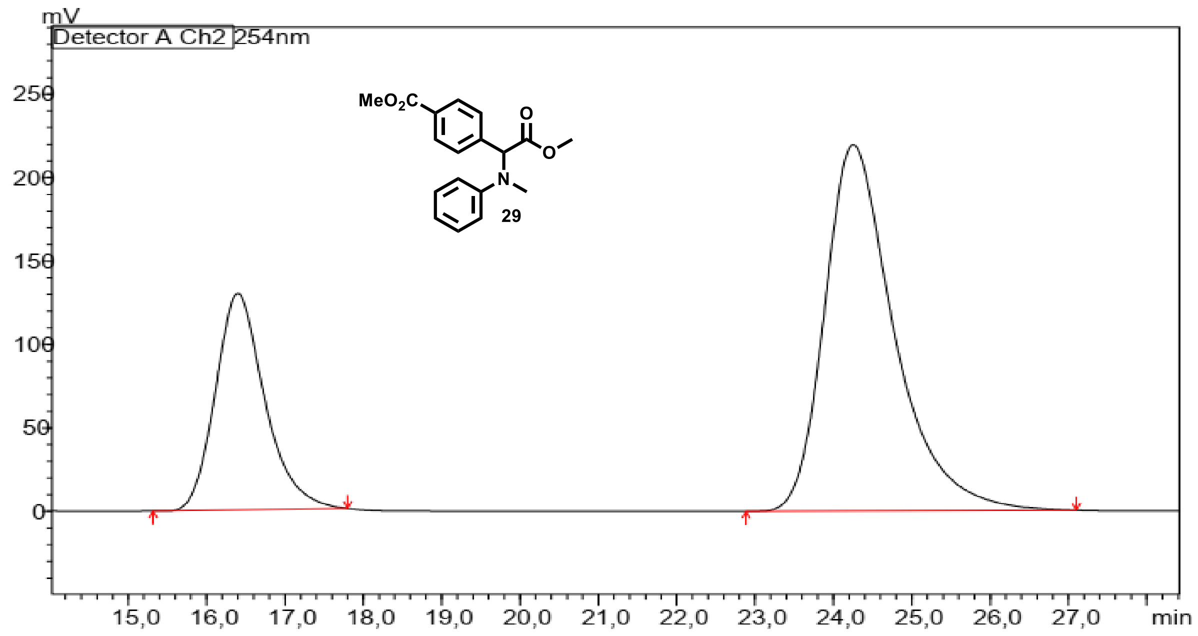

Detector A Channel 2 254nm

Peak#	Ret. Time	Area	Height	Area%
1	16,399	5696404	129802	29,545
2	24,253	13584280	219618	70,455

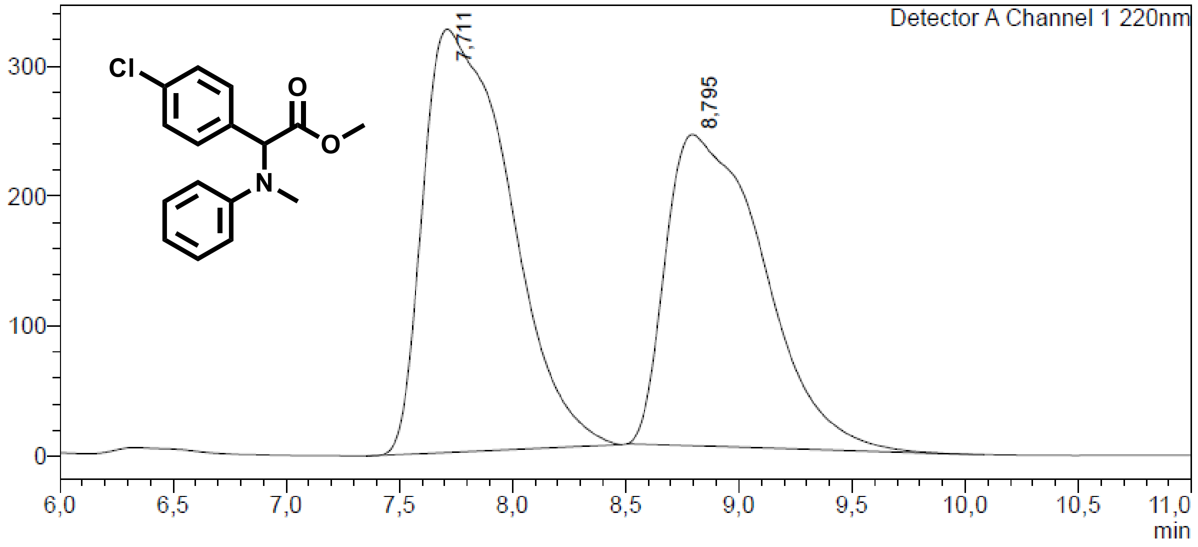

Peak#	Ret. Time	Area	Height	Conc.	Unit	Mark	Name
1	7,711	8676776	325125	54,567		M	
2	8,795	7224287	239040	45,433		M	
Total		15901064	564165				

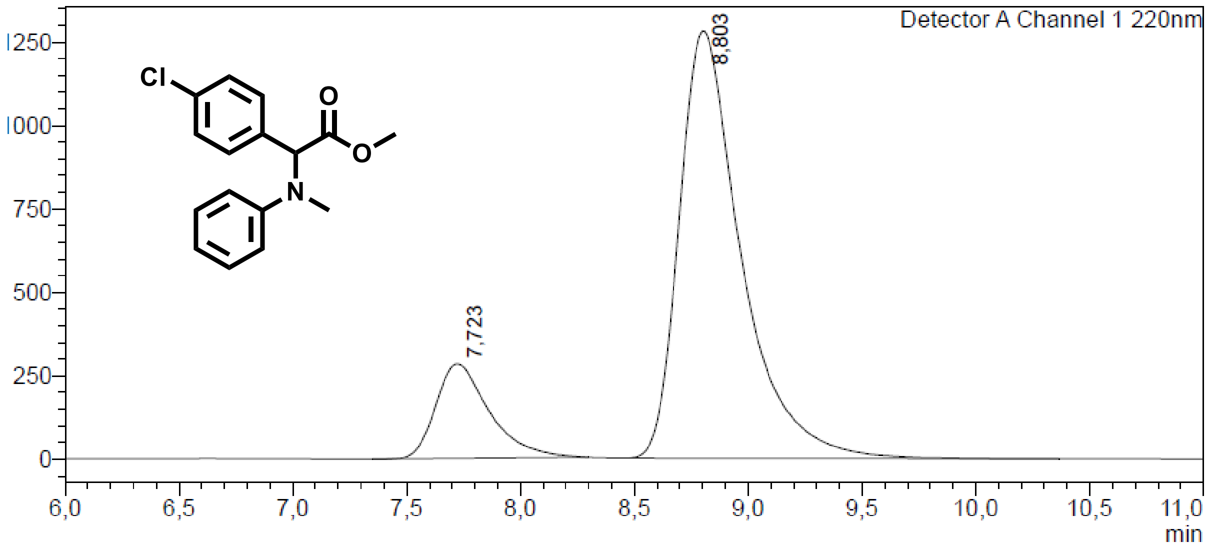

Peak#	Ret. Time	Area	Height	Conc.	Unit	Mark	Name
1	7,723	4637054	283479	16,061		M	
2	8,803	24234736	1279819	83,939		M	
Total		28871790	1563297				

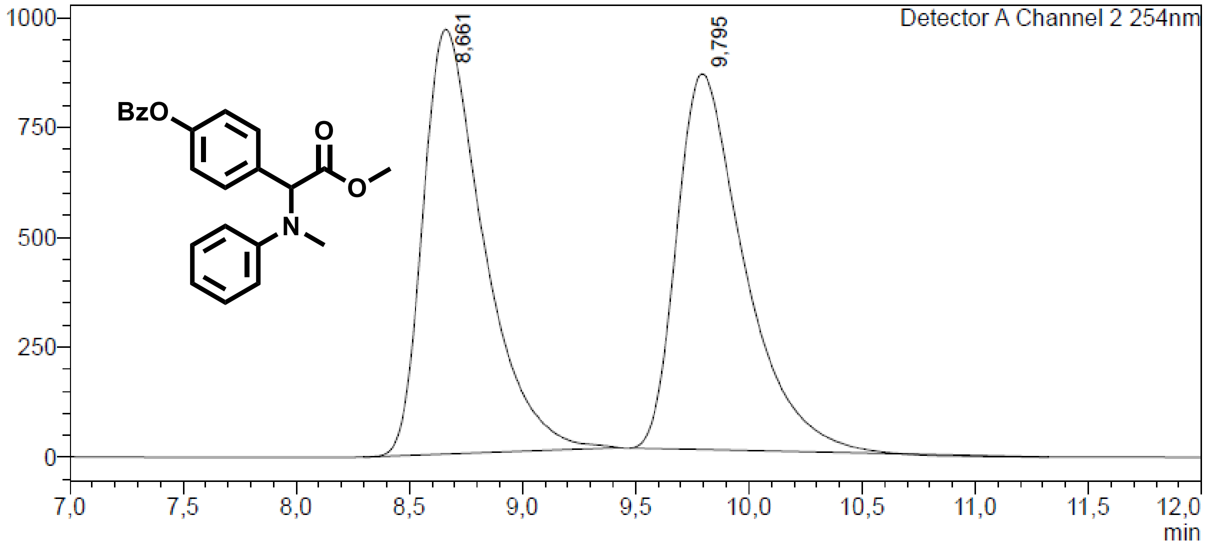

Detector A Channel 2 254nm

Peak#	Ret. Time	Area	Height	Conc.	Unit	Mark	Name
1	8,661	18010077	965916	50,381		M	
2	9,795	17737983	854464	49,619		M	
Total		35748060	1820380				

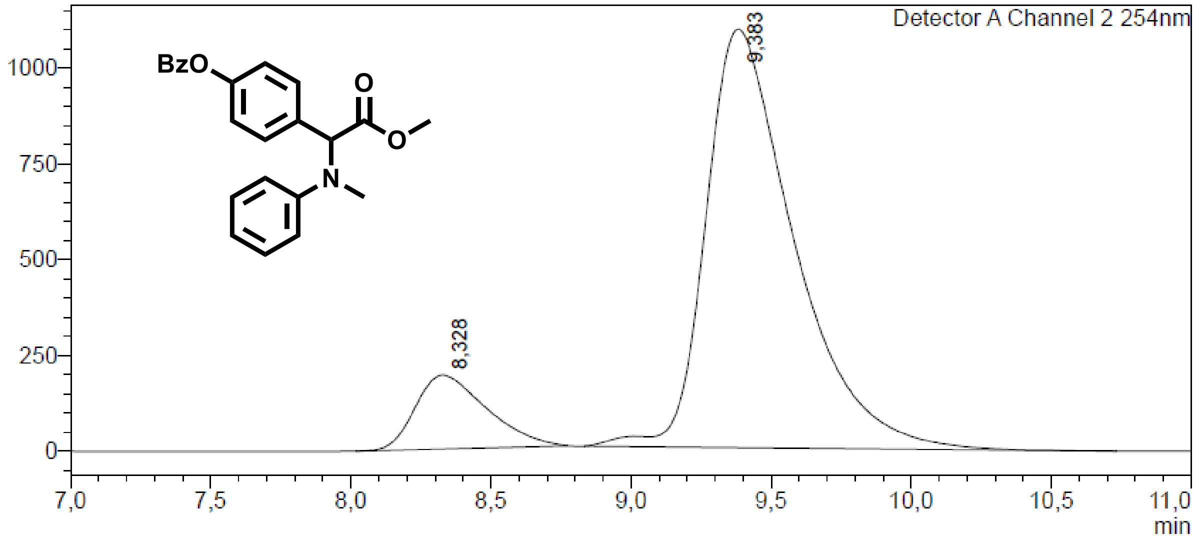

Detector A Channel 2 254nm

Peak#	Ret. Time	Area	Height	Conc.	Unit	Mark	Name
1	8,328	3404829	192582	12,431		M	
2	9,383	23984963	1092079	87,569		M	
Total		27389791	1284661				

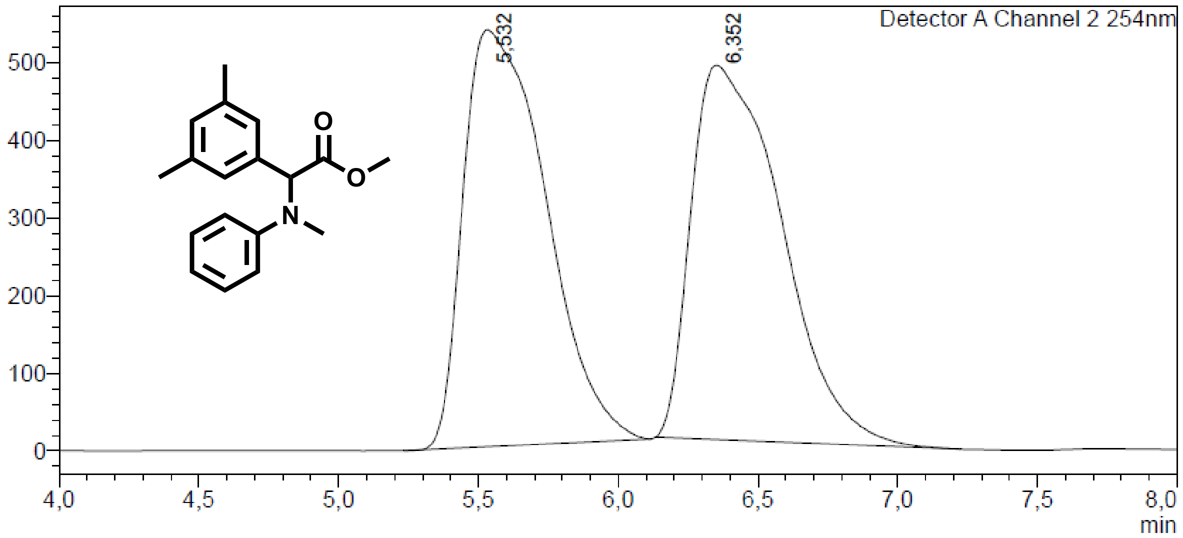

Detector A Channel 2 254nm

Peak#	Ret. Time	Area	Height	Conc.	Unit	Mark	Name
1	5,532	11072204	537208	50,354		M	
2	6,352	10916408	482686	49,646		M	
Total		21988612	1019894				

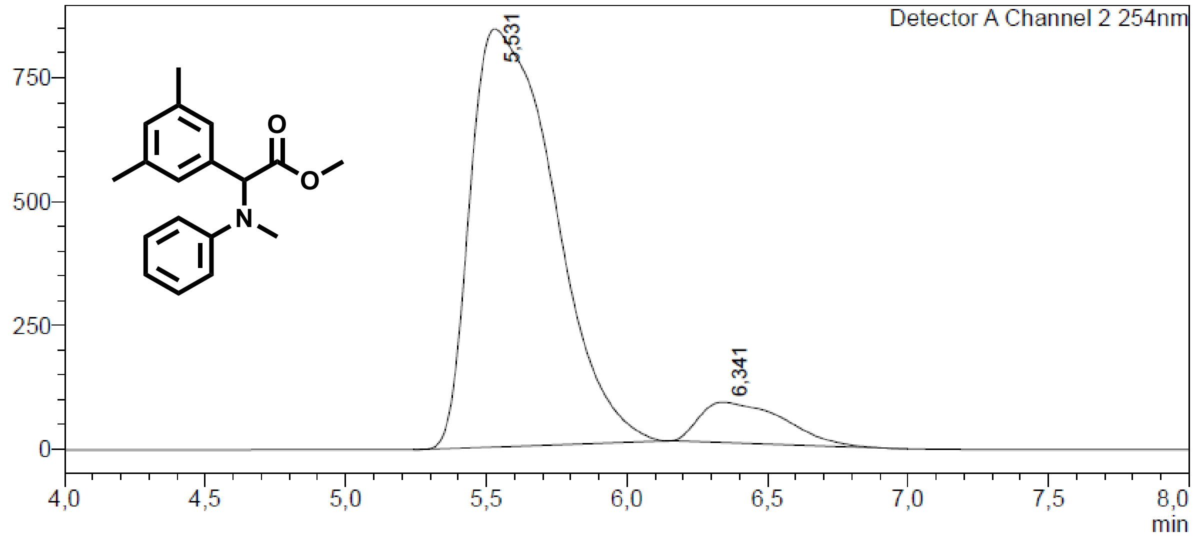

Detector A Channel 2 254nm

Peak#	Ret. Time	Area	Height	Conc.	Unit	Mark	Name
1	5,531	17573487	842979	91,062		M	
2	6,341	1724928	81598	8,938		M	
Total		19298415	924577				

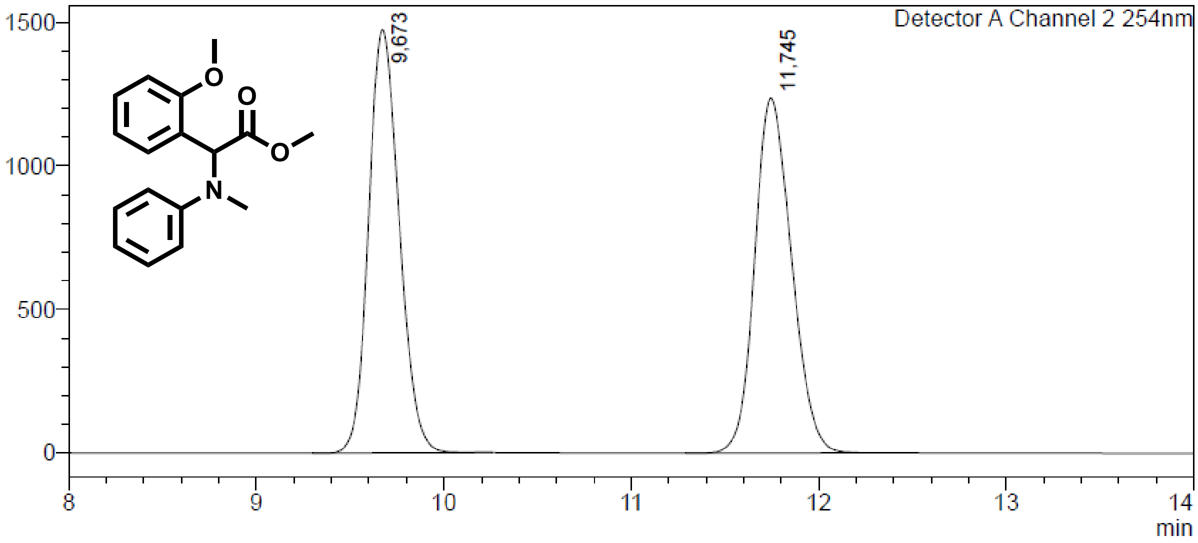

Detector A Channel 2 254nm

Peak#	Ret. Time	Area	Height	Conc.	Unit	Mark	Name
1	9,673	16763683	1474397	49,781		M	
2	11,745	16911321	1235233	50,219		M	
Total		33675004	2709631				

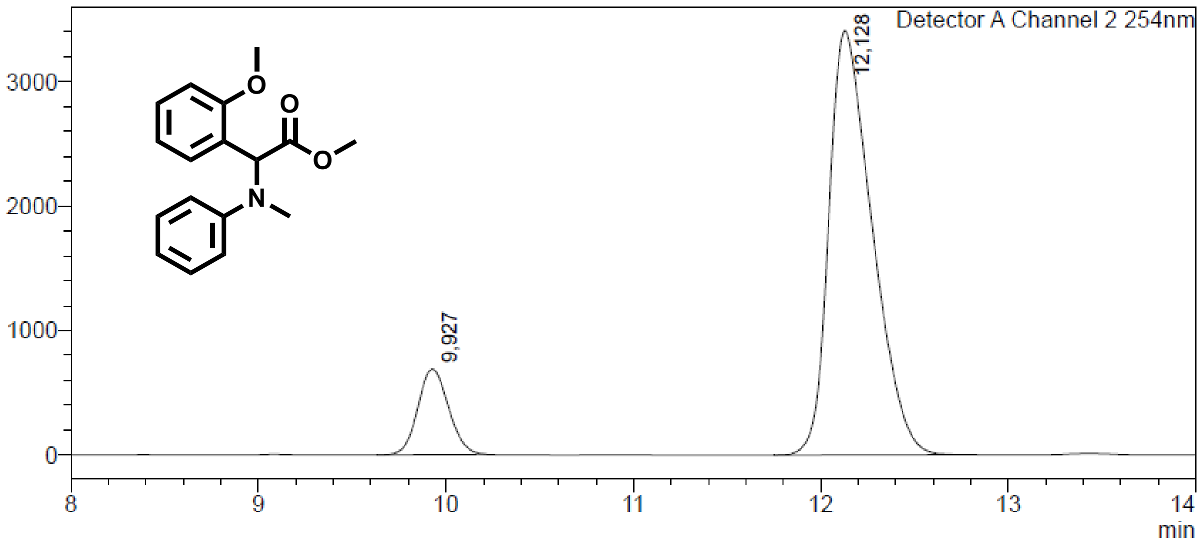

Detector A Channel 2 254nm

Peak#	Ret. Time	Area	Height	Conc.	Unit	Mark	Name
1	9,927	7825256	688029	12,531		M	
2	12,128	54622222	3406246	87,469		M	
Total		62447478	4094275				

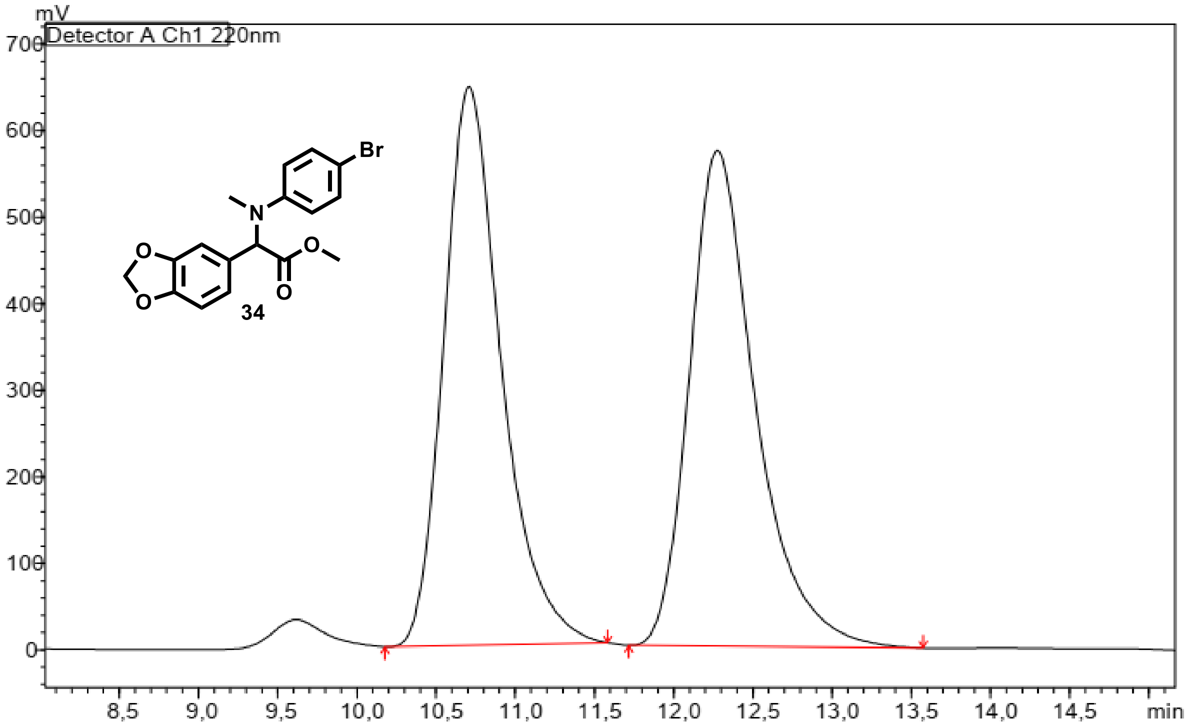

Detector A Channel 1 220nm

Peak#	Ret. Time	Area	Height	Conc.	Area%
1	10,707	16329314	645415	49,508	49,508
2	12,276	16654020	572948	50,492	50,492

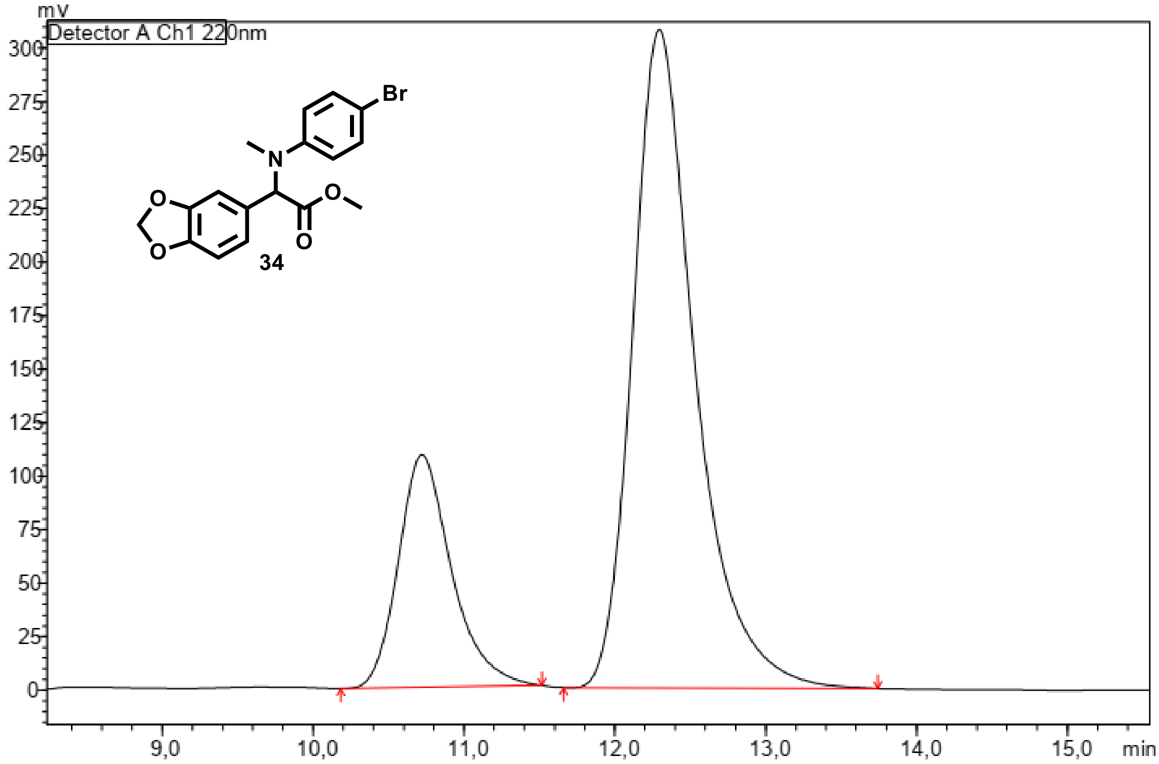

Detector A Channel 1 220nm

Peak#	Ret. Time	Area	Height	Conc.	Area%
1	10,720	2661764	108641	23,062	23,062
2	12,294	8879779	307628	76,938	76,938

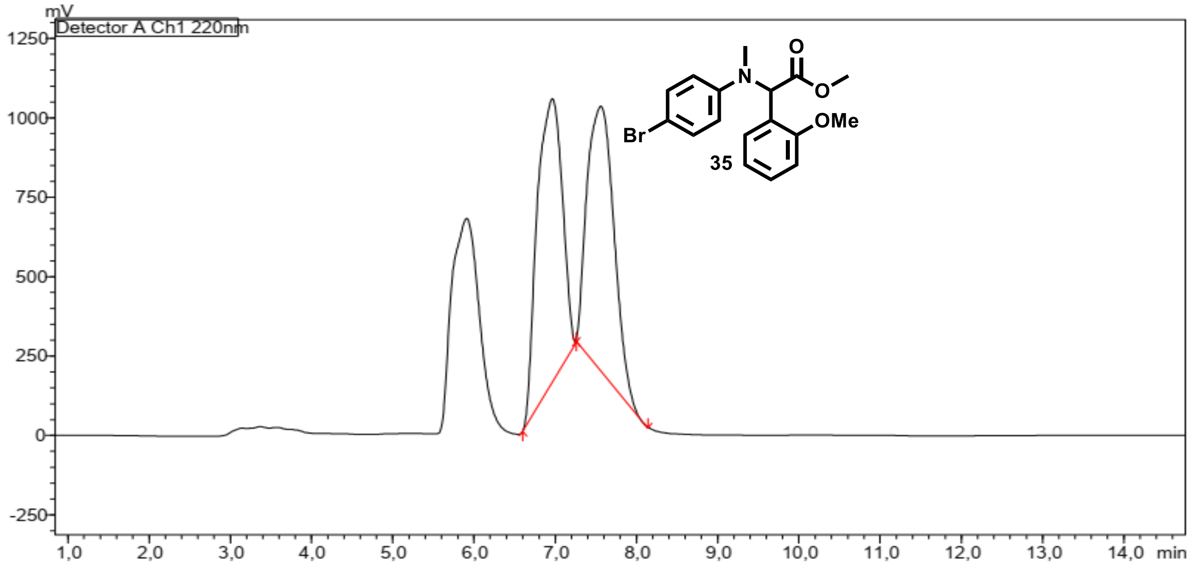

Detector A Channel 1 220nm

Peak#	Ret. Time	Area	Height	Conc.	Area%
1	6,965	19418572	891340	50,407	50,407
2	7,562	19105059	834210	49,593	49,593

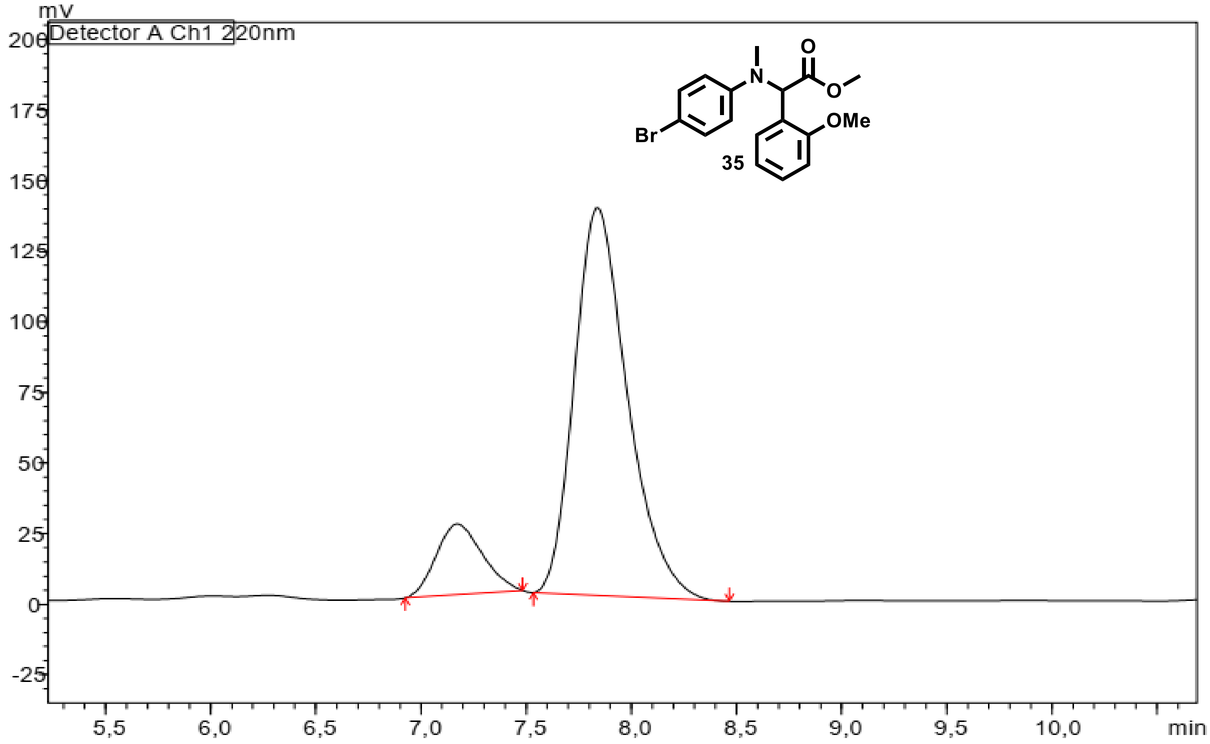

Detector A Channel 1 220nm

Peak#	Ret. Time	Area	Height	Conc.	Area%
1	7,172	381420	24970	13,629	13,629
2	7,839	2417146	137206	86,371	86,371

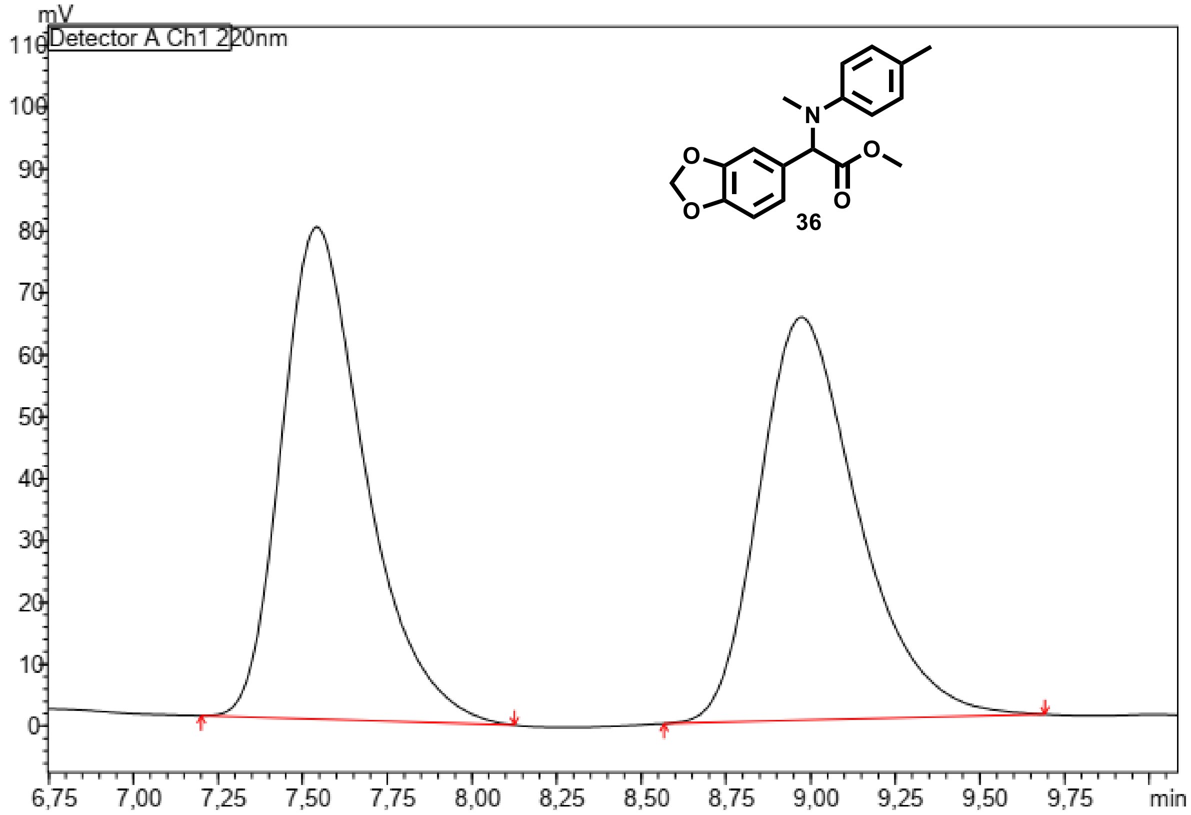

Detector A Channel 1 220nm

Peak#	Ret. Time	Area	Height	Conc.	Area%
1	7,542	1369321	79566	50,689	50,689
2	8,973	1332090	65154	49,311	49,311

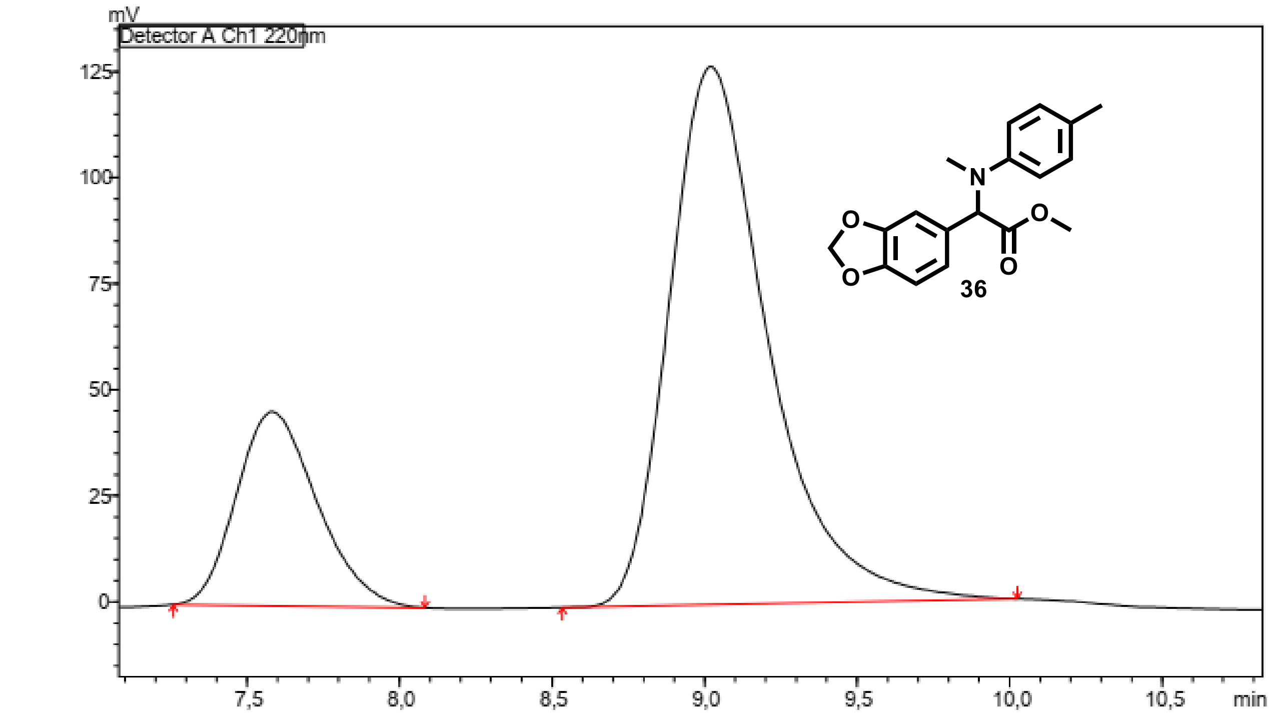

Detector A Channel 1 220nm

Peak#	Ret. Time	Area	Height	Conc.	Area%
1	7,582	841192	45735	22,714	22,714
2	9,021	2862188	126802	77,286	77,286

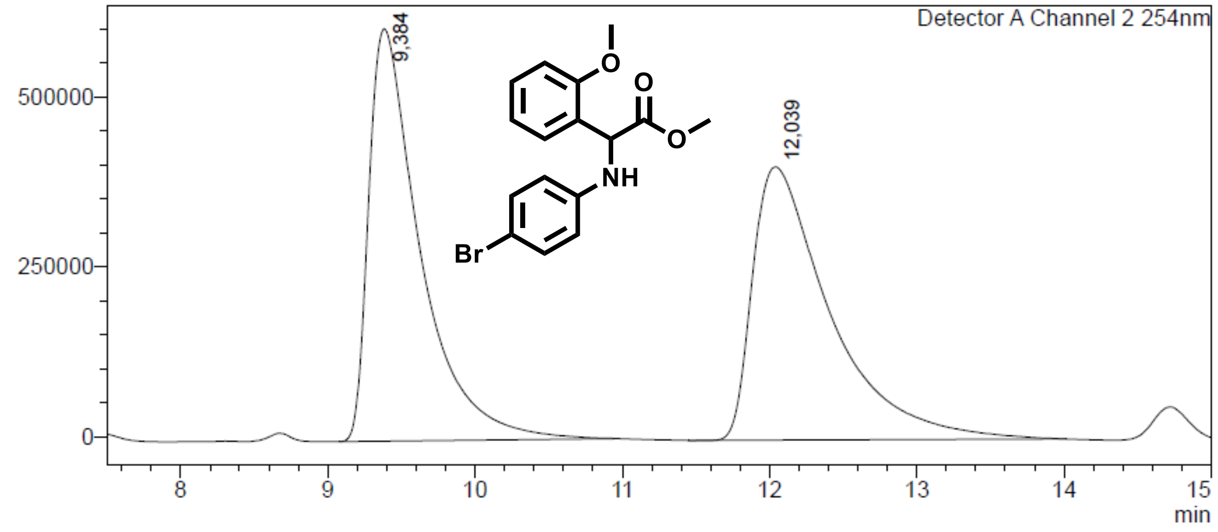

Detector A Channel 2 254nm

Peak#	Ret. Time	Area	Height	Conc.	Unit	Mark	Name
1	9,384	14798292	607200	50,101		M	
2	12,039	14738674	402204	49,899		M	
Total		29536966	1009404				

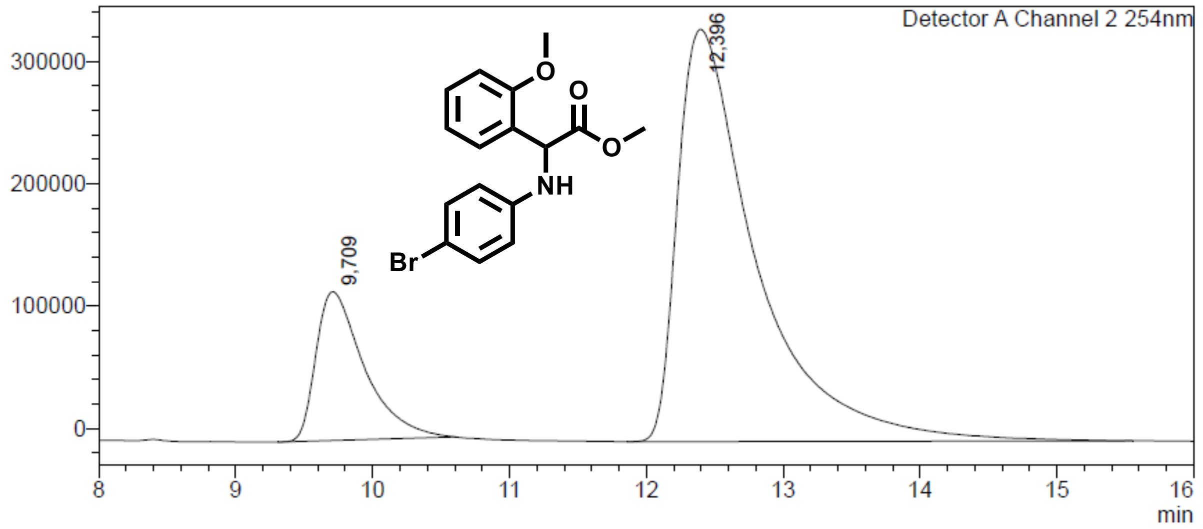

Detector A Channel 2 254nm

Peak#	Ret. Time	Area	Height	Conc.	Unit	Mark	Name
1	9,709	2984172	121476	18,096		M	
2	12,396	13506584	336725	81,904		M	
Total		16490756	458201				

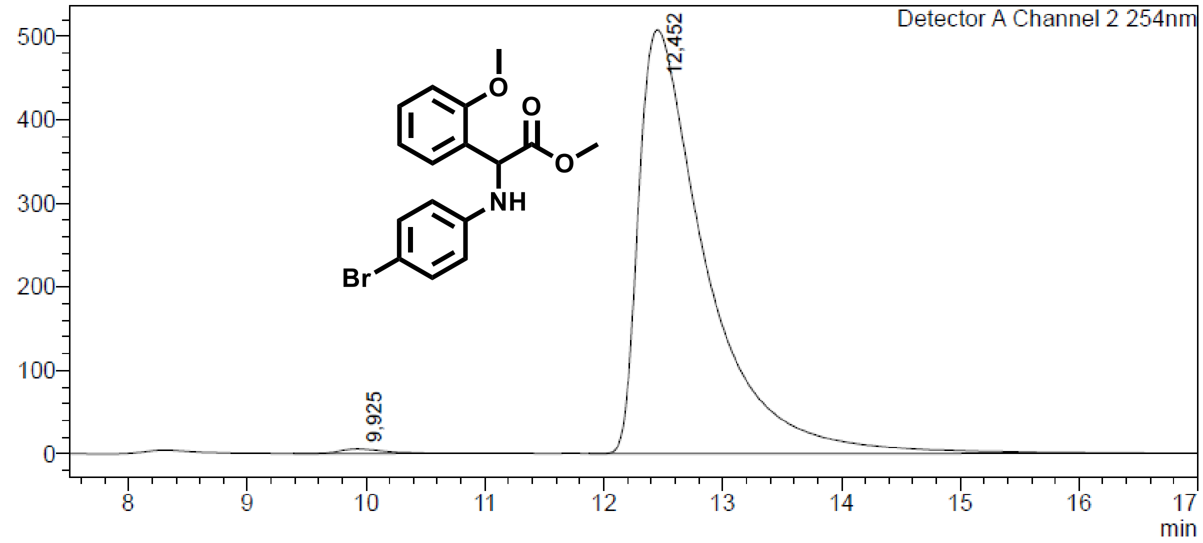

Detector A Channel 2 254nm

Peak#	Ret. Time	Area	Height	Conc.	Unit	Mark	Name
1	9,925	128659	5228	0,638		M	
2	12,452	20045601	507682	99,362		M	
Total		20174260	512910				

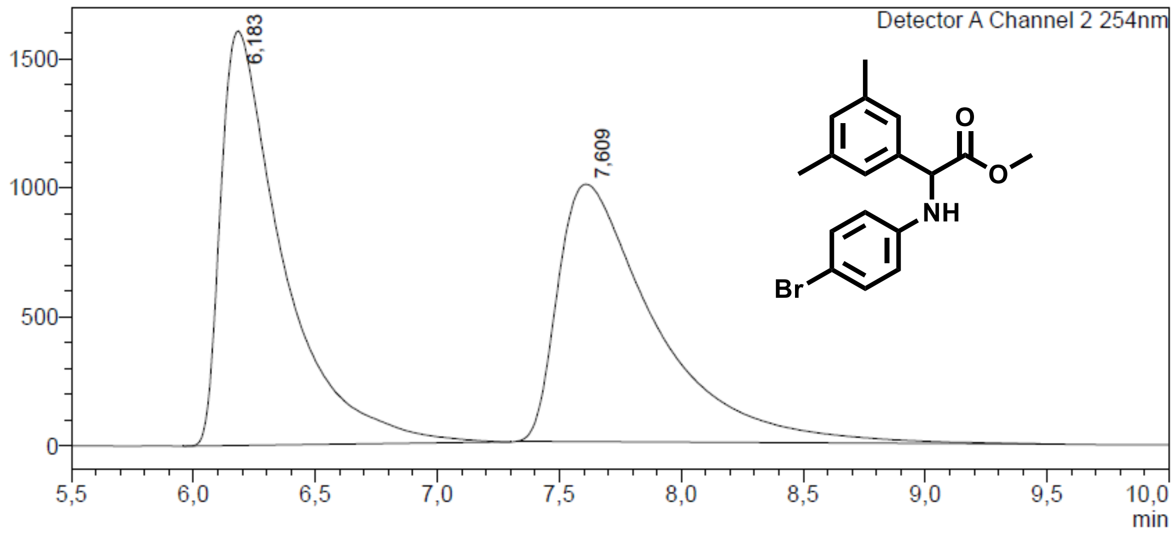

Detector A Channel 2 254nm

Peak#	Ret. Time	Area	Height	Conc.	Unit	Mark	Name
1	6,183	28423611	1604835	50,481		M	
2	7,609	27882420	997739	49,519		M	
Total		56306032	2602574				

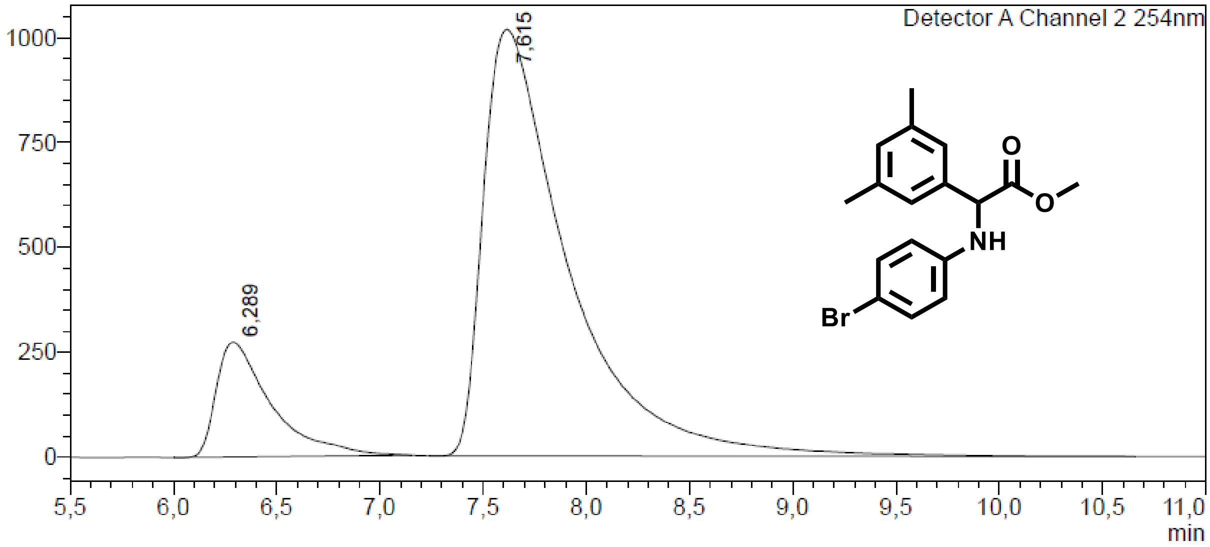

Detector A Channel 2 254nm

Peak#	Ret. Time	Area	Height	Conc.	Unit	Mark	Name
1	6,289	5051735	273304	14,757		M	
2	7,615	29181576	1016556	85,243		M	
Total		34233310	1289859				

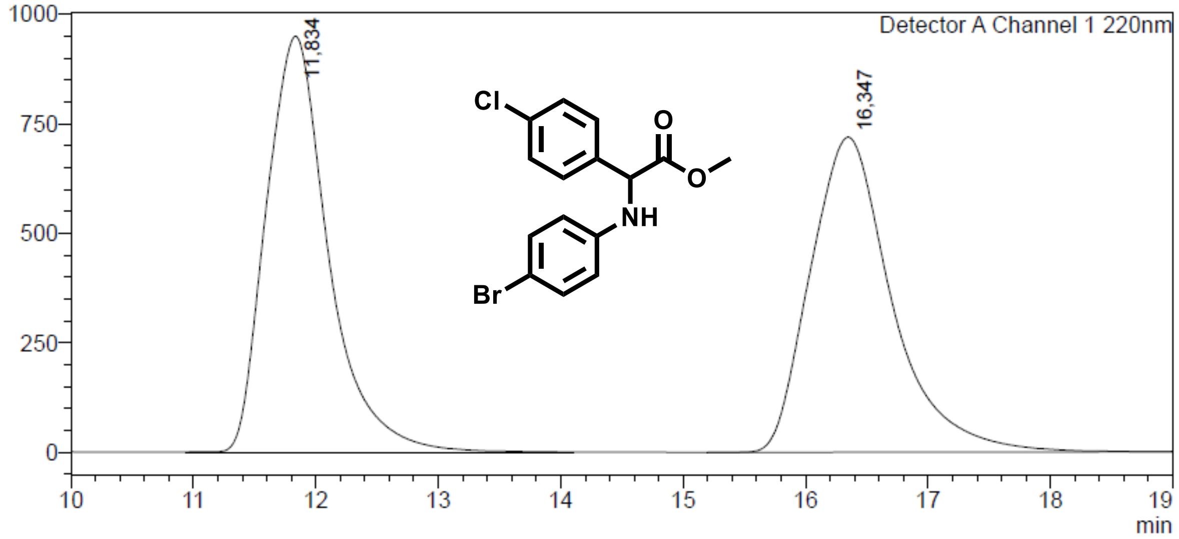

Peak#	Ret. Time	Area	Height	Conc.	Unit	Mark	Name
1	11,834	33494194	948843	50,120		M	
2	16,347	33333777	718964	49,880		M	
Total		66827971	1667808				

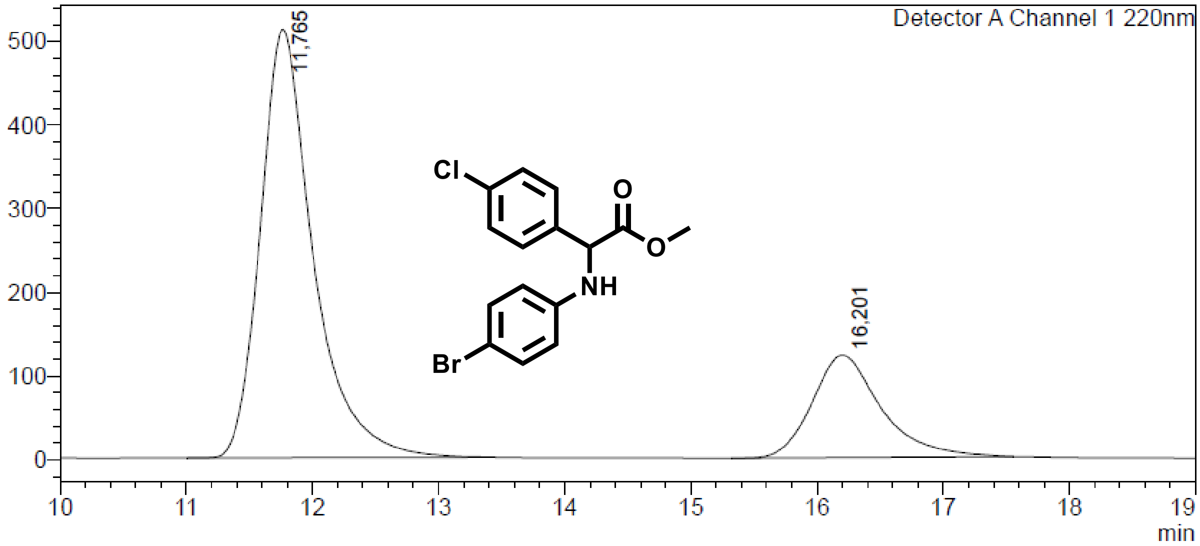

Peak#	Ret. Time	Area	Height	Conc.	Unit	Mark	Name
1	11,765	14925076	511577	76,310		M	
2	16,201	4633359	122285	23,690		M	
Total		19558435	633862				

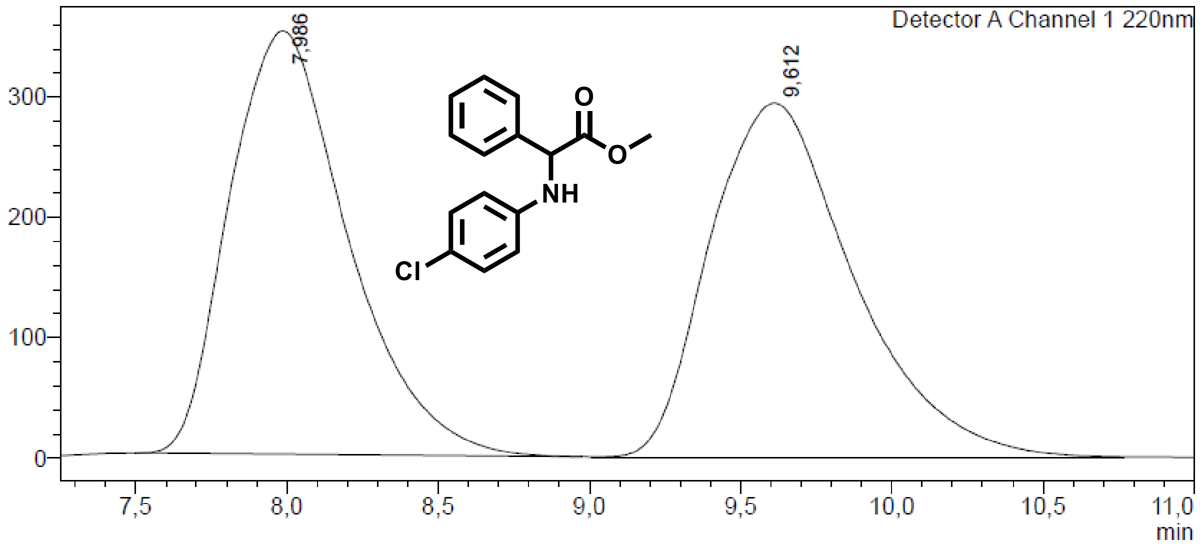

Peak#	Ret. Time	Area	Height	Conc.	Unit	Mark	Name
1	7,986	9599671	351724	49,700		M	
2	9,612	9715397	294056	50,300		M	
Total		19315069	645781				

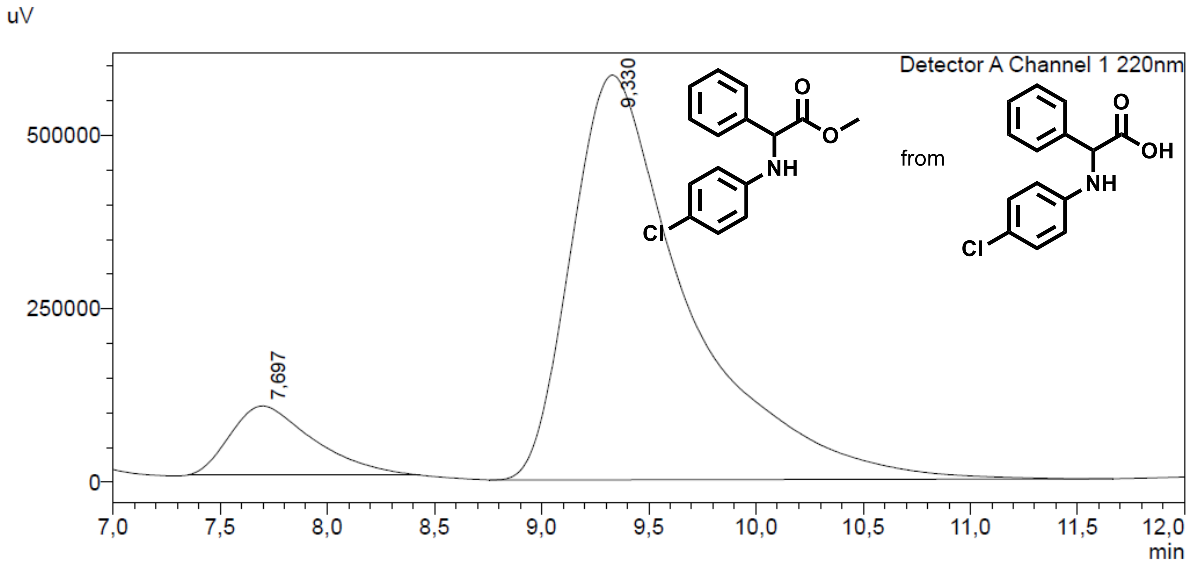

Peak#	Ret. Time	Area	Height	Conc.	Unit	Mark	Name
1	7,697	2691660	99556	10,799		M	
2	9,330	22234235	583953	89,201		M	
Total		24925895	683509				

8. References

1 L. G. Furniel and A. C. B. Burtoloso, *Tetrahedron*, 2020, **76**, 131313.

2 H. Konishi, T. Y. Lam, J. P. Malerich and V. H. Rawal, *Org. Lett.*, 2010, **12**, 2028–2031.

3 W. Yang and D.-M. Du, *Org. Lett.* 2010, **12**, 5450–5453.

4 X. Ni, X. Li, Z. Wang and J.-P. Cheng, *Org. Lett.*, 2014, **16**, 1786–1789.

5 J.-Y. Qian, C.-C. Wang, F. Sha and X.-Y. Wu, *RSC Advances*, 2012, **2**, 6042.

6 A. S. Kucherenko, A. A. Kostenko, A. N. Komogortsev, B. V. Lichitsky, M. Yu. Fedotov and S. G. Zlotin, *J. Org. Chem.*, 2019, **84**, 4304–4311.

7 R. R. Knowles, S. Lin and E. N. Jacobsen, *J. Am. Chem. Soc.*, 2010, **132**, 5030–5032.

8 A. G. Talero, B. S. Martins and A. C. B. Burtoloso, *Org. Lett.*, 2018, **20**, 7206–7211.
9 L. C. Morrill, T. Lebl, A. M. Z. Slawin and A. D. Smith, *Chem. Sci.*, 2012, **3**, 2088.
10 G. Amari, M. Riccaboni, US Pat., 20110311459A1, 2011.
11 K. H. Baggaley, R. Fears, H. Ferres, G. R. Geen, I. K. Hatton, L. J. A. Jennings and A. W. R. Tyrrell, *Eur. J. Med. Chem.*, 1988, **23**, 523–531.
